# Supplementary material for: Rare variants analyses suggest novel cleft genes in the African population
Source: Sci Rep. 2024 Jun 20;14:14279. doi: 10.1038/s41598-024-65151-9 (PMC11189897; doi:10.1038/s41598-024-65151-9)
Supplement: Supplementary file 1 — Supplementary Information. [file 41598_2024_65151_MOESM1_ESM.docx]

**Supplementary data**

**Supplementary Table 1: Showing reports on prioritized genes and NSOFCs.**

| **Prioritized genes** | **Previous report(s)** | **References** |
| --- | --- | --- |
| ***ABCB1*** | **Common variations in the ABCB1 gene are associated with NSOFCs** | **1** |
| ***TTC28*** | **Deletion overlapping the gene reported in patients with cleft palate** | **2** |
| ***PDZD8*** | **Pathogenic variations in the gene present with cleft palate among other clinical features** | **3** |
| ***CENPF*** | **Pathogenic gene variations associated with dysmorphic craniofacial features including cleft palate** | **4** |
| ***ALKBH8*** | **Gene mutations implicated in patients with cleft related phenotypes (high arched palate, thin upper lip)** | **5** |
| ***EXPH5*** | **Gene variant showed suggestive association with NSOFCs in gene by sex interaction analysis (using the same African GWAS data)** | **6** |
| ***CSAD*** | **Not previously associated with NSOFCs** |  |
| ***SLC16A9*** | **Not previously associated with NSOFCs** |  |

Sample Size and Power

Based on the published literature, effect sizes for rare variants associated with NSOFCs range from 1.0-4.0. Using SEQPower ^7^ with our fixed sample size of 3178 (1,019 cases vs 2,159 unrelated controls), we estimated statistical power for genome-wide analyses of rare variant aggregates using 10,000 genes (based on our preliminary results on genes with more than one rare variant in our data) at an alpha level of 0.05 (Bonferroni correction = 0.05/10000 = 5E-06). Estimates generated for rare variants with MAF <= 0.01 at odds ratios ranging from 1-4 under an additive genetic model are shown in Figure 3.1.

**Sample size and power estimation**

Plot of power against detectable odds ratio for 1019 cases and 2159 controls for rare


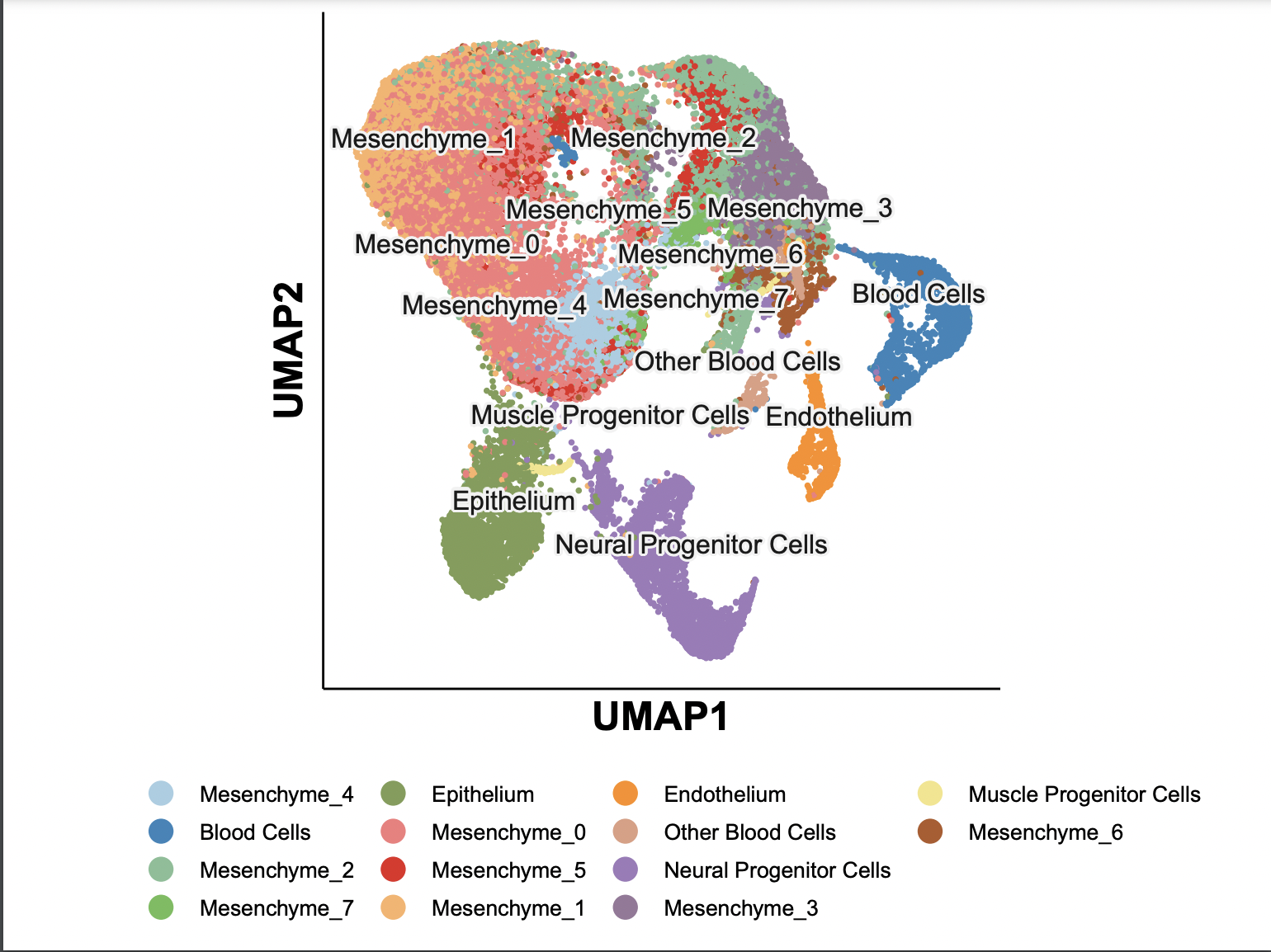


Supplementary Figure 1: UMAPs of CS17 human craniofacial tissues showing all the cell types.


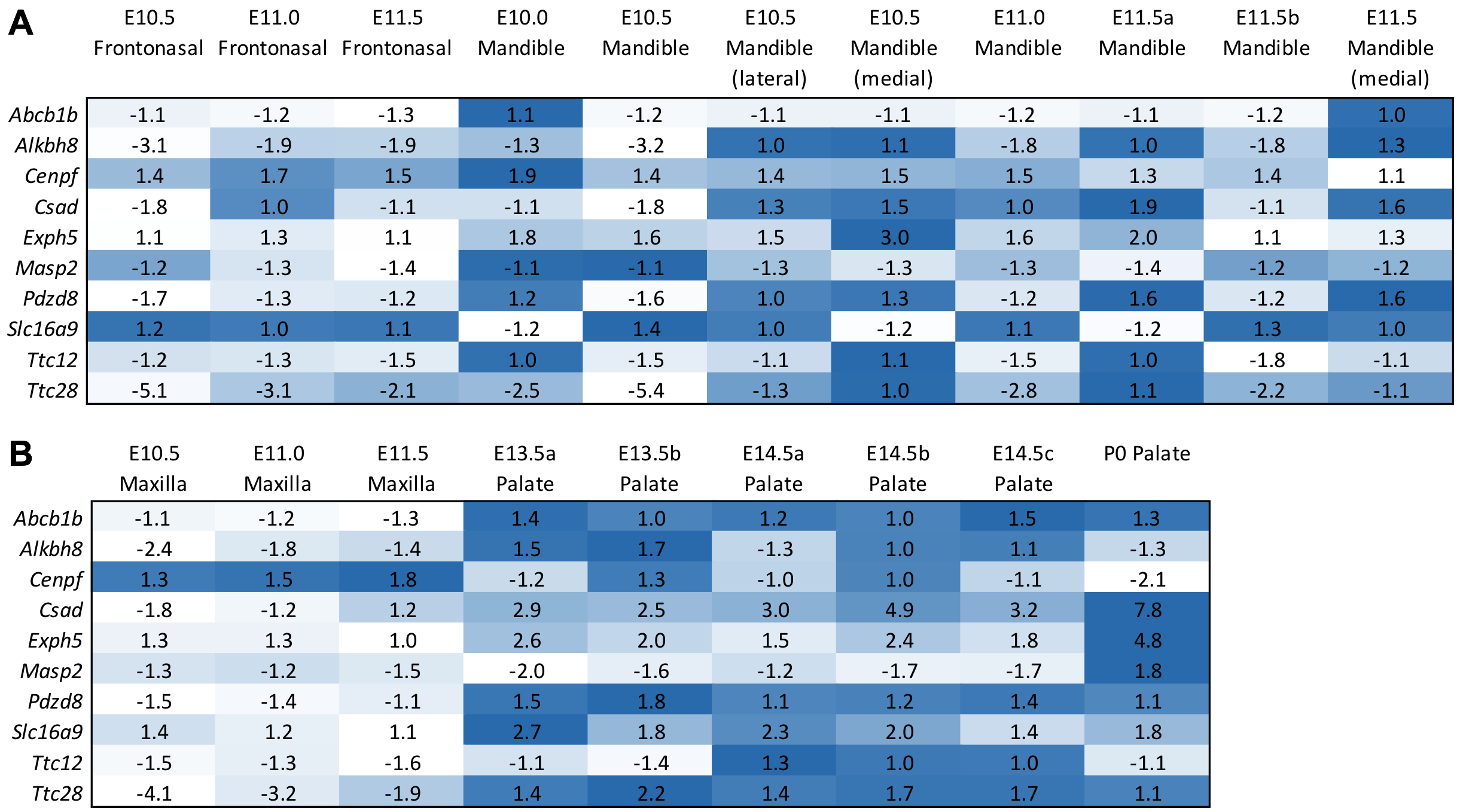


Supplementary Figure 2. SysFACE-based facial-tissue enriched expression analysis of candidate genes in mouse development. Mouse orthologs for 10 of the 13 human candidate genes were examined using the SysFACE tool that is based on microarray gene expression data on Affymetrix Mouse Genome 430 2.0 Array platform from isolated facial tissue in mouse development. Heat-map denotes the relative enriched expression in fold-change of individual genes at various stages of mouse embryonic (E) or postnatal (P) development in specific facial tissues, namely (A) frontonasal and mandible, and (B) maxilla and palate. The intensity of the color in the heat-map (row-wise) is representative of the extent of enriched-expression of the candidate gene expression compared to its expression in whole body embryonic tissue. Note that for palate, there were independent datasets for E13.5 and E14.5 and these are denoted as E13.5a, E13.5b, etc.

Supplementary Table 2: List of all included rare variants with their minor allele count in cases and controls.

CHR BP(GRCh37/hg19) rs effect_allele Non-effect allele gene Count_cases Count_controls

1 100732126 1:100732126-GA A G RTCA 3 10

1 100964793 1:100964793-CT A G CDC14A 5 1

1 101339871 1:101339871-TC G A EXTL2 1 3

1 103488374 1:103488374-TC G A COL11A1 9 11

1 10532436 1:10532436-TC G A DFFA 2 4

1 10719934 1:10719934-CA A C CASZ1 0 1

1 108735195 1:108735195-CT A G SLC25A24 9 27

1 110257647 1:110257647-TC G A GSTM5 11 19

1 110304359 1:110304359-TC G A EPS8L3 4 5

1 110466397 1:110466397-AG G A CSF1 9 20

1 111862042 1:111862042-TC G A CHIA 3 5

1 113253207 1:113253207-AC C A PPM1J 1 6

1 118039481 1:118039481-GA A G MAN1A2 6 18

1 118166001 1:118166001-GA A G TENT5C 7 16

1 118482149 1:118482149-CT A G WDR3 3 10

1 119958047 1:119958047-GT A C HSD3B2 15 23

1 119958174 1:119958174-GT A C HSD3B2 0 2

1 120345651 1:120345651-CT A G REG4 10 17

1 120378722 1:120378722-CT A G NBPF7 13 40

1 12371900 1:12371900-AG G A VPS13D 0 4

1 12779564 1:12779564-GA A G AADACL3 3 6

1 1309490 1:1309490-CT A G AURKAIP1 5 5

1 14108321 1:14108321-AG G A PRDM2 3 9

1 144879386 1:144879386-CT A G PDE4DIP 6 7

1 149906167 1:149906167-TG C A MTMR11 4 7

1 149906992 1:149906992-GA A G MTMR11 1 0

1 150116978 1:150116978-GA A G VPS45 10 15

1 151499468 1:151499468-TC G A CGN 8 20

1 151739327 1:151739327-AG G A OAZ3 4 7

1 152058140 1:152058140-GA A G TCHHL1 2 9

1 152059809 1:152059809-CT A G TCHHL1 8 23

1 152083114 1:152083114-TC G A TCHH 1 3

1 152277415 1:152277415-GC G C FLG 7 30

1 152282590 1:152282590-CA A C FLG 18 30

1 152692217 1:152692217-TC G A C1orf68 9 36

1 154061911 1:154061911-GA A G NUP210L 0 1

1 155015891 1:155015891-CT A G DCST1 2 10

1 155296421 1:155296421-GA A G RUSC1 12 23

1 155447868 1:155447868-GA A G ASH1L 8 41

1 155911828 1:155911828-GA A G RXFP4 3 5

1 156640498 1:156640498-CT A G NES 5 9

1 156821942 1:156821942-CT A G INSRR 3 7

1 15686990 1:15686990-GA A G FHAD1 12 29

1 156874544 1:156874544-AG G A PEAR1 18 35

1 156879557 1:156879557-AG G A PEAR1 14 29

1 15702189 1:15702189-CT A G FHAD1 4 10

1 157659701 1:157659701-GT A C FCRL3 1 4

1 158390380 1:158390380-AG G A OR10K2 23 49

1 158644160 1:158644160-CT A G SPTA1 1 6

1 15890542 1:15890542-GA A G DNAJC16 2 5

1 159176061 1:159176061-G-A A G ACKR1 0 1

1 159283537 1:159283537-GA A G OR10J3 11 20

1 159858188 1:159858188-AG G A CFAP45 5 7

1 159906278 1:159906278-CT A G IGSF9 2 3

1 160722032 1:160722032-GA A G SLAMF7 10 25

1 16258302 1:16258302-AG G A SPEN 2 1

1 16528284 1:16528284-CA A C ARHGEF19 18 36

1 167663486 1:167663486-CT A G RCSD1 1 2

1 167830254 1:167830254-TC G A ADCY10 1 3

1 168698160 1:168698160-CT A G DPT 3 9

1 169951978 1:169951978-AC C A KIFAP3 23 34

1 170136860 1:170136860-GA A G METTL11B 5 4

1 171031327 1:171031327-TC G A MROH9 1 4

1 171086559 1:171086559-GA A G FMO3 0 1

1 171511200 1:171511200-AG G A PRRC2C 10 22

1 171553294 1:171553294-GA A G PRRC2C 0 6

1 171605595 1:171605595-CT A G MYOC 17 38

1 17413096 1:17413096-CT A G PADI2 0 1

1 175092680 1:175092680-GA A G TNN 6 15

1 175375513 1:175375513-GA A G TNR 3 11

1 17599856 1:17599856-GA A G PADI3 6 14

1 17668888 1:17668888-AG G A PADI4 8 27

1 176708831 1:176708831-GA A G PAPPA2 2 4

1 17708528 1:17708528-GA A G PADI6 22 32

1 178834515 1:178834515-GA A G ANGPTL1 2 1

1 17928671 1:17928671-GA A G ARHGEF10L 1 4

1 180059860 1:180059860-CA A C CEP350 3 10

1 180063108 1:180063108-TC G A CEP350 0 1

1 180163513 1:180163513-AG G A QSOX1 0 1

1 181686261 1:181686261-GT A C CACNA1E 1 4

1 181702616 1:181702616-GA A G CACNA1E 26 49

1 182026812 1:182026812-CT A G ZNF648 10 24

1 182443520 1:182443520-GA A G RGSL1 8 20

1 182571619 1:182571619-AC C A RGS16 6 11

1 183196028 1:183196028-GA A G LAMC2 5 13

1 184764964 1:184764964-GA A G NIBAN1 2 7

1 185880877 1:185880877-GA A G HMCN1 9 23

1 186052024 1:186052024-GA A G HMCN1 11 26

1 186092230 1:186092230-GA A G HMCN1 2 8

1 186277690 1:186277690-AG G A PRG4 2 5

1 1897917 1:1897917-CT A G CFAP74 1 3

1 19611537 1:19611537-CA A C AKR7A3 9 13

1 196748972 1:196748972-AG G A CFHR3 7 16

1 196749027 1:196749027-CA A C CFHR3 3 8

1 196887457 1:196887457-GA A G CFHR4 28 57

1 196952188 1:196952188-TC G A CFHR5 7 22

1 197062204 1:197062204-CT A G ASPM 2 9

1 197704779 1:197704779-TC G A DENND1B 2 7

1 20027268 1:20027268-AG G A TMCO4 5 15

1 200569581 1:200569581-TC G A KIF14 0 1

1 200586864 1:200586864-CT A G KIF14 2 1

1 200635333 1:200635333-TC G A DDX59 12 32

1 20067395 1:20067395-CT A G TMCO4 12 33

1 20073656 1:20073656-CT A G TMCO4 9 14

1 200817621 1:200817621-TC G A CAMSAP2 0 2

1 201175222 1:201175222-AG G A IGFN1 3 8

1 201181403 1:201181403-GA A G IGFN1 10 15

1 204210936 1:204210936-CT A G PLEKHA6 0 8

1 205884226 1:205884226-CA A C SLC26A9 1 2

1 205897044 1:205897044-CT A G SLC26A9 11 20

1 20645117 1:20645117-CA A C VWA5B1 14 31

1 207648456 1:207648456-GT A C CR2 3 3

1 207857222 1:207857222-GA A G CR1L 4 8

1 207891012 1:207891012-CT A G CR1L 14 33

1 213303019 1:213303019-GT A C RPS6KC1 14 35

1 213414441 1:213414441-GA A G RPS6KC1 1 2

1 214820457 1:214820457-AC C A CENPF 18 34

1 215901460 1:215901460-CT A G USH2A 4 11

1 215953266 1:215953266-TC G A USH2A 14 32

1 215990470 1:215990470-AG G A USH2A 1 0

1 216052206 1:216052206-CT A G USH2A 18 24

1 216173883 1:216173883-TC G A USH2A 0 3

1 220330827 1:220330827-CT A G RAB3GAP2 1 0

1 22174247 1:22174247-CT A G HSPG2 0 1

1 22192290 1:22192290-CT A G HSPG2 2 2

1 222838660 1:222838660-TC G A MIA3 4 21

1 223116172 1:223116172-AG G A DISP1 13 27

1 225239186 1:225239186-AG G A DNAH14 2 3

1 225446838 1:225446838-GA A G DNAH14 20 41

1 225495168 1:225495168-GA A G DNAH14 7 19

1 225565066 1:225565066-CA A C DNAH14 1 5

1 225576087 1:225576087-GA A G DNAH14 2 4

1 225586274 1:225586274-GT A C DNAH14 26 39

1 226019631 1:226019631-GA A G EPHX1 1 1

1 227149148 1:227149148-CT A G COQ8A 1 2

1 228459677 1:228459677-CT A G OBSCN 0 1

1 228469642 1:228469642-GA A G OBSCN 1 0

1 228492186 1:228492186-CA A C OBSCN 14 20

1 228493064 1:228493064-CT A G OBSCN 10 17

1 228511220 1:228511220-GA A G OBSCN 2 3

1 228529159 1:228529159-GA A G OBSCN 1 0

1 228596825 1:228596825-CT A G TRIM17 3 13

1 22902827 1:22902827-GA A G EPHA8 35 39

1 229770955 1:229770955-GA A G URB2 8 24

1 230895279 1:230895279-CT A G CAPN9 0 2

1 231906698 1:231906698-CT A G DISC1 0 2

1 232564162 1:232564162-TC G A SIPA1L2 9 17

1 233807173 1:233807173-CT A G KCNK1 1 0

1 235907403 1:235907403-CA A C LYST 7 12

1 235922666 1:235922666-CT A G LYST 1 3

1 235966237 1:235966237-TC G A LYST 22 34

1 236750727 1:236750727-CT A G HEATR1 7 15

1 237881790 1:237881790-TC G A RYR2 1 2

1 23842943 1:23842943-GA A G E2F2 12 22

1 242253227 1:242253227-AG G A PLD5 6 7

1 243504395 1:243504395-AG G A SDCCAG8 1 4

1 24447534 1:24447534-GT A C IL22RA1 1 9

1 244803370 1:244803370-GA A G CATSPERE 3 6

1 247614593 1:247614593-CA A C OR2B11 1 7

1 248004442 1:248004442-CT A G OR11L1 11 16

1 248129325 1:248129325-C-G C G OR2AK2 1 2

1 248366733 1:248366733-CT A G OR2M3 12 22

1 248524941 1:248524941-GA A G OR2T4 3 18

1 248525847 1:248525847-AG G A OR2T4 12 22

1 248813317 1:248813317-TC G A OR2T27 15 21

1 26695062 1:26695062-TC G A ZNF683 3 14

1 27874606 1:27874606-AC C A AHDC1 27 58

1 27877180 1:27877180-CT A G AHDC1 4 6

1 29314121 1:29314121-GA A G EPB41 3 7

1 2939155 1:2939155-CT A G ACTRT2 2 9

1 3102700 1:3102700-GA A G PRDM16 14 24

1 31230583 1:31230583-GA A G LAPTM5 0 2

1 32098801 1:32098801-TC G A PEF1 6 9

1 32673279 1:32673279-CT A G IQCC 0 2

1 32740924 1:32740924-GA A G LCK 16 19

1 33944890 1:33944890-AG G A ZSCAN20 10 22

1 34204903 1:34204903-CT A G CSMD2 8 21

1 35227390 1:35227390-CT A G GJB4 0 1

1 35227625 1:35227625-CT A G GJB4 1 2

1 35453058 1:35453058-GA A G ZMYM6 1 2

1 36205008 1:36205008-GA A G CLSPN 1 3

1 36230135 1:36230135-TC G A CLSPN 1 1

1 36553732 1:36553732-GT A C TEKT2 0 2

1 37307528 1:37307528-CT A G GRIK3 1 1

1 38079517 1:38079517-TG C A RSPO1 24 28

1 38483420 1:38483420-AG G A UTP11 2 6

1 40776916 1:40776916-GT A C COL9A2 11 27

1 40960943 1:40960943-TG C A ZFP69 3 5

1 43675553 1:43675553-AG G A CFAP57 6 9

1 45796198 1:45796198-CT A G MUTYH 2 8

1 48698061 1:48698061-CT A G SLC5A9 0 2

1 48708250 1:48708250-CT A G SLC5A9 18 33

1 48713100 1:48713100-CA A C SLC5A9 13 32

1 50317124 1:50317124-CA A C AGBL4 13 21

1 51929454 1:51929454-TC G A EPS15 20 44

1 52825505 1:52825505-CT A G CC2D1B 2 3

1 53553885 1:53553885-GA A G SLC1A7 10 21

1 53974903 1:53974903-CT A G GLIS1 1 1

1 54534466 1:54534466-CA A C TCEANC2 7 11

1 55183212 1:55183212-AG G A TTC4 15 35

1 55186871 1:55186871-GA A G TTC4 2 8

1 55451977 1:55451977-GA A G TMEM61 5 12

1 55474261 1:55474261-CT A G BSND 0 1

1 55523822 1:55523822-GA A G PCSK9 1 6

1 57411587 1:57411587-GA A G C8B 1 3

1 60392303 1:60392303-CT A G CYP2J2 2 13

1 60521016 1:60521016-GT A C C1orf87 2 5

1 62673193 1:62673193-GA A G L1TD1 1 3

1 62676659 1:62676659-TC G A L1TD1 21 32

1 65047888 1:65047888-AG G A CACHD1 11 22

1 6638905 1:6638905-TC G A TAS1R1 3 18

1 67147839 1:67147839-CT A G SGIP1 5 7

1 67370898 1:67370898-TC G A WDR78 1 4

1 67371055 1:67371055-GT A C WDR78 4 7

1 67861270 1:67861270-GT A C IL12RB2 3 7

1 74929131 1:74929131-CA A C FPGT-TNNI3K;TNNI3K 1 3

1 75038984 1:75038984-CT A G ERICH3 3 11

1 76313960 1:76313960-CT A G MSH4 1 2

1 7895966 1:7895966-CT A G PER3 6 11

1 79093698 1:79093698-AC C A IFI44L 10 24

1 84982065 1:84982065-CT A G SPATA1 1 3

1 85136332 1:85136332-AC C A SSX2IP 4 8

1 86947950 1:86947950-CT A G CLCA1 3 11

1 86961313 1:86961313-CA A C CLCA1 1 1

1 87538689 1:87538689-CT A G HS2ST1 3 7

1 881602 1:881602-CT A G NOC2L 6 14

1 889451 1:889451-GA A G NOC2L 1 2

1 89477478 1:89477478-CT A G GBP3 18 27

1 89575391 1:89575391-TC G A GBP2 7 15

1 91403089 1:91403089-CT A G ZNF644 1 2

1 92646551 1:92646551-CT A G KIAA1107 2 11

1 92647960 1:92647960-GA A G KIAA1107 17 30

1 92979460 1:92979460-TC G A EVI5 2 4

1 93722055 1:93722055-CT A G CCDC18 10 24

1 94341986 1:94341986-TG C A DNTTIP2 5 8

1 9931307 1:9931307-CT A G CTNNBIP1 0 1

1 152282533 exm100068 A G FLG 4 12

1 6649228 exm10030 C A ZBTB48 4 8

1 152324559 exm100676 G C FLG2 2 6

1 152329951 exm100965 A G FLG2 14 35

1 152331330 exm100975 A G FLG2 26 44

1 152382312 exm100986 A G CRNN 12 21

1 152383382 exm101023 A G CRNN 27 54

1 152733275 exm101386 A G KPRP 0 3

1 152733472 exm101400 A G KPRP 10 18

1 152883207 exm101701 A C IVL 0 4

1 152883702 exm101747 A G IVL 15 41

1 153270461 exm102037 C A PGLYRP3 7 9

1 153271621 exm102045 A G PGLYRP3 7 6

1 153271682 exm102048 A G PGLYRP3 9 17

1 153317699 exm102165 A G PGLYRP4 1 5

1 153317723 exm102168 G A PGLYRP4 10 29

1 153507812 exm102325 A G S100A6 1 1

1 153614810 exm102500 A G CHTOP 3 10

1 153617603 exm102527 G C CHTOP 3 8

1 153656228 exm102651 A C NPR1 3 13

1 153658297 exm102667 A G NPR1 1 5

1 153659550 exm102680 G A NPR1 1 4

1 153660154 exm102693 C G NPR1 14 19

1 153749062 exm102911 C G SLC27A3 8 11

1 153750664 exm102945 A G SLC27A3 13 25

1 153751267 exm102981 A G SLC27A3 1 2

1 6705944 exm10307 A G DNAJC11 1 6

1 6706030 exm10310 A G DNAJC11 2 4

1 153905788 exm103136 A G DENND4B 3 3

1 153906179 exm103145 A G DENND4B 7 20

1 153946189 exm103465 C G CREB3L4 1 2

1 153954646 exm103508 A G RAB13 13 14

1 153998032 exm103625 G A NUP210L 6 25

1 154018835 exm103643 C A NUP210L 3 7

1 154076490 exm103705 G A NUP210L 2 2

1 154110654 exm103741 A G NUP210L 20 37

1 154173005 exm103826 A G C1orf189 1 5

1 154178081 exm103831 A G C1orf189 9 15

1 154247666 exm104042 A G HAX1 2 3

1 154248166 exm104058 A G HAX1 0 1

1 7723991 exm10416 A G CAMTA1 3 5

1 154461587 exm104368 A G SHE 5 11

1 7724513 exm10443 G A CAMTA1 2 9

1 7724562 exm10446 A G CAMTA1 0 1

1 154918369 exm104853 A G PBXIP1 1 1

1 154920822 exm104923 A G PBXIP1 22 33

1 154960848 exm105101 A G FLAD1 0 3

1 154961241 exm105124 A G FLAD1 7 17

1 154962678 exm105138 A G FLAD1 1 2

1 154962679 exm105139 A G FLAD1 3 11

1 7797627 exm10547 G C CAMTA1 9 30

1 155018436 exm105491 G A DCST1 11 25

1 155019711 exm105506 G A DCST1 4 17

1 155025945 exm105541 A T ADAM15 14 19

1 7798367 exm10557 A G CAMTA1 17 29

1 7798493 exm10562 A G CAMTA1 0 2

1 7798520 exm10563 A G CAMTA1 5 14

1 155159955 exm105931 A C MUC1 2 6

1 155160049 exm105936 A G MUC1 15 22

1 155162050 exm105968 A C MUC1 5 2

1 155205047 exm106204 A G GBA 16 21

1 7848255 exm10658 A G PER3 3 4

1 155300291 exm106866 A C RUSC1 9 16

1 155385662 exm106972 A G ASH1L 10 21

1 155449342 exm107052 G A ASH1L 36 68

1 155450938 exm107094 C A ASH1L 4 8

1 155649223 exm107334 C G YY1AP1 1 1

1 7886750 exm10759 A G PER3 10 20

1 155823151 exm107677 A G GON4L 1 1

1 155823496 exm107688 A G GON4L 0 1

1 7887499 exm10782 C A PER3 11 16

1 155912426 exm107884 G A RXFP4 15 34

1 155921320 exm107927 A G ARHGEF2 8 8

1 155932915 exm107997 A G ARHGEF2 0 1

1 156132745 exm108352 A G SEMA4A 1 5

1 156145436 exm108389 A G SEMA4A 1 6

1 156146751 exm108417 T A SEMA4A 5 11

1 7895841 exm10846 G A PER3 1 0

1 156220455 exm108606 C G SMG5 1 4

1 156237971 exm108697 C A SMG5 11 28

1 156255162 exm108752 A G TMEM79 13 24

1 156256195 exm108807 G A TMEM79 4 18

1 156256257 exm108811 A G TMEM79 9 18

1 156504372 exm109264 G C IQGAP3 4 10

1 156507067 exm109286 A G IQGAP3 0 1

1 156526327 exm109389 C G IQGAP3 2 6

1 156536256 exm109452 C A IQGAP3 1 2

1 156566195 exm109626 G A GPATCH4 13 18

1 156571216 exm109655 A G GPATCH4 4 13

1 156639545 exm109848 A G NES 16 29

1 156641200 exm109919 A T NES 3 15

1 156642158 exm109955 G A NES 1 1

1 8073943 exm11015 A G ERRFI1 16 39

1 8074094 exm11021 G A ERRFI1 18 40

1 156823801 exm110595 A C INSRR 0 1

1 156838333 exm110669 A G NTRK1 4 11

1 156843514 exm110692 A G NTRK1 5 8

1 156882654 exm110920 A G PEAR1 4 6

1 156907247 exm111080 G A ARHGEF11 5 12

1 156939114 exm111238 A G ARHGEF11 9 24

1 156954136 exm111266 A G ARHGEF11 4 7

1 157062575 exm111284 G A ETV3L 6 10

1 157509125 exm111498 C A FCRL5 12 16

1 157514228 exm111536 A G FCRL5 6 15

1 157551336 exm111584 A G FCRL4 13 17

1 157551405 exm111590 A G FCRL4 5 6

1 157556042 exm111602 G C FCRL4 4 6

1 157557721 exm111630 A G FCRL4 1 0

1 157559122 exm111648 A G FCRL4 3 3

1 157566136 exm111654 A G FCRL4 0 1

1 157648597 exm111672 G C FCRL3 8 21

1 157650828 exm111692 A G FCRL3 5 13

1 157659642 exm111704 A G FCRL3 5 15

1 157666047 exm111751 C G FCRL3 1 3

1 157667571 exm111779 G A FCRL3 1 0

1 157737113 exm111859 A G FCRL2 0 9

1 157804260 exm112025 A G CD5L 1 0

1 8403835 exm11203 A G SLC45A1 2 5

1 8403840 exm11205 A G SLC45A1 1 4

1 157809185 exm112054 C G CD5L 23 36

1 158298714 exm112365 A G CD1B 0 1

1 158300684 exm112412 A G CD1B 2 2

1 158325180 exm112452 A G CD1E 7 23

1 158368392 exm112502 A G OR10T2 0 1

1 158389863 exm112566 G A OR10K2 4 5

1 158435658 exm112667 G A OR10K1 15 29

1 158435866 exm112676 G A OR10K1 7 28

1 158436280 exm112703 G A OR10K1 5 13

1 158517376 exm112799 G A OR6Y1 4 4

1 158517865 exm112828 A G OR6Y1 0 1

1 158581131 exm112985 G A SPTA1 2 1

1 158587844 exm113027 A G SPTA1 17 34

1 158589121 exm113030 A G SPTA1 19 39

1 158612244 exm113134 T A SPTA1 17 33

1 158612618 exm113142 C G SPTA1 17 33

1 158612719 exm113148 A G SPTA1 2 2

1 158615349 exm113180 G A SPTA1 0 2

1 158618391 exm113197 A G SPTA1 1 1

1 158622402 exm113217 A G SPTA1 4 15

1 158623085 exm113222 A C SPTA1 6 8

1 158632660 exm113276 A G SPTA1 2 8

1 158644397 exm113344 G A SPTA1 27 67

1 158651447 exm113386 G A SPTA1 2 7

1 158655137 exm113407 A C SPTA1 1 0

1 158670025 exm113460 G A OR6K2 2 3

1 158670195 exm113467 A G OR6K2 4 11

1 158725348 exm113591 T A OR6K6 15 18

1 158746606 exm113674 C G OR6N2 8 17

1 158746612 exm113675 A G OR6N2 10 29

1 158747137 exm113704 G A OR6N2 0 1

1 158747296 exm113710 A G OR6N2 16 35

1 158817597 exm113789 A G MNDA 8 21

1 158817666 exm113794 A G MNDA 2 0

1 158988413 exm113912 G A IFI16 14 30

1 159175494 exm114098 A G ACKR1 1 7

1 159175884 exm114114 A G ACKR1 0 1

1 159283494 exm114173 G A OR10J3 26 56

1 159283642 exm114180 A G OR10J3 15 37

1 159283925 exm114188 G C OR10J3 14 27

1 159284083 exm114196 A G OR10J3 2 5

1 159284245 exm114202 G A OR10J3 16 33

1 159284295 exm114204 G A OR10J3 1 4

1 159505373 exm114308 A G OR10J5 1 4

1 159683683 exm114370 A G CRP 1 2

1 159683816 exm114382 G C CRP 0 1

1 159683856 exm114385 A G CRP 0 9

1 159799731 exm114551 A G SLAMF8 3 2

1 159802845 exm114582 A G SLAMF8 5 13

1 159846376 exm114695 A G CFAP45 18 25

1 159897559 exm114837 A C IGSF9 5 8

1 159898422 exm114861 A G IGSF9 4 8

1 160064833 exm115274 A G IGSF8 2 6

1 160123000 exm115432 A G ATP1A4 1 2

1 160133955 exm115468 A G ATP1A4 3 3

1 160134056 exm115477 A G ATP1A4 5 10

1 160136447 exm115492 A G ATP1A4 5 12

1 9009377 exm11550 G C CA6 6 10

1 160137113 exm115508 G A ATP1A4 4 11

1 160147429 exm115579 A T ATP1A4 5 7

1 160160675 exm115616 A C CASQ1 8 20

1 160160767 exm115622 A G CASQ1 18 38

1 160209860 exm115750 A G DCAF8 2 1

1 160210035 exm115757 A G DCAF8 2 6

1 160264641 exm115861 A G COPA 7 20

1 9034717 exm11617 G A CA6 3 7

1 160518056 exm116177 G A CD84 11 16

1 160518074 exm116180 A G CD84 5 8

1 160520774 exm116192 A G CD84 1 2

1 160535367 exm116230 A G CD84 6 8

1 160650997 exm116297 A G CD48 3 4

1 160654715 exm116315 A G CD48 14 42

1 160681531 exm116337 A G CD48 11 23

1 160719843 exm116369 C A SLAMF7 12 20

1 160769666 exm116415 A G LY9 2 10

1 160769866 exm116422 A G LY9 0 4

1 160784549 exm116461 T A LY9 8 12

1 160811558 exm116569 C G CD244 0 4

1 160968873 exm116750 A G F11R 23 38

1 160969752 exm116765 A G F11R 11 23

1 160970535 exm116784 G A F11R 10 16

1 9074861 exm11686 A G SLC2A7 34 54

1 161017761 exm116882 A G ARHGAP30 16 42

1 9075221 exm11699 A G SLC2A7 7 17

1 161024455 exm117007 G A ARHGAP30 1 8

1 161042515 exm117027 A C NECTIN4 1 4

1 161042657 exm117037 A G NECTIN4 0 3

1 161049509 exm117086 A C NECTIN4 7 22

1 161068749 exm117112 A G KLHDC9 22 32

1 161089650 exm117188 A G NIT1 5 13

1 9086388 exm11731 A G SLC2A7 14 25

1 9097966 exm11750 A G SLC2A5 1 7

1 161180473 exm117631 G A NDUFS2 1 3

1 161182208 exm117637 G C NDUFS2 15 41

1 982722 exm1179 G A AGRN 8 12

1 9101826 exm11794 A G SLC2A5 2 5

1 161677073 exm118319 A G FCRLA 4 10

1 161680583 exm118327 C G FCRLA 3 1

1 161681238 exm118351 A C FCRLA 10 14

1 161681736 exm118357 A G FCRLA 4 9

1 161692468 exm118394 C G FCRLB 8 18

1 161697319 exm118456 A G FCRLB 7 15

1 161697382 exm118458 A G FCRLB 1 0

1 161736209 exm118521 G A ATF6 0 1

1 161748100 exm118528 G A ATF6 2 10

1 161816280 exm118585 A G ATF6 4 7

1 161967722 exm118671 A G OLFML2B 4 8

1 161967881 exm118674 A T OLFML2B 16 34

1 161967974 exm118680 A C OLFML2B 14 27

1 161970046 exm118700 G A OLFML2B 0 1

1 161987190 exm118723 C G OLFML2B 6 12

1 161993187 exm118753 A C OLFML2B 2 6

1 162344365 exm118863 A G SPATA46 2 5

1 162345160 exm118872 G C SPATA46 13 25

1 162740121 exm119114 A G DDR2 1 1

1 162740180 exm119117 A G DDR2 17 33

1 9307061 exm11941 A G H6PD 9 22

1 9307089 exm11944 A G H6PD 4 2

1 165175206 exm119445 C A LMX1A 8 12

1 165182976 exm119458 A G LMX1A 3 10

1 983005 exm1196 A G AGRN 2 3

1 165632360 exm119642 A G ALDH9A1 5 4

1 165634336 exm119650 A G ALDH9A1 17 26

1 165648710 exm119668 A G ALDH9A1 17 29

1 165664552 exm119705 G A ALDH9A1 9 10

1 165667714 exm119717 A G ALDH9A1 9 21

1 166908766 exm119985 A G ILDR2 9 13

1 166958715 exm120017 G C MAEL 10 13

1 166990359 exm120060 G A MAEL 0 2

1 9323885 exm12008 A G H6PD 11 22

1 167023590 exm120081 A G GPA33 3 2

1 167032908 exm120109 A C GPA33 3 3

1 167042765 exm120143 A G GPA33 9 15

1 167059511 exm120147 G A GPA33 1 2

1 9324233 exm12048 A G H6PD 1 1

1 167653143 exm120545 A G RCSD1 8 27

1 167666892 exm120622 A G RCSD1 10 38

1 167666981 exm120630 C G RCSD1 2 4

1 167778926 exm120664 G A ADCY10 0 1

1 167778979 exm120667 T A ADCY10 0 1

1 167780103 exm120682 G C ADCY10 3 20

1 167787475 exm120694 T A ADCY10 3 5

1 167791328 exm120696 G A ADCY10 5 7

1 167805621 exm120744 A G ADCY10 7 20

1 167847696 exm120828 A G ADCY10 19 26

1 168066072 exm121064 A G GPR161 3 9

1 168066205 exm121073 A G GPR161 14 30

1 168066400 exm121085 A G GPR161 3 5

1 169345883 exm121497 G A BLZF1 7 16

1 169356298 exm121541 T A BLZF1 10 22

1 169483561 exm121681 G A F5 25 40

1 169505880 exm121765 A T F5 2 6

1 169509923 exm121781 G A F5 14 21

1 169511071 exm121850 G A F5 1 3

1 169511117 exm121855 A G F5 13 20

1 169511389 exm121862 A C F5 20 36

1 169511429 exm121868 A G F5 3 2

1 169511878 exm121903 C A F5 31 52

1 169521853 exm121977 G A F5 32 51

1 169559396 exm122039 A G SELP 1 1

1 169562881 exm122045 A G SELP 5 4

1 169563951 exm122058 C A SELP 8 26

1 169578810 exm122119 T A SELP 0 3

1 169586577 exm122190 A G SELP 0 5

1 169695945 exm122254 A G SELE 5 20

1 169697312 exm122282 A G SELE 2 11

1 169698647 exm122297 A G SELE 14 36

1 169701892 exm122329 A C SELE 1 3

1 169702116 exm122337 A C SELE 15 22

1 169761910 exm122371 G C METTL18 3 11

1 169775208 exm122456 G A C1orf112 2 4

1 169812828 exm122563 A G C1orf112 6 13

1 169821055 exm122590 C A C1orf112 5 15

1 169823790 exm122617 C G SCYL3 17 49

1 169824981 exm122635 G A SCYL3 1 5

1 169825074 exm122639 A G SCYL3 1 2

1 169825081 exm122640 A G SCYL3 24 44

1 169951967 exm122706 T A KIFAP3 1 2

1 170115331 exm122764 C A METTL11B 4 7

1 170521163 exm122841 G A GORAB 3 10

1 170965700 exm122981 A G MROH9 13 26

1 171083398 exm123079 G A FMO3 14 31

1 171085414 exm123088 A G FMO3 2 9

1 171173160 exm123164 A G FMO2 0 12

1 171177968 exm123195 C A FMO2 8 17

1 171250000 exm123239 G A FMO1 2 7

1 171250049 exm123243 G A FMO1 8 13

1 171251367 exm123259 A G FMO1 4 26

1 171310562 exm123337 C A FMO4 13 18

1 171310844 exm123352 C A FMO4 4 16

1 171501752 exm123405 G A PRRC2C 0 2

1 171509486 exm123447 A G PRRC2C 13 26

1 171510590 exm123484 A G PRRC2C 7 11

1 171557600 exm123622 G A PRRC2C 1 1

1 171605081 exm123635 G A MYOC 3 5

1 172411374 exm123933 A G PIGC 1 4

1 172411702 exm123945 A G PIGC 1 3

1 173494045 exm124310 A G SLC9C2 5 11

1 173628509 exm124417 A C ANKRD45 14 22

1 173628536 exm124420 G C ANKRD45 10 41

1 173772210 exm124480 G A CENPL 16 36

1 173842630 exm124649 A C ZBTB37 5 9

1 173842735 exm124651 A G ZBTB37 2 2

1 173930910 exm124767 G A RC3H1 2 2

1 175046557 exm125033 A C TNN 11 22

1 175049379 exm125097 A G TNN 9 22

1 175049514 exm125102 A G TNN 8 11

1 175097863 exm125251 A G TNN 3 21

1 175113607 exm125275 A G TNN 4 9

1 175116207 exm125284 G A TNN 3 8

1 175323562 exm125351 A G TNR 1 4

1 175325553 exm125368 A G TNR 10 14

1 175331892 exm125385 G A TNR 1 6

1 175336392 exm125422 A G TNR 11 17

1 175348819 exm125436 C A TNR 2 1

1 175365772 exm125475 A G TNR 1 5

1 175375540 exm125517 G A TNR 1 4

1 176564092 exm125662 A G PAPPA2 16 36

1 176564113 exm125663 A G PAPPA2 6 11

1 176659510 exm125700 A G PAPPA2 31 60

1 176709189 exm125791 G C PAPPA2 5 7

1 176760483 exm125827 A G PAPPA2 11 22

1 9801233 exm12583 A G CLSTN1 9 19

1 176903378 exm125907 G A ASTN1 13 34

1 176915101 exm125928 G A ASTN1 14 29

1 176918480 exm125941 A C ASTN1 4 11

1 176926872 exm125950 A G ASTN1 2 6

1 176999963 exm126001 G C ASTN1 3 4

1 9804525 exm12604 G A CLSTN1 6 18

1 177199049 exm126060 A G BRINP2 1 1

1 177242718 exm126091 G A BRINP2 5 14

1 9804623 exm12610 G A CLSTN1 11 18

1 177245423 exm126101 A G BRINP2 11 27

1 9804630 exm12612 G A CLSTN1 13 23

1 177250549 exm126164 A G BRINP2 0 1

1 177906633 exm126233 C A CRYZL2P-SEC16B;SEC16B 29 49

1 177934291 exm126316 A G CRYZL2P-SEC16B;SEC16B 26 55

1 177937038 exm126333 T A CRYZL2P-SEC16B;SEC16B 7 20

1 177937065 exm126335 A G CRYZL2P-SEC16B;SEC16B 21 37

1 178483868 exm126482 A G TEX35 0 3

1 9815287 exm12650 A G CLSTN1 3 8

1 178489858 exm126514 G A TEX35 21 31

1 178834112 exm126611 A G ANGPTL1 11 25

1 179013058 exm126701 A G FAM20B 10 15

1 9931246 exm12673 A C CTNNBIP1 10 23

1 179041137 exm126746 G A FAM20B 9 26

1 179064123 exm126823 A G TOR3A 1 1

1 9932101 exm12683 A G CTNNBIP1 2 8

1 179064279 exm126839 A C TOR3A 3 10

1 179320578 exm127073 G A SOAT1 7 7

1 179338048 exm127089 A G AXDND1 3 13

1 179363143 exm127127 A C AXDND1 12 17

1 179364256 exm127129 G A AXDND1 0 1

1 179638476 exm127405 A T TDRD5 1 0

1 179965828 exm127641 A G CEP350 9 12

1 179965962 exm127646 A G CEP350 4 12

1 179966115 exm127656 A G CEP350 4 8

1 179989296 exm127711 C G CEP350 9 14

1 179989560 exm127720 A T CEP350 2 3

1 180000542 exm127759 A C CEP350 32 66

1 180006126 exm127779 A G CEP350 0 1

1 180012213 exm127799 G A CEP350 5 11

1 180013308 exm127809 A G CEP350 8 11

1 180031395 exm127835 A G CEP350 0 2

1 180044257 exm127849 A G CEP350 0 4

1 180049723 exm127862 A T CEP350 3 8

1 180062369 exm127901 A G CEP350 10 28

1 180062586 exm127906 G A CEP350 9 15

1 180062624 exm127907 C A CEP350 16 24

1 180063075 exm127928 G A CEP350 0 4

1 180151343 exm128015 A G QSOX1 6 7

1 180885699 exm128370 A G KIAA1614 4 6

1 180885874 exm128387 A G KIAA1614 7 13

1 180886063 exm128398 A G KIAA1614 1 9

1 180905344 exm128473 G A KIAA1614 1 1

1 180913544 exm128515 A G KIAA1614 1 0

1 180914453 exm128523 A G KIAA1614 5 14

1 181754880 exm128872 A G CACNA1E 10 35

1 182026914 exm128999 A G ZNF648 0 1

1 182551266 exm129215 A G RNASEL 3 7

1 182555149 exm129256 A C RNASEL 1 2

1 182555169 exm129257 T A RNASEL 7 6

1 182555767 exm129286 A G RNASEL 1 2

1 182569501 exm129305 A C RGS16 3 21

1 182571193 exm129318 A G RGS16 1 6

1 182869224 exm129529 C A SHCBP1L 3 7

1 182898813 exm129554 C A SHCBP1L 8 21

1 183072751 exm129634 G A LAMC1 5 5

1 183084676 exm129660 G A LAMC1 11 17

1 183086017 exm129675 G A LAMC1 1 4

1 183086470 exm129679 A C LAMC1 6 8

1 183086509 exm129684 A C LAMC1 0 2

1 183094585 exm129735 A G LAMC1 2 2

1 183096522 exm129759 A G LAMC1 1 8

1 183099560 exm129778 A G LAMC1 1 1

1 183099605 exm129782 A G LAMC1 3 5

1 183184621 exm129867 A G LAMC2 1 6

1 10363929 exm12988 A G KIF1B 3 14

1 183192389 exm129903 A G LAMC2 5 11

1 183200177 exm129951 A C LAMC2 1 2

1 183207460 exm130008 A T LAMC2 2 2

1 183495812 exm130107 G A SMG7 6 8

1 10364578 exm13013 G A KIF1B 1 6

1 183520043 exm130206 C A SMG7 19 27

1 183525282 exm130222 G C NCF2 8 17

1 183534901 exm130253 A G NCF2 16 34

1 183617288 exm130344 G A APOBEC4 6 18

1 183617654 exm130363 G C APOBEC4 9 12

1 183914681 exm130511 A T COLGALT2 10 17

1 183938537 exm130537 A G COLGALT2 2 7

1 183944245 exm130554 C A COLGALT2 8 22

1 184690424 exm130697 A G EDEM3 2 5

1 184764908 exm130785 A C NIBAN1 7 9

1 184787810 exm130825 C A NIBAN1 18 36

1 184868383 exm130882 A G NIBAN1 2 3

1 185143701 exm131015 A C SWT1 4 21

1 185143825 exm131022 A C SWT1 15 29

1 185149547 exm131043 A G SWT1 4 6

1 185175889 exm131074 A G SWT1 1 2

1 185878632 exm131189 G A HMCN1 10 17

1 185951496 exm131277 A G HMCN1 3 7

1 185956606 exm131288 G A HMCN1 9 12

1 185962370 exm131308 A G HMCN1 0 1

1 185987370 exm131393 G A HMCN1 9 20

1 186010251 exm131424 T A HMCN1 15 31

1 186024710 exm131461 A C HMCN1 4 9

1 186026366 exm131462 A G HMCN1 1 1

1 186057762 exm131579 A G HMCN1 13 31

1 186064612 exm131623 G A HMCN1 10 20

1 186081996 exm131655 G A HMCN1 5 5

1 186092103 exm131714 A G HMCN1 14 48

1 186105800 exm131770 A G HMCN1 10 21

1 186135415 exm131857 A G HMCN1 1 4

1 186136015 exm131863 A G HMCN1 0 1

1 186269331 exm131945 A G PRG4 7 23

1 186277313 exm132104 A G PRG4 13 26

1 186281328 exm132160 G A PRG4 13 37

1 186319387 exm132317 G A TPR 5 9

1 186320479 exm132323 A G TPR 11 23

1 192778206 exm132845 G A RGS2 6 13

1 192778208 exm132846 G A RGS2 2 8

1 192998369 exm132892 C G UCHL5 1 3

1 10521644 exm13313 A G DFFA 0 1

1 196646650 exm133360 A G CFH 1 9

1 196695911 exm133432 A G CFH 4 13

1 196696005 exm133437 A C CFH 4 6

1 196706659 exm133458 A C CFH 2 8

1 196871610 exm133639 T A CFHR4 1 1

1 196871693 exm133646 A G CFHR4 3 4

1 10659333 exm13373 C A PEX14 1 0

1 196928188 exm133734 G A CFHR2 9 14

1 196963211 exm133769 T A CFHR5 2 8

1 196965196 exm133791 T A CFHR5 23 59

1 196977664 exm133828 T A CFHR5 7 12

1 197009688 exm133842 A G F13B 14 30

1 197009702 exm133843 A G F13B 12 22

1 197061086 exm133968 C A ASPM 16 34

1 197069857 exm134011 A G ASPM 1 0

1 197070126 exm134018 C A ASPM 12 38

1 197070313 exm134024 A G ASPM 2 3

1 197072256 exm134098 G A ASPM 2 9

1 197073648 exm134170 A G ASPM 8 14

1 197073885 exm134174 A C ASPM 2 12

1 197396853 exm134563 G A CRB1 2 11

1 197396961 exm134571 A C CRB1 6 17

1 197479756 exm134648 A G DENND1B 3 21

1 197480994 exm134664 A G DENND1B 6 11

1 197887095 exm134779 A G LHX9 15 39

1 197898187 exm134811 T A LHX9 1 3

1 198665894 exm134890 G A PTPRC 16 25

1 198668761 exm134903 C G PTPRC 16 37

1 198676063 exm134933 C A PTPRC 26 51

1 198682103 exm134953 G A PTPRC 2 2

1 198691595 exm134976 A T PTPRC 0 10

1 198717311 exm135018 G A PTPRC 2 6

1 198721822 exm135032 A G PTPRC 2 5

1 198723466 exm135040 C A PTPRC 0 2

1 198725303 exm135055 G C PTPRC 5 8

1 200522628 exm135309 A G KIF14 2 2

1 200534255 exm135332 T A KIF14 0 2

1 200586911 exm135462 G A KIF14 13 30

1 200587632 exm135486 G A KIF14 5 20

1 200618278 exm135531 G A DDX59 5 3

1 200635483 exm135584 C A DDX59 4 8

1 200816372 exm135640 A G CAMSAP2 10 19

1 200817285 exm135650 G A CAMSAP2 16 36

1 200818827 exm135699 A G CAMSAP2 1 7

1 200818980 exm135702 C G CAMSAP2 2 5

1 200869239 exm135799 A G INAVA 1 2

1 201016296 exm136224 A G CACNA1S 17 23

1 201028417 exm136293 C A CACNA1S 13 24

1 201031133 exm136321 A G CACNA1S 9 27

1 201046164 exm136404 A G CACNA1S 10 36

1 201054602 exm136457 A G CACNA1S 3 7

1 201079344 exm136514 C G CACNA1S 2 6

1 201176711 exm136651 A C IGFN1 34 66

1 10720369 exm13677 A G CASZ1 3 0

1 201181070 exm136804 A G IGFN1 15 28

1 201183364 exm136859 A G IGFN1 11 18

1 201184670 exm136875 A G IGFN1 7 8

1 10725193 exm13698 A G CASZ1 0 1

1 201196066 exm137020 A G IGFN1 0 1

1 201282405 exm137073 A G PKP1 4 10

1 201289495 exm137136 A G PKP1 11 23

1 201292223 exm137160 A G PKP1 3 8

1 201351421 exm137287 A T LAD1 4 14

1 201353941 exm137305 A G LAD1 3 5

1 201759701 exm137658 A C NAV1 16 13

1 201777136 exm137678 A G NAV1 0 2

1 201777250 exm137682 A G NAV1 2 2

1 201777553 exm137684 A G NAV1 14 34

1 201966638 exm138033 A C RNPEP 1 4

1 201972448 exm138066 G C RNPEP 12 24

1 202121683 exm138286 A G PTPN7 5 28

1 202128508 exm138333 A G PTPN7 2 8

1 202128601 exm138344 A G PTPN7 20 57

1 202245498 exm138420 A G LGR6 0 3

1 202273729 exm138450 A G LGR6 3 11

1 202276045 exm138457 A C LGR6 2 3

1 202287139 exm138487 A G LGR6 2 8

1 202411679 exm138680 G A PPP1R12B 5 20

1 202531968 exm138734 A G PPP1R12B 15 18

1 202702918 exm138854 A G KDM5B 2 2

1 202710733 exm138893 C G KDM5B 0 1

1 202714989 exm138912 G A KDM5B 2 2

1 11087207 exm13927 A G MASP2 7 14

1 203140586 exm139494 A G MYBPH 18 31

1 203150364 exm139574 A G CHI3L1 7 14

1 203154379 exm139607 A G CHI3L1 11 27

1 203188905 exm139675 A G CHIT1 9 24

1 203194848 exm139716 A G CHIT1 12 29

1 203317031 exm139806 A C FMOD 0 1

1 203317208 exm139820 A G FMOD 3 19

1 203317274 exm139825 C A FMOD 6 24

1 203452763 exm139856 A G PRELP 6 15

1 203452781 exm139858 G A PRELP 8 11

1 203465312 exm139908 G A OPTC 2 9

1 203467949 exm139929 A G OPTC 14 22

1 203472838 exm139964 A G OPTC 10 29

1 203743063 exm140172 A G LAX1 4 12

1 203743128 exm140179 A C LAX1 1 3

1 203743546 exm140192 A G LAX1 16 27

1 11106628 exm14025 C A MASP2 29 29

1 203816665 exm140281 G A ZC3H11A 8 15

1 203819654 exm140306 A G ZC3H11A 4 5

1 204218355 exm140722 G A PLEKHA6 3 4

1 204230576 exm140790 A G PLEKHA6 1 0

1 204379297 exm140865 G A PPP1R15B 9 30

1 204380375 exm140920 C G PPP1R15B 3 3

1 204380464 exm140925 A G PPP1R15B 6 18

1 204394023 exm140936 G C PIK3C2B 10 32

1 204394081 exm140942 A G PIK3C2B 21 37

1 204396787 exm140953 G A PIK3C2B 6 11

1 204411734 exm141036 A G PIK3C2B 3 16

1 204412558 exm141038 A C PIK3C2B 10 33

1 204416625 exm141067 G A PIK3C2B 0 3

1 204419062 exm141083 A G PIK3C2B 3 8

1 204434438 exm141131 A G PIK3C2B 9 9

1 204438340 exm141173 A T PIK3C2B 10 36

1 204588444 exm141347 A G LRRN2 0 1

1 204588859 exm141376 C G LRRN2 4 9

1 204957826 exm141566 A G NFASC 1 6

1 204978703 exm141619 A G NFASC 6 14

1 205035727 exm141753 G A CNTN2 1 0

1 205036357 exm141757 G A CNTN2 9 15

1 205038625 exm141760 A G CNTN2 3 8

1 205116729 exm141911 A G DSTYK 6 14

1 205131207 exm141951 A G DSTYK 0 8

1 205156739 exm141987 A G DSTYK 7 12

1 205589237 exm142534 A G ELK4 5 14

1 206320300 exm143219 G A CTSE 1 3

1 206327553 exm143239 A C CTSE 9 28

1 206328987 exm143252 G A CTSE 0 7

1 206648217 exm143407 A G IKBKE 0 1

1 206658621 exm143493 A C IKBKE 2 8

1 11194467 exm14355 A C MTOR 4 3

1 206811042 exm143674 G A DYRK3 10 29

1 206820793 exm143676 G A DYRK3 1 4

1 206821032 exm143679 C A DYRK3 2 8

1 206821286 exm143690 A G DYRK3 9 25

1 207039345 exm143859 G C IL20 11 30

1 207039838 exm143864 A G IL20 2 2

1 207109104 exm144028 A G PIGR 5 10

1 11249992 exm14406 A G ANGPTL7 0 1

1 207134410 exm144122 C A FCAMR 2 5

1 207135810 exm144145 A G FCAMR 8 14

1 207195437 exm144183 A G C1orf116 5 6

1 11253722 exm14420 A G ANGPTL7 4 6

1 207196028 exm144214 C G C1orf116 12 31

1 207228098 exm144315 G C PFKFB2 1 1

1 207252330 exm144381 A G PFKFB2 10 29

1 207495871 exm144514 C A CD55 10 22

1 207510691 exm144548 A G CD55 19 38

1 207643362 exm144632 A G CR2 6 10

1 207644414 exm144653 G A CR2 4 8

1 207646222 exm144665 A G CR2 9 15

1 207646941 exm144683 A G CR2 1 2

1 207649698 exm144723 A G CR2 10 22

1 207651374 exm144732 A G CR2 34 69

1 207679398 exm144765 A G CR1 5 12

1 11288898 exm14489 A G MTOR 0 3

1 207760805 exm144891 A G CR1 14 31

1 207760852 exm144895 A G CR1 8 11

1 207803936 exm144981 A G CR1 19 40

1 207925595 exm145089 A G CD46 0 4

1 207943690 exm145140 A G CD46 16 25

1 208062046 exm145181 C G CD34 26 40

1 208212224 exm145290 A G PLXNA2 5 9

1 208213363 exm145308 A G PLXNA2 10 14

1 208252777 exm145403 C G PLXNA2 9 15

1 208270177 exm145433 A G PLXNA2 3 7

1 208391177 exm145520 A G PLXNA2 7 12

1 209790859 exm145660 A G LAMB3 5 11

1 209799292 exm145766 A C LAMB3 1 5

1 209805938 exm145840 A C LAMB3 1 3

1 209811293 exm145869 G A LAMB3 12 35

1 210015757 exm146179 A C UTP25 11 26

1 210016920 exm146203 A T UTP25 5 4

1 11561204 exm14638 A G DISP3 12 33

1 210577884 exm146397 A G HHAT 11 19

1 210761405 exm146441 A G HHAT 0 1

1 211526639 exm146626 A G TRAF5 1 4

1 211533309 exm146643 A G TRAF5 7 14

1 211844613 exm146808 A G NEK2 16 33

1 211846971 exm146816 A G NEK2 6 9

1 212798487 exm147238 A G FAM71A 6 17

1 212798638 exm147249 A G FAM71A 16 22

1 212798808 exm147260 A G FAM71A 9 34

1 212799040 exm147275 G C FAM71A 13 26

1 212799171 exm147288 G A FAM71A 13 24

1 212799478 exm147302 A C FAM71A 1 1

1 212799541 exm147309 A G FAM71A 3 4

1 11585308 exm14731 A G DISP3 2 5

1 212799664 exm147321 A C FAM71A 12 22

1 213146166 exm147563 C A VASH2 3 8

1 213147400 exm147582 A G VASH2 29 54

1 11589979 exm14764 A G DISP3 2 9

1 213303007 exm147694 C G RPS6KC1 11 30

1 213414501 exm147751 C G RPS6KC1 11 20

1 213415122 exm147768 G A RPS6KC1 3 4

1 213415395 exm147776 A C RPS6KC1 2 6

1 214814079 exm148215 G A CENPF 5 14

1 214814794 exm148240 G A CENPF 1 0

1 214815888 exm148288 A G CENPF 5 9

1 214816201 exm148302 A G CENPF 1 1

1 214816297 exm148306 G A CENPF 12 27

1 214818803 exm148357 A G CENPF 5 10

1 214818821 exm148360 G A CENPF 6 23

1 214819463 exm148389 A G CENPF 18 34

1 214819757 exm148404 G A CENPF 8 13

1 214820079 exm148422 C G CENPF 2 6

1 214820239 exm148427 A G CENPF 7 22

1 215813908 exm148676 A G USH2A 1 1

1 215824001 exm148726 A G USH2A 3 14

1 215847892 exm148777 A T USH2A 2 2

1 215847910 exm148778 G A USH2A 1 0

1 215847956 exm148779 A C USH2A 5 8

1 215848696 exm148826 G A USH2A 15 18

1 215901392 exm148861 A G USH2A 0 4

1 215916600 exm148896 A G USH2A 6 13

1 215933042 exm148912 G C USH2A 11 24

1 215955515 exm148940 A G USH2A 2 0

1 216011361 exm149022 G A USH2A 3 4

1 216051181 exm149064 A G USH2A 7 13

1 216051206 exm149067 A G USH2A 14 24

1 216052349 exm149073 A G USH2A 1 1

1 216062306 exm149090 G A USH2A 1 1

1 216062396 exm149094 C A USH2A 2 4

1 216138719 exm149138 A G USH2A 2 5

1 216138790 exm149147 G A USH2A 7 8

1 216143995 exm149151 A G USH2A 1 2

1 216243512 exm149210 A G USH2A 1 4

1 216243560 exm149215 A G USH2A 12 22

1 216262444 exm149274 A G USH2A 3 4

1 216348678 exm149291 G A USH2A 10 26

1 216373385 exm149351 A G USH2A 13 34

1 216390860 exm149367 A G USH2A 0 2

1 216420226 exm149384 A G USH2A 12 17

1 216420277 exm149387 G A USH2A 1 7

1 216595201 exm149480 A G USH2A 15 30

1 217604654 exm149579 G A GPATCH2 12 23

1 217665070 exm149588 A G GPATCH2 0 1

1 11733801 exm14973 A C FBXO6 2 8

1 11733894 exm14979 A G FBXO6 12 16

1 220156616 exm149958 A G EPRS 7 4

1 220162030 exm149980 A C EPRS 11 19

1 220162132 exm149982 A T EPRS 4 5

1 220213564 exm150072 A G EPRS 0 1

1 220233090 exm150093 A G BPNT1 12 21

1 220324715 exm150263 G A RAB3GAP2 14 26

1 220331205 exm150302 C G RAB3GAP2 13 26

1 220928438 exm150537 A G 44622 2 2

1 220936333 exm150548 T A 44622 9 11

1 220970101 exm150600 A G 44621 4 11

1 220978444 exm150616 A G 44621 1 2

1 222717132 exm150870 A G HHIPL2 4 5

1 222717264 exm150875 A G HHIPL2 11 20

1 222801601 exm150985 A G MIA3 13 35

1 222802243 exm151006 C A MIA3 7 14

1 222802327 exm151011 A T MIA3 8 16

1 222802735 exm151027 G A MIA3 4 6

1 222803332 exm151053 A C MIA3 7 14

1 222803457 exm151057 C G MIA3 2 5

1 222832102 exm151119 C A MIA3 22 47

1 222892660 exm151207 C A BROX 0 13

1 223176061 exm151343 A G DISP1 11 31

1 223178670 exm151448 A G DISP1 7 7

1 223284120 exm151487 G A TLR5 6 19

1 223284713 exm151509 G A TLR5 7 12

1 223284731 exm151512 A G TLR5 7 12

1 223285435 exm151537 C A TLR5 5 5

1 223285910 exm151559 C A TLR5 1 3

1 223396624 exm151584 G C SUSD4 14 17

1 223934794 exm151876 G A CAPN2 4 9

1 223938665 exm151884 A G CAPN2 4 8

1 223959543 exm151969 G A CAPN2 4 7

1 224482084 exm152266 A G NVL 22 39

1 11850750 exm15227 A G MTHFR 1 0

1 224491438 exm152276 G A NVL 11 21

1 224492783 exm152287 A T NVL 3 6

1 225142710 exm152427 T A DNAH14 6 17

1 11852412 exm15255 A G MTHFR 10 10

1 225452983 exm152585 G A DNAH14 6 16

1 225521101 exm152640 G C DNAH14 5 20

1 225603006 exm152790 A G LBR 16 33

1 225706908 exm152906 A G ENAH 5 8

1 225707058 exm152921 A G ENAH 1 1

1 226016551 exm152977 A G EPHX1 9 9

1 226019646 exm152995 G A EPHX1 12 18

1 226026513 exm153011 A G EPHX1 1 2

1 11855398 exm15306 C A MTHFR 0 1

1 226037685 exm153086 A G TMEM63A 6 13

1 226040373 exm153091 A G TMEM63A 8 13

1 226040388 exm153092 A G TMEM63A 2 3

1 226040404 exm153094 A G TMEM63A 17 23

1 226041377 exm153100 A G TMEM63A 6 10

1 226065232 exm153194 G A TMEM63A 4 13

1 226065268 exm153199 C G TMEM63A 16 26

1 226550829 exm153581 G A PARP1 5 11

1 226553677 exm153599 A G PARP1 6 11

1 226568798 exm153657 G A PARP1 1 7

1 226573216 exm153671 A G PARP1 16 31

1 226924363 exm153876 A G ITPKB 0 1

1 227076717 exm153972 A G PSEN2 2 7

1 227083263 exm154012 A G PSEN2 4 6

1 227152821 exm154039 A C COQ8A 3 9

1 227170644 exm154098 G A COQ8A 2 8

1 227171916 exm154140 G A COQ8A 1 3

1 227174370 exm154185 A G COQ8A 4 9

1 227843003 exm154472 A G ZNF678 0 1

1 227843423 exm154496 A G ZNF678 6 11

1 227921714 exm154550 A G JMJD4 3 11

1 227921717 exm154551 A G JMJD4 6 10

1 227947141 exm154638 G A SNAP47 3 3

1 227954716 exm154646 A G SNAP47 8 6

1 228432264 exm155405 A T OBSCN 1 5

1 11907328 exm15541 A G NPPA 8 31

1 11907659 exm15554 G A NPPA 6 22

1 228461936 exm155556 A G OBSCN 6 6

1 228462332 exm155581 A G OBSCN 7 10

1 228464316 exm155626 C G OBSCN 7 11

1 228468069 exm155722 A G OBSCN 8 11

1 228469801 exm155754 C G OBSCN 1 4

1 228481314 exm155930 G A OBSCN 11 18

1 228495226 exm156045 A C OBSCN 2 8

1 228526067 exm156303 A C OBSCN 11 17

1 228548255 exm156461 C G OBSCN 11 22

1 228558892 exm156606 A G OBSCN 25 41

1 12010487 exm15712 C A PLOD1 1 0

1 229633970 exm157351 G C NUP133 0 1

1 229643815 exm157373 A G NUP133 5 10

1 229772137 exm157617 A G URB2 1 13

1 229772285 exm157625 A G URB2 16 34

1 229772519 exm157636 A G URB2 1 2

1 229772630 exm157640 G A URB2 1 1

1 229773691 exm157676 A G URB2 5 6

1 229783267 exm157701 A C URB2 3 8

1 229787030 exm157722 A G URB2 3 4

1 12025600 exm15796 A G PLOD1 2 2

1 230800250 exm157966 G A COG2 8 18

1 12025648 exm15798 A G PLOD1 4 4

1 230807296 exm157992 G A COG2 1 2

1 230822828 exm158018 A G COG2 4 7

1 230838903 exm158058 A G AGT 4 6

1 230839020 exm158065 A G AGT 7 10

1 12030760 exm15810 A G PLOD1 0 1

1 230845866 exm158104 C A AGT 7 11

1 230846305 exm158130 A G AGT 21 36

1 230846385 exm158136 A G AGT 7 18

1 1116188 exm1582 A G TTLL10 1 2

1 230915993 exm158243 G A CAPN9 4 5

1 230916057 exm158247 C A CAPN9 11 18

1 230930967 exm158293 A C CAPN9 10 36

1 231377189 exm158660 G A GNPAT 4 9

1 231401099 exm158678 A G GNPAT 10 24

1 12064087 exm15888 A G MFN2 4 3

1 231829740 exm158964 A G DISC1 1 0

1 232144751 exm159088 A G DISC1 7 23

1 232649979 exm159305 A G SIPA1L2 1 5

1 12069725 exm15931 A G MFN2 1 2

1 232940908 exm159363 G A MAP10 13 36

1 232941039 exm159371 A C MAP10 1 8

1 232941173 exm159386 C G MAP10 0 3

1 232941844 exm159412 A C MAP10 6 16

1 232942469 exm159437 A G MAP10 5 11

1 233091465 exm159509 A G NTPCR 17 30

1 233092169 exm159512 A C NTPCR 8 21

1 233105691 exm159517 A G NTPCR 0 5

1 233121884 exm159550 G A PCNX2 0 1

1 233134132 exm159584 A G PCNX2 0 13

1 233313594 exm159697 A C PCNX2 5 8

1 233314839 exm159699 A G PCNX2 4 10

1 233335945 exm159710 G A PCNX2 7 16

1 233394781 exm159796 A C PCNX2 4 10

1 233397855 exm159811 A G PCNX2 1 4

1 233398867 exm159823 A C PCNX2 12 23

1 233518291 exm159958 A G MAP3K21 0 2

1 233750008 exm159971 C A KCNK1 4 10

1 12089946 exm15999 G C MIIP 8 10

1 233802497 exm159998 A G KCNK1 8 14

1 233807077 exm160014 G A KCNK1 4 7

1 234564902 exm160211 A C TARBP1 5 7

1 234564951 exm160217 A T TARBP1 7 9

1 234565176 exm160224 A G TARBP1 2 5

1 234573025 exm160257 G A TARBP1 9 15

1 234582720 exm160265 A G TARBP1 3 5

1 12091851 exm16033 A G MIIP 16 33

1 235345674 exm160590 G A ARID4B 4 6

1 235345832 exm160601 A G ARID4B 5 13

1 235577753 exm160708 A G TBCE 6 19

1 235599875 exm160738 C G TBCE 1 4

1 235606224 exm160762 A G TBCE 2 16

1 235860421 exm160890 A G LYST 0 3

1 235922698 exm161045 A G LYST 9 12

1 235955205 exm161135 A G LYST 12 35

1 235969711 exm161205 A G LYST 0 2

1 235972432 exm161245 C G LYST 0 1

1 235972553 exm161254 A G LYST 0 3

1 236212117 exm161521 A G NID1 1 4

1 236228164 exm161534 G C NID1 9 23

1 236557766 exm161688 G C EDARADD 2 3

1 236645609 exm161709 A G EDARADD 25 21

1 236711344 exm161808 A C LGALS8 1 3

1 236722318 exm161904 G A HEATR1 2 5

1 236734764 exm161970 A G HEATR1 1 1

1 236740135 exm162011 G A HEATR1 3 8

1 236750709 exm162073 A G HEATR1 33 55

1 236979764 exm162348 A G MTR 9 26

1 236990141 exm162358 A G MTR 27 54

1 236995331 exm162372 A G MTR 6 12

1 237001869 exm162387 A G MTR 12 23

1 237057789 exm162467 A G MTR 12 21

1 12304403 exm16253 A C VPS13D 11 20

1 1119361 exm1626 G A TTLL10 0 2

1 237868623 exm162821 G A RYR2 13 16

1 237880555 exm162855 G A RYR2 4 6

1 12318027 exm16297 G A VPS13D 11 28

1 238050155 exm163023 A G ZP4 4 26

1 238050801 exm163034 A G ZP4 1 0

1 238053179 exm163043 G A ZP4 0 1

1 240070944 exm163083 A G CHRM3 2 3

1 240071937 exm163105 A C CHRM3 0 8

1 240256767 exm163202 A G FMN2 10 33

1 241753378 exm163688 A G KMO 21 27

1 241755453 exm163712 G A KMO 3 7

1 241761077 exm163730 G C OPN3 5 10

1 241767708 exm163747 A G OPN3 2 5

1 241798007 exm163786 G A CHML 16 30

1 241798302 exm163808 C A CHML 2 4

1 241798451 exm163815 C A CHML 21 36

1 241798684 exm163821 G A CHML 1 1

1 241875105 exm163880 A C WDR64 4 11

1 241912941 exm163922 A G WDR64 16 33

1 242023898 exm164041 G A EXO1 2 0

1 242042437 exm164105 A G EXO1 2 7

1 243456418 exm164373 A G SDCCAG8 12 25

1 243480091 exm164396 A G SDCCAG8 2 2

1 243480113 exm164397 A G SDCCAG8 19 31

1 243507540 exm164412 C G SDCCAG8 4 7

1 244538684 exm164550 A G C1orf100 1 4

1 244538691 exm164552 G A C1orf100 0 1

1 244538702 exm164554 A G C1orf100 0 1

1 244641216 exm164635 A C CATSPERE 14 29

1 244745107 exm164711 G A CATSPERE 0 1

1 244756720 exm164724 G C CATSPERE 23 37

1 245772651 exm165012 C G KIF26B 23 52

1 245772663 exm165014 A G KIF26B 0 2

1 245772826 exm165026 A G KIF26B 17 27

1 245809554 exm165050 A G KIF26B 6 10

1 245849622 exm165121 A G KIF26B 1 1

1 245849742 exm165133 A G KIF26B 12 24

1 245850303 exm165163 C A KIF26B 1 3

1 245850369 exm165166 G A KIF26B 11 20

1 245861419 exm165228 A G KIF26B 1 4

1 245865900 exm165261 A G KIF26B 3 7

1 12364696 exm16532 A G VPS13D 15 36

1 246755204 exm165419 A G CNST 19 23

1 246810488 exm165458 C A CNST 15 26

1 246810869 exm165480 C G CNST 19 38

1 247006035 exm165601 A G AHCTF1 16 36

1 247012943 exm165612 A G AHCTF1 14 14

1 247030547 exm165716 G A AHCTF1 2 10

1 247040261 exm165730 C A AHCTF1 1 5

1 247051481 exm165752 G A AHCTF1 16 22

1 247264347 exm165977 G A ZNF669 33 63

1 247267269 exm166005 A G ZNF669 26 41

1 247267417 exm166019 A G ZNF669 11 28

1 247267486 exm166024 A C ZNF669 8 12

1 247471846 exm166179 A G ZNF496 5 16

1 247582305 exm166248 G A NLRP3 4 9

1 247588390 exm166315 A T NLRP3 1 6

1 247597571 exm166351 A C NLRP3 6 10

1 247614685 exm166406 A C OR2B11 0 2

1 247695143 exm166509 A G OR2C3 6 11

1 247695698 exm166532 A G OR2C3 19 32

1 247695726 exm166534 C A OR2C3 3 8

1 247695729 exm166535 C A OR2C3 17 46

1 247769378 exm166638 G A OR2G3 15 40

1 247769419 exm166641 G A OR2G3 1 5

1 247875276 exm166702 A G OR6F1 2 5

1 247875604 exm166729 A G OR6F1 4 9

1 248004787 exm166913 A G OR11L1 3 16

1 248039555 exm166995 A G TRIM58 12 29

1 248039789 exm167009 G A TRIM58 0 1

1 248084321 exm167073 G A OR2T8 16 22

1 248085233 exm167149 A G OR2T8 10 18

1 12438584 exm16730 A G VPS13D 3 10

1 248309260 exm167542 G C OR2M5 4 4

1 12446332 exm16755 G A VPS13D 4 16

1 248366370 exm167630 G A OR2M3 2 3

1 248366400 exm167637 A G OR2M3 2 5

1 248402897 exm167723 A G OR2M4 10 13

1 248403015 exm167732 A C OR2M4 2 2

1 248403054 exm167737 A G OR2M4 2 1

1 12475249 exm16782 G A VPS13D 10 11

1 248524890 exm168031 G A OR2T4 8 12

1 248525484 exm168104 A G OR2T4 1 0

1 248525856 exm168126 A G OR2T4 11 26

1 248525864 exm168127 G C OR2T4 12 21

1 248525923 exm168130 A G OR2T4 5 19

1 248551346 exm168170 A G OR2T6 0 3

1 248551598 exm168179 A G OR2T6 17 32

1 248685089 exm168448 A G OR2G6 8 25

1 248685260 exm168463 C A OR2G6 6 22

1 248685425 exm168486 C A OR2G6 9 27

1 248813498 exm168804 A G OR2T27 16 34

1 248844851 exm168885 A G OR14I1 1 1

1 248845025 exm168897 G A OR14I1 19 39

1 248845062 exm168898 A G OR14I1 12 31

1 101343310 exm1690021 A G EXTL2 0 1

1 12726049 exm16904 A C AADACL4 4 5

1 109806964 exm1691311 C G CELSR2 11 13

1 249210835 exm169139 A C PGBD2 0 2

1 249211618 exm169173 A G PGBD2 2 7

1 249211903 exm169186 A G PGBD2 4 5

1 111490672 exm1695394 G A LRIF1 1 2

1 12779654 exm16970 A G AADACL3 5 21

1 12780909 exm16975 A G AADACL3 2 4

1 111957729 exm1697697 G A OVGP1 0 1

1 12785380 exm16994 A G AADACL3 12 42

1 12785451 exm17000 G A AADACL3 12 42

1 113655293 exm1701686 G A LRIG2 0 1

1 12815668 exm17034 G A C1orf158 13 31

1 12835144 exm17071 A G PRAMEF12 12 31

1 12835158 exm17074 A G PRAMEF12 4 18

1 12835240 exm17077 A T PRAMEF12 14 25

1 12835805 exm17090 C G PRAMEF12 2 3

1 1391192 exm1709164 A G ATAD3C 11 25

1 12835930 exm17098 A G PRAMEF12 4 7

1 145704116 exm1710711 A C CD160 14 21

1 151742726 exm1714417 A C OAZ3 1 1

1 152191637 exm1714751 C G HRNR 0 1

1 153314113 exm1715483 G C PGLYRP4 15 36

1 12837669 exm17162 A C PRAMEF12 10 30

1 12837728 exm17168 A G PRAMEF12 10 30

1 155255014 exm1717319 A G HCN3 2 5

1 155658015 exm1717783 A G YY1AP1 5 26

1 157548565 exm1720203 G A FCRL4 1 3

1 158435704 exm1720883 G A OR10K1 6 21

1 158650431 exm1721153 G A SPTA1 2 2

1 167095588 exm1731713 A G DUSP27 2 1

1 169346051 exm1733616 A C BLZF1 13 30

1 170521212 exm1734327 A G GORAB 0 1

1 175063127 exm1737146 C A TNN 1 1

1 182026840 exm1740183 C A ZNF648 0 1

1 183514391 exm1740836 C A SMG7 6 5

1 203186990 exm1747099 G A CHIT1 1 4

1 20440684 exm1747582 A G PLA2G2D 2 11

1 205589788 exm1748104 A G ELK4 11 17

1 207105843 exm1748637 G C PIGR 16 30

1 207803958 exm1748962 A T CR1 27 73

1 208383663 exm1749197 G C PLXNA2 7 12

1 209804028 exm1749381 A C LAMB3 17 31

1 209964095 exm1749474 A G IRF6 2 4

1 12921254 exm17605 A G PRAMEF2 8 10

1 214815609 exm1760977 G C CENPF 7 22

1 12921311 exm17613 A G PRAMEF2 10 30

1 235577756 exm1768956 A C TBCE 1 3

1 235602102 exm1768989 A G TBCE 3 3

1 236702219 exm1769386 A G LGALS8 0 1

1 238053235 exm1770077 G C ZP4 0 1

1 243493867 exm1771270 A G SDCCAG8 6 17

1 247614465 exm1772907 A G OR2B11 10 29

1 32089307 exm1792172 A G HCRTR1 0 3

1 36552901 exm1799351 A C TEKT2 11 20

1 36553752 exm1799360 G A TEKT2 1 4

1 38184120 exm1800866 A C EPHA10 3 5

1 55077377 exm1833481 A G FAM151A 5 10

1 6637055 exm1863306 C G TAS1R1 13 41

1 14105049 exm18656 A C PRDM2 4 8

1 14105291 exm18671 A G PRDM2 8 14

1 14105639 exm18687 A G PRDM2 5 9

1 14106623 exm18736 T A PRDM2 0 4

1 14107135 exm18751 G C PRDM2 4 8

1 14107360 exm18766 A T PRDM2 19 31

1 14108821 exm18825 G A PRDM2 1 4

1 14109031 exm18838 G A PRDM2 0 5

1 1164018 exm1886 C G SDF4 4 7

1 1164065 exm1890 A G SDF4 10 31

1 15546012 exm19068 A G TMEM51 7 7

1 91845778 exm1927669 T A HFM1 3 9

1 15789260 exm19338 A G CELA2A 8 13

1 15793924 exm19374 A G CELA2A 1 4

1 15833450 exm19511 A G CASP9 2 10

1 15871023 exm19603 A G DNAJC16 7 9

1 15890424 exm19633 A G DNAJC16 0 1

1 15986454 exm19789 A G RSC1A1 8 16

1 15987274 exm19820 A G RSC1A1 4 9

1 15987724 exm19841 A G RSC1A1 2 3

1 16053627 exm19917 A G PLEKHM2 16 28

1 16060330 exm20018 A C PLEKHM2 5 19

1 16235842 exm20324 G A SPEN 12 20

1 16247386 exm20341 A G SPEN 24 35

1 16256621 exm20408 G C SPEN 2 6

1 16259205 exm20518 A C SPEN 4 15

1 16261683 exm20628 A G SPEN 1 1

1 16261685 exm20630 A G SPEN 10 16

1 16456763 exm21359 A G EPHA2 17 24

1 16456811 exm21360 A G EPHA2 0 2

1 16534047 exm21591 A G ARHGEF19 8 14

1 16727361 exm21806 A C SPATA21 7 12

1 16731572 exm21828 G A SPATA21 10 17

1 16736451 exm21864 A G SPATA21 4 11

1 16736538 exm21872 A G SPATA21 3 9

1 114680359 exm2230842 A G SYT6 2 5

1 36645615 exm2233283 A G MAP7D1 0 1

1 55248120 exm2233617 G A TTC22 6 28

1 86904587 exm2273658 A C CLCA2 1 0

1 22163446 exm2277027 A G HSPG2 0 1

1 222716993 exm2277028 A C HHIPL2 7 11

1 17313049 exm22865 A G ATP13A2 0 1

1 17316686 exm22926 A G ATP13A2 12 18

1 17371286 exm23101 G A SDHB 15 34

1 17380483 exm23111 A G SDHB 14 29

1 17395655 exm23125 A G PADI2 3 18

1 17413075 exm23205 A G PADI2 8 17

1 17531755 exm23270 G A PADI1 1 1

1 17531782 exm23273 A G PADI1 10 15

1 17548945 exm23288 G A PADI1 0 2

1 17552548 exm23307 G A PADI1 1 1

1 17555490 exm23331 A C PADI1 10 19

1 17594433 exm23455 A G PADI3 0 3

1 17596818 exm23464 A G PADI3 18 37

1 17603075 exm23518 A G PADI3 2 2

1 17662705 exm23601 C G PADI4 13 39

1 17668827 exm23644 A G PADI4 1 1

1 17690206 exm23705 A C PADI4 1 1

1 17698855 exm23715 A G PADI6 6 10

1 17914057 exm23891 A G ARHGEF10L 18 33

1 17914122 exm23900 A G ARHGEF10L 3 6

1 17961432 exm24001 G C ARHGEF10L 4 11

1 18021668 exm24081 A C ARHGEF10L 4 7

1 880502 exm241 A G NOC2L 9 16

1 18152871 exm24195 A G ACTL8 3 4

1 18809019 exm24404 G A KLHDC7A 17 37

1 18809613 exm24436 A G KLHDC7A 0 3

1 19166482 exm24567 A G TAS1R2 1 1

1 19166592 exm24573 A G TAS1R2 2 4

1 19166647 exm24582 A G TAS1R2 5 18

1 19166668 exm24585 A G TAS1R2 8 14

1 19166829 exm24594 G A TAS1R2 0 2

1 19201071 exm24761 A G ALDH4A1 3 6

1 19201951 exm24769 C A ALDH4A1 13 35

1 19215927 exm24881 A G ALDH4A1 0 1

1 19437319 exm25082 A G UBR4 2 1

1 19504046 exm25489 A G UBR4 0 1

1 19564614 exm25684 G A EMC1 2 5

1 20073044 exm26385 A G TMCO4 2 6

1 20107136 exm26426 G C TMCO4 3 12

1 20141552 exm26485 A G RNF186 2 7

1 20246852 exm26538 G A PLA2G2E 4 10

1 20248865 exm26548 A G PLA2G2E 11 25

1 20442878 exm26672 A G PLA2G2D 0 1

1 20466711 exm26688 A G PLA2G2F 0 4

1 20645086 exm26817 T A VWA5B1 2 1

1 20672051 exm26893 A G VWA5B1 0 1

1 881918 exm269 A G NOC2L 3 5

1 20827784 exm26973 G A MUL1 9 11

1 20829761 exm26990 C A MUL1 4 10

1 20960385 exm27075 T A PINK1 5 6

1 20964573 exm27091 A G PINK1 1 0

1 20966404 exm27097 A G PINK1 0 1

1 20972168 exm27128 A G PINK1 1 2

1 20998491 exm27291 A G KIF17 1 0

1 20998548 exm27296 A G KIF17 4 9

1 1266738 exm2745 A G TAS1R3 1 1

1 21036276 exm27463 G A KIF17 7 22

1 21042033 exm27474 G A KIF17 1 0

1 21050969 exm27548 A G SH2D5 17 44

1 21180098 exm27691 A G EIF4G3 14 20

1 1267253 exm2774 A G TAS1R3 2 5

1 21231460 exm27744 C A EIF4G3 17 29

1 21267993 exm27747 C G EIF4G3 1 2

1 21268164 exm27751 A G EIF4G3 2 1

1 21268346 exm27764 G A EIF4G3 21 31

1 21268644 exm27774 G A EIF4G3 17 29

1 21306835 exm27790 G A EIF4G3 1 4

1 21560060 exm27850 G A ECE1 2 2

1 21573855 exm27882 A G ECE1 1 3

1 21586862 exm27911 A G ECE1 7 13

1 22150671 exm28558 A G HSPG2 7 15

1 22154365 exm28574 A G HSPG2 1 6

1 22160020 exm28704 A G HSPG2 12 24

1 22174498 exm28924 A G HSPG2 4 9

1 22186712 exm29134 A G HSPG2 1 3

1 22206617 exm29308 A G HSPG2 10 23

1 22213821 exm29412 A G HSPG2 0 1

1 22214436 exm29435 G A HSPG2 7 12

1 22852880 exm29914 C G ZBTB40 6 8

1 22903176 exm29948 A G EPHA8 15 17

1 22927533 exm30099 A G EPHA8 1 3

1 22927541 exm30100 A G EPHA8 8 19

1 23417738 exm30510 A G LUZP1 0 1

1 23417742 exm30512 A G LUZP1 4 7

1 23418576 exm30551 G A LUZP1 14 31

1 23418803 exm30559 G C LUZP1 13 22

1 23419058 exm30571 A C LUZP1 4 6

1 23419914 exm30607 A G LUZP1 8 15

1 23763767 exm30959 A G ASAP3 6 10

1 23842860 exm31044 A G E2F2 16 35

1 23847529 exm31060 A G E2F2 0 1

1 24077451 exm31243 A G ELOA 26 58

1 24077943 exm31272 A G ELOA 3 2

1 24077994 exm31278 G A ELOA 9 23

1 24080617 exm31299 C A ELOA 2 0

1 24082334 exm31308 G A ELOA 5 11

1 24082853 exm31320 A G ELOA 9 11

1 24083471 exm31322 G A ELOA 0 2

1 24083609 exm31334 G C ELOA 1 4

1 24181041 exm31573 A G FUCA1 18 34

1 24383994 exm31698 G A MYOM3 5 9

1 24388465 exm31716 A G MYOM3 0 4

1 24389694 exm31731 A G MYOM3 2 5

1 24401926 exm31797 G A MYOM3 4 11

1 24411054 exm31856 G A MYOM3 17 42

1 24411058 exm31857 A C MYOM3 0 2

1 24417388 exm31900 A G MYOM3 1 2

1 24449814 exm32074 A G IL22RA1 2 6

1 24454636 exm32081 G A IL22RA1 0 2

1 24463705 exm32102 A G IL22RA1 1 3

1 1309405 exm3211 G A AURKAIP1 0 1

1 24483832 exm32136 G A IFNLR1 3 5

1 24484220 exm32152 A C IFNLR1 15 27

1 24663184 exm32232 G A GRHL3 6 11

1 24664220 exm32253 A G GRHL3 1 4

1 24668716 exm32273 A G GRHL3 1 4

1 24669457 exm32288 A G GRHL3 3 9

1 24671418 exm32299 G A GRHL3 6 11

1 24746056 exm32388 A G NIPAL3 0 1

1 24782763 exm32447 A G NIPAL3 1 8

1 1322753 exm3254 A G CCNL2 1 6

1 1322765 exm3257 A G CCNL2 1 1

1 888563 exm332 A G NOC2L 38 56

1 1334519 exm3320 G C CCNL2 0 2

1 26107481 exm33252 A G MAN1C1 0 2

1 26110236 exm33269 A G MAN1C1 2 5

1 26131638 exm33277 G A SELENON 3 7

1 1337481 exm3329 C A MRPL20 4 5

1 1337539 exm3332 T A MRPL20 29 55

1 26142081 exm33340 A G SELENON 11 24

1 26161671 exm33403 A G AUNIP 0 3

1 26162259 exm33428 A G AUNIP 3 5

1 26162313 exm33430 C A AUNIP 1 3

1 26356190 exm33667 C G EXTL1 1 2

1 26361467 exm33708 A G EXTL1 0 4

1 26365752 exm33726 A G SLC30A2 1 0

1 26372371 exm33774 G A SLC30A2 3 9

1 26508416 exm33962 A G CNKSR1 15 29

1 1372441 exm3397 A C VWA1 1 0

1 26509049 exm33979 A G CNKSR1 0 2

1 26509070 exm33983 G C CNKSR1 13 31

1 26510563 exm34008 A G CNKSR1 1 2

1 26514732 exm34052 A G CNKSR1 0 1

1 26515840 exm34088 A G CNKSR1 12 30

1 26524450 exm34149 A G CATSPER4 1 6

1 26524534 exm34158 A G CATSPER4 9 9

1 1372743 exm3425 A G VWA1 2 13

1 26582340 exm34256 G A CEP85 4 7

1 1372750 exm3426 A G VWA1 1 3

1 26597521 exm34292 C A CEP85 3 3

1 26620806 exm34433 G A UBXN11 3 6

1 26629304 exm34469 A G UBXN11 3 7

1 26688173 exm34735 A G ZNF683 2 4

1 27219192 exm35569 A G GPATCH3 0 2

1 27220873 exm35583 A C GPATCH3 3 11

1 27223977 exm35593 A G GPATCH3 1 3

1 27224070 exm35601 A G GPATCH3 0 3

1 27226817 exm35621 C G GPATCH3 0 1

1 27277916 exm35738 A G KDF1 0 1

1 27278439 exm35762 A G KDF1 0 1

1 27589698 exm35980 A G WDTC1 10 19

1 27622832 exm36016 T A WDTC1 7 16

1 27684948 exm36292 A G MAP3K6 17 27

1 27687248 exm36334 G C MAP3K6 11 19

1 27688031 exm36363 C G MAP3K6 4 29

1 27697425 exm36475 G C FCN3 3 17

1 27697444 exm36476 A T FCN3 3 7

1 27699647 exm36486 G A FCN3 14 19

1 28261712 exm37224 G C SMPDL3B 7 11

1 28279854 exm37252 A G SMPDL3B 15 21

1 28559436 exm37483 C G DNAJC8 2 6

1 28559479 exm37485 A G DNAJC8 9 20

1 29314212 exm38083 G A EPB41 3 11

1 29320013 exm38093 A G EPB41 18 29

1 29587158 exm38399 A G PTPRU 6 9

1 29644320 exm38571 A G PTPRU 1 2

1 31212666 exm38728 A G LAPTM5 6 10

1 31439065 exm38929 G A PUM1 8 22

1 31902357 exm39205 G A SERINC2 0 3

1 31905860 exm39214 A G SERINC2 10 22

1 32042762 exm39247 A G TINAGL1 4 5

1 32051421 exm39312 A C TINAGL1 0 2

1 32086654 exm39356 A G HCRTR1 2 8

1 32096386 exm39406 A G PEF1 1 5

1 32119265 exm39452 A G COL16A1 9 11

1 892380 exm395 A G NOC2L 34 53

1 32130793 exm39505 A G COL16A1 1 0

1 32138045 exm39539 A G COL16A1 0 1

1 32146520 exm39565 A G COL16A1 3 7

1 32156167 exm39636 A G COL16A1 10 29

1 32158384 exm39667 A G COL16A1 14 34

1 32159764 exm39677 C A COL16A1 1 2

1 32163574 exm39706 A G COL16A1 13 22

1 32256329 exm39989 A G SPOCD1 15 36

1 32259363 exm40026 A G SPOCD1 1 3

1 32259791 exm40038 A G SPOCD1 1 2

1 32262292 exm40046 A G SPOCD1 0 2

1 32265676 exm40066 A C SPOCD1 2 5

1 32279871 exm40100 G A SPOCD1 23 39

1 32280660 exm40131 A G SPOCD1 1 0

1 32671801 exm40422 A C IQCC 19 40

1 32672143 exm40427 A G IQCC 14 44

1 32672932 exm40453 A G IQCC 6 11

1 33235660 exm41158 A G KIAA1522 6 20

1 33236624 exm41212 A G KIAA1522 1 5

1 33236845 exm41221 A G KIAA1522 12 36

1 33237266 exm41231 A G KIAA1522 11 19

1 33562312 exm41667 A G AZIN2 14 26

1 33563718 exm41683 A G AZIN2 6 19

1 33820016 exm41954 A G PHC2 8 12

1 33836164 exm42016 A G PHC2 5 4

1 33960087 exm42133 C A ZSCAN20 3 11

1 34015851 exm42264 A G CSMD2 4 8

1 34015872 exm42266 A G CSMD2 1 1

1 34035009 exm42282 G A CSMD2 6 13

1 34035114 exm42285 G A CSMD2 2 13

1 34066506 exm42338 A G CSMD2 5 15

1 34068114 exm42355 A G CSMD2 10 23

1 34080121 exm42397 A G CSMD2 1 2

1 34190239 exm42554 G A CSMD2 13 19

1 34204887 exm42576 G A CSMD2 3 2

1 34291367 exm42648 A C CSMD2 3 3

1 34662914 exm42750 A G C1orf94 11 22

1 34666589 exm42796 A G C1orf94 1 2

1 34667724 exm42804 G A C1orf94 7 17

1 34678007 exm42826 A C C1orf94 2 5

1 35223118 exm42867 A G GJB5 12 13

1 35223502 exm42891 A G GJB5 3 5

1 35223509 exm42892 A G GJB5 9 24

1 35227276 exm42967 A G GJB4 0 1

1 35227362 exm42977 G C GJB4 7 13

1 35476609 exm43405 C A ZMYM6 20 41

1 35480426 exm43419 G A ZMYM6 2 1

1 35486022 exm43440 C A ZMYM6 26 36

1 35579565 exm43514 A C ZMYM1 2 6

1 35579797 exm43523 A G ZMYM1 3 6

1 36181316 exm44162 G C C1orf216 9 17

1 36181502 exm44174 A G C1orf216 3 12

1 36181522 exm44177 A C C1orf216 11 16

1 36211151 exm44239 A G CLSPN 0 2

1 36214065 exm44255 A G CLSPN 16 23

1 36226161 exm44301 G A CLSPN 12 13

1 36226432 exm44310 A C CLSPN 2 12

1 36230911 exm44351 G A CLSPN 11 23

1 36235452 exm44354 A G CLSPN 4 12

1 36551587 exm44613 A G TEKT2 22 45

1 36636854 exm44880 A G MAP7D1 7 14

1 1686081 exm4503 A G NADK 11 27

1 36754784 exm45038 A G THRAP3 12 24

1 36755361 exm45054 A G THRAP3 2 9

1 1688694 exm4527 A G NADK 0 1

1 36932062 exm45467 A G CSF3R 7 16

1 36933198 exm45489 A G CSF3R 8 22

1 36937878 exm45547 A G CSF3R 8 17

1 37325609 exm45691 A G GRIK3 14 26

1 37941196 exm45759 A C ZC3H12A 2 2

1 37948886 exm45852 A G ZC3H12A 24 44

1 38006105 exm45917 C A SNIP1 13 38

1 38006337 exm45931 A G SNIP1 31 43

1 38027737 exm46001 C A DNALI1 10 11

1 38027796 exm46007 C A DNALI1 5 14

1 38033291 exm46033 A G GNL2 11 27

1 38033930 exm46037 G A GNL2 1 7

1 38078561 exm46136 A G RSPO1 4 21

1 38186226 exm46320 A G EPHA10 4 12

1 38188736 exm46339 A G EPHA10 8 13

1 38197170 exm46368 A G EPHA10 2 13

1 38197247 exm46376 A G EPHA10 5 14

1 1847886 exm4638 A G CALML6 0 1

1 1847940 exm4640 A G CALML6 3 3

1 1847968 exm4644 A C CALML6 8 11

1 38483428 exm46876 A G UTP11 4 6

1 39340839 exm47008 G A GJA9 4 2

1 39816626 exm47535 A G MACF1 4 12

1 39823135 exm47552 G A MACF1 1 1

1 39824930 exm47586 G A MACF1 1 3

1 39838268 exm47622 C G MACF1 1 6

1 39844154 exm47623 A G MACF1 1 3

1 39844949 exm47628 A G MACF1 3 5

1 39852883 exm47662 A G MACF1 24 28

1 39854156 exm47703 C G MACF1 1 2

1 39877514 exm47769 G A KIAA0754 8 17

1 39878015 exm47790 G A KIAA0754 7 25

1 39878443 exm47808 A G KIAA0754 0 1

1 39909220 exm48003 A C MACF1 4 4

1 39920726 exm48058 A G MACF1 2 5

1 40228799 exm48510 G A BMP8B 5 8

1 40702377 exm48959 A G RLF 1 0

1 40702974 exm48971 A C RLF 1 0

1 40702991 exm48972 A G RLF 16 33

1 40704525 exm49009 A C RLF 2 5

1 40705578 exm49047 C A RLF 13 29

1 40768830 exm49188 A G COL9A2 10 22

1 1896433 exm4922 A G CFAP74 13 38

1 40773119 exm49231 A C COL9A2 4 16

1 40775800 exm49250 A G COL9A2 4 5

1 40775937 exm49256 A G COL9A2 1 2

1 40777347 exm49278 A G COL9A2 15 22

1 40928708 exm49416 A G ZFP69B 6 6

1 40928950 exm49426 T A ZFP69B 1 3

1 40928969 exm49427 G A ZFP69B 0 1

1 40945067 exm49438 A G ZFP69 7 18

1 40954838 exm49443 C G ZFP69 7 11

1 40960902 exm49463 A G ZFP69 0 1

1 40960959 exm49465 A C ZFP69 0 1

1 40980715 exm49510 A G EXO5 19 38

1 40980724 exm49511 A C EXO5 3 5

1 41012533 exm49562 C A ZNF684 1 1

1 41483369 exm49898 A G SLFNL1 7 16

1 41483437 exm49905 A G SLFNL1 17 38

1 41486245 exm49967 A G SLFNL1 25 52

1 41945170 exm50095 A C EDN2 0 1

1 41949761 exm50113 A G EDN2 12 24

1 41949838 exm50116 A G EDN2 12 33

1 41976928 exm50188 A G HIVEP3 18 34

1 41979337 exm50244 G A HIVEP3 5 14

1 41984079 exm50249 A G HIVEP3 4 12

1 42045709 exm50280 A G HIVEP3 4 20

1 42047234 exm50347 A C HIVEP3 5 7

1 42047846 exm50376 A G HIVEP3 9 14

1 42048122 exm50392 A G HIVEP3 8 16

1 43204251 exm50954 G A CLDN19 1 2

1 1959617 exm5098 A G GABRD 2 0

1 43213914 exm51005 A G P3H1 0 1

1 43215930 exm51014 A G P3H1 20 48

1 43217985 exm51022 G A P3H1 8 19

1 43218010 exm51024 G C P3H1 1 0

1 43224953 exm51079 A G P3H1 4 10

1 1960633 exm5108 A G GABRD 2 9

1 43240962 exm51140 G A C1orf50 4 6

1 43240998 exm51143 A G C1orf50 0 1

1 43296594 exm51179 A G ERMAP 1 2

1 43301427 exm51192 A G ERMAP 8 13

1 43308524 exm51234 A G ERMAP 4 9

1 43647483 exm51487 G A CFAP57 13 29

1 43672499 exm51553 A G CFAP57 2 11

1 43675499 exm51566 A G CFAP57 0 1

1 43772818 exm51705 A G TIE1 10 26

1 43777350 exm51738 A G TIE1 1 1

1 43804340 exm51879 A G MPL 2 7

1 43805227 exm51895 A C MPL 7 17

1 43805698 exm51898 G A MPL 8 10

1 43891286 exm52149 G C SZT2 1 2

1 43896413 exm52243 A G SZT2 1 5

1 43902884 exm52321 A C SZT2 1 0

1 43903318 exm52325 C G SZT2 4 6

1 43913531 exm52468 G A SZT2 1 1

1 44474098 exm53530 A G SLC6A9 34 64

1 44474143 exm53534 A G SLC6A9 16 27

1 2234791 exm5368 A G SKI 1 1

1 44685725 exm53700 G A DMAP1 3 19

1 44685886 exm53709 A G DMAP1 4 3

1 2234824 exm5373 A G SKI 11 24

1 45213152 exm53940 G A KIF2C 2 5

1 45224990 exm53978 G A KIF2C 0 1

1 45228274 exm54013 T A KIF2C 11 11

1 45232574 exm54021 A G KIF2C 7 26

1 45288336 exm54311 G A PTCH2 15 38

1 45288988 exm54324 A G PTCH2 3 7

1 45292234 exm54348 A G PTCH2 0 2

1 45292576 exm54358 A G PTCH2 7 13

1 45293579 exm54409 A G PTCH2 8 14

1 45294261 exm54432 A G PTCH2 5 15

1 45294291 exm54434 A G PTCH2 2 4

1 45479354 exm54719 A G UROD 10 16

1 45479431 exm54722 A G UROD 1 9

1 2290143 exm5488 A G MORN1 21 36

1 2305932 exm5498 C A MORN1 24 46

1 45800156 exm55053 A G MUTYH 1 2

1 45808937 exm55114 A G TOE1 3 8

1 45809028 exm55122 G A TOE1 0 1

1 45810808 exm55147 A G TESK2 13 18

1 45810865 exm55149 A G TESK2 2 7

1 45851627 exm55203 G A TESK2 2 6

1 46027477 exm55337 A G AKR1A1 13 37

1 46032311 exm55344 G A AKR1A1 2 2

1 46034263 exm55370 A C AKR1A1 4 3

1 46034329 exm55372 A G AKR1A1 4 12

1 46035621 exm55390 A G AKR1A1 13 31

1 46082061 exm55481 G C NASP 5 6

1 46083166 exm55493 G A NASP 1 2

1 46099788 exm55605 A C GPBP1L1 12 32

1 46120885 exm55642 G A GPBP1L1 0 1

1 2338015 exm5594 G A PEX10 1 3

1 46499526 exm55949 G A MAST2 17 39

1 46500251 exm55966 A G MAST2 17 39

1 865700 exm56 A G SAMD11 10 26

1 46501599 exm56028 A G MAST2 3 2

1 2339908 exm5618 A G PEX10 1 3

1 46739409 exm56380 A G RAD54L 11 24

1 46874215 exm56654 A G FAAH 0 1

1 46874246 exm56655 A G FAAH 7 20

1 46977907 exm56757 A G DMBX1 2 4

1 47264766 exm57055 T A CYP4B1 11 15

1 47279699 exm57110 G A CYP4B1 12 26

1 47283818 exm57155 A G CYP4B1 1 7

1 47284367 exm57160 A G CYP4B1 10 19

1 47498925 exm57298 A G CYP4X1 6 12

1 47533310 exm57350 A G CYP4Z1 5 9

1 47716980 exm57573 A G STIL 1 1

1 47725974 exm57599 A C STIL 14 31

1 48694594 exm57856 A G SLC5A9 13 32

1 48697226 exm57878 G A SLC5A9 13 31

1 48697713 exm57891 A G SLC5A9 11 22

1 48764525 exm57986 A G SPATA6 3 9

1 48825355 exm57998 A G SPATA6 1 1

1 49119052 exm58086 A G AGBL4 5 10

1 51871634 exm58476 C A EPS15 14 32

1 52238313 exm58614 A G OSBPL9 8 14

1 52251499 exm58639 T A OSBPL9 8 13

1 52385693 exm58793 G A RAB3B 11 20

1 52385700 exm58794 G A RAB3B 5 4

1 52498721 exm58841 C G KTI12 1 1

1 52499058 exm58863 G C KTI12 7 12

1 52499253 exm58877 A C KTI12 4 6

1 52704153 exm58965 A G ZFYVE9 10 14

1 52704264 exm58970 G A ZFYVE9 19 36

1 52704350 exm58974 G A ZFYVE9 22 36

1 52747446 exm59042 G A ZFYVE9 0 2

1 52824767 exm59177 A G CC2D1B 3 3

1 52825833 exm59202 A G CC2D1B 28 41

1 52826729 exm59221 A G CC2D1B 1 3

1 52838992 exm59254 G A ORC1 6 6

1 52847342 exm59271 G A ORC1 1 4

1 52851608 exm59318 A G ORC1 0 1

1 52902525 exm59464 A G TUT4 3 3

1 52902540 exm59465 A G TUT4 1 4

1 52940543 exm59505 A T TUT4 12 18

1 52941198 exm59529 A G TUT4 3 11

1 53158534 exm59670 G A COA7 1 1

1 53527905 exm59937 G C PODN 20 31

1 53535699 exm59969 G C PODN 8 12

1 53541538 exm59994 A G PODN 4 14

1 53542915 exm60000 G A PODN 6 15

1 53554565 exm60099 A G SLC1A7 7 5

1 53569237 exm60180 A G SLC1A7 1 0

1 53580433 exm60188 A T SLC1A7 4 12

1 53580593 exm60205 A G SLC1A7 6 22

1 53580601 exm60207 A G SLC1A7 8 12

1 53676258 exm60273 A C CPT2 9 18

1 53676718 exm60300 C A CPT2 6 11

1 53676865 exm60313 A G CPT2 9 20

1 54301238 exm60822 A T NDC1 2 2

1 54433454 exm60990 G C LRRC42 7 24

1 54534492 exm61101 G A TCEANC2 16 29

1 54644859 exm61248 A G CYB5RL 7 11

1 54644923 exm61251 A C CYB5RL 7 11

1 54656446 exm61267 A G CYB5RL 17 31

1 54656470 exm61268 A G CYB5RL 8 9

1 54661234 exm61280 A G CYB5RL 1 2

1 54670744 exm61311 A G MRPL37 0 1

1 54678184 exm61332 C A MRPL37 4 8

1 54678318 exm61342 A G MRPL37 4 11

1 55014013 exm61537 A G ACOT11 13 23

1 55072826 exm61632 A G ACOT11 1 2

1 55073660 exm61649 A C ACOT11 4 7

1 55075006 exm61673 A G FAM151A 1 3

1 55118982 exm61837 G A MROH7 7 7

1 55119693 exm61866 G A MROH7 0 1

1 55139471 exm61907 A G MROH7 12 29

1 55144968 exm61931 G A MROH7 12 18

1 55145619 exm61943 A G MROH7 22 30

1 55151986 exm61956 A G MROH7 7 22

1 55166016 exm61986 A G MROH7 8 15

1 55172208 exm62041 G A MROH7 7 8

1 55175775 exm62058 C G MROH7 2 2

1 55175807 exm62061 A G MROH7 16 35

1 55197246 exm62104 C A TTC4 3 8

1 55251766 exm62222 G A TTC22 24 37

1 55253386 exm62239 A G TTC22 7 8

1 55273670 exm62319 A G LEXM 1 2

1 55277818 exm62339 A G LEXM 7 13

1 55282719 exm62353 A G LEXM 2 3

1 55451770 exm62465 G A TMEM61 16 32

1 55474053 exm62561 A G BSND 2 6

1 55512222 exm62606 G C PCSK9 5 9

1 55518422 exm62636 A G PCSK9 11 28

1 55523779 exm62666 A C PCSK9 14 15

1 55524222 exm62678 A G PCSK9 17 29

1 55525315 exm62700 C G PCSK9 6 12

1 55529215 exm62720 A C PCSK9 11 34

1 55603229 exm62853 G A USP24 10 20

1 55631586 exm62928 G A USP24 13 30

1 57209939 exm63129 A G FYB2 12 25

1 57216786 exm63133 G A FYB2 3 8

1 57258320 exm63192 G A FYB2 9 9

1 57333311 exm63206 A C C8A 7 6

1 57383295 exm63300 A G C8A 9 29

1 57411642 exm63360 C G C8B 1 8

1 57415368 exm63379 A G C8B 1 4

1 57425792 exm63418 C G C8B 5 9

1 57425810 exm63419 G C C8B 20 32

1 58946710 exm63542 G A OMA1 5 6

1 58971748 exm63549 A T OMA1 0 1

1 59002200 exm63585 C A OMA1 3 6

1 59002282 exm63591 C A OMA1 10 28

1 905669 exm636 C G PLEKHN1 7 14

1 59004602 exm63610 C A OMA1 9 22

1 59004804 exm63616 A G OMA1 9 18

1 59134708 exm63705 A G MYSM1 22 36

1 59147497 exm63726 G C MYSM1 8 7

1 59147962 exm63747 C A MYSM1 0 2

1 59148136 exm63755 G A MYSM1 1 4

1 905723 exm638 A G PLEKHN1 0 2

1 59787414 exm63822 A G FGGY 4 2

1 60019853 exm63860 A G FGGY 2 2

1 60091687 exm63876 A C FGGY 6 9

1 60103910 exm63880 G A FGGY 0 1

1 2938506 exm6394 A G ACTRT2 14 38

1 60331556 exm63993 G A HOOK1 10 23

1 60370671 exm64041 A G CYP2J2 5 12

1 60370710 exm64046 A G CYP2J2 6 17

1 60377960 exm64070 A G CYP2J2 1 4

1 60392390 exm64090 A C CYP2J2 1 8

1 60456365 exm64097 A G C1orf87 5 10

1 60463283 exm64110 A G C1orf87 3 8

1 60505774 exm64158 A G C1orf87 5 17

1 62253483 exm64355 A G PATJ 29 69

1 62257036 exm64365 C A PATJ 25 50

1 62267355 exm64389 G A PATJ 5 7

1 62380262 exm64483 G A PATJ 0 1

1 62455935 exm64551 G A PATJ 14 35

1 62579891 exm64596 A C PATJ 0 5

1 62582847 exm64617 C G PATJ 12 44

1 62593737 exm64634 A G PATJ 7 12

1 62673126 exm64693 G A L1TD1 10 19

1 62676688 exm64761 G A L1TD1 7 21

1 62703977 exm64783 A G KANK4 12 26

1 62704046 exm64789 A G KANK4 3 8

1 62737151 exm64853 A G KANK4 3 9

1 62739302 exm64885 A G KANK4 0 1

1 62739542 exm64899 A G KANK4 4 12

1 62739801 exm64912 G C KANK4 6 7

1 62740027 exm64925 A G KANK4 8 20

1 62740264 exm64944 G A KANK4 15 29

1 62962089 exm65118 A T DOCK7 4 4

1 62993823 exm65153 A G DOCK7 15 30

1 3328187 exm6534 A G PRDM16 8 7

1 63128715 exm65360 G A DOCK7 5 3

1 63991328 exm65609 C G EFCAB7 13 18

1 63991379 exm65611 A G EFCAB7 8 16

1 64011693 exm65647 G A EFCAB7 2 6

1 64017419 exm65651 A G EFCAB7 0 1

1 64034140 exm65671 A G EFCAB7 17 33

1 65145283 exm66073 A G CACHD1 2 8

1 65255129 exm66145 A G RAVER2 8 17

1 65273018 exm66163 A G RAVER2 7 10

1 65871579 exm66423 A T DNAJC6 22 36

1 3342659 exm6666 A G PRDM16 11 13

1 67292585 exm66937 A G WDR78 8 18

1 67313256 exm66994 C A WDR78 15 28

1 67313349 exm66998 C G WDR78 7 23

1 67359042 exm67018 A C WDR78 3 6

1 67705958 exm67254 A G IL23R 15 35

1 67786061 exm67275 G A IL12RB2 1 2

1 67796490 exm67321 A G IL12RB2 1 2

1 67816631 exm67327 G A IL12RB2 1 11

1 67833643 exm67345 A G IL12RB2 1 0

1 68603512 exm67526 A G WLS 0 1

1 68659719 exm67576 C G WLS 12 31

1 68944843 exm67682 G A DEPDC1 7 11

1 68947133 exm67689 A G DEPDC1 11 29

1 70504688 exm67850 G A LRRC7 3 2

1 70621412 exm67930 G A LRRC40 10 21

1 70639372 exm67946 G A LRRC40 2 2

1 70646822 exm67967 G A LRRC40 6 21

1 70654950 exm67985 C A LRRC40 8 23

1 70671201 exm67993 A G LRRC40 4 8

1 70895478 exm68147 A G CTH 0 5

1 70896038 exm68152 G A CTH 8 19

1 72058610 exm68319 A G NEGR1 2 15

1 74648325 exm68425 G A LRRIQ3 3 7

1 74649209 exm68443 A G LRRIQ3 1 8

1 74670617 exm68492 G A FPGT 4 18

1 74905291 exm68632 A G FPGT-TNNI3K;TNNI3K 3 12

1 74929170 exm68638 G A FPGT-TNNI3K;TNNI3K 9 31

1 75009655 exm68689 A C FPGT-TNNI3K;TNNI3K 1 7

1 75037108 exm68709 A G ERICH3 1 0

1 75037756 exm68739 G A ERICH3 14 22

1 75037757 exm68740 A G ERICH3 0 1

1 75038603 exm68784 A G ERICH3 2 1

1 75038747 exm68796 A G ERICH3 3 9

1 75065459 exm68873 A T ERICH3 4 15

1 75086586 exm68915 G A ERICH3 7 12

1 76262773 exm69332 A C MSH4 2 3

1 76272681 exm69351 A G MSH4 9 11

1 76343943 exm69391 G A MSH4 15 26

1 78097803 exm69777 G A ZZZ3 7 15

1 78098403 exm69804 G A ZZZ3 24 49

1 78098409 exm69805 A G ZZZ3 2 10

1 79093821 exm70324 G A IFI44L 5 8

1 79093910 exm70327 A G IFI44L 23 46

1 79095439 exm70348 G A IFI44L 5 11

1 3425238 exm7035 A G MEGF6 6 19

1 79095565 exm70358 A G IFI44L 0 2

1 79095572 exm70359 A G IFI44L 19 31

1 79358827 exm70494 A C ADGRL4 1 1

1 79385972 exm70516 C A ADGRL4 16 41

1 79386047 exm70519 G A ADGRL4 1 0

1 79402069 exm70541 G A ADGRL4 8 20

1 79403796 exm70550 G A ADGRL4 7 29

1 79411968 exm70562 A C ADGRL4 1 2

1 82456432 exm70721 G A ADGRL2 5 14

1 85397076 exm71248 A G MCOLN2 3 8

1 85412716 exm71286 T A MCOLN2 23 32

1 85412803 exm71290 A T MCOLN2 0 1

1 85498408 exm71382 G C MCOLN3 15 25

1 85498591 exm71387 A G MCOLN3 3 9

1 85499910 exm71394 A G MCOLN3 1 1

1 86241379 exm71879 G A COL24A1 4 7

1 86334472 exm71932 A C COL24A1 5 15

1 86590940 exm72061 G A COL24A1 7 15

1 86590959 exm72066 A G COL24A1 2 10

1 86591142 exm72078 G A COL24A1 18 28

1 86818544 exm72127 G A ODF2L 3 5

1 86852625 exm72222 A G ODF2L 1 1

1 86891073 exm72255 A G CLCA2 30 42

1 86894231 exm72264 A G CLCA2 17 31

1 86907149 exm72322 A T CLCA2 12 17

1 86965343 exm72499 G A CLCA1 8 18

1 87043728 exm72603 A G CLCA4 1 2

1 87045727 exm72617 C G CLCA4 3 8

1 87329250 exm72703 A G SELENOF 1 2

1 87369131 exm72711 A C SELENOF 1 1

1 87380813 exm72739 A C HS2ST1 5 19

1 87569237 exm72769 A G HS2ST1 15 24

1 89475080 exm73093 G A GBP3 11 28

1 89479102 exm73145 A G GBP3 7 12

1 89481013 exm73168 G C GBP3 8 11

1 89525989 exm73300 A G GBP1 6 24

1 89528914 exm73316 A C GBP1 3 14

1 89575547 exm73342 A G GBP2 0 3

1 89575548 exm73343 A G GBP2 0 2

1 89654395 exm73538 A G GBP4 6 10

1 89657124 exm73567 A G GBP4 1 0

1 89660988 exm73585 A C GBP4 17 41

1 89662813 exm73592 A G GBP4 3 6

1 89734459 exm73695 A C GBP5 8 21

1 89735085 exm73700 G A GBP5 15 24

1 89844035 exm73732 G A GBP6 9 24

1 89844088 exm73734 A G GBP6 5 7

1 89846152 exm73750 A G GBP6 23 61

1 90179420 exm73932 G A LRRC8C 7 14

1 90398839 exm73989 A G LRRC8D 1 6

1 91404617 exm74230 C A ZNF644 1 2

1 91404768 exm74238 A G ZNF644 7 13

1 91405961 exm74279 G A ZNF644 1 7

1 91406006 exm74283 A G ZNF644 3 7

1 91406186 exm74292 A G ZNF644 9 23

1 91840949 exm74445 A C HFM1 11 15

1 91843626 exm74455 G A HFM1 11 16

1 92195428 exm74662 A G TGFBR3 2 8

1 92200413 exm74670 A C TGFBR3 1 4

1 92442802 exm74731 A G BRDT 12 22

1 92442902 exm74736 T A BRDT 1 11

1 92467669 exm74810 A G BRDT 8 11

1 92554358 exm74877 G A BTBD8 18 35

1 92568089 exm74881 G A BTBD8 1 4

1 92604915 exm74890 G A BTBD8 13 37

1 92647636 exm74960 C A KIAA1107 3 11

1 92735301 exm75047 C A GLMN 9 7

1 92737046 exm75051 G A GLMN 2 9

1 92756989 exm75072 A G GLMN 1 2

1 3672048 exm7522 A G CCDC27 16 29

1 93089887 exm75253 G A EVI5 6 17

1 93657581 exm75517 G C CCDC18 1 2

1 3677983 exm7554 A G CCDC27 17 36

1 93677761 exm75549 G A CCDC18 5 9

1 93680333 exm75552 G A CCDC18 6 13

1 93680515 exm75557 A G CCDC18 7 10

1 93704878 exm75591 G A CCDC18 15 36

1 93724389 exm75620 G A CCDC18 3 15

1 93730286 exm75622 G A CCDC18 1 12

1 94037330 exm75745 A G BCAR3 3 11

1 94037397 exm75750 A G BCAR3 15 24

1 94054577 exm75801 A G BCAR3 13 32

1 94054765 exm75816 A G BCAR3 8 20

1 3679925 exm7582 G A CCDC27 10 26

1 94057835 exm75831 A G BCAR3 2 3

1 94140470 exm75855 A T BCAR3 1 1

1 94335436 exm75867 C A DNTTIP2 9 21

1 94342647 exm75909 A G DNTTIP2 2 4

1 94343294 exm75925 G A DNTTIP2 1 0

1 3680345 exm7598 A G CCDC27 1 0

1 94473846 exm76031 A G ABCA4 1 1

1 94485257 exm76083 A G ABCA4 1 1

1 94495073 exm76126 A C ABCA4 9 16

1 94510248 exm76225 G C ABCA4 15 18

1 3684005 exm7623 A C CCDC27 2 4

1 94514477 exm76253 A G ABCA4 2 3

1 94548928 exm76363 T A ABCA4 7 12

1 94564544 exm76383 A G ABCA4 19 26

1 94640188 exm76437 A G ARHGAP29 12 19

1 94640254 exm76442 G A ARHGAP29 3 7

1 94643209 exm76448 A G ARHGAP29 9 18

1 94643466 exm76453 A G ARHGAP29 10 16

1 94668734 exm76520 C A ARHGAP29 1 3

1 95290064 exm76673 G A SLC44A3 13 20

1 95322902 exm76722 A G SLC44A3 2 10

1 95322939 exm76723 A G SLC44A3 5 12

1 95358036 exm76754 A G SLC44A3 2 3

1 97544632 exm76936 C A DPYD 1 2

1 97770919 exm76966 C A DPYD 2 5

1 97981407 exm77004 A G DPYD 6 10

1 97981421 exm77005 A G DPYD 9 22

1 98015291 exm77021 A G DPYD 4 6

1 100183072 exm77386 G A FRRS1 2 5

1 100194151 exm77395 A C FRRS1 8 22

1 100214243 exm77437 A G FRRS1 0 3

1 100214289 exm77441 G A FRRS1 0 3

1 100340252 exm77495 A G AGL 4 6

1 100340787 exm77503 A G AGL 0 1

1 100346262 exm77531 C A AGL 1 8

1 100346337 exm77535 A G AGL 10 17

1 100358107 exm77580 G A AGL 6 13

1 100382037 exm77636 G A AGL 3 2

1 100382256 exm77645 A G AGL 2 1

1 100676303 exm77954 G A DBT 5 4

1 100681641 exm77967 A C DBT 2 4

1 100756991 exm78058 T A RTCA 3 7

1 100818541 exm78067 A G CDC14A 3 2

1 100856373 exm78079 C G CDC14A 0 7

1 100949904 exm78120 A G CDC14A 4 7

1 100964826 exm78151 A G CDC14A 15 39

1 101185447 exm78182 A G VCAM1 6 9

1 101198111 exm78247 C G VCAM1 0 3

1 101339737 exm78284 G A EXTL2 5 8

1 3751634 exm7833 A G CEP104 9 15

1 3753157 exm7846 A G CEP104 0 1

1 103491777 exm78764 G A COL11A1 4 10

1 108185314 exm79177 A G VAV3 4 1

1 108417605 exm79252 A G VAV3 1 1

1 108690906 exm79283 G A SLC25A24 3 9

1 109154881 exm79433 C A FAM102B 2 3

1 109170871 exm79442 A C FAM102B 14 25

1 109197351 exm79495 G A HENMT1 9 21

1 109200108 exm79510 A G HENMT1 8 8

1 109260477 exm79591 G C FNDC7 0 1

1 109261509 exm79601 G A FNDC7 12 42

1 109270464 exm79630 C G FNDC7 5 13

1 109358897 exm79765 C G AKNAD1 14 30

1 109394964 exm79887 A G AKNAD1 8 27

1 109394976 exm79888 A G AKNAD1 4 13

1 109446750 exm79944 A G GPSM2 7 27

1 109466760 exm79978 A G GPSM2 5 14

1 109472378 exm79983 A G GPSM2 1 2

1 3806624 exm8010 A G C1orf174 1 0

1 109707313 exm80249 A G KIAA1324 1 2

1 109727733 exm80275 A G KIAA1324 2 4

1 109735267 exm80314 G A KIAA1324 2 9

1 109740138 exm80339 A G KIAA1324 11 22

1 109772103 exm80400 A G SARS 0 2

1 109793128 exm80470 A G CELSR2 0 1

1 109801345 exm80613 A G CELSR2 1 1

1 109808448 exm80745 G A CELSR2 15 27

1 109815868 exm80897 C G CELSR2 19 42

1 109823458 exm80934 A G PSRC1 1 3

1 109824422 exm80974 A G PSRC1 4 8

1 109865630 exm81112 A G SORT1 3 16

1 109867673 exm81122 A G SORT1 11 22

1 4772585 exm8120 G A AJAP1 14 34

1 110030311 exm81288 C A ATXN7L2 10 27

1 110032976 exm81319 A C ATXN7L2 2 9

1 4832549 exm8143 A G AJAP1 10 15

1 110116625 exm81525 A G GNAI3 8 21

1 110121922 exm81531 A G GNAI3 1 5

1 110151365 exm81585 A G GNAT2 0 2

1 110151395 exm81589 A C GNAT2 7 19

1 5924483 exm8173 G A NPHP4 19 23

1 5927169 exm8201 A G NPHP4 5 9

1 5927943 exm8208 A G NPHP4 0 2

1 110301919 exm82130 A G EPS8L3 8 28

1 110301983 exm82132 G C EPS8L3 17 38

1 110301991 exm82133 A G EPS8L3 4 9

1 110302450 exm82145 A G EPS8L3 5 9

1 5937289 exm8271 G A NPHP4 10 21

1 110906406 exm82801 A G SLC16A4 4 10

1 110924406 exm82852 A C SLC16A4 6 13

1 110946636 exm82871 C G LAMTOR5 4 8

1 5940266 exm8288 A G NPHP4 5 15

1 110950259 exm82881 G A LAMTOR5 11 18

1 111059966 exm82932 G A KCNA10 4 7

1 111061161 exm83009 C A KCNA10 0 1

1 5951013 exm8315 A G NPHP4 2 6

1 111492547 exm83183 G A LRIF1 4 22

1 5965440 exm8342 G A NPHP4 9 20

1 111781397 exm83460 C A CHI3L2 15 20

1 111781504 exm83465 A G CHI3L2 11 23

1 111861217 exm83565 G A CHIA 4 7

1 111861248 exm83567 A G CHIA 4 8

1 5965833 exm8359 A G NPHP4 20 40

1 111862064 exm83592 G A CHIA 11 12

1 111862973 exm83607 G C CHIA 3 6

1 111889636 exm83625 G A PIFO 4 7

1 111957879 exm83711 A G OVGP1 12 17

1 111962256 exm83724 A C OVGP1 17 31

1 111965646 exm83743 A G OVGP1 3 7

1 112026370 exm83888 A G TMIGD3 18 26

1 112028460 exm83900 A G TMIGD3 4 6

1 112269757 exm84025 A G INKA2 2 8

1 112302035 exm84100 C G DDX20 7 19

1 112303415 exm84113 C G DDX20 13 22

1 112303861 exm84122 A G DDX20 18 37

1 112309184 exm84182 G A DDX20 17 35

1 113057708 exm84404 G A WNT2B 2 5

1 113068676 exm84446 A G ST7L 1 7

1 113125080 exm84482 A G ST7L 7 20

1 113143441 exm84501 A G ST7L 8 17

1 113161699 exm84525 A G ST7L 9 21

1 113255456 exm84788 A C PPM1J 25 57

1 113264856 exm84808 G A TAFA3 1 0

1 113456602 exm84847 A G SLC16A1 2 0

1 113636134 exm84917 A T LRIG2 15 30

1 113638635 exm84940 A G LRIG2 1 4

1 113659023 exm85009 A G LRIG2 13 22

1 113666613 exm85030 A G LRIG2 8 19

1 114133195 exm85095 G A MAGI3 6 13

1 114184827 exm85115 A G MAGI3 1 8

1 114375747 exm85413 A G PTPN22 9 14

1 114394689 exm85468 A G PTPN22 1 5

1 114399229 exm85488 A G PTPN22 3 6

1 114414169 exm85500 G A PTPN22 3 7

1 114438064 exm85540 C A AP4B1 3 5

1 114440547 exm85570 A G AP4B1 2 7

1 114442673 exm85584 A T AP4B1 0 3

1 114442742 exm85589 A C AP4B1 11 17

1 114448279 exm85641 A G DCLRE1B 1 3

1 114454062 exm85672 A G DCLRE1B 0 2

1 114524275 exm85883 G A OLFML3 19 29

1 114524315 exm85886 A G OLFML3 10 22

1 6181177 exm8597 A C CHD5 1 0

1 114945393 exm85988 A G TRIM33 11 32

1 114948210 exm86001 G A TRIM33 6 7

1 114948338 exm86006 G A TRIM33 5 16

1 6181252 exm8602 A G CHD5 2 3

1 115138455 exm86106 A G DENND2C 3 4

1 115144789 exm86140 A G DENND2C 1 3

1 115168119 exm86199 A T DENND2C 6 16

1 115168226 exm86204 A G DENND2C 22 38

1 115220593 exm86274 A G AMPD1 12 21

1 115221116 exm86287 A C AMPD1 4 2

1 115222959 exm86301 G A AMPD1 0 1

1 115229413 exm86320 A G AMPD1 11 22

1 115229512 exm86324 A G AMPD1 10 22

1 115229523 exm86325 A G AMPD1 6 23

1 115231254 exm86332 A G AMPD1 14 40

1 115236057 exm86345 A G AMPD1 14 36

1 116206384 exm86719 A G VANGL1 10 18

1 116206614 exm86736 A G VANGL1 2 1

1 116280895 exm86847 G A CASQ2 0 1

1 116310937 exm86859 A G CASQ2 0 1

1 116569622 exm86923 A G SLC22A15 6 12

1 116609280 exm86956 A G SLC22A15 15 25

1 117552675 exm87589 A G CD101 0 1

1 117552727 exm87593 G A CD101 25 43

1 117560990 exm87674 A G CD101 15 24

1 117568358 exm87704 A G CD101 15 14

1 117576619 exm87724 A G CD101 29 40

1 117617598 exm87757 G A TTF2 10 22

1 117618235 exm87781 C G TTF2 3 9

1 117620574 exm87807 G A TTF2 1 5

1 117626751 exm87830 G A TTF2 2 17

1 117633170 exm87852 A G TTF2 1 6

1 117635449 exm87873 A G TTF2 4 8

1 874456 exm88 A G SAMD11 5 11

1 118065541 exm88102 A G MAN1A2 2 3

1 118166547 exm88147 A G TENT5C 3 9

1 118476052 exm88226 A G WDR3 13 28

1 118483474 exm88255 G C WDR3 6 22

1 118483544 exm88257 A G WDR3 5 6

1 118506441 exm88360 A G SPAG17 9 19

1 118506531 exm88364 A G SPAG17 1 1

1 118527339 exm88409 A T SPAG17 7 15

1 118534138 exm88425 G A SPAG17 3 5

1 118537076 exm88438 A G SPAG17 8 21

1 118574322 exm88511 A T SPAG17 6 19

1 118583458 exm88536 A G SPAG17 3 3

1 118598452 exm88557 G A SPAG17 7 20

1 118657952 exm88649 A G SPAG17 27 51

1 118727720 exm88658 C A SPAG17 11 17

1 119427839 exm88686 A G TBX15 22 42

1 119441695 exm88703 A G TBX15 16 24

1 119469188 exm88721 A C TBX15 10 24

1 119575863 exm88750 A G WARS2 3 11

1 119683231 exm88783 C A WARS2 0 6

1 120254732 exm89058 A C PHGDH 8 18

1 120286620 exm89157 A C PHGDH 10 22

1 120300018 exm89188 A G HMGCS2 21 30

1 120341140 exm89246 A G REG4 11 18

1 120341176 exm89247 A C REG4 3 13

1 120378787 exm89303 A G NBPF7 4 8

1 120379932 exm89309 A C NBPF7 10 14

1 120436918 exm89394 A G ADAM30 12 35

1 120458579 exm89517 A G NOTCH2 1 3

1 120464998 exm89582 G A NOTCH2 6 10

1 120484300 exm89675 A C NOTCH2 5 10

1 120509025 exm89721 G A NOTCH2 3 4

1 144911949 exm90437 G A PDE4DIP 15 24

1 144930756 exm90565 A G PDE4DIP 0 1

1 145415669 exm91023 G C HJV 1 2

1 145416337 exm91030 A C HJV 17 32

1 145525085 exm91279 A G ITGA10 20 40

1 145530939 exm91313 A G ITGA10 16 27

1 145533451 exm91341 A G ITGA10 2 4

1 145534914 exm91362 A T ITGA10 4 11

1 145561192 exm91517 G A ANKRD35 9 18

1 145562149 exm91566 A C ANKRD35 13 30

1 145578333 exm91648 A G PIAS3 7 12

1 145585576 exm91720 A C PIAS3 2 5

1 145585582 exm91721 A G PIAS3 16 37

1 145587383 exm91756 A G NUDT17 1 9

1 145588901 exm91783 A G NUDT17 14 21

1 145608122 exm91861 C A POLR3C 3 2

1 145608146 exm91862 G A POLR3C 2 3

1 145698959 exm91941 A G CD160 21 38

1 145704146 exm91952 G A CD160 1 1

1 145704304 exm91963 A G CD160 4 6

1 146658563 exm92138 A C FMO5 6 14

1 146680559 exm92186 A G FMO5 2 6

1 146687509 exm92208 G A FMO5 10 26

1 146758168 exm92348 A G CHD1L 0 1

1 146765310 exm92368 A G CHD1L 6 18

1 146765358 exm92371 C G CHD1L 2 6

1 147090956 exm92429 A G BCL9 1 4

1 147091108 exm92438 A G BCL9 11 17

1 147092372 exm92497 G A BCL9 1 0

1 147096112 exm92552 A G BCL9 3 20

1 147096575 exm92572 C G BCL9 9 13

1 147119257 exm92590 T A ACP6 0 1

1 147120196 exm92609 A G ACP6 12 32

1 147126437 exm92626 A G ACP6 5 20

1 147127320 exm92629 G C ACP6 4 12

1 147131811 exm92652 A G ACP6 8 10

1 147380101 exm92700 A C GJA8 1 3

1 147380975 exm92737 G A GJA8 3 12

1 147381239 exm92750 A G GJA8 6 17

1 149905827 exm93877 A C MTMR11 3 3

1 149906902 exm93889 A G MTMR11 8 28

1 150049220 exm94035 G A VPS45 1 2

1 150429944 exm94425 G A RPRD2 7 27

1 150445514 exm94503 A G RPRD2 22 41

1 150471410 exm94574 A G TARS2 7 11

1 150525425 exm94711 A G ADAMTSL4 8 26

1 150525935 exm94729 A G ADAMTSL4 3 10

1 150526270 exm94752 A G ADAMTSL4 2 11

1 150530584 exm94843 A G ADAMTSL4 0 1

1 150530967 exm94854 A G ADAMTSL4 5 11

1 150531113 exm94869 C G ADAMTSL4 4 7

1 150789560 exm95209 A G ARNT 4 8

1 150804813 exm95244 A C ARNT 9 17

1 150921669 exm95338 A G SETDB1 4 7

1 150923100 exm95353 A C SETDB1 7 11

1 150955582 exm95494 G A ANXA9 4 7

1 150955610 exm95499 A G ANXA9 0 1

1 150956535 exm95515 A C ANXA9 4 16

1 150990292 exm95624 A G PRUNE1 14 25

1 150998110 exm95649 G A PRUNE1 18 27

1 151009208 exm95682 G A BNIPL 3 4

1 151011347 exm95698 A G BNIPL 4 6

1 151015564 exm95712 A G BNIPL 1 0

1 151020856 exm95764 A G C1orf56 8 18

1 151021096 exm95772 C A C1orf56 4 6

1 970687 exm958 A G AGRN 24 44

1 151062922 exm95814 G A GABPB2 1 5

1 151090542 exm95857 A G GABPB2 5 14

1 151090581 exm95861 A G GABPB2 2 12

1 151259628 exm96394 T A ZNF687 1 0

1 151259666 exm96395 A C ZNF687 1 5

1 151263315 exm96504 A G ZNF687 8 17

1 151263456 exm96514 A G ZNF687 4 2

1 151315134 exm96633 A G RFX5 4 9

1 151315287 exm96637 G C RFX5 12 47

1 151315354 exm96641 G A RFX5 1 3

1 151379094 exm96891 G A POGZ 3 7

1 151397526 exm96935 G A POGZ 2 5

1 151491026 exm96981 A G CGN 6 21

1 151491027 exm96982 A G CGN 7 9

1 151491318 exm97000 C A CGN 16 33

1 151491788 exm97023 A G CGN 11 22

1 151502427 exm97106 A G CGN 3 10

1 152058563 exm97834 A G TCHHL1 12 33

1 152059329 exm97866 A G TCHHL1 10 26

1 6615467 exm9789 A G TAS1R1 8 18

1 152080512 exm97940 A C TCHH 8 21

1 152081701 exm98036 G C TCHH 0 3

1 152082068 exm98052 G A TCHH 1 9

1 6631157 exm9806 G A TAS1R1 9 29

1 152082957 exm98126 C G TCHH 13 31

1 152083457 exm98156 C G TCHH 2 8

1 152083641 exm98167 C G TCHH 2 6

1 152128121 exm98335 A G RPTN 1 2

1 152128422 exm98360 C G RPTN 7 24

1 152185641 exm98434 A C HRNR 18 38

1 6635259 exm9850 G C TAS1R1 1 1

1 6635294 exm9851 C G TAS1R1 3 16

1 6636662 exm9875 G A TAS1R1 8 22

1 6637056 exm9881 A G TAS1R1 5 17

1 152191640 exm98933 A C HRNR 4 7

1 152192549 exm99064 C A HRNR 24 56

1 6639436 exm9930 A G TAS1R1 5 7

1 6641348 exm9965 A G ZBTB48 1 1

1 152281730 exm99971 T A FLG 7 9

1 152281856 exm99988 C A FLG 11 21

1 119427467 JHU_1.119427466 C A TBX15 2 6

1 159799910 JHU_1.159799909 A G SLAMF8 3 11

1 180163390 JHU_1.180163389 G A QSOX1 27 60

1 53980389 JHU_1.53980388 A G GLIS1 0 2

1 94512565 JHU_1.94512564 A G ABCA4 2 10

1 171086461 kgp15922089 A G FMO3 3 7

1 226016558 kgp25117122 C G EPHX1 9 42

1 89476589 newrs12087251 A G GBP3 20 33

1 63063509 newrs139334976 G A ANGPTL3 1 4

1 180165699 rs3738115 C A QSOX1 12 37

1 33237269 Variant37841 A G KIAA1522 1 0

1 155206060 Variant49117 A G GBA 0 4

2 102031360 2:102031360-CT A G RFX8 18 28

2 102038894 2:102038894-GT A C RFX8 19 28

2 108881399 2:108881399-CT A G SULT1C3 5 13

2 109088331 2:109088331-AG G A GCC2 1 2

2 111562932 2:111562932-CT A G ACOXL 10 17

2 11284225 2:11284225-GA A G C2orf50 7 10

2 113321966 2:113321966-CT A G POLR1B 10 18

2 113514547 2:113514547-AC C A CKAP2L 12 28

2 116497319 2:116497319-AC C A DPP10 13 33

2 120125791 2:120125791-GA A G DBI 1 2

2 120362293 2:120362293-CA A C CFAP221 5 10

2 120714478 2:120714478-TC G A PTPN4 0 1

2 128388437 2:128388437-AC C A MYO7B 9 26

2 133539621 2:133539621-CT A G NCKAP5 10 21

2 135781051 2:135781051-GA A G MAP3K19 4 4

2 141259438 2:141259438-AG G A LRP1B 1 4

2 141473664 2:141473664-TC G A LRP1B 13 26

2 141773450 2:141773450-TC G A LRP1B 10 18

2 148712961 2:148712961-TC G A ORC4 2 6

2 149227151 2:149227151-GA A G MBD5 0 1

2 152112226 2:152112226-GT A C RBM43 5 11

2 152313108 2:152313108-AG G A RIF1 6 7

2 152355870 2:152355870-GA A G NEB 16 33

2 152359900 2:152359900-AG G A NEB 1 0

2 152409968 2:152409968-CT A G NEB 6 24

2 152982814 2:152982814-GA A G STAM2 1 2

2 160087329 2:160087329-GA A G TANC1 5 11

2 163134176 2:163134176-CT A G IFIH1 5 20

2 168108221 2:168108221-AG G A XIRP2 3 3

2 170044497 2:170044497-CT A G LRP2 6 19

2 170063719 2:170063719-TC G A LRP2 0 1

2 170090091 2:170090091-CT A G LRP2 2 0

2 170129547 2:170129547-CT A G LRP2 0 1

2 170131548 2:170131548-TC G A LRP2 11 22

2 170163815 2:170163815-CT A G LRP2 12 32

2 170177304 2:170177304-GA A G LRP2 10 16

2 170806461 2:170806461-CT A G UBR3 0 1

2 178417010 2:178417010-CT A G TTC30B 14 34

2 179391962 2:179391962-CT A G TTN 1 4

2 179401845 2:179401845-AG G A TTN 13 20

2 179417446 2:179417446-CT A G TTN 0 1

2 179418854 2:179418854-CT A G TTN 0 3

2 179430442 2:179430442-TC G A TTN 1 7

2 179440955 2:179440955-CT A G TTN 0 1

2 179441679 2:179441679-GT A C TTN 8 24

2 179446705 2:179446705-TC G A TTN 2 4

2 179458403 2:179458403-AG G A TTN 0 1

2 179500810 2:179500810-CT A G TTN 2 2

2 179529387 2:179529387-CT A G TTN 3 5

2 179534380 2:179534380-AC C A TTN 4 14

2 179568916 2:179568916-TG C A TTN 1 5

2 179576932 2:179576932-TC G A TTN 0 3

2 179589058 2:179589058-GA A G TTN 7 12

2 183605075 2:183605075-AG G A DNAJC10 6 13

2 18757524 2:18757524-GA A G NT5C1B;NT5C1B-RDH14 1 1

2 187627477 2:187627477-CA A C FAM171B 0 2

2 187692819 2:187692819-CT A G ZSWIM2 5 5

2 190541532 2:190541532-GA A G ANKAR 3 1

2 197183701 2:197183701-AG G A HECW2 19 32

2 197986225 2:197986225-GA A G ANKRD44 0 1

2 198593274 2:198593274-TG C A BOLL 8 15

2 198631285 2:198631285-TC G A BOLL 6 17

2 20113365 2:20113365-AC C A WDR35 3 4

2 201790584 2:201790584-GT A C ORC2 5 19

2 20192921 2:20192921-TG C A MATN3 1 4

2 202492092 2:202492092-CT A G TMEM237 0 2

2 203996775 2:203996775-CT A G NBEAL1 1 3

2 204032070 2:204032070-AG G A NBEAL1 8 25

2 206614493 2:206614493-GA A G NRP2 4 9

2 207170555 2:207170555-CT A G ZDBF2 0 1

2 207621974 2:207621974-AG G A MDH1B 16 36

2 20845111 2:20845111-CT A G HS1BP3 2 5

2 20886706 2:20886706-TC G A LDAH 4 11

2 210517908 2:210517908-GA A G MAP2 1 2

2 21228020 2:21228020-TC G A APOB 1 0

2 215595164 2:215595164-GA A G BARD1 1 1

2 215840669 2:215840669-GA A G ABCA12 3 6

2 216213910 2:216213910-GA A G ATIC 6 17

2 216240375 2:216240375-GT A C FN1 2 3

2 217234805 2:217234805-GA A G MARCHF4 7 15

2 219878665 2:219878665-TC G A CFAP65 1 2

2 219892344 2:219892344-AG G A CFAP65 10 15

2 219892620 2:219892620-GA A G CFAP65 5 17

2 219892683 2:219892683-TC G A CFAP65 4 6

2 220072127 2:220072127-GT A C ZFAND2B 13 27

2 220099808 2:220099808-CT A G ANKZF1 2 11

2 220419294 2:220419294-CT A G OBSL1 3 5

2 227660781 2:227660781-TC G A IRS1 0 2

2 228113222 2:228113222-GA A G COL4A3 4 4

2 228563995 2:228563995-CT A G SLC19A3 1 3

2 231192003 2:231192003-GA A G SP140L 1 6

2 232393416 2:232393416-CT A G NMUR1 2 6

2 232995433 2:232995433-AG G A DIS3L2 4 10

2 233744249 2:233744249-GA A G NGEF 0 1

2 233839504 2:233839504-CT A G NGEF 10 17

2 234438129 2:234438129-AG G A USP40 3 3

2 234756059 2:234756059-AC C A HJURP 0 2

2 238622911 2:238622911-GA A G LRRFIP1 0 4

2 239155077 2:239155077-GA A G PER2 14 26

2 239253197 2:239253197-CT A G TRAF3IP1 1 2

2 242047665 2:242047665-GA A G PASK 0 1

2 247563 2:247563-CT A G SH3YL1 1 5

2 27256992 2:27256992-GA A G TMEM214 6 10

2 27324216 2:27324216-CT A G CGREF1 3 6

2 27324647 2:27324647-TC G A CGREF1 5 6

2 27670770 2:27670770-CT A G IFT172 0 1

2 27676351 2:27676351-CT A G IFT172 16 20

2 27800953 2:27800953-A-T A T C2orf16 16 39

2 28824168 2:28824168-GA A G PLB1 15 30

2 29169578 2:29169578-CT A G WDR43 1 5

2 29247233 2:29247233-GA A G TOGARAM2 3 9

2 29294511 2:29294511-TC G A PCARE 13 32

2 29296598 2:29296598-GA A G PCARE 9 22

2 31425056 2:31425056-CT A G CAPN14 3 4

2 3727503 2:3727503-CT A G ALLC 1 11

2 38156661 2:38156661-GA A G RMDN2 14 32

2 39085918 2:39085918-AG G A DHX57 12 22

2 39164676 2:39164676-GA A G ARHGEF33 11 21

2 45832558 2:45832558-GA A G SRBD1 0 2

2 47378410 2:47378410-AG G A STPG4 2 5

2 55863500 2:55863500-CT A G PNPT1 1 1

2 71047637 2:71047637-GA A G CLEC4F 5 20

2 71654445 2:71654445-AG G A ZNF638 4 10

2 73447903 2:73447903-GA A G SMYD5 16 34

2 73676190 2:73676190-GA A G ALMS1 1 0

2 73680604 2:73680604-TC G A ALMS1 0 1

2 74275133 2:74275133-AG G A TET3 5 14

2 74275179 2:74275179-GA A G TET3 13 30

2 74460656 2:74460656-GA A G SLC4A5 11 37

2 74654688 2:74654688-TG C A RTKN 1 1

2 74709847 2:74709847-GA A G CCDC142 8 18

2 74883750 2:74883750-GA A G SEMA4F 0 1

2 74903021 2:74903021-CT A G SEMA4F 1 4

2 75276650 2:75276650-GA A G TACR1 10 14

2 75907408 2:75907408-TC G A GCFC2 11 15

2 80085238 2:80085238-CT A G CTNNA2 5 15

2 84960602 2:84960602-CT A G DNAH6 14 27

2 85097590 2:85097590-CT A G TRABD2A 3 12

2 85571458 2:85571458-CT A G RETSAT 10 10

2 85596931 2:85596931-CA A C ELMOD3 6 12

2 85922101 2:85922101-TC G A GNLY 1 3

2 86709651 2:86709651-CT A G KDM3A 8 22

2 88890089 2:88890089-TC G A EIF2AK3 3 8

2 97270582 2:97270582-AG G A KANSL3 2 0

2 97357566 2:97357566-TC G A FER1L5 9 20

2 97464904 2:97464904-GA A G CNNM4 0 2

2 99013601 2:99013601-GA A G CNGA3 4 7

2 1241713 exm169585 A G SNTG2 1 0

2 1263163 exm169593 A G SNTG2 3 3

2 1271185 exm169597 A G SNTG2 1 4

2 1418188 exm169619 A G TPO 2 3

2 1488512 exm169776 A G TPO 13 26

2 1497621 exm169806 A G TPO 3 6

2 1520672 exm169867 A G TPO 11 17

2 1651993 exm169974 A G PXDN 0 1

2 1670156 exm170100 A G PXDN 9 29

2 1684063 exm170125 A G PXDN 2 2

2 3726136 exm170763 G A ALLC 6 11

2 3727551 exm170776 A G ALLC 4 6

2 3730644 exm170789 G A ALLC 15 26

2 3743399 exm170795 A G ALLC 15 39

2 7023598 exm170981 A G RSAD2 17 36

2 7027135 exm170991 A G RSAD2 0 1

2 7027188 exm170994 G A RSAD2 2 7

2 8873708 exm171156 A G KIDINS220 9 14

2 8873780 exm171163 A G KIDINS220 3 3

2 8928894 exm171250 A G KIDINS220 4 12

2 9000756 exm171321 A G MBOAT2 16 31

2 9004345 exm171334 A T MBOAT2 1 1

2 9630541 exm171674 A G ADAM17 14 30

2 10053341 exm171855 C G TAF1B 14 23

2 10059844 exm171872 A G TAF1B 2 6

2 10186320 exm171990 A G KLF11 5 22

2 10188005 exm172013 A G KLF11 12 26

2 10192524 exm172066 G A KLF11 0 1

2 10729287 exm172309 A G NOL10 14 23

2 10894132 exm172382 A G ATP6V1C2 14 27

2 10914975 exm172402 C G ATP6V1C2 3 4

2 10917840 exm172416 A G ATP6V1C2 4 7

2 10918722 exm172417 A G ATP6V1C2 12 29

2 10924397 exm172438 G A PDIA6 6 7

2 10925095 exm172448 A C PDIA6 11 31

2 11284116 exm172636 A G C2orf50 18 29

2 11312136 exm172674 A G SLC66A3 6 15

2 11317922 exm172685 A G SLC66A3 3 5

2 11317929 exm172686 A G SLC66A3 8 15

2 11706672 exm172858 A G GREB1 0 1

2 11750960 exm173021 G C GREB1 5 13

2 11756801 exm173055 A G GREB1 6 18

2 11767103 exm173140 A G GREB1 0 1

2 11798694 exm173236 A C NTSR2 10 23

2 11800247 exm173255 G A NTSR2 10 20

2 11802105 exm173258 A G NTSR2 3 5

2 11927238 exm173368 A G LPIN1 4 9

2 15319162 exm173539 G C NBAS 18 35

2 15358939 exm173560 C A NBAS 9 27

2 15468351 exm173660 A G NBAS 3 5

2 15470775 exm173673 A G NBAS 3 5

2 15492151 exm173682 G A NBAS 0 1

2 15557664 exm173765 G A NBAS 9 25

2 15613369 exm173806 A G NBAS 0 7

2 15613402 exm173807 G A NBAS 1 4

2 15615971 exm173835 G A NBAS 11 26

2 15679468 exm173875 A G NBAS 2 4

2 17696802 exm174123 A C RAD51AP2 2 3

2 17697168 exm174143 A C RAD51AP2 5 12

2 17697252 exm174148 G A RAD51AP2 10 22

2 17699417 exm174219 A G RAD51AP2 6 12

2 17962376 exm174414 C A GEN1 0 5

2 17962577 exm174425 G A GEN1 10 6

2 17962914 exm174434 G A GEN1 3 19

2 17963123 exm174445 G A GEN1 8 22

2 18112418 exm174489 G A KCNS3 9 14

2 18112637 exm174507 G A KCNS3 7 17

2 18112645 exm174508 A G KCNS3 1 5

2 18112763 exm174515 A G KCNS3 10 16

2 18112840 exm174519 G A KCNS3 17 29

2 18112990 exm174529 A G KCNS3 15 34

2 18758156 exm174649 G C NT5C1B;NT5C1B-RDH14 6 16

2 18768421 exm174711 G C NT5C1B;NT5C1B-RDH14 11 23

2 18768432 exm174712 A G NT5C1B;NT5C1B-RDH14 4 12

2 20137705 exm174881 A G WDR35 15 22

2 20146265 exm174892 C G WDR35 11 24

2 20160322 exm174914 C A WDR35 13 23

2 20200274 exm174982 A C MATN3 7 13

2 20402961 exm175071 A G SDC1 14 29

2 20403741 exm175081 A C SDC1 4 11

2 20840864 exm175314 A G HS1BP3 19 25

2 20974586 exm175412 A G LDAH 1 2

2 21001192 exm175434 G A LDAH 6 14

2 21224624 exm175438 G C APOB 3 5

2 21224853 exm175449 A G APOB 17 26

2 21225491 exm175474 G A APOB 20 36

2 21225528 exm175477 A G APOB 12 25

2 21227517 exm175526 A G APOB 8 24

2 21227979 exm175531 A G APOB 7 23

2 21228339 exm175547 A T APOB 2 3

2 21229803 exm175618 A C APOB 1 1

2 21229873 exm175622 A C APOB 8 12

2 21230514 exm175660 A C APOB 9 30

2 21231431 exm175693 A G APOB 2 2

2 21232498 exm175744 C A APOB 24 37

2 21233972 exm175802 G A APOB 1 2

2 21234630 exm175833 A G APOB 2 4

2 21234674 exm175836 A G APOB 0 1

2 21235365 exm175868 G A APOB 1 11

2 21237422 exm175908 G A APOB 1 11

2 21237450 exm175910 A C APOB 6 14

2 21238368 exm175928 A G APOB 1 3

2 21239387 exm175931 A G APOB 3 9

2 21251367 exm176016 A G APOB 12 34

2 21252534 exm176020 A G APOB 4 9

2 21255268 exm176039 A G APOB 10 12

2 21260024 exm176072 A G APOB 6 13

2 21263886 exm176094 G A APOB 8 16

2 23977086 exm176229 A G ATAD2B 1 3

2 23977145 exm176231 A C ATAD2B 2 2

2 24042727 exm176282 C A ATAD2B 9 10

2 24090746 exm176307 A G ATAD2B 3 9

2 24205852 exm176362 A G UBXN2A 14 37

2 24222712 exm176373 C G UBXN2A 5 8

2 24244603 exm176410 A G MFSD2B 5 13

2 24247104 exm176451 A C MFSD2B 5 8

2 24253941 exm176461 C G WDCP 12 25

2 24260578 exm176473 G A WDCP 5 13

2 24260709 exm176479 C G WDCP 3 9

2 24262071 exm176530 A C WDCP 10 28

2 24302317 exm176586 C A TP53I3 5 12

2 24302463 exm176597 A G TP53I3 11 24

2 24303706 exm176599 A G TP53I3 1 1

2 24303723 exm176600 C G TP53I3 12 27

2 24426581 exm176723 A G ITSN2 2 4

2 24426587 exm176724 G A ITSN2 27 50

2 24431163 exm176752 A G ITSN2 4 5

2 24522811 exm176905 A C ITSN2 2 4

2 24533360 exm176933 A G ITSN2 13 24

2 24974856 exm177081 A G NCOA1 5 11

2 24974958 exm177083 A G NCOA1 1 2

2 25037327 exm177154 G A CENPO 14 34

2 25038614 exm177171 C G CENPO 5 13

2 25059895 exm177286 C G ADCY3 3 3

2 25966104 exm177757 A C ASXL2 7 17

2 25972653 exm177808 A G ASXL2 16 33

2 25972816 exm177815 G A ASXL2 2 6

2 26151896 exm177858 G C KIF3C 5 15

2 26203948 exm177914 G C KIF3C 5 9

2 26437421 exm178079 A G HADHA 3 8

2 26453147 exm178088 A C HADHA 3 10

2 26533808 exm178219 A G ADGRF3 8 17

2 26536414 exm178318 A C ADGRF3 1 6

2 26663266 exm178483 A C DRC1 18 35

2 26667128 exm178488 A G DRC1 20 36

2 26679362 exm178575 A G DRC1 34 57

2 26682949 exm178592 A C OTOF 5 7

2 26688863 exm178683 A G OTOF 13 15

2 26690244 exm178717 A G OTOF 1 1

2 26693567 exm178732 C A OTOF 6 9

2 26695500 exm178746 C A OTOF 1 7

2 26700099 exm178830 A G OTOF 12 33

2 26700309 exm178837 A G OTOF 1 0

2 26703073 exm178885 G A OTOF 1 1

2 26707412 exm178922 A G OTOF 1 3

2 26726645 exm178966 A C OTOF 3 7

2 26739428 exm178982 A G OTOF 5 4

2 26741960 exm178991 A G OTOF 9 20

2 26750769 exm178996 A G OTOF 0 1

2 26798961 exm179040 A G FAM166C 8 13

2 27261377 exm179400 C A TMEM214 5 12

2 27292523 exm179572 A G AGBL5 0 1

2 27305241 exm179635 G A EMILIN1 18 35

2 27308030 exm179718 C G EMILIN1 6 9

2 27320480 exm179783 A G KHK 10 22

2 27322140 exm179789 A G KHK 6 16

2 27322538 exm179806 G A KHK 0 2

2 27356164 exm180014 A G PREB 3 4

2 27424936 exm180177 G A SLC5A6 5 12

2 27457017 exm180467 A G CAD 8 16

2 27682309 exm181484 A G IFT172 1 1

2 27682621 exm181488 G A IFT172 0 2

2 27682667 exm181489 A C IFT172 14 30

2 27686003 exm181520 A T IFT172 0 1

2 27720456 exm181660 G A GCKR 7 16

2 27726476 exm181687 A G GCKR 3 10

2 27729397 exm181704 C A GCKR 1 4

2 27801822 exm181849 A G C2orf16 2 7

2 27803308 exm181917 G A C2orf16 5 11

2 27803514 exm181920 A G C2orf16 16 36

2 27804192 exm181951 A G C2orf16 5 7

2 27805351 exm182019 G A C2orf16 3 21

2 27907920 exm182326 A G SLC4A1AP 4 9

2 28741357 exm182538 G A PLB1 1 2

2 28800989 exm182643 A C PLB1 2 5

2 28805279 exm182656 A G PLB1 8 19

2 28812569 exm182684 A G PLB1 0 1

2 28821560 exm182735 A G PLB1 5 11

2 28825724 exm182765 A T PLB1 2 5

2 28825739 exm182766 A G PLB1 0 5

2 28828760 exm182792 G A PLB1 7 9

2 28849308 exm182825 G A PLB1 3 5

2 28854946 exm182851 A G PLB1 2 6

2 29084160 exm182994 A G TRMT61B 1 5

2 29092825 exm183017 A G TRMT61B 1 3

2 29092920 exm183024 A G TRMT61B 18 32

2 29093143 exm183039 G A TRMT61B 1 8

2 29124889 exm183050 A G WDR43 19 34

2 29135479 exm183065 G C WDR43 15 25

2 29225566 exm183169 A G TOGARAM2 21 35

2 29294091 exm183403 G C PCARE 7 12

2 29295293 exm183472 G A PCARE 4 10

2 29295843 exm183497 A G PCARE 8 20

2 29296169 exm183509 A G PCARE 3 7

2 29416157 exm183674 A C ALK 7 14

2 29448419 exm183771 A G ALK 1 5

2 29543736 exm183872 G A ALK 1 2

2 29606603 exm183877 G C ALK 6 8

2 29917793 exm183900 A G ALK 1 1

2 30957326 exm184040 G A CAPN13 18 44

2 30966255 exm184057 G C CAPN13 4 14

2 30966345 exm184063 A T CAPN13 7 8

2 30966371 exm184065 G C CAPN13 9 19

2 30966387 exm184067 A C CAPN13 8 19

2 30987041 exm184110 G C CAPN13 3 4

2 31178579 exm184220 A G GALNT14 10 17

2 31400632 exm184282 G A CAPN14 0 6

2 31489270 exm184412 A T EHD3 0 3

2 31489431 exm184426 G A EHD3 0 1

2 31560583 exm184445 G A XDH 19 29

2 31562365 exm184452 T A XDH 6 11

2 31572992 exm184516 A G XDH 20 37

2 31593265 exm184570 G A XDH 11 25

2 31595176 exm184580 A G XDH 8 13

2 31598325 exm184606 A G XDH 1 0

2 31609402 exm184655 A G XDH 3 4

2 31621475 exm184679 A G XDH 6 19

2 31758781 exm184718 G C SRD5A2 20 40

2 32460623 exm184949 A G NLRC4 6 9

2 32476301 exm185010 A G NLRC4 7 17

2 32476500 exm185024 A G NLRC4 6 5

2 32476635 exm185025 A G NLRC4 6 9

2 32477509 exm185027 G A NLRC4 2 3

2 33412076 exm185761 A G LTBP1 1 4

2 33413908 exm185787 G A LTBP1 14 37

2 33484669 exm185827 A G LTBP1 17 30

2 33505139 exm185860 A C LTBP1 8 14

2 33572566 exm185922 A G LTBP1 25 42

2 33588519 exm185943 A G LTBP1 2 1

2 33623566 exm185979 A G LTBP1 5 13

2 33752435 exm186026 G A RASGRP3 10 34

2 33764176 exm186031 G A RASGRP3 12 33

2 33783392 exm186054 A G RASGRP3 2 8

2 33810638 exm186113 G A FAM98A 0 1

2 33812318 exm186124 A G FAM98A 9 18

2 36749369 exm186288 A G CRIM1 12 27

2 37085047 exm186581 C A STRN 2 1

2 37105041 exm186597 A G STRN 0 3

2 37235927 exm186737 T A HEATR5B 13 22

2 37241120 exm186742 G A HEATR5B 0 1

2 37255995 exm186767 G A HEATR5B 3 10

2 37293012 exm186845 A G HEATR5B 7 23

2 37306288 exm186877 G A HEATR5B 4 5

2 37319156 exm186898 C A GPATCH11 2 5

2 37319321 exm186901 C A GPATCH11 1 7

2 37395103 exm186975 G A SULT6B1 1 3

2 37406645 exm187006 G A SULT6B1 6 17

2 37406661 exm187008 G C SULT6B1 10 23

2 37430140 exm187049 C A CEBPZ 5 11

2 37455492 exm187138 G A CEBPZ 3 1

2 37455683 exm187146 G A CEBPZ 13 17

2 37455922 exm187159 C G CEBPZ 21 35

2 37458862 exm187184 G A NDUFAF7 5 15

2 37458871 exm187185 A G NDUFAF7 16 23

2 37475421 exm187253 A T NDUFAF7 5 6

2 37475489 exm187262 G A NDUFAF7 1 1

2 37543392 exm187352 G A PRKD3 1 14

2 37543555 exm187360 A G PRKD3 18 32

2 37543567 exm187361 A G PRKD3 1 6

2 37586907 exm187394 G A QPCT 10 34

2 37599963 exm187440 C A QPCT 9 15

2 37873058 exm187446 C A CDC42EP3 1 4

2 37873087 exm187448 G A CDC42EP3 6 14

2 38179110 exm187544 A G RMDN2 6 10

2 38179253 exm187559 A G RMDN2 13 20

2 38218381 exm187598 G A RMDN2 11 21

2 38224596 exm187604 A G RMDN2 22 35

2 38526420 exm187747 A G ATL2 5 6

2 38537579 exm187763 G A ATL2 6 7

2 38908500 exm187879 A G GALM 15 28

2 38917004 exm187887 G A GALM 7 15

2 38960634 exm187908 A G GALM 1 3

2 39050135 exm188038 A T DHX57 13 22

2 39050358 exm188053 A G DHX57 12 17

2 39050386 exm188056 C G DHX57 9 20

2 39088158 exm188138 A G DHX57 25 45

2 39088732 exm188164 G A DHX57 3 3

2 39095336 exm188207 A G DHX57 4 8

2 39095444 exm188217 A G DHX57 1 11

2 39184050 exm188257 G A ARHGEF33 3 10

2 39184060 exm188258 C G ARHGEF33 3 10

2 39241107 exm188345 A G SOS1 1 6

2 40404966 exm188792 A G SLC8A1 12 19

2 40656318 exm188836 A G SLC8A1 10 30

2 42472672 exm188973 A G EML4 3 15

2 42513424 exm189022 A G EML4 0 3

2 42990237 exm189269 C G OXER1 23 38

2 42990476 exm189283 A G OXER1 4 2

2 43547672 exm189559 A G THADA 1 2

2 43625142 exm189572 A G THADA 0 5

2 43798899 exm189669 C G THADA 8 21

2 43801829 exm189694 C G THADA 11 27

2 43804251 exm189715 A G THADA 2 1

2 43903314 exm189798 G A C1GALT1C1L 14 27

2 43922335 exm189830 G C PLEKHH2 1 6

2 43927620 exm189897 G A PLEKHH2 11 26

2 43989525 exm190056 G C PLEKHH2 19 33

2 43991488 exm190063 A G PLEKHH2 10 18

2 44031852 exm190150 A G DYNC2LI1 6 10

2 44041634 exm190172 A G ABCG5 5 7

2 44051205 exm190206 A G ABCG5 0 1

2 44099243 exm190358 G A ABCG8 1 8

2 44099244 exm190359 A G ABCG8 5 6

2 44102441 exm190405 A G ABCG8 8 20

2 44428390 exm190652 A G PPM1B 6 9

2 44428819 exm190666 C G PPM1B 7 12

2 44502964 exm190746 G A SLC3A1 8 18

2 44528256 exm190787 A G SLC3A1 4 5

2 44547566 exm190841 A G SLC3A1 2 5

2 44559717 exm190921 G A PREPL 8 10

2 44559767 exm190923 G A PREPL 8 10

2 45778330 exm191201 A G SRBD1 1 5

2 45780845 exm191211 C G SRBD1 6 20

2 45801854 exm191242 G A SRBD1 14 23

2 45826663 exm191272 A C SRBD1 1 10

2 45829094 exm191292 G A SRBD1 23 30

2 45829107 exm191293 A G SRBD1 1 8

2 46574026 exm191411 A G EPAS1 13 27

2 46597004 exm191448 C A EPAS1 1 4

2 46603773 exm191463 A G EPAS1 1 1

2 46607645 exm191503 A G EPAS1 0 3

2 46607784 exm191515 A G EPAS1 4 4

2 46608828 exm191527 G C EPAS1 3 11

2 46842080 exm191676 G A PIGF 1 4

2 46842119 exm191677 G A PIGF 1 1

2 47177506 exm191825 C G TTC7A 3 7

2 47177664 exm191835 A G TTC7A 3 13

2 47202150 exm191852 A G TTC7A 5 11

2 47233133 exm191895 A G TTC7A 6 13

2 47250568 exm191922 G A TTC7A 11 7

2 47251490 exm191933 A G TTC7A 3 11

2 47357121 exm192020 A G STPG4 0 4

2 47378611 exm192041 G A STPG4 1 0

2 48692644 exm192739 A G PPP1R21 1 3

2 48707080 exm192763 G C PPP1R21 3 5

2 48707122 exm192767 C G PPP1R21 11 14

2 48718209 exm192782 G A PPP1R21 9 23

2 48722897 exm192792 A C PPP1R21 10 25

2 48725795 exm192800 G A PPP1R21 14 22

2 48737227 exm192822 A C PPP1R21 0 1

2 48807968 exm192874 G A STON1;STON1-GTF2A1L 6 20

2 48808125 exm192882 A C STON1;STON1-GTF2A1L 7 13

2 48809369 exm192938 A C STON1;STON1-GTF2A1L 4 6

2 48809450 exm192950 A C STON1;STON1-GTF2A1L 8 9

2 48809682 exm192964 A G STON1;STON1-GTF2A1L 10 17

2 48874060 exm193032 A G GTF2A1L;STON1-GTF2A1L 12 39

2 48896937 exm193044 A G GTF2A1L;STON1-GTF2A1L 1 1

2 48915020 exm193075 A G LHCGR 2 3

2 48915576 exm193093 A G LHCGR 15 33

2 48921438 exm193110 G A LHCGR 13 26

2 48941162 exm193132 A C LHCGR 2 10

2 49247259 exm193252 A C FSHR 1 4

2 49295373 exm193258 C G FSHR 7 17

2 53941684 exm193524 A G ASB3;GPR75-ASB3 7 13

2 53955982 exm193545 A T ASB3;GPR75-ASB3 0 1

2 54081548 exm193765 A G GPR75 19 26

2 54119987 exm193819 G A PSME4 2 8

2 54146292 exm193892 G A PSME4 5 12

2 54164528 exm193960 G A PSME4 23 40

2 54482136 exm194017 A G TSPYL6 9 14

2 54482404 exm194025 C G TSPYL6 5 14

2 54482553 exm194032 A G TSPYL6 1 4

2 54483287 exm194087 G A TSPYL6 10 19

2 54587451 exm194134 G A C2orf73 7 16

2 55252354 exm194675 C G RTN4 2 6

2 55252414 exm194678 C A RTN4 2 6

2 55252692 exm194690 G C RTN4 0 8

2 55253869 exm194734 A C RTN4 5 15

2 55408742 exm194830 A G CLHC1 5 19

2 55436876 exm194859 A C CLHC1 3 12

2 55439916 exm194872 A G CLHC1 1 0

2 55449412 exm194888 A C CLHC1 9 14

2 55463798 exm194924 A G MTIF2 2 2

2 55463915 exm194929 G A MTIF2 1 3

2 55481953 exm195003 G A MTIF2 0 1

2 55489517 exm195007 C A MTIF2 5 11

2 55523143 exm195045 G A CCDC88A 0 5

2 55561354 exm195108 A G CCDC88A 1 8

2 55561585 exm195114 G A CCDC88A 3 1

2 55563939 exm195138 G A CCDC88A 3 4

2 55900032 exm195460 A G PNPT1 9 25

2 56098015 exm195530 A G EFEMP1 0 1

2 56144971 exm195557 A G EFEMP1 2 4

2 56420383 exm195638 A C CCDC85A 4 8

2 56420416 exm195641 A C CCDC85A 2 4

2 61148973 exm196037 G A REL 1 3

2 61258687 exm196160 G A PEX13 0 3

2 61272966 exm196187 G A PEX13 5 7

2 61275771 exm196196 C G PEX13 10 14

2 61324902 exm196271 C G KIAA1841 3 7

2 61343134 exm196291 G A KIAA1841 0 3

2 61415274 exm196375 A G USP34 16 32

2 61415798 exm196412 C A USP34 1 2

2 61483548 exm196550 A T USP34 26 38

2 61523960 exm196625 A G USP34 9 23

2 62449516 exm197079 C G B3GNT2 3 10

2 62449581 exm197083 G A B3GNT2 2 2

2 63086366 exm197176 A T EHBP1 1 1

2 63175957 exm197234 C G EHBP1 13 27

2 63176050 exm197237 A G EHBP1 1 1

2 63220713 exm197268 A G EHBP1 1 16

2 63265937 exm197282 G A EHBP1 10 26

2 63605540 exm197357 C A WDPCP 1 2

2 63631355 exm197375 C A WDPCP 8 18

2 63667005 exm197411 C G WDPCP 13 30

2 64682532 exm197702 A G LGALSL 0 3

2 64685425 exm197717 A T LGALSL 12 25

2 64780376 exm197794 A G AFTPH 11 28

2 64819143 exm197848 C A AFTPH 5 14

2 65296657 exm197987 G A CEP68 16 28

2 65296892 exm198004 A G CEP68 4 11

2 65298927 exm198031 G C CEP68 1 1

2 65299020 exm198038 G A CEP68 0 2

2 65299471 exm198065 G C CEP68 5 22

2 65299552 exm198070 A G CEP68 1 4

2 65540986 exm198176 C G SPRED2 2 8

2 67630835 exm198326 G C ETAA1 20 36

2 67631541 exm198354 G A ETAA1 2 2

2 67637150 exm198394 A G ETAA1 19 37

2 68613809 exm198625 C G PLEK 0 3

2 152370194 exm1986346 A G NEB 5 9

2 68622924 exm198644 A C PLEK 2 5

2 68717350 exm198668 A T APLF 0 1

2 15417152 exm1986907 A G NBAS 1 2

2 15427245 exm1986908 A C NBAS 16 34

2 68772437 exm198728 G A APLF 0 2

2 68794483 exm198733 A C APLF 26 42

2 69015032 exm198845 A G ARHGAP25 1 1

2 69049487 exm198885 G A ARHGAP25 1 0

2 167760318 exm1988991 A G XIRP2 19 30

2 69049787 exm198906 A G ARHGAP25 14 29

2 69049916 exm198911 A G ARHGAP25 15 19

2 69049920 exm198912 G A ARHGAP25 13 29

2 69053318 exm198927 A G ARHGAP25 11 29

2 175338074 exm1990509 A G GPR155 2 15

2 69708033 exm199330 A G AAK1 6 9

2 69746244 exm199371 A T AAK1 3 11

2 197878306 exm1993937 A G ANKRD44 11 22

2 202494495 exm1994809 G A TMEM237 2 2

2 207170490 exm1995672 C G ZDBF2 4 4

2 70187808 exm199582 A G ASPRV1 5 11

2 70187809 exm199583 A G ASPRV1 3 3

2 70188556 exm199622 A G ASPRV1 0 2

2 70387822 exm199688 G C C2orf42 0 1

2 70402809 exm199709 A C C2orf42 9 14

2 70402882 exm199713 C G C2orf42 3 16

2 70406721 exm199719 G A C2orf42 27 39

2 218679712 exm1997982 A G TNS1 11 20

2 218683462 exm1998002 A G TNS1 8 10

2 70502735 exm199838 G A PCYOX1 1 2

2 70502747 exm199839 G A PCYOX1 21 36

2 70680410 exm199936 A G TGFA 6 17

2 70680445 exm199937 A G TGFA 6 17

2 70903881 exm200018 A G ADD2 0 3

2 220348880 exm2000422 A C SPEG 20 31

2 70910844 exm200052 A T ADD2 2 5

2 70917935 exm200060 C G ADD2 2 8

2 71036914 exm200151 A G CLEC4F 6 15

2 71039638 exm200160 A G CLEC4F 7 19

2 71043375 exm200177 C A CLEC4F 8 20

2 71043959 exm200214 G A CLEC4F 5 11

2 228162451 exm2004179 A G COL4A3 10 13

2 71360281 exm200730 A G MPHOSPH10 1 2

2 71366957 exm200779 A C MPHOSPH10 16 34

2 71576163 exm200843 A G ZNF638 5 6

2 71577027 exm200883 G A ZNF638 6 9

2 71577172 exm200891 C A ZNF638 13 17

2 71592723 exm200929 G A ZNF638 8 11

2 71655733 exm201107 G C ZNF638 3 5

2 71709066 exm201129 G C DYSF 5 11

2 71742796 exm201190 C A DYSF 1 5

2 71755521 exm201232 A G DYSF 0 2

2 71762413 exm201240 A G DYSF 1 0

2 71838385 exm201437 A C DYSF 8 20

2 71883359 exm201488 C A DYSF 5 8

2 71892333 exm201520 A T DYSF 1 3

2 71895929 exm201537 A G DYSF 5 22

2 71908183 exm201569 A G DYSF 11 18

2 72359400 exm201593 A G CYP26B1 0 1

2 28796073 exm2020005 A G PLB1 5 12

2 29940455 exm2020439 A G ALK 17 32

2 33505123 exm2021203 G A LTBP1 2 4

2 42522368 exm2022490 G A EML4 0 3

2 73675492 exm202505 A G ALMS1 6 27

2 73675692 exm202515 G C ALMS1 6 16

2 73675963 exm202526 A G ALMS1 2 5

2 73678054 exm202615 A C ALMS1 13 16

2 73678642 exm202636 A G ALMS1 14 34

2 73679956 exm202683 A G ALMS1 34 61

2 74327784 exm2027588 A G TET3 0 3

2 73717445 exm202765 G A ALMS1 2 5

2 73762055 exm202849 G A ALMS1 13 33

2 85589319 exm2028687 A T ELMOD3 0 1

2 73799755 exm202879 A T ALMS1 5 20

2 73799896 exm202887 A G ALMS1 2 3

2 73800217 exm202897 A G ALMS1 2 5

2 73800411 exm202908 A G ALMS1 9 19

2 73827901 exm202929 G A ALMS1 6 13

2 88387517 exm2029327 A G SMYD1 1 1

2 96933660 exm2030481 G C CIAO1 1 1

2 99688332 exm2031413 G C TSGA10 1 1

2 74040841 exm203201 A G C2orf78 5 12

2 74041314 exm203220 C A C2orf78 4 4

2 74042489 exm203231 G A C2orf78 5 11

2 74043074 exm203251 A C C2orf78 2 5

2 74043956 exm203290 A G C2orf78 18 49

2 74074603 exm203322 A C STAMBP 6 12

2 74074637 exm203324 A G STAMBP 17 27

2 74076532 exm203328 A G STAMBP 0 1

2 74274336 exm203495 G C TET3 4 15

2 74589235 exm203951 C G DCTN1 3 12

2 74594230 exm204013 A G DCTN1 6 17

2 74654665 exm204258 A C RTKN 8 15

2 74702529 exm204589 A T CCDC142 0 1

2 74709256 exm204629 G C CCDC142 20 35

2 74709268 exm204630 G A CCDC142 20 34

2 74808875 exm205288 A G M1AP 3 8

2 74867339 exm205321 G C M1AP 4 9

2 74901773 exm205378 A G SEMA4F 4 13

2 74906896 exm205422 A G SEMA4F 1 0

2 75276801 exm205631 A G TACR1 7 13

2 75914986 exm205795 G A GCFC2 1 5

2 75916193 exm205803 A C GCFC2 4 6

2 75917161 exm205811 G C GCFC2 5 7

2 75921502 exm205831 C A GCFC2 9 19

2 79312725 exm205978 A G REG1B 13 38

2 79313544 exm205986 G C REG1B 7 13

2 79313574 exm205989 C G REG1B 7 12

2 80530625 exm206217 G A LRRTM1 0 1

2 80530868 exm206231 A G LRRTM1 8 13

2 80646623 exm206242 G A CTNNA2 10 29

2 84745113 exm206363 A G DNAH6 5 5

2 84804522 exm206432 A C DNAH6 2 5

2 84846930 exm206493 G A DNAH6 2 4

2 84897501 exm206540 G A DNAH6 25 61

2 85097476 exm206707 C A TRABD2A 20 34

2 85536415 exm206819 A G TCF7L1 5 8

2 85536565 exm206832 A G TCF7L1 6 7

2 85553842 exm206846 A G TGOLN2 7 6

2 85554265 exm206869 A G TGOLN2 19 30

2 85555069 exm206898 A G TGOLN2 7 6

2 85576627 exm206986 A G RETSAT 0 1

2 85577285 exm207000 A G RETSAT 0 3

2 85581474 exm207032 A T RETSAT 6 11

2 85866329 exm207578 A C USP39 2 5

2 85921546 exm207681 A G GNLY 2 8

2 85921549 exm207682 A G GNLY 11 34

2 85923114 exm207708 A G GNLY 14 25

2 86292438 exm207984 G C POLR1A 1 3

2 86305397 exm208026 A G POLR1A 6 13

2 86308769 exm208034 G A POLR1A 11 15

2 86350837 exm208132 G A PTCD3 7 12

2 86693992 exm208457 G A KDM3A 4 9

2 87042825 exm208764 G A CD8B 18 32

2 88367478 exm208931 G A SMYD1 0 2

2 88383996 exm208943 A C SMYD1 1 0

2 88472737 exm209061 G A THNSL2 6 10

2 88474166 exm209074 A G THNSL2 11 30

2 88474173 exm209076 C G THNSL2 4 8

2 88474273 exm209080 A C THNSL2 2 6

2 88474872 exm209089 A C THNSL2 1 2

2 88485439 exm209133 A G THNSL2 16 19

2 88485551 exm209139 A G THNSL2 2 6

2 88485632 exm209142 A G THNSL2 6 22

2 88828696 exm209176 A G TEX37 12 29

2 88828961 exm209195 A G TEX37 9 21

2 88857275 exm209198 A T EIF2AK3 1 2

2 88882960 exm209246 A G EIF2AK3 1 3

2 88888321 exm209260 A G EIF2AK3 15 34

2 95766599 exm211672 A G MRPS5 22 42

2 95815233 exm211738 A G ZNF514 0 4

2 95941820 exm211885 A G PROM2 18 33

2 95943162 exm211912 A G PROM2 2 15

2 95954315 exm212016 A G PROM2 12 26

2 95954724 exm212020 A G PROM2 2 14

2 96789930 exm212462 A G ASTL 1 5

2 96795639 exm212474 A C ASTL 11 35

2 96935044 exm212720 G A CIAO1 16 36

2 96962702 exm212892 A G SNRNP200 2 2

2 96992631 exm212956 C G ITPRIPL1 1 4

2 96992974 exm212967 A G ITPRIPL1 3 13

2 96993612 exm212995 G C ITPRIPL1 3 19

2 96993930 exm213010 G C ITPRIPL1 0 1

2 97007457 exm213020 A G NCAPH 25 48

2 97007810 exm213032 G A NCAPH 5 13

2 97031782 exm213099 A G NCAPH 3 6

2 97039069 exm213112 A C NCAPH 1 0

2 97217176 exm213186 G A ARID5A 5 16

2 97271083 exm213257 A G KANSL3 7 16

2 97276865 exm213285 G A KANSL3 3 11

2 97369361 exm213444 A T FER1L5 13 13

2 97462887 exm213557 G A CNNM4 6 20

2 97492578 exm213656 A G CNNM3 8 23

2 97493505 exm213663 A C CNNM3 7 11

2 97494385 exm213686 A G CNNM3 4 2

2 98392393 exm214767 A T TMEM131 9 14

2 98419146 exm214835 G A TMEM131 4 10

2 98427662 exm214853 G A TMEM131 5 18

2 98431707 exm214876 A G TMEM131 3 13

2 98779439 exm214966 A C VWA3B 7 7

2 98797509 exm214968 A G VWA3B 15 34

2 98851206 exm215022 A G VWA3B 10 16

2 98887209 exm215059 A G VWA3B 2 3

2 98996706 exm215142 A G CNGA3 7 6

2 99013260 exm215237 A G CNGA3 14 22

2 99685416 exm215647 A T TSGA10 5 7

2 99985866 exm215947 G A EIF5B 5 13

2 99995486 exm215975 A G EIF5B 1 3

2 99995516 exm215978 A C EIF5B 11 23

2 100019558 exm216051 A C REV1 15 28

2 100020945 exm216056 A G REV1 7 17

2 100046395 exm216130 A G REV1 6 20

2 100081415 exm216198 G A REV1 21 45

2 100199295 exm216245 A G AFF3 0 1

2 100623404 exm216369 G A AFF3 3 11

2 100916221 exm216442 G C LONRF2 3 9

2 101011988 exm216517 A T CHST10 12 26

2 101627925 exm216825 A G TBC1D8 8 34

2 101644566 exm216857 G A TBC1D8 8 21

2 101646143 exm216874 A G TBC1D8 6 11

2 101666919 exm216934 T A TBC1D8 18 26

2 102808560 exm217498 A G IL1RL2 24 54

2 102851588 exm217557 A G IL1RL2 4 3

2 102851729 exm217567 A G IL1RL2 11 25

2 102957204 exm217606 A G IL1RL1 8 15

2 102957217 exm217607 A G IL1RL1 7 5

2 102964490 exm217638 A G IL1RL1 13 35

2 102968159 exm217660 C G IL1RL1 18 30

2 102968266 exm217668 A T IL1RL1 5 10

2 102992407 exm217698 A G IL18R1 13 27

2 102998083 exm217702 A G IL18R1 10 21

2 103068382 exm217811 A T IL18RAP 1 3

2 103068513 exm217816 A G IL18RAP 1 1

2 103090237 exm217823 A G SLC9A4 13 27

2 103090405 exm217832 G C SLC9A4 14 33

2 103121783 exm217892 G A SLC9A4 3 8

2 103141573 exm217935 A C SLC9A4 3 5

2 103141583 exm217936 A G SLC9A4 1 7

2 103335354 exm218091 A G MFSD9 1 3

2 103335540 exm218107 A G MFSD9 13 27

2 105654617 exm218237 G A MRPS9 7 18

2 105915058 exm218413 A G TGFBRAP1 4 12

2 106690498 exm218622 A G ECRG4 13 23

2 106694270 exm218630 G A ECRG4 12 25

2 107446665 exm218790 G C ST6GAL2 2 7

2 107459864 exm218819 A C ST6GAL2 23 37

2 108626811 exm218975 A G SLC5A7 7 20

2 108863758 exm219000 A G SULT1C3 5 18

2 108881733 exm219050 A G SULT1C3 2 5

2 108921033 exm219089 A G SULT1C2 10 15

2 108921036 exm219090 G A SULT1C2 21 47

2 108921889 exm219103 A G SULT1C2 13 26

2 109087563 exm219220 G C GCC2 8 17

2 109088231 exm219238 G A GCC2 5 21

2 109092302 exm219269 G A GCC2 21 43

2 109380358 exm219574 A C RANBP2 23 44

2 109399295 exm219789 A G RANBP2 1 4

2 109403311 exm219801 C G CCDC138 21 45

2 109405332 exm219815 A T CCDC138 1 4

2 110919181 exm220354 A G NPHP1 3 5

2 110920681 exm220361 G A NPHP1 11 18

2 110922206 exm220373 A G NPHP1 19 34

2 110959008 exm220393 C A NPHP1 1 6

2 110959026 exm220394 A C NPHP1 22 29

2 110962532 exm220397 A C NPHP1 0 1

2 111542370 exm220546 A G ACOXL 0 2

2 111556617 exm220569 A G ACOXL 21 31

2 111559231 exm220577 A C ACOXL 1 2

2 111789287 exm220655 A C ACOXL 2 3

2 112686742 exm220937 A G MERTK 8 21

2 112740536 exm220989 A G MERTK 5 7

2 112786025 exm221069 A G MERTK 2 11

2 112786167 exm221078 A G MERTK 1 0

2 112786292 exm221084 A G MERTK 11 22

2 113082564 exm221356 A C ZC3H6 9 14

2 113089799 exm221389 C A ZC3H6 4 18

2 113513803 exm221785 G C CKAP2L 0 1

2 113670627 exm221930 A G IL37 20 36

2 113764238 exm221995 G A IL36A 7 17

2 113764243 exm221997 A C IL36A 0 3

2 113765467 exm222001 G A IL36A 1 1

2 113765587 exm222006 T A IL36A 7 9

2 113780274 exm222010 G A IL36B 5 11

2 113783803 exm222021 A G IL36B 1 2

2 113819754 exm222065 A G IL36RN 9 21

2 113940172 exm222205 A G PSD4 1 0

2 113940476 exm222225 A G PSD4 1 9

2 113940916 exm222255 G A PSD4 1 9

2 113941048 exm222262 A G PSD4 23 47

2 113943688 exm222296 A G PSD4 1 1

2 113950118 exm222309 A G PSD4 8 30

2 116534862 exm222801 G A DPP10 1 6

2 118575152 exm222871 A T DDX18 16 23

2 118582279 exm222904 G C DDX18 7 16

2 119699959 exm223152 A G MARCO 4 15

2 119727771 exm223170 G A MARCO 2 4

2 119739077 exm223209 A G MARCO 13 14

2 120003085 exm223305 G A STEAP3 7 19

2 120005456 exm223347 A G STEAP3 6 6

2 120005562 exm223356 G A STEAP3 6 12

2 120020868 exm223412 A G STEAP3 8 20

2 120129841 exm223485 G A DBI 1 3

2 120219480 exm223576 A G SCTR 0 2

2 120231050 exm223598 A C SCTR 5 22

2 105897176 exm2236041 A G TGFBRAP1 2 2

2 120362518 exm223639 C A CFAP221 8 5

2 120388231 exm223681 A G CFAP221 3 4

2 120397363 exm223701 G A CFAP221 8 11

2 120413972 exm223718 A G CFAP221 0 7

2 120438521 exm223736 A G TMEM177 6 5

2 120438671 exm223742 G A TMEM177 13 25

2 120439282 exm223759 A G TMEM177 0 1

2 120658328 exm223791 G A PTPN4 11 33

2 120692506 exm223811 A G PTPN4 16 27

2 120903841 exm223942 G A EPB41L5 0 1

2 120925492 exm223960 G A EPB41L5 11 33

2 120932430 exm223967 C A EPB41L5 1 2

2 121732611 exm224147 A G GLI2 18 35

2 121748153 exm224342 G A GLI2 9 35

2 125192238 exm224709 A G CNTNAP5 2 9

2 125204383 exm224713 A G CNTNAP5 0 2

2 125281910 exm224735 A G CNTNAP5 25 28

2 125547599 exm224802 A G CNTNAP5 12 22

2 125660609 exm224823 A G CNTNAP5 2 3

2 128263222 exm225373 G A IWS1 15 25

2 128281281 exm225375 A G IWS1 15 36

2 128351163 exm225511 A G MYO7B 5 8

2 128367144 exm225544 A G MYO7B 33 42

2 128370070 exm225562 G A MYO7B 10 10

2 128370111 exm225563 A G MYO7B 2 1

2 128379611 exm225576 A G MYO7B 18 32

2 128387373 exm225657 A G MYO7B 9 16

2 128388902 exm225673 A G MYO7B 11 24

2 128477878 exm226078 A G WDR33 1 1

2 128522189 exm226109 G A WDR33 2 10

2 131103803 exm227355 A G IMP4 1 1

2 38178864 exm2273781 A C RMDN2 2 2

2 167266219 exm2273834 A G SCN7A 3 3

2 189863424 exm2275622 A C COL3A1 15 29

2 131486965 exm227582 A G GPR148 6 11

2 131487166 exm227595 C G GPR148 9 21

2 131487719 exm227636 A G GPR148 13 22

2 220342799 exm2277215 A G SPEG 3 7

2 71047602 exm2277238 A G CLEC4F 5 7

2 133174742 exm228663 A T GPR39 1 5

2 133402718 exm228711 C A GPR39 0 1

2 133483265 exm228767 G A NCKAP5 9 27

2 133538668 exm228796 T A NCKAP5 20 34

2 133539637 exm228808 G C NCKAP5 2 5

2 133539660 exm228809 A G NCKAP5 0 1

2 133540714 exm228860 A G NCKAP5 1 1

2 133541206 exm228882 G C NCKAP5 6 11

2 133541262 exm228885 A G NCKAP5 0 3

2 133542004 exm228918 G A NCKAP5 8 19

2 133542010 exm228919 G A NCKAP5 11 13

2 133542105 exm228923 C G NCKAP5 10 27

2 133542166 exm228925 A G NCKAP5 0 3

2 133543200 exm228976 A G NCKAP5 0 1

2 133547732 exm228982 A C NCKAP5 0 1

2 133887676 exm229016 G A NCKAP5 11 24

2 135102556 exm229061 G A MGAT5 2 4

2 135119909 exm229068 G A MGAT5 3 2

2 135738757 exm229295 A G MAP3K19 15 31

2 135743594 exm229327 G A MAP3K19 14 31

2 135743805 exm229332 T A MAP3K19 1 2

2 135744139 exm229339 G A MAP3K19 11 21

2 135744271 exm229346 C A MAP3K19 3 7

2 135745034 exm229370 G A MAP3K19 14 31

2 135848601 exm229458 A T RAB3GAP1 3 9

2 135887597 exm229490 A G RAB3GAP1 1 1

2 135888281 exm229499 A G RAB3GAP1 0 3

2 135920164 exm229541 A G RAB3GAP1 3 6

2 135922233 exm229554 A C RAB3GAP1 16 34

2 135988200 exm229609 A C ZRANB3 6 6

2 135988235 exm229612 A G ZRANB3 9 20

2 135988236 exm229613 A G ZRANB3 9 14

2 136033279 exm229643 A G ZRANB3 17 38

2 136399355 exm229726 G A R3HDM1 8 17

2 136480112 exm229796 A G R3HDM1 11 22

2 136558293 exm229916 A G LCT 0 3

2 136570168 exm230029 A G LCT 1 1

2 136668732 exm230224 A G DARS 3 2

2 136673814 exm230234 A G DARS 14 21

2 137852550 exm230368 A G THSD7B 0 1

2 137928320 exm230386 A G THSD7B 6 12

2 137928506 exm230393 A G THSD7B 15 25

2 141081479 exm230705 A G LRP1B 5 10

2 141128758 exm230765 G C LRP1B 14 14

2 141200082 exm230791 A G LRP1B 6 18

2 141299501 exm230952 A C LRP1B 6 12

2 141986901 exm231239 G A LRP1B 8 17

2 142012161 exm231245 A G LRP1B 19 42

2 144704655 exm231418 A C GTDC1 2 2

2 144704689 exm231420 A G GTDC1 13 26

2 144764858 exm231445 G A GTDC1 19 26

2 144764927 exm231451 A C GTDC1 6 12

2 144899457 exm231462 A T GTDC1 6 13

2 144899591 exm231472 A C GTDC1 3 20

2 148730367 exm231665 C G ORC4 33 67

2 149227542 exm231735 A G MBD5 17 31

2 149247796 exm231812 A G MBD5 4 12

2 150017286 exm231963 A G LYPD6B 10 18

2 152107491 exm232110 G A RBM43 2 1

2 152276777 exm232286 A G RIF1 13 38

2 152318818 exm232414 G A RIF1 3 13

2 152320704 exm232481 A G RIF1 6 7

2 152321679 exm232531 G A RIF1 1 5

2 152322139 exm232555 C A RIF1 2 1

2 152346894 exm232616 G A NEB 3 6

2 152363455 exm232691 A G NEB 7 16

2 152363502 exm232695 G A NEB 3 11

2 152385786 exm232754 A T NEB 7 15

2 152394695 exm232779 C G NEB 16 26

2 152417201 exm232874 A G NEB 2 8

2 152419274 exm232896 A G NEB 2 5

2 152420160 exm232902 A G NEB 20 31

2 152448567 exm232986 G A NEB 13 31

2 152476093 exm233056 A G NEB 10 27

2 152496541 exm233139 A G NEB 4 16

2 152499143 exm233162 A G NEB 5 9

2 152499752 exm233170 A G NEB 8 12

2 152512884 exm233245 G A NEB 3 7

2 152520078 exm233267 G A NEB 0 4

2 152527577 exm233323 A G NEB 22 38

2 152528984 exm233340 A G NEB 1 6

2 152529193 exm233348 G A NEB 2 4

2 152536279 exm233384 C A NEB 2 3

2 152536306 exm233387 G A NEB 2 4

2 152537279 exm233396 G C NEB 3 11

2 152544153 exm233423 G A NEB 9 20

2 152584282 exm233511 A G NEB 5 8

2 152989998 exm233640 A G STAM2 3 14

2 153004633 exm233657 A C STAM2 5 6

2 157427763 exm234206 G C GPD2 14 27

2 157435476 exm234212 A G GPD2 7 22

2 159028765 exm234611 C A CCDC148 10 26

2 159033160 exm234618 A G CCDC148 0 1

2 159035416 exm234621 A G CCDC148 15 36

2 159166142 exm234644 A G CCDC148 2 13

2 159215032 exm234679 A G CCDC148 2 0

2 159672322 exm234904 A G DAPL1 2 6

2 160005754 exm234939 G A TANC1 2 0

2 160028693 exm234993 C A TANC1 1 5

2 160035346 exm235017 A G TANC1 13 34

2 160050798 exm235050 G A TANC1 9 25

2 160076227 exm235104 G A TANC1 6 12

2 160082256 exm235124 G A TANC1 0 1

2 160182374 exm235309 G A BAZ2B 0 1

2 160205691 exm235338 G C BAZ2B 7 8

2 160242917 exm235394 A C BAZ2B 4 7

2 160287443 exm235438 C A BAZ2B 16 30

2 160604372 exm235561 G A MARCHF7 13 20

2 160604379 exm235562 A G MARCHF7 2 8

2 160604768 exm235583 A G MARCHF7 0 3

2 160663568 exm235656 G A LY75;LY75-CD302 10 19

2 160667124 exm235671 A G LY75;LY75-CD302 3 9

2 160673519 exm235684 A G LY75;LY75-CD302 1 0

2 160676431 exm235693 G A LY75;LY75-CD302 12 32

2 160735802 exm235834 A G LY75;LY75-CD302 11 24

2 160798408 exm235915 A G PLA2R1 1 2

2 160832643 exm235998 C A PLA2R1 1 1

2 160876769 exm236069 A G PLA2R1 6 12

2 160879259 exm236072 A G PLA2R1 7 13

2 160885465 exm236096 A G PLA2R1 1 3

2 160889598 exm236102 G A PLA2R1 11 26

2 160898643 exm236111 G A PLA2R1 23 32

2 162830765 exm236561 C G SLC4A10 16 22

2 163130397 exm236872 A G IFIH1 15 29

2 163134757 exm236920 G A IFIH1 9 21

2 163139085 exm236950 T A IFIH1 2 9

2 163215601 exm237032 G A GCA 15 35

2 163374564 exm237145 A G KCNH7 10 14

2 164466648 exm237177 G A FIGN 7 27

2 165353983 exm237276 A T GRB14 3 3

2 165404220 exm237317 G A GRB14 5 14

2 165550888 exm237355 C A COBLL1 1 4

2 165551404 exm237372 A G COBLL1 15 35

2 165551614 exm237382 A G COBLL1 12 30

2 165578602 exm237453 A G COBLL1 1 1

2 165578629 exm237456 A G COBLL1 18 23

2 165755170 exm237508 A C SLC38A11 0 2

2 165768180 exm237522 C A SLC38A11 2 3

2 165946838 exm237564 G A SCN3A 2 7

2 166003301 exm237695 A G SCN3A 1 1

2 166032861 exm237744 A G SCN3A 3 5

2 166536237 exm237974 A G CSRNP3 18 44

2 166616018 exm238018 A G GALNT3 13 23

2 166732698 exm238074 G C TTC21B 3 7

2 166758389 exm238134 A G TTC21B 12 27

2 166769046 exm238150 G A TTC21B 4 3

2 166773990 exm238192 C A TTC21B 3 11

2 166799770 exm238246 A G TTC21B 12 22

2 166854631 exm238330 G A SCN1A 14 27

2 166930064 exm238470 A G SCN1A 7 14

2 167055358 exm238475 C G SCN9A 5 16

2 167099133 exm238539 C A SCN9A 14 21

2 167108344 exm238544 A G SCN9A 2 8

2 167129209 exm238564 C G SCN9A 3 10

2 167129240 exm238567 A G SCN9A 6 10

2 167134745 exm238591 G A SCN9A 1 7

2 167141109 exm238622 A C SCN9A 3 4

2 167145152 exm238652 A G SCN9A 10 14

2 167262238 exm238700 A G SCN7A 1 12

2 167266390 exm238744 G A SCN7A 3 5

2 167288943 exm238786 T A SCN7A 9 8

2 167288989 exm238789 G A SCN7A 5 12

2 167289204 exm238796 A G SCN7A 1 1

2 167298015 exm238803 A G SCN7A 3 2

2 167298034 exm238804 C A SCN7A 1 3

2 167301486 exm238829 A C SCN7A 2 5

2 167304217 exm238834 A C SCN7A 7 11

2 168096375 exm238924 G A XIRP2 4 5

2 168099500 exm238951 A G XIRP2 16 40

2 168099557 exm238953 G C XIRP2 1 0

2 168100724 exm239006 G A XIRP2 4 9

2 168101984 exm239052 A G XIRP2 2 6

2 168103565 exm239102 A C XIRP2 8 32

2 168105449 exm239171 A G XIRP2 3 10

2 168106796 exm239229 G A XIRP2 6 9

2 168106979 exm239236 G A XIRP2 6 9

2 168107009 exm239238 G C XIRP2 4 10

2 168108245 exm239291 A G XIRP2 4 9

2 169312974 exm239481 A G CERS6 36 45

2 169417757 exm239494 A G CERS6 3 9

2 169764338 exm239687 G A G6PC2 2 6

2 169764491 exm239698 G A G6PC2 0 6

2 169783736 exm239722 G A ABCB11 1 6

2 169783772 exm239725 G A ABCB11 10 24

2 169792786 exm239758 A G ABCB11 0 3

2 169820793 exm239778 G A ABCB11 4 4

2 169820807 exm239780 A G ABCB11 2 3

2 169828359 exm239799 A C ABCB11 2 8

2 169869788 exm239856 A G ABCB11 2 8

2 169870836 exm239865 A G ABCB11 2 3

2 169938144 exm239879 A G DHRS9 1 3

2 169938367 exm239888 G C DHRS9 1 4

2 169985220 exm239928 A T LRP2 17 31

2 170010978 exm239992 G A LRP2 9 20

2 170022537 exm240020 A G LRP2 20 40

2 170029657 exm240046 A G LRP2 12 16

2 170038097 exm240087 A G LRP2 5 18

2 170042381 exm240112 A G LRP2 10 20

2 170042445 exm240113 A G LRP2 17 36

2 170059298 exm240179 C G LRP2 0 1

2 170060603 exm240196 G A LRP2 1 1

2 170062683 exm240219 G A LRP2 14 31

2 170062987 exm240226 G A LRP2 3 6

2 170063292 exm240237 A G LRP2 4 4

2 170063757 exm240261 C A LRP2 9 27

2 170068582 exm240274 A G LRP2 12 27

2 170072852 exm240302 G C LRP2 10 24

2 170103283 exm240428 G A LRP2 10 20

2 170103305 exm240431 G A LRP2 10 20

2 170113670 exm240454 G C LRP2 14 32

2 170115592 exm240462 A G LRP2 22 39

2 170136101 exm240513 A T LRP2 12 17

2 170137012 exm240519 G A LRP2 2 5

2 170366485 exm240647 G A KLHL41 4 7

2 170367151 exm240675 C A KLHL41 3 8

2 170367201 exm240677 A G KLHL41 6 10

2 170371159 exm240686 A C KLHL41 4 12

2 170401248 exm240752 A T FASTKD1 13 32

2 170402937 exm240770 G C FASTKD1 6 14

2 170411698 exm240788 G C FASTKD1 5 3

2 170428379 exm240824 A G FASTKD1 6 10

2 170897489 exm241255 A G UBR3 16 36

2 171191899 exm241326 A G MYO3B 9 25

2 171238608 exm241336 C G MYO3B 13 26

2 171256771 exm241373 A G MYO3B 14 29

2 171358311 exm241439 A T MYO3B 10 22

2 171371514 exm241450 A G MYO3B 14 20

2 171376005 exm241459 A G MYO3B 0 14

2 171508618 exm241482 A G MYO3B 17 34

2 172379099 exm241866 A G CYBRD1 0 4

2 172411061 exm241892 A G CYBRD1 14 25

2 172650165 exm241975 A G SLC25A12 5 3

2 172650198 exm241978 A G SLC25A12 1 0

2 172823408 exm242054 C G HAT1 0 3

2 172841111 exm242056 G A HAT1 12 24

2 172841176 exm242059 A G HAT1 13 18

2 173349882 exm242257 G A ITGA6 17 37

2 173355989 exm242302 A G ITGA6 0 1

2 173356028 exm242307 A G ITGA6 3 6

2 173787027 exm242426 C A RAPGEF4 21 34

2 174224215 exm242692 G C CDCA7 4 7

2 174230229 exm242724 A G CDCA7 1 0

2 175243669 exm242984 A G CIR1 2 4

2 175265782 exm243030 C A SCRN3 2 17

2 175326204 exm243126 A G GPR155 3 9

2 175337871 exm243158 A G GPR155 0 1

2 175613411 exm243272 G A CHRNA1 5 7

2 175613486 exm243275 G A CHRNA1 1 4

2 176981692 exm243718 G C HOXD10 3 12

2 176982167 exm243740 G C HOXD10 8 19

2 177033955 exm243908 A G HOXD3 10 27

2 177033966 exm243911 A G HOXD3 7 11

2 177191571 exm244009 G A MTX2 12 23

2 177191606 exm244010 G A MTX2 13 34

2 178096062 exm244098 A C NFE2L2 2 5

2 178096406 exm244114 A G NFE2L2 12 17

2 178096675 exm244119 G A NFE2L2 10 10

2 178097185 exm244123 C A NFE2L2 16 30

2 178415889 exm244223 C G TTC30B 14 28

2 178416285 exm244240 A T TTC30B 2 6

2 178481449 exm244316 G A TTC30A 1 2

2 178565913 exm244463 G A PDE11A 18 42

2 178937010 exm244572 C G PDE11A 0 1

2 179170987 exm244634 G A OSBPL6 0 1

2 179188937 exm244637 G A OSBPL6 4 15

2 179393898 exm245238 A T TTN 25 38

2 179396977 exm245337 A G TTN 11 32

2 179397979 exm245386 A G TTN 18 33

2 179399239 exm245435 A G TTN 20 38

2 179404187 exm245559 A G TTN 2 1

2 179404628 exm245577 A T TTN 21 47

2 179407583 exm245626 A G TTN 6 12

2 179408940 exm245661 A G TTN 0 4

2 179411969 exm245745 A G TTN 5 13

2 179413138 exm245790 A G TTN 0 4

2 179413816 exm245815 G A TTN 20 38

2 179414162 exm245829 G A TTN 0 1

2 179414512 exm245841 G A TTN 3 21

2 179417245 exm245901 T A TTN 0 4

2 179419846 exm245979 G C TTN 2 10

2 179421584 exm245981 A G TTN 5 18

2 179421853 exm245988 A G TTN 1 7

2 179422944 exm246019 C A TTN 4 10

2 179427560 exm246131 A C TTN 5 23

2 179429320 exm246193 G A TTN 13 36

2 179431247 exm246270 G A TTN 7 21

2 179435968 exm246444 A G TTN 10 23

2 179438077 exm246510 A G TTN 9 16

2 179438722 exm246528 A G TTN 7 16

2 179439907 exm246575 C A TTN 11 34

2 179447754 exm246756 A G TTN 2 4

2 179449186 exm246795 A G TTN 6 13

2 179449470 exm246804 A G TTN 0 1

2 179453429 exm246867 A G TTN 1 3

2 179455130 exm246931 G A TTN 4 9

2 179457531 exm247006 A G TTN 9 28

2 179460398 exm247080 A G TTN 8 32

2 179467050 exm247192 A G TTN 4 10

2 179468729 exm247208 A G TTN 1 0

2 179477267 exm247370 C A TTN 14 27

2 179480377 exm247423 G A TTN 1 4

2 179483397 exm247483 A G TTN 12 31

2 179484351 exm247487 A C TTN 10 32

2 179485946 exm247528 A G TTN 4 22

2 179498042 exm247616 G A TTN 11 21

2 179515483 exm247712 C G TTN 6 13

2 179535861 exm247824 C A TTN 12 30

2 179544983 exm247896 C G TTN 1 0

2 179547465 exm247909 A G TTN 4 11

2 179548782 exm247922 A G TTN 25 30

2 179548789 exm247923 C G TTN 9 28

2 179549438 exm247936 G C TTN 9 19

2 179553415 exm247957 A G TTN 4 15

2 179554579 exm247975 A G TTN 1 3

2 179578713 exm248170 G A TTN 16 26

2 179585257 exm248319 G C TTN 7 13

2 179589241 exm248406 A G TTN 3 1

2 179590301 exm248414 G A TTN 11 23

2 179593702 exm248474 A C TTN 6 14

2 179593804 exm248480 G A TTN 3 15

2 179594027 exm248487 A G TTN 7 20

2 179594224 exm248497 G C TTN 11 26

2 179594955 exm248519 A G TTN 0 3

2 179598160 exm248610 A G TTN 8 17

2 179598170 exm248611 A G TTN 18 23

2 179605559 exm248736 A T TTN 0 2

2 179612383 exm248866 A G TTN 2 13

2 179613264 exm248902 A G TTN 2 3

2 179614082 exm248945 A C TTN 6 13

2 179615873 exm249009 A T TTN 3 9

2 179616716 exm249054 A G TTN 13 24

2 179621349 exm249074 C A TTN 18 20

2 179633625 exm249179 A G TTN 12 23

2 179634936 exm249199 A G TTN 0 1

2 179634961 exm249200 A C TTN 4 1

2 179642019 exm249322 A G TTN 10 18

2 179643649 exm249355 G A TTN 3 2

2 179658175 exm249481 A G TTN 1 0

2 179702437 exm249621 G A CCDC141 23 35

2 179718336 exm249641 A G CCDC141 7 15

2 179730517 exm249661 A G CCDC141 7 10

2 179979874 exm249750 G A SESTD1 0 1

2 180014052 exm249793 A G SESTD1 4 12

2 182542978 exm250285 A G NEUROD1 2 6

2 182542998 exm250286 A C NEUROD1 9 20

2 182780484 exm250385 A G ITPRID2 0 2

2 182780637 exm250394 A G ITPRID2 1 8

2 182780874 exm250405 A G ITPRID2 33 59

2 182783334 exm250416 G A ITPRID2 22 39

2 182786740 exm250440 A G ITPRID2 2 3

2 183594584 exm250604 G A DNAJC10 1 4

2 185800842 exm250974 A G ZNF804A 1 4

2 185801073 exm250988 G A ZNF804A 20 35

2 185801613 exm251006 G A ZNF804A 13 20

2 185802398 exm251045 G A ZNF804A 18 37

2 185802591 exm251054 G A ZNF804A 2 5

2 185803160 exm251073 T A ZNF804A 20 35

2 185803262 exm251078 A G ZNF804A 8 19

2 185803445 exm251089 A G ZNF804A 9 18

2 186678633 exm251375 A G FSIP2 18 37

2 187506276 exm251487 G A ITGAV 0 1

2 187521051 exm251511 C A ITGAV 7 16

2 187692986 exm251706 A G ZSWIM2 5 9

2 188332602 exm251838 G A TFPI 5 13

2 188343515 exm251847 A C TFPI 3 4

2 189849536 exm251939 A G COL3A1 19 25

2 189860458 exm252000 A G COL3A1 8 7

2 189861933 exm252017 A C COL3A1 24 40

2 189864044 exm252040 G C COL3A1 8 21

2 189929341 exm252279 A G COL5A2 0 2

2 189931144 exm252286 G A COL5A2 12 26

2 189931456 exm252291 A C COL5A2 18 37

2 189940142 exm252304 C A COL5A2 19 37

2 190338959 exm252462 C G WDR75 2 2

2 190338996 exm252464 A G WDR75 3 16

2 190428384 exm252492 A G SLC40A1 5 15

2 190541743 exm252637 A G ANKAR 2 2

2 190561003 exm252669 A T ANKAR 16 33

2 190569908 exm252679 A G ANKAR 2 5

2 190611135 exm252766 G A ANKAR 3 22

2 190656614 exm252841 C G PMS1 1 1

2 190708697 exm252870 A T PMS1 1 2

2 190719584 exm252903 A G PMS1 3 7

2 190719742 exm252908 A G PMS1 1 1

2 190728507 exm252916 G A PMS1 13 29

2 190742119 exm252944 A G PMS1 1 3

2 192711244 exm253732 G C CAVIN2 6 8

2 192711403 exm253738 G C CAVIN2 5 10

2 196602811 exm253888 A G DNAH7 4 9

2 196602849 exm253890 A G DNAH7 5 22

2 196642600 exm253934 A T DNAH7 10 15

2 196642694 exm253947 A G DNAH7 5 23

2 196664076 exm253974 A G DNAH7 1 0

2 196689136 exm254040 C G DNAH7 13 32

2 196709779 exm254048 C A DNAH7 2 3

2 196728983 exm254116 A G DNAH7 11 31

2 196729589 exm254146 A C DNAH7 12 27

2 196729650 exm254149 C A DNAH7 9 22

2 196750887 exm254198 A G DNAH7 5 13

2 196788366 exm254262 A G DNAH7 7 13

2 196791265 exm254276 G A DNAH7 9 14

2 196799394 exm254284 G A DNAH7 37 59

2 196825238 exm254315 A C DNAH7 1 1

2 196825573 exm254330 A G DNAH7 1 0

2 196834679 exm254341 A C DNAH7 15 29

2 196852767 exm254367 A G DNAH7 3 6

2 196852787 exm254368 A G DNAH7 11 43

2 196865441 exm254377 G A DNAH7 19 34

2 196865498 exm254381 G A DNAH7 17 29

2 196865579 exm254385 A C DNAH7 5 23

2 196866433 exm254394 C A DNAH7 0 2

2 196877562 exm254407 G A DNAH7 1 4

2 196892695 exm254424 G A DNAH7 1 2

2 196892746 exm254428 A G DNAH7 11 26

2 196892763 exm254429 G A DNAH7 14 23

2 197183524 exm254606 A G HECW2 0 1

2 197194337 exm254666 G A HECW2 10 24

2 197298051 exm254690 A G HECW2 1 0

2 197521829 exm254721 A C CCDC150 6 10

2 197523583 exm254729 C A CCDC150 1 1

2 197541314 exm254758 A C CCDC150 10 19

2 197541421 exm254763 A G CCDC150 7 20

2 197709298 exm254958 A G PGAP1 0 1

2 197711869 exm254969 A G PGAP1 1 1

2 198436886 exm255437 A G RFTN2 3 2

2 198436926 exm255440 G A RFTN2 0 1

2 198498573 exm255465 C G RFTN2 15 42

2 198498595 exm255467 A G RFTN2 0 2

2 201337629 exm255927 A C SPATS2L 1 1

2 201397724 exm256010 A G SGO2 3 10

2 201434523 exm256030 A G SGO2 17 40

2 201436870 exm256078 A G SGO2 9 20

2 201438086 exm256119 A G SGO2 10 40

2 201438365 exm256131 G A SGO2 14 26

2 201440089 exm256147 G A SGO2 9 11

2 201485943 exm256251 A G AOX1 1 1

2 201499577 exm256275 A G AOX1 1 5

2 201527618 exm256340 A C AOX1 8 13

2 201807434 exm256599 A G ORC2 6 12

2 202122956 exm256838 G A CASP8 2 11

2 202139671 exm256876 A T CASP8 12 28

2 202195204 exm256950 A G FLACC1 3 0

2 202195515 exm256956 C A FLACC1 12 32

2 202207132 exm256960 A C FLACC1 8 13

2 202211395 exm256972 C G FLACC1 4 5

2 202245546 exm257003 A G TRAK2 1 2

2 202252597 exm257040 A G TRAK2 4 6

2 202254210 exm257055 A C TRAK2 3 7

2 202272186 exm257096 A G TRAK2 11 28

2 202352364 exm257157 A G C2CD6 1 1

2 202352433 exm257159 A G C2CD6 9 13

2 202352480 exm257161 A G C2CD6 11 13

2 202466507 exm257281 C G C2CD6 22 48

2 202466535 exm257284 A G C2CD6 13 31

2 202467970 exm257290 G A C2CD6 1 0

2 202483711 exm257304 A G C2CD6 9 23

2 202501551 exm257344 G A TMEM237 9 23

2 202580494 exm257480 A G ALS2 9 15

2 203329541 exm257957 G A BMPR2 19 48

2 203421084 exm258057 A G BMPR2 2 0

2 203964406 exm258325 A G NBEAL1 11 24

2 203996813 exm258363 T A NBEAL1 3 13

2 204000937 exm258378 A G NBEAL1 3 7

2 204073414 exm258483 G A NBEAL1 6 11

2 204305693 exm258695 G C RAPH1 15 32

2 204355977 exm258790 A G RAPH1 1 2

2 204571441 exm258803 A G CD28 2 4

2 204571465 exm258805 A G CD28 6 11

2 204591575 exm258814 A G CD28 5 6

2 205986331 exm258933 A G PARD3B 1 1

2 205986611 exm258943 A G PARD3B 6 11

2 206023465 exm258965 A G PARD3B 9 25

2 206023605 exm258969 G A PARD3B 1 2

2 206165371 exm259002 A G PARD3B 1 2

2 206305134 exm259035 G A PARD3B 8 33

2 206617651 exm259177 A G NRP2 1 0

2 206869373 exm259247 A G INO80D 1 0

2 206872112 exm259281 G A INO80D 5 4

2 207007482 exm259386 T A NDUFS1 2 7

2 207011753 exm259404 G A NDUFS1 4 13

2 207041527 exm259505 A G GPR1 6 18

2 207041824 exm259517 C G GPR1 11 29

2 207169911 exm259547 G A ZDBF2 11 20

2 207170040 exm259553 A G ZDBF2 17 29

2 207171885 exm259610 G C ZDBF2 1 4

2 207173160 exm259644 A G ZDBF2 5 16

2 207516624 exm259892 G A DYTN 5 20

2 207564900 exm259943 C A DYTN 6 16

2 207564915 exm259945 A G DYTN 6 16

2 207569623 exm259955 C A DYTN 8 23

2 207570565 exm259962 A G DYTN 5 5

2 207572186 exm259973 A G DYTN 18 30

2 207619795 exm260021 A G MDH1B 1 1

2 207619837 exm260025 G A MDH1B 4 6

2 207620125 exm260046 A G MDH1B 1 2

2 207804388 exm260154 C A CPO 12 31

2 207820198 exm260161 A G CPO 15 34

2 207823034 exm260167 A G CPO 5 17

2 207823062 exm260170 A G CPO 2 10

2 207825603 exm260180 G C CPO 9 21

2 207827220 exm260191 A G CPO 14 19

2 207833993 exm260208 A C CPO 4 8

2 208841875 exm260467 A G PLEKHM3 3 4

2 208866200 exm260499 A G PLEKHM3 1 5

2 210518057 exm261230 A G MAP2 0 2

2 210559187 exm261300 A G MAP2 11 20

2 210559865 exm261334 A G MAP2 1 1

2 210560646 exm261370 A G MAP2 9 26

2 210894597 exm261712 A G KANSL1L 9 19

2 211018363 exm261770 A G KANSL1L 3 14

2 211302479 exm261920 A C LANCL1 2 10

2 211305413 exm261931 A G LANCL1 17 28

2 211465359 exm262068 T A CPS1 6 12

2 211504733 exm262125 G A CPS1 1 4

2 211539650 exm262165 A G CPS1 16 35

2 214160817 exm262440 A G SPAG16 13 41

2 214354811 exm262486 A G SPAG16 15 45

2 215617178 exm262588 C G BARD1 3 5

2 215645834 exm262639 G A BARD1 3 2

2 215645978 exm262647 G A BARD1 14 30

2 215657077 exm262656 A G BARD1 3 12

2 215848503 exm262822 A G ABCA12 9 19

2 215914462 exm262997 G A ABCA12 1 0

2 216214314 exm263140 G A ATIC 17 25

2 216251580 exm263274 A G FN1 7 13

2 216257690 exm263297 A G FN1 1 3

2 216269235 exm263356 A G FN1 0 1

2 216274441 exm263411 A G FN1 1 3

2 216930191 exm263568 C A PECR 6 11

2 217124100 exm263745 A G MARCHF4 8 14

2 218669225 exm264068 G A TNS1 24 46

2 218682509 exm264112 C A TNS1 0 2

2 218712605 exm264251 A G TNS1 1 3

2 218712802 exm264266 A G TNS1 16 36

2 218713469 exm264311 A G TNS1 4 4

2 218747073 exm264339 C A TNS1 1 0

2 218758200 exm264368 C A TNS1 2 5

2 218937527 exm264401 A G RUFY4 3 3

2 218940243 exm264431 A T RUFY4 18 27

2 219142150 exm264821 G A TMBIM1 0 2

2 219146663 exm264858 A G TMBIM1 0 1

2 219249024 exm265105 G A SLC11A1 1 9

2 219288517 exm265274 A G VIL1 12 34

2 219297562 exm265356 A G VIL1 2 4

2 219483469 exm265526 A G PLCD4 5 9

2 219507434 exm265663 A G ZNF142 4 13

2 219508180 exm265713 A G ZNF142 1 3

2 219508523 exm265734 C A ZNF142 2 5

2 219515103 exm265822 A C ZNF142 10 31

2 219528817 exm265940 A G RNF25 0 2

2 219529005 exm265947 A G RNF25 4 2

2 219544733 exm266060 A G STK36 11 16

2 219549901 exm266072 A G STK36 2 5

2 219564067 exm266215 G A STK36 4 14

2 219603583 exm266279 A G TTLL4 3 10

2 219603790 exm266286 G A TTLL4 4 15

2 219617897 exm266390 C A TTLL4 2 5

2 219617907 exm266392 C A TTLL4 0 1

2 219619099 exm266410 A C TTLL4 3 11

2 219869043 exm266853 C G CFAP65 3 11

2 219869076 exm266856 C A CFAP65 12 15

2 219878616 exm266932 A C CFAP65 10 14

2 219887050 exm266985 A G CFAP65 12 28

2 219888753 exm266998 G C CFAP65 0 1

2 219888865 exm267002 A G CFAP65 2 3

2 219888924 exm267008 G A CFAP65 4 6

2 219888945 exm267011 A G CFAP65 2 1

2 219892626 exm267044 A G CFAP65 12 38

2 219894286 exm267073 A G CFAP65 6 10

2 219897193 exm267132 A G CFAP65 0 3

2 219903664 exm267181 A G CFAP65 1 4

2 220037600 exm267357 A G CNPPD1 15 45

2 220038094 exm267374 A G CNPPD1 8 20

2 220046957 exm267470 G A RETREG2 1 1

2 220072431 exm267515 A C ZFAND2B 13 21

2 220073991 exm267554 A G ZFAND2B 3 6

2 220077134 exm267603 A G ABCB6 1 14

2 220078595 exm267633 A G ABCB6 4 12

2 220079686 exm267663 C G ABCB6 15 44

2 220082906 exm267704 A G ABCB6 1 4

2 220096678 exm267859 C A ANKZF1 0 2

2 220096757 exm267862 A G ANKZF1 18 28

2 220098859 exm267901 C A ANKZF1 2 5

2 220100476 exm267940 A G ANKZF1 7 17

2 220103915 exm268011 A G GLB1L 15 44

2 220104543 exm268024 A G GLB1L 0 5

2 220156220 exm268243 A G PTPRN 3 7

2 220161037 exm268257 A G PTPRN 1 1

2 220161204 exm268261 A G PTPRN 1 4

2 220164968 exm268324 A G PTPRN 2 2

2 220166368 exm268325 A C PTPRN 3 8

2 220166873 exm268334 A G PTPRN 2 10

2 220285266 exm268533 A T DES 7 11

2 220285586 exm268541 A G DES 4 16

2 220285587 exm268542 C A DES 9 10

2 220337041 exm268752 A G SPEG 6 10

2 220338578 exm268774 A G SPEG 7 26

2 220356901 exm269000 A G SPEG 1 1

2 220366273 exm269037 A G GMPPA 7 13

2 220367160 exm269049 G C GMPPA 7 13

2 220420874 exm269482 A G OBSL1 12 34

2 220422071 exm269516 A G OBSL1 1 2

2 220422740 exm269541 A G OBSL1 9 20

2 220424046 exm269574 A G OBSL1 1 4

2 220431774 exm269660 A G OBSL1 8 28

2 220466716 exm269843 A G STK11IP 5 5

2 220496794 exm270051 A G SLC4A3 1 1

2 220499190 exm270093 G A SLC4A3 2 4

2 223085050 exm270360 A G PAX3 3 6

2 223158829 exm270385 G A PAX3 11 21

2 223559222 exm270610 A G MOGAT1 0 1

2 223559945 exm270618 A C MOGAT1 22 46

2 223574560 exm270628 A T MOGAT1 5 18

2 223783881 exm270669 G A ACSL3 11 14

2 223783909 exm270670 G A ACSL3 12 19

2 223789292 exm270689 A G ACSL3 8 21

2 223789309 exm270690 A G ACSL3 8 21

2 223799341 exm270711 C G ACSL3 1 6

2 224630005 exm270828 G A AP1S3 9 36

2 224849649 exm271006 G A SERPINE2 2 3

2 224862903 exm271025 A G SERPINE2 3 4

2 225657713 exm271211 T A DOCK10 2 7

2 225672767 exm271268 A G DOCK10 8 12

2 225698868 exm271284 T A DOCK10 8 15

2 225740784 exm271344 A G DOCK10 7 21

2 225751189 exm271361 A G DOCK10 1 3

2 227660049 exm271476 A G IRS1 15 39

2 227660234 exm271493 A G IRS1 9 26

2 227661003 exm271537 C G IRS1 1 0

2 227663262 exm271629 A C IRS1 3 4

2 227915847 exm271805 A G COL4A4 2 6

2 227920748 exm271824 A G COL4A4 3 3

2 228134637 exm272090 A G COL4A3 1 4

2 228137792 exm272105 A G COL4A3 11 15

2 228172618 exm272210 A G COL4A3 9 25

2 228228462 exm272321 C A TM4SF20 7 9

2 228560632 exm272485 A G SLC19A3 6 6

2 228563874 exm272513 G A SLC19A3 2 3

2 228750073 exm272573 G A DAW1 7 12

2 228754609 exm272582 A G DAW1 8 19

2 228758537 exm272587 A G DAW1 10 29

2 228855879 exm272650 A G SPHKAP 4 2

2 228860349 exm272667 A G SPHKAP 19 38

2 228883043 exm272772 G A SPHKAP 25 51

2 228883801 exm272812 A G SPHKAP 5 13

2 228883976 exm272820 G A SPHKAP 25 51

2 228884530 exm272842 A G SPHKAP 18 31

2 228884623 exm272847 A G SPHKAP 19 27

2 228884650 exm272848 C A SPHKAP 25 51

2 230282859 exm272975 G A DNER 11 13

2 230377595 exm273000 A G DNER 1 3

2 230411730 exm273010 A G DNER 16 33

2 230450646 exm273018 T A DNER 4 7

2 230910614 exm273287 G C SLC16A14 2 8

2 230911292 exm273311 A C SLC16A14 6 10

2 231036860 exm273368 A G SP110 21 46

2 231108447 exm273488 G C SP140 4 14

2 231110649 exm273495 G A SP140 2 5

2 231222543 exm273601 G A SP140L 5 18

2 231226371 exm273620 G A SP140L 1 3

2 231235641 exm273625 A G SP140L 3 12

2 231256879 exm273648 A G SP140L 2 10

2 231264872 exm273662 A G SP140L 21 44

2 231861210 exm274026 A G SPATA3 1 2

2 231973425 exm274126 A G HTR2B 7 13

2 231988352 exm274169 G A HTR2B 1 5

2 231988465 exm274175 C A HTR2B 11 17

2 232087444 exm274247 C A ARMC9 7 23

2 232141438 exm274306 A G ARMC9 9 23

2 232457921 exm274656 A G TEX44 10 18

2 232458321 exm274675 A G TEX44 6 12

2 232458377 exm274677 A C TEX44 10 20

2 233127939 exm274894 A G DIS3L2 2 2

2 233346268 exm275353 G A ECEL1 10 11

2 233349197 exm275415 A G ECEL1 1 3

2 233407701 exm275628 A C CHRNG 12 40

2 233407762 exm275634 A G CHRNG 12 21

2 233675965 exm275916 A G GIGYF2 15 29

2 233757720 exm276099 A G NGEF 10 14

2 234235819 exm276499 A C SAG 6 15

2 234243673 exm276527 G A SAG 2 5

2 234250943 exm276541 A G SAG 0 3

2 234394489 exm276776 G A USP40 2 1

2 234394520 exm276779 A G USP40 3 7

2 234399886 exm276804 A G USP40 0 2

2 234418717 exm276818 G A USP40 4 7

2 234436069 exm276857 A G USP40 13 21

2 234590826 exm277123 A C UGT1A7 10 20

2 234591295 exm277150 A G UGT1A7 3 6

2 234627668 exm277288 A G UGT1A4 6 15

2 234627963 exm277318 A G UGT1A4 2 10

2 234638110 exm277372 G C UGT1A3 5 8

2 234749352 exm277636 A G HJURP 5 15

2 234749783 exm277663 C G HJURP 9 16

2 234749810 exm277664 A G HJURP 5 9

2 234858728 exm277848 A G TRPM8 1 1

2 234873276 exm277895 A G TRPM8 10 11

2 236957873 exm278325 A G AGAP1 0 1

2 237028849 exm278330 A G AGAP1 2 9

2 237032671 exm278361 A G AGAP1 12 19

2 237349715 exm278549 C G IQCA1 3 5

2 237374286 exm278561 A G IQCA1 3 12

2 238247734 exm278798 G C COL6A3 23 52

2 238249710 exm278846 A G COL6A3 7 8

2 238253261 exm278875 A G COL6A3 10 24

2 238261167 exm278933 A G COL6A3 5 9

2 238277218 exm279053 A G COL6A3 5 8

2 238280366 exm279096 A G COL6A3 1 2

2 238280570 exm279116 A G COL6A3 3 6

2 238296323 exm279315 G A COL6A3 3 5

2 238296513 exm279330 A G COL6A3 13 21

2 238419321 exm279413 G A MLPH 26 44

2 238434379 exm279455 A G MLPH 17 33

2 238436131 exm279465 A G MLPH 3 4

2 238455318 exm279505 A G MLPH 1 1

2 238632224 exm279656 A G LRRFIP1 1 3

2 238643972 exm279670 A G LRRFIP1 5 10

2 238664765 exm279703 C A LRRFIP1 0 2

2 238668783 exm279714 G A LRRFIP1 14 33

2 238671853 exm279738 G C LRRFIP1 6 13

2 238672425 exm279769 C G LRRFIP1 14 33

2 238678622 exm279794 A G LRRFIP1 4 5

2 238725768 exm279814 A G RBM44 9 20

2 239098594 exm280490 G A ILKAP 20 40

2 239160264 exm280570 G A PER2 6 17

2 239162038 exm280607 G A PER2 9 18

2 239165577 exm280646 A G PER2 12 20

2 239184399 exm280741 A G PER2 13 22

2 239184431 exm280745 A G PER2 4 2

2 239185804 exm280752 C A PER2 3 17

2 239185817 exm280753 C A PER2 7 6

2 239186363 exm280755 A G PER2 4 15

2 239237388 exm280791 A G TRAF3IP1 3 2

2 239237460 exm280793 G A TRAF3IP1 22 39

2 239237753 exm280804 A G TRAF3IP1 2 5

2 239247029 exm280835 G A TRAF3IP1 1 0

2 240002858 exm280989 A G HDAC4 1 3

2 240061399 exm281109 G A HDAC4 6 12

2 240984559 exm281550 A G OR6B3 4 5

2 240984574 exm281551 A G OR6B3 16 26

2 241448854 exm281925 A G ANKMY1 10 27

2 241459876 exm281952 G A ANKMY1 3 5

2 241463494 exm281982 A G ANKMY1 4 14

2 241463495 exm281983 A G ANKMY1 27 46

2 241468671 exm282058 A G ANKMY1 19 48

2 241468875 exm282068 A G ANKMY1 7 11

2 241492342 exm282077 A G ANKMY1 18 39

2 241570006 exm282466 A G GPR35 14 23

2 241570127 exm282479 A G GPR35 7 9

2 241570226 exm282483 A G GPR35 4 16

2 241815411 exm282971 G A AGXT 1 0

2 241817544 exm282991 A G AGXT 4 5

2 241969887 exm283174 A G SNED1 1 5

2 241987798 exm283221 A G SNED1 2 6

2 242021197 exm283374 A G SNED1 11 19

2 242035594 exm283426 A G MTERF4 5 12

2 242063459 exm283561 A G PASK 7 12

2 242065698 exm283579 A G PASK 0 1

2 242065754 exm283584 A G PASK 8 16

2 242066688 exm283651 A C PASK 3 5

2 242072325 exm283664 G A PASK 0 1

2 242078094 exm283716 A G PASK 9 32

2 242078148 exm283724 A G PASK 4 8

2 242139600 exm283907 A G ANO7 1 4

2 242195731 exm284261 G C HDLBP 1 0

2 242195787 exm284266 A G HDLBP 12 25

2 242312572 exm284337 A G FARP2 34 51

2 242402016 exm284448 A G FARP2 0 1

2 242407673 exm284487 G A FARP2 3 6

2 242407748 exm284494 A G FARP2 1 3

2 242423712 exm284515 A G FARP2 3 7

2 242429405 exm284520 T A FARP2 8 19

2 242429428 exm284523 A G FARP2 7 6

2 242430495 exm284527 C A FARP2 13 27

2 242738488 exm285077 A G GAL3ST2 2 12

2 242742837 exm285108 C G GAL3ST2 3 12

2 103335441 JHU_2.103335440 G A MFSD9 14 25

2 113520129 JHU_2.113520128 A G CKAP2L 28 57

2 11943082 JHU_2.11943081 A G LPIN1 2 3

2 27423913 JHU_2.27423912 A G SLC5A6 20 42

2 31593333 kgp14497001 A G XDH 7 26

2 99012315 newrs147415641 A G CNGA3 5 17

2 173337618 newrs74728869 A G ITGA6 34 45

2 29295602 Variant16772 D I PCARE 2 1

2 18766156 Variant38673 A C NT5C1B;NT5C1B-RDH14 2 1

3 10302160 3:10302160-GA A G TATDN2 1 1

3 107435470 3:107435470-CT A G BBX 8 19

3 107520030 3:107520030-CT A G BBX 7 13

3 108214644 3:108214644-CT A G MYH15 3 6

3 108751579 3:108751579-CT A G MORC1 14 25

3 111664163 3:111664163-GT A C PHLDB2 3 8

3 111775954 3:111775954-GA A G TMPRSS7 0 1

3 112993344 3:112993344-AG G A BOC 0 3

3 113049044 3:113049044-CT A G CFAP44 5 9

3 113077614 3:113077614-CT A G CFAP44 5 16

3 113098217 3:113098217-GA A G CFAP44 4 8

3 113187136 3:113187136-TG C A SPICE1 16 35

3 118866429 3:118866429-AG G A TEX55 1 4

3 119367415 3:119367415-GA A G POPDC2 5 9

3 119469883 3:119469883-AG G A MAATS1 1 3

3 120128417 3:120128417-CT A G FSTL1 0 3

3 120495311 3:120495311-CT A G GTF2E1 5 16

3 120500166 3:120500166-TC G A GTF2E1 8 13

3 121415528 3:121415528-GA A G GOLGB1 14 38

3 122679987 3:122679987-CT A G SEMA5B 8 17

3 124738372 3:124738372-GA A G HEG1 4 7

3 124826777 3:124826777-GA A G SLC12A8 2 3

3 12546693 3:12546693-AG G A TSEN2 17 39

3 126180820 3:126180820-GA A G ZXDC 0 3

3 130287097 3:130287097-GA A G COL6A6 0 8

3 130290132 3:130290132-GA A G COL6A6 1 2

3 130368215 3:130368215-TC G A COL6A6 2 3

3 130368272 3:130368272-GT A C COL6A6 0 2

3 130380718 3:130380718-AG G A COL6A6 2 18

3 13395067 3:13395067-CT A G NUP210 1 2

3 137786493 3:137786493-GA A G DZIP1L 0 2

3 139280082 3:139280082-CT A G NMNAT3 8 9

3 141164795 3:141164795-GA A G ZBTB38 3 5

3 143371167 3:143371167-CT A G SLC9A9 1 1

3 14535325 3:14535325-CT A G GRIP2 7 25

3 14548315 3:14548315-CA A C GRIP2 16 26

3 148559694 3:148559694-CT A G CPB1 6 14

3 148895719 3:148895719-CT A G CP 8 15

3 149498089 3:149498089-GA A G ANKUB1 7 9

3 149498146 3:149498146-CA A C ANKUB1 8 21

3 151046222 3:151046222-TC G A P2RY13 4 7

3 154145317 3:154145317-CT A G GPR149 0 4

3 155222394 3:155222394-CT A G PLCH1 5 3

3 167090650 3:167090650-GT A C ZBBX 12 32

3 167322163 3:167322163-CT A G WDR49 1 2

3 167747022 3:167747022-CT A G GOLIM4 17 34

3 169485941 3:169485941-AG G A ACTRT3 6 20

3 172631512 3:172631512-GA A G SPATA16 24 47

3 174577235 3:174577235-TC G A NAALADL2 1 1

3 183521787 3:183521787-AG G A YEATS2 2 4

3 183750748 3:183750748-TG C A HTR3D 1 4

3 183911415 3:183911415-CT A G ABCF3 0 3

3 184039242 3:184039242-GA A G EIF4G1 5 7

3 184911173 3:184911173-TC G A EHHADH 0 1

3 185332451 3:185332451-AG G A SENP2 0 1

3 186331129 3:186331129-AG G A AHSG 2 4

3 186392973 3:186392973-CA A C HRG 33 62

3 186445096 3:186445096-TC G A KNG1 8 35

3 186760664 3:186760664-CA A C ST6GAL1 11 11

3 186947631 3:186947631-CT A G MASP1 6 27

3 186953749 3:186953749-CA A C MASP1 1 2

3 186970985 3:186970985-CT A G MASP1 20 33

3 187447701 3:187447701-CA A C BCL6 24 37

3 190573484 3:190573484-TC G A GMNC 6 11

3 190578547 3:190578547-GA A G GMNC 2 10

3 193048922 3:193048922-CT A G ATP13A5 0 3

3 193082039 3:193082039-GA A G ATP13A5 0 1

3 194157936 3:194157936-CT A G ATP13A3 1 1

3 195516350 3:195516350-CT A G MUC4 0 3

3 196386965 3:196386965-CT A G NRROS 8 16

3 27183037 3:27183037-CT A G NEK10 14 29

3 27335141 3:27335141-AC C A NEK10 2 6

3 33194262 3:33194262-TC G A SUSD5 4 15

3 36905897 3:36905897-TC G A TRANK1 8 22

3 37089130 3:37089130-AG G A MLH1 0 1

3 38051211 3:38051211-GA A G PLCD1 1 4

3 38592012 3:38592012-CA A C SCN5A 5 5

3 38645379 3:38645379-CA A C SCN5A 3 13

3 38645495 3:38645495-C-T A G SCN5A 0 3

3 39228161 3:39228161-CT A G XIRP1 0 5

3 39432974 3:39432975-ACT-A D I SLC25A38 0 1

3 41954333 3:41954333-CT A G ULK4 0 1

3 43073835 3:43073835-CT A G GASK1A 5 9

3 44296474 3:44296474-TC G A TOPAZ1 7 29

3 44308512 3:44308512-TC G A TOPAZ1 10 17

3 44670732 3:44670732-GA A G ZNF197;ZNF660-ZNF197 5 10

3 44762584 3:44762584-AC C A ZNF502 2 4

3 45130609 3:45130609-GA A G CDCP1 10 24

3 45134811 3:45134811-AG G A CDCP1 1 1

3 46007823 3:46007823-GT A C FYCO1 23 57

3 46245525 3:46245525-TG C A CCR1 12 18

3 46542370 3:46542370-TC G A RTP3 6 10

3 46563113 3:46563113-GT A C LRRC2 2 6

3 47108560 3:47108560-TC G A SETD2 6 7

3 47164921 3:47164921-CT A G SETD2 2 9

3 47165791 3:47165791-GA A G SETD2 3 2

3 48621470 3:48621470-GA A G COL7A1 1 1

3 48732733 3:48732733-CT A G IP6K2 5 11

3 49042515 3:49042515-TG C A P4HTM 5 16

3 49691710 3:49691710-CT A G BSN 0 1

3 49830624 3:49830624-GT A C CDHR4 7 16

3 49847737 3:49847737-CT A G UBA7 10 13

3 49848255 3:49848255-CT A G UBA7 9 28

3 51929202 3:51929202-TC G A IQCF1 2 3

3 52360860 3:52360860-AG G A DNAH1 0 1

3 52429684 3:52429684-AG G A DNAH1 7 9

3 52745831 3:52745831-GA A G NEK4 1 3

3 52853477 3:52853477-AC C A ITIH4 1 9

3 53267183 3:53267183-CT A G TKT 0 4

3 57542485 3:57542485-TG C A PDE12 0 1

3 58410550 3:58410550-GA A G PXK 10 21

3 66432786 3:66432786-AG G A LRIG1 1 0

3 69072335 3:69072335-AC C A TMF1 2 5

3 69435131 3:69435131-CT A G FRMD4B 1 1

3 77607239 3:77607239-CT A G ROBO2 9 14

3 81584403 3:81584403-TC G A GBE1 8 17

3 97660010 3:97660010-AG G A CRYBG3 14 32

3 97868404 3:97868404-AG G A OR5H14 1 0

3 97868747 3:97868747-TC G A OR5H14 6 12

3 98188548 3:98188548-TC G A OR5K1 9 8

3 98189122 3:98189122-GT A C OR5K1 2 2

3 99569010 3:99569010-GA A G FILIP1L 2 1

3 9971546 3:9971546-CT A G IL17RC 5 9

3 100365528 exm2031681 A G ADGRG7 2 13

3 113115376 exm2033645 G C CFAP44 4 6

3 113375764 exm2033793 A G USF3 1 8

3 121415471 exm2034913 A G GOLGB1 0 1

3 126154470 exm2036403 A G CFAP100 0 1

3 12859262 exm2037070 A G CAND2 1 7

3 131068498 exm2037966 A G NEK11 3 7

3 132378559 exm2038231 A C ACAD11 6 16

3 170198555 exm2043728 A G SLC7A14 1 2

3 184090610 exm2045749 G C THPO 0 1

3 186276213 exm2046312 A T TBCCD1 0 1

3 193081095 exm2047086 G A ATP13A5 3 7

3 20164184 exm2048597 G C KAT2B 9 21

3 45748283 exm2052601 G A SACM1L 6 12

3 52255540 exm2057045 A G TLR9 0 1

3 52420212 exm2057334 A C DNAH1 0 8

3 52430775 exm2057390 C A DNAH1 10 16

3 9881921 exm2062073 A G RPUSD3 6 13

3 138187527 exm2275701 A G ESYT3 4 6

3 361493 exm285445 A G CHL1 1 8

3 403395 exm285519 A C CHL1 2 4

3 404982 exm285533 G A CHL1 14 21

3 423904 exm285559 G A CHL1 1 0

3 1189697 exm285668 A G CNTN6 5 13

3 1320121 exm285689 A G CNTN6 8 15

3 1320181 exm285695 C G CNTN6 1 0

3 1425055 exm285824 A G CNTN6 12 35

3 3030052 exm285920 G A CNTN4 7 19

3 3139568 exm286061 G C IL5RA 0 5

3 3144410 exm286085 A C IL5RA 21 29

3 3887508 exm286294 C G LRRN1 21 61

3 3887643 exm286300 A G LRRN1 3 8

3 3888392 exm286333 T A LRRN1 5 9

3 4355347 exm286393 A G SETMAR 6 10

3 8590374 exm287213 A G LMCD1 0 1

3 8590449 exm287218 A G LMCD1 0 1

3 8590576 exm287225 G A LMCD1 11 26

3 8609276 exm287255 A G LMCD1 0 1

3 8667299 exm287280 A G SSUH2 5 6

3 8672586 exm287315 A G SSUH2 7 16

3 8673791 exm287320 A G SSUH2 3 8

3 8944169 exm287474 A C RAD18 0 3

3 8983427 exm287502 G A RAD18 17 31

3 9413122 exm287715 G A THUMPD3 5 13

3 9426209 exm287749 A G THUMPD3 1 4

3 9726918 exm287973 A G MTMR14 2 7

3 9796510 exm288276 G C OGG1 4 16

3 9798740 exm288286 G A OGG1 6 9

3 9798760 exm288288 A G OGG1 23 28

3 9862346 exm288501 A G ARPC4-TTLL3;TTLL3 9 16

3 9868870 exm288529 A G ARPC4-TTLL3;TTLL3 10 15

3 9879810 exm288616 A G RPUSD3 1 1

3 9911717 exm288708 A G CIDEC 5 17

3 9920128 exm288733 A T CIDEC 7 19

3 9948783 exm288825 A T IL17RE 1 1

3 9953248 exm288861 A C IL17RE 5 2

3 9959160 exm288909 G A IL17RC 3 0

3 9959687 exm288931 C G IL17RC 8 13

3 9965576 exm288949 A G IL17RC 1 0

3 9965582 exm288950 A G IL17RC 1 2

3 9974348 exm289025 A G IL17RC 4 5

3 10076416 exm289343 G A FANCD2 3 13

3 10140614 exm289587 A C FANCD2 6 18

3 10219638 exm289687 A G IRAK2 3 7

3 10242097 exm289700 A G IRAK2 1 0

3 10264378 exm289741 A C IRAK2 8 13

3 10264477 exm289747 A C IRAK2 19 40

3 10291231 exm289819 A G TATDN2 5 12

3 10302076 exm289838 G C TATDN2 12 29

3 10312346 exm289874 A C TATDN2 1 2

3 10328453 exm289930 A T GHRL 0 1

3 10331447 exm289939 A G GHRL 1 4

3 10347312 exm289991 A G SEC13 13 32

3 10357018 exm290018 A G SEC13 8 13

3 10953786 exm290254 A T SLC6A11 4 12

3 10976854 exm290303 A G SLC6A11 13 32

3 11300765 exm290409 A G HRH1 6 20

3 11301672 exm290449 A T HRH1 10 22

3 11374505 exm290501 A G ATG7 5 10

3 11389502 exm290518 A G ATG7 0 3

3 11871215 exm290654 G A TAMM41 9 20

3 11871299 exm290659 G A TAMM41 3 7

3 12393125 exm290816 G C PPARG 22 32

3 12421253 exm290822 A T PPARG 0 7

3 12458386 exm290853 G C PPARG 2 14

3 12545039 exm290913 A C TSEN2 22 52

3 12777111 exm291122 A G TMEM40 3 7

3 12780070 exm291142 A G TMEM40 9 24

3 12854873 exm291229 A T CAND2 4 5

3 12858290 exm291283 A G CAND2 12 20

3 12950002 exm291474 A G IQSEC1 11 15

3 12961945 exm291501 G A IQSEC1 9 26

3 12962074 exm291505 A G IQSEC1 13 36

3 13363102 exm291664 A G NUP210 3 14

3 13364910 exm291688 A C NUP210 9 20

3 13371970 exm291725 G A NUP210 11 28

3 13383511 exm291796 A G NUP210 0 3

3 13660487 exm292142 A C FBLN2 3 9

3 13660506 exm292143 G A FBLN2 2 10

3 13660508 exm292144 G A FBLN2 5 7

3 13679190 exm292239 A G FBLN2 2 7

3 14199603 exm292512 A G XPC 17 32

3 14199908 exm292529 A G XPC 17 49

3 14209787 exm292561 A G XPC 1 0

3 14214524 exm292574 A G XPC 0 1

3 14700436 exm292902 A G CCDC174 13 32

3 14703094 exm292909 A G CCDC174 3 6

3 14725878 exm293003 G A C3orf20 1 3

3 14731623 exm293012 C G C3orf20 9 26

3 14861540 exm293160 C G FGD5 11 14

3 14905700 exm293242 A G FGD5 4 7

3 14939082 exm293252 A G FGD5 11 14

3 14939462 exm293257 A G FGD5 4 16

3 14939471 exm293259 A G FGD5 3 5

3 14942551 exm293274 A G FGD5 5 16

3 15119634 exm293502 G A RBSN 5 5

3 15127459 exm293529 A G RBSN 1 1

3 15471471 exm293764 G A EAF1 1 0

3 15478047 exm293791 A G EAF1 1 0

3 15609448 exm293934 A C HACL1 10 21

3 15624419 exm293967 A G HACL1 2 6

3 15686799 exm294095 A G BTD 0 1

3 15718494 exm294114 A G ANKRD28 3 11

3 16268940 exm294313 A G GALNT15 6 11

3 16336503 exm294371 A G OXNAD1 5 6

3 16344229 exm294394 G A OXNAD1 1 1

3 16358552 exm294409 G A RFTN1 15 38

3 16358653 exm294416 C A RFTN1 0 1

3 17202649 exm294664 A T TBC1D5 9 31

3 17349498 exm294714 G A TBC1D5 1 1

3 19384171 exm294837 A C KCNH8 6 6

3 19559510 exm294917 G C KCNH8 1 1

3 19575434 exm294945 A G KCNH8 2 3

3 19961395 exm295038 G A EFHB 19 35

3 19975008 exm295055 A C EFHB 8 18

3 19975194 exm295061 A G EFHB 0 1

3 20181809 exm295176 A G KAT2B 1 1

3 20212227 exm295214 A G SGO1 38 61

3 20212652 exm295223 G A SGO1 6 12

3 20216097 exm295255 G A SGO1 13 29

3 20225429 exm295292 A C SGO1 10 16

3 24003590 exm295467 A G NR1D2 9 20

3 24003959 exm295485 C A NR1D2 1 1

3 25770656 exm295791 G A NGLY1 2 7

3 25770727 exm295795 A G NGLY1 13 25

3 25792697 exm295847 A C NGLY1 18 40

3 27385817 exm296139 A G NEK10 0 1

3 27424710 exm296164 G A SLC4A7 2 5

3 27436597 exm296184 A G SLC4A7 15 38

3 27473124 exm296255 G C SLC4A7 0 3

3 27473139 exm296257 A G SLC4A7 8 15

3 28476661 exm296431 A T ZCWPW2 2 2

3 28566101 exm296464 G A ZCWPW2 12 20

3 30713717 exm296592 A G TGFBR2 0 1

3 32932183 exm297299 A G TRIM71 0 1

3 33059977 exm297421 G A GLB1 7 23

3 33060002 exm297423 A G GLB1 4 4

3 33134756 exm297499 A G TMPPE 4 11

3 33135030 exm297512 G A TMPPE 14 28

3 33166039 exm297610 G A CRTAP 3 4

3 33194596 exm297656 G A SUSD5 0 1

3 33195152 exm297675 C G SUSD5 18 27

3 33195213 exm297679 A G SUSD5 2 2

3 35778697 exm298199 G C ARPP21 1 2

3 35835320 exm298254 A G ARPP21 8 12

3 36545938 exm298317 G A STAC 8 20

3 36570440 exm298334 A G STAC 13 32

3 36897597 exm298680 A C TRANK1 8 22

3 36898973 exm298739 A G TRANK1 0 2

3 36931397 exm298772 A G TRANK1 0 5

3 37070285 exm298902 G C MLH1 0 1

3 37125165 exm298994 A T LRRFIP2 10 24

3 37151052 exm299041 A G LRRFIP2 19 23

3 37152519 exm299051 G A LRRFIP2 8 12

3 37323465 exm299095 A G GOLGA4 10 25

3 37365275 exm299172 T A GOLGA4 7 13

3 37366598 exm299215 A G GOLGA4 1 2

3 37366967 exm299229 G A GOLGA4 14 39

3 37367386 exm299250 G A GOLGA4 21 30

3 37367989 exm299271 G A GOLGA4 0 1

3 37368961 exm299305 G A GOLGA4 2 7

3 37369008 exm299307 A C GOLGA4 2 0

3 37512596 exm299415 A G ITGA9 15 21

3 37514860 exm299421 A G ITGA9 8 14

3 37559040 exm299466 G A ITGA9 16 31

3 37725466 exm299532 G A ITGA9 2 3

3 38049310 exm299807 G A PLCD1 11 26

3 38052725 exm299903 A G PLCD1 18 22

3 38052845 exm299921 A C PLCD1 9 23

3 38058171 exm299948 A G PLCD1 7 15

3 38103776 exm300021 A G DLEC1 0 1

3 38125685 exm300063 A G DLEC1 5 8

3 38136401 exm300123 G A DLEC1 29 58

3 38151053 exm300190 A G DLEC1 10 16

3 38158031 exm300252 A C DLEC1 7 21

3 38163803 exm300323 A G DLEC1 1 1

3 38167095 exm300361 G A ACAA1 20 26

3 38170838 exm300391 G A ACAA1 0 3

3 38316168 exm300546 A C SLC22A13 1 2

3 38317414 exm300578 G A SLC22A13 3 3

3 38317417 exm300579 A G SLC22A13 14 25

3 38317882 exm300599 A G SLC22A13 4 11

3 38355325 exm300717 A G SLC22A14 10 18

3 38357987 exm300748 A G SLC22A14 17 19

3 38390056 exm300760 G C XYLB 3 0

3 38407198 exm300791 C A XYLB 4 22

3 38420828 exm300840 A G XYLB 2 7

3 38436996 exm300842 G A XYLB 1 2

3 38442402 exm300854 A G XYLB 17 34

3 38592152 exm301022 A G SCN5A 0 3

3 38592356 exm301030 G A SCN5A 5 16

3 38640465 exm301149 A G SCN5A 9 22

3 38645241 exm301154 A G SCN5A 7 31

3 38651303 exm301207 A C SCN5A 0 1

3 38739054 exm301255 A G SCN10A 21 44

3 38739622 exm301288 A G SCN10A 7 27

3 38743435 exm301325 A G SCN10A 2 3

3 38763895 exm301392 A C SCN10A 2 2

3 38791663 exm301467 A G SCN10A 9 21

3 38797287 exm301487 A G SCN10A 8 18

3 38812827 exm301546 C A SCN10A 0 1

3 38888281 exm301579 A T SCN11A 4 8

3 38936089 exm301685 A G SCN11A 3 6

3 38936310 exm301694 G A SCN11A 3 19

3 38938526 exm301715 A G SCN11A 16 23

3 38950678 exm301752 A G SCN11A 4 15

3 38968310 exm301780 A T SCN11A 4 10

3 38988312 exm301792 G C SCN11A 11 8

3 39150636 exm301988 A C TTC21A 13 36

3 39156081 exm302003 A G TTC21A 1 3

3 39170782 exm302075 A C TTC21A 1 0

3 39170800 exm302077 A G TTC21A 2 3

3 39170801 exm302078 A G TTC21A 21 41

3 39174543 exm302099 A C TTC21A 3 13

3 39178437 exm302122 A C TTC21A 2 9

3 39178453 exm302124 C G TTC21A 5 14

3 39225835 exm302287 G A XIRP1 25 41

3 39226079 exm302306 A G XIRP1 3 14

3 39226916 exm302346 G A XIRP1 12 22

3 39228311 exm302420 C G XIRP1 6 5

3 39229879 exm302519 A G XIRP1 2 6

3 39229978 exm302525 A G XIRP1 24 37

3 39230072 exm302533 A G XIRP1 1 0

3 39230281 exm302547 A G XIRP1 1 1

3 39307063 exm302606 A G CX3CR1 3 7

3 39307162 exm302610 A G CX3CR1 20 44

3 39433345 exm302715 A G SLC25A38 7 10

3 40524168 exm303132 A G ZNF619 6 10

3 40529046 exm303162 G A ZNF619 6 15

3 40529155 exm303167 A G ZNF619 2 5

3 40553958 exm303213 A G ZNF620 4 8

3 40553984 exm303215 C G ZNF620 1 0

3 40557891 exm303234 T A ZNF620 1 5

3 40558088 exm303247 A G ZNF620 2 2

3 40573556 exm303278 G A ZNF621 1 1

3 40574249 exm303311 G A ZNF621 0 2

3 41723244 exm303449 C G ULK4 3 4

3 41938364 exm303544 G C ULK4 10 17

3 42242363 exm303714 G A TRAK1 4 9

3 42243951 exm303732 A G TRAK1 22 38

3 42264948 exm303832 A G TRAK1 3 2

3 42675205 exm304084 A C NKTR 4 10

3 42675220 exm304085 A G NKTR 1 2

3 42680033 exm304157 G C NKTR 15 31

3 42680854 exm304187 G A NKTR 6 19

3 42738359 exm304439 A G HHATL 0 11

3 42740553 exm304479 A C HHATL 2 4

3 42751249 exm304509 A G CCDC13 0 1

3 43096966 exm304868 A G GASK1A 21 32

3 43121705 exm304912 A G POMGNT2 3 10

3 43122436 exm304959 G A POMGNT2 3 7

3 43408427 exm305083 G A ANO10 9 25

3 43618202 exm305121 A G ANO10 1 2

3 43618558 exm305144 A G ANO10 3 3

3 44283844 exm305263 G A TOPAZ1 16 33

3 44488293 exm305413 G A ZNF445 8 22

3 44489137 exm305442 A G ZNF445 5 4

3 44489700 exm305457 A G ZNF445 0 2

3 44490049 exm305468 G A ZNF445 4 22

3 44612684 exm305619 C A ZKSCAN7 4 11

3 44635975 exm305650 A G ZNF660 5 8

3 44683983 exm305736 A T ZNF197;ZNF660-ZNF197 7 16

3 44776337 exm305945 A G ZNF501 8 14

3 44835838 exm306038 A G KIF15 4 5

3 44844424 exm306062 G C KIF15 1 3

3 44852341 exm306079 G A KIF15 2 7

3 44852497 exm306084 C G KIF15 2 7

3 44856496 exm306099 G C KIF15 10 14

3 44856528 exm306104 A G KIF15 8 14

3 44881897 exm306141 A C KIF15 7 10

3 44938291 exm306254 G A TGM4 7 20

3 44943401 exm306284 C A TGM4 13 34

3 44945443 exm306291 A C TGM4 21 31

3 45160038 exm306588 A C CDCP1 1 5

3 45588930 exm306748 A G LARS2 1 1

3 45636805 exm306775 A G LIMD1 15 22

3 45751035 exm306903 C A SACM1L 2 9

3 45811741 exm307015 A G SLC6A20 11 29

3 45817414 exm307051 G A SLC6A20 11 37

3 45821536 exm307058 A G SLC6A20 4 3

3 45942538 exm307127 A C CCR9 1 2

3 45942584 exm307129 A G CCR9 0 1

3 45999920 exm307246 C G FYCO1 20 36

3 46003762 exm307270 C A FYCO1 3 3

3 46003807 exm307272 A G FYCO1 2 2

3 46003831 exm307273 G A FYCO1 11 35

3 46005863 exm307278 A C FYCO1 12 22

3 46009387 exm307372 A G FYCO1 19 35

3 46009763 exm307387 A G FYCO1 20 33

3 46009957 exm307398 A G FYCO1 22 43

3 46062676 exm307455 C A XCR1 9 26

3 46062776 exm307459 A G XCR1 3 13

3 46244937 exm307499 A G CCR1 5 6

3 46245329 exm307505 A G CCR1 9 32

3 46307313 exm307562 G A CCR3 16 21

3 46307695 exm307582 A G CCR3 2 8

3 46307701 exm307583 G A CCR3 16 29

3 46414529 exm307642 A G CCR5 2 7

3 46414712 exm307656 A G CCR5 2 1

3 46414766 exm307661 A G CCR5 1 1

3 46415066 exm307689 A G CCR5 2 1

3 46449637 exm307714 A G CCRL2 5 6

3 46449751 exm307722 A G CCRL2 17 21

3 46450190 exm307742 A G CCRL2 15 33

3 46450297 exm307746 G A CCRL2 21 40

3 46450472 exm307751 A G CCRL2 0 3

3 46450604 exm307756 C A CCRL2 13 41

3 46486841 exm307802 A C LTF 7 18

3 46487937 exm307810 A G LTF 2 3

3 46490497 exm307836 A C LTF 9 17

3 46497342 exm307879 G A LTF 4 9

3 46542052 exm307935 A G RTP3 8 16

3 46563074 exm307959 A C LRRC2 5 10

3 46586682 exm307996 G A LRRC2 0 1

3 46593054 exm308007 C A LRRC2 4 12

3 46935449 exm308530 A G PTH1R 11 30

3 46945000 exm308619 A G PTH1R 1 2

3 47036859 exm308756 A G NBEAL2 13 20

3 47036892 exm308759 A G NBEAL2 1 8

3 47037437 exm308783 A G NBEAL2 8 14

3 47041706 exm308860 C G NBEAL2 2 4

3 47044679 exm308930 A G NBEAL2 18 29

3 47048629 exm309006 A C NBEAL2 3 7

3 47049387 exm309019 A G NBEAL2 3 7

3 47103773 exm309107 G A SETD2 11 14

3 47125458 exm309131 A G SETD2 5 13

3 47162886 exm309254 A G SETD2 10 26

3 47163343 exm309273 C G SETD2 24 42

3 47163676 exm309285 G A SETD2 5 13

3 47163824 exm309290 C G SETD2 11 29

3 47163875 exm309291 A C SETD2 29 43

3 47284676 exm309389 C A KIF9 3 13

3 47308746 exm309447 A G KIF9 1 2

3 47312813 exm309452 A G KIF9 0 1

3 47312827 exm309454 A G KIF9 17 31

3 47316952 exm309470 G A KIF9 0 1

3 47374744 exm309506 A G KLHL18 1 8

3 47378219 exm309524 G A KLHL18 5 3

3 47957480 exm310532 A G MAP4 13 30

3 47957717 exm310547 A G MAP4 3 1

3 47958574 exm310578 G C MAP4 3 1

3 48040283 exm310618 A G MAP4 3 14

3 48414274 exm310831 A G FBXW12 0 1

3 48419898 exm310864 A G FBXW12 4 6

3 48453279 exm310962 A G PLXNB1 14 30

3 48456440 exm311018 A G PLXNB1 2 5

3 48461377 exm311112 A G PLXNB1 26 47

3 48508733 exm311433 A G TREX1 26 47

3 48508774 exm311437 G C TREX1 0 1

3 48508793 exm311439 C G TREX1 24 46

3 48603736 exm311635 A G COL7A1 1 1

3 48610968 exm311754 A G COL7A1 12 19

3 48617085 exm311847 A G COL7A1 0 1

3 48619376 exm311893 C G COL7A1 3 12

3 48619779 exm311897 A G COL7A1 10 23

3 48622363 exm311962 A G COL7A1 6 6

3 48638801 exm312277 A G UQCRC1 5 5

3 48643235 exm312300 A C UQCRC1 19 46

3 48677153 exm312493 A G CELSR3 1 0

3 48677569 exm312513 A G CELSR3 0 5

3 48680265 exm312560 A G CELSR3 3 5

3 48680431 exm312566 A G CELSR3 2 0

3 48682503 exm312583 G A CELSR3 16 19

3 48683218 exm312612 A G CELSR3 5 3

3 48697329 exm312803 C A CELSR3 0 1

3 48716148 exm312932 G A NCKIPSD 9 17

3 48716361 exm312934 A G NCKIPSD 1 3

3 48718827 exm312970 A G NCKIPSD 1 4

3 48719798 exm312996 A G NCKIPSD 2 4

3 48732618 exm313071 G A IP6K2 3 3

3 49039984 exm313278 G A P4HTM 3 3

3 49040002 exm313280 A G P4HTM 5 5

3 49044290 exm313315 A G P4HTM 1 4

3 49049934 exm313366 A G WDR6 8 17

3 49050304 exm313380 A G WDR6 9 21

3 49050499 exm313395 A G WDR6 3 3

3 49052453 exm313471 A G WDR6 3 6

3 49054758 exm313525 A C DALRD3 14 23

3 49136421 exm313765 A G QARS 8 17

3 49147897 exm313879 A C USP19 5 9

3 49149169 exm313911 A G USP19 11 31

3 49150049 exm313929 A G USP19 15 33

3 49156473 exm314031 G C USP19 17 37

3 49160987 exm314124 T A LAMB2 4 16

3 49162268 exm314167 G A LAMB2 1 3

3 49162469 exm314172 A T LAMB2 0 3

3 49165978 exm314209 A G LAMB2 9 25

3 49166718 exm314229 A G LAMB2 5 5

3 49168821 exm314270 A G LAMB2 4 17

3 49293819 exm314457 C A CCDC36 3 4

3 49294425 exm314483 A G CCDC36 3 12

3 49321998 exm314562 C G USP4 1 5

3 49330026 exm314581 G A USP4 17 37

3 49335360 exm314603 G A USP4 1 4

3 49377436 exm314677 C G USP4 0 1

3 49455040 exm314764 A G AMT 11 25

3 49455386 exm314779 G A AMT 17 26

3 49456758 exm314790 A G AMT 5 13

3 49548226 exm314883 G A DAG1 3 4

3 49548245 exm314885 G A DAG1 1 4

3 49691290 exm315179 G A BSN 8 16

3 49691430 exm315184 A T BSN 18 45

3 49692096 exm315219 G A BSN 10 20

3 49698498 exm315392 A G BSN 11 25

3 49698732 exm315406 A G BSN 1 1

3 49715033 exm315581 C G APEH 0 4

3 49718643 exm315597 A G APEH 3 5

3 49737954 exm315855 A G RNF123 14 23

3 49749976 exm315947 A G RNF123 6 13

3 49756129 exm316023 A G AMIGO3 0 1

3 49832788 exm316236 A G CDHR4 26 50

3 49842319 exm316316 A G INKA1 21 36

3 49842325 exm316318 A C INKA1 17 24

3 49845170 exm316341 A G UBA7 19 32

3 49847614 exm316388 A G UBA7 0 2

3 49847997 exm316404 A G UBA7 3 11

3 49950751 exm316873 A G MON1A 18 43

3 50326292 exm317554 A G IFRD2 1 0

3 50326986 exm317576 A C IFRD2 9 13

3 50339622 exm317799 A G HYAL1 0 2

3 50392847 exm318152 A G TMEM115 0 2

3 50395926 exm318163 A C TMEM115 9 18

3 50395984 exm318167 G A TMEM115 13 33

3 50615262 exm318400 A G HEMK1 6 18

3 50617609 exm318416 A G HEMK1 1 4

3 51101887 exm318529 C A DOCK3 1 1

3 51266985 exm318567 A G DOCK3 1 0

3 51367600 exm318600 C G DOCK3 5 12

3 51929069 exm319313 A G IQCF1 9 23

3 51929096 exm319314 A G IQCF1 1 2

3 51970535 exm319396 A G RRP9 5 16

3 51978193 exm319436 A G PARP3 12 36

3 51978494 exm319443 G A PARP3 12 25

3 52159166 exm319922 A G POC1A 3 9

3 52183872 exm319955 A G POC1A 3 4

3 52185053 exm319961 G A POC1A 3 4

3 52233264 exm319971 G A ALAS1 9 12

3 52233384 exm319984 A G ALAS1 5 2

3 52236580 exm319991 C G ALAS1 4 14

3 52242152 exm320038 G A ALAS1 5 18

3 52256157 exm320142 C G TLR9 16 28

3 52256315 exm320152 C G TLR9 2 11

3 52256621 exm320166 A G TLR9 3 14

3 52356727 exm320492 A G DNAH1 2 3

3 52378603 exm320551 A G DNAH1 10 17

3 52380519 exm320564 A G DNAH1 10 17

3 52384020 exm320612 A G DNAH1 2 6

3 52387519 exm320663 G A DNAH1 11 27

3 52390692 exm320685 C G DNAH1 0 1

3 52394055 exm320721 A G DNAH1 5 11

3 52397071 exm320749 A G DNAH1 10 28

3 52404776 exm320819 G A DNAH1 1 9

3 52409379 exm320844 C A DNAH1 6 11

3 52409393 exm320847 C A DNAH1 22 36

3 52409421 exm320851 G A DNAH1 26 43

3 52417508 exm320889 A G DNAH1 3 9

3 52420180 exm320916 A T DNAH1 2 11

3 52420199 exm320920 C G DNAH1 11 30

3 52420231 exm320921 G A DNAH1 0 2

3 52420751 exm320930 C A DNAH1 11 21

3 52422898 exm320958 A T DNAH1 22 32

3 52423541 exm320964 G A DNAH1 1 3

3 52427491 exm321021 G A DNAH1 1 8

3 52428959 exm321031 G A DNAH1 11 27

3 52433600 exm321107 C G DNAH1 4 6

3 52535766 exm321762 A G STAB1 10 32

3 52539369 exm321829 A G STAB1 1 1

3 52550569 exm322013 A G STAB1 7 11

3 52550722 exm322017 A G STAB1 6 11

3 52556184 exm322178 G C STAB1 9 18

3 52727275 exm322600 C G GNL3 8 19

3 52727807 exm322621 G A GNL3 23 45

3 52728055 exm322622 C A GNL3 3 15

3 52729969 exm322653 G A GLT8D1 7 11

3 52780205 exm322735 G A NEK4 1 5

3 52799944 exm322776 G A NEK4 7 17

3 52818446 exm322892 A G ITIH1 14 28

3 52820356 exm322906 G A ITIH1 0 8

3 52825574 exm322985 A G ITIH1 4 9

3 52833924 exm323052 A G ITIH3 2 10

3 52834608 exm323054 A G ITIH3 17 52

3 52837989 exm323098 A G ITIH3 3 13

3 53126443 exm323528 C A RFT1 6 7

3 53133487 exm323549 A G RFT1 13 27

3 53156464 exm323582 A G RFT1 8 24

3 53226232 exm323712 A G PRKCD 4 9

3 53269028 exm323799 A C TKT 0 3

3 53835422 exm324105 A G CACNA1D 13 16

3 53845384 exm324164 A G CACNA1D 6 11

3 53891003 exm324301 C A IL17RB 14 23

3 53891665 exm324308 A G IL17RB 17 30

3 53892830 exm324319 G A IL17RB 3 0

3 53899178 exm324341 G A IL17RB 7 10

3 54925426 exm324557 A G CACNA2D3 1 5

3 54933872 exm324565 G A CACNA2D3 3 5

3 54952734 exm324588 G C LRTM1 4 9

3 54958901 exm324612 C G LRTM1 9 27

3 55018689 exm324642 C A CACNA2D3 5 7

3 55922576 exm324741 G A ERC2 3 7

3 56114861 exm324777 G A ERC2 6 17

3 56657447 exm324998 G A TASOR 3 7

3 56667377 exm325035 A G TASOR 0 1

3 56674140 exm325070 A G TASOR 3 2

3 57108101 exm325231 A G SPATA12 3 5

3 57108282 exm325242 G A SPATA12 3 15

3 57389124 exm325606 A G DNAH12 0 2

3 57401178 exm325625 C G DNAH12 6 14

3 57419482 exm325660 T A DNAH12 13 28

3 57448553 exm325712 A G DNAH12 14 33

3 57496630 exm325783 G A DNAH12 6 13

3 57528440 exm325800 C A DNAH12 2 6

3 57528509 exm325805 G A DNAH12 14 29

3 57542456 exm325824 G A PDE12 5 12

3 57994565 exm326069 G A FLNB 16 36

3 58088056 exm326114 G A FLNB 11 32

3 58107156 exm326193 A G FLNB 2 5

3 58108856 exm326204 A G FLNB 15 28

3 58108862 exm326205 C A FLNB 5 15

3 58135658 exm326385 G A FLNB 0 2

3 58140566 exm326412 G A FLNB 9 20

3 58178478 exm326482 A G DNASE1L3 0 1

3 58179089 exm326487 G A DNASE1L3 1 4

3 58183636 exm326495 A G DNASE1L3 1 2

3 58302285 exm326607 G A RPP14 10 18

3 58302657 exm326608 A C RPP14 0 1

3 58302718 exm326612 G C RPP14 11 22

3 58376908 exm326640 G C PXK 5 14

3 58394693 exm326675 G A PXK 7 16

3 58395281 exm326679 A G PXK 2 8

3 58503063 exm326795 A G ACOX2 2 8

3 58516239 exm326836 A G ACOX2 3 9

3 58520228 exm326895 A G ACOX2 16 22

3 58520731 exm326904 A G ACOX2 0 1

3 58520737 exm326905 A G ACOX2 12 21

3 58739502 exm327000 G C C3orf67 0 1

3 58855204 exm327054 A G C3orf67 1 4

3 62153771 exm327201 A G PTPRG 13 22

3 62189570 exm327263 G A PTPRG 1 0

3 62258700 exm327322 A G PTPRG 16 27

3 62385193 exm327418 A G CADPS 2 6

3 62501769 exm327475 G A CADPS 8 20

3 62631482 exm327527 A C CADPS 8 17

3 63968089 exm327713 G A ATXN7 5 13

3 63973888 exm327723 A G ATXN7 5 12

3 63981977 exm327789 G A ATXN7 2 15

3 64507857 exm327952 A G ADAMTS9 0 1

3 64526815 exm327977 A G ADAMTS9 20 35

3 64601793 exm328128 A G ADAMTS9 1 2

3 64617162 exm328149 G C ADAMTS9 1 2

3 64619218 exm328162 A G ADAMTS9 1 1

3 65415516 exm328391 A G MAGI1 8 17

3 65479192 exm328465 A T MAGI1 8 21

3 66023926 exm328490 A G MAGI1 5 10

3 66431279 exm328603 A C LRIG1 13 33

3 66433730 exm328665 A G LRIG1 2 2

3 66434696 exm328693 G A LRIG1 5 11

3 66465378 exm328781 A G LRIG1 15 21

3 67054564 exm328868 T A KBTBD8 3 5

3 69031963 exm329047 G A EOGT 6 15

3 69058876 exm329098 A C EOGT 13 22

3 69082708 exm329144 G C TMF1 21 39

3 69082740 exm329146 A G TMF1 18 26

3 69097630 exm329223 A G TMF1 26 44

3 69167851 exm329335 A C LMOD3 1 2

3 69168718 exm329381 G A LMOD3 13 34

3 69171290 exm329409 A G LMOD3 2 6

3 69171291 exm329410 A G LMOD3 8 13

3 69230566 exm329480 G A FRMD4B 2 5

3 69231756 exm329500 C G FRMD4B 5 5

3 69242802 exm329518 A G FRMD4B 17 31

3 69243011 exm329524 G A FRMD4B 12 21

3 69244252 exm329534 G A FRMD4B 5 13

3 69265419 exm329561 A G FRMD4B 1 3

3 71015105 exm329716 A G FOXP1 4 11

3 71015168 exm329723 A G FOXP1 1 3

3 71019907 exm329730 A G FOXP1 12 25

3 72799870 exm329934 C A SHQ1 2 2

3 72897412 exm329972 A G SHQ1 3 2

3 73111447 exm330069 G A EBLN2 1 4

3 73111900 exm330108 A C EBLN2 7 13

3 74334458 exm330358 A G CNTN3 1 0

3 74334561 exm330362 A G CNTN3 5 7

3 74384005 exm330415 A G CNTN3 4 17

3 74535742 exm330459 C G CNTN3 15 30

3 77530342 exm330886 C G ROBO2 16 48

3 77623717 exm330939 A G ROBO2 0 1

3 77651445 exm330976 A G ROBO2 2 14

3 78710296 exm331132 A G ROBO1 6 19

3 78717699 exm331155 A G ROBO1 6 18

3 81539593 exm331234 A G GBE1 6 9

3 81640290 exm331281 C A GBE1 12 24

3 88188791 exm331570 A G ZNF654 3 10

3 88188798 exm331571 G A ZNF654 1 2

3 88189878 exm331596 A G ZNF654 5 13

3 93783369 exm331987 A G NSUN3 3 3

3 93813980 exm332013 T A NSUN3 1 4

3 97202829 exm332099 A C EPHA6 3 6

3 97251297 exm332105 A G EPHA6 1 3

3 97439178 exm332128 A G EPHA6 4 9

3 97466388 exm332144 G A EPHA6 12 34

3 97668796 exm332304 A G RIOX2 0 1

3 97677933 exm332321 A G RIOX2 0 1

3 97686251 exm332358 G C RIOX2 1 2

3 98309571 exm333152 A G CPOX 9 19

3 98520449 exm333264 A G DCBLD2 3 14

3 98526982 exm333269 G A DCBLD2 6 5

3 99568437 exm333508 G A FILIP1L 12 31

3 99568634 exm333515 C G FILIP1L 10 13

3 99568956 exm333528 C A FILIP1L 2 8

3 100058733 exm333739 G C NIT2 0 2

3 100059943 exm333742 C A NIT2 2 4

3 100148679 exm333832 A G LNP1 0 1

3 100170715 exm333851 A T LNP1 1 4

3 100352151 exm333941 G A ADGRG7 7 23

3 100365420 exm333978 G A ADGRG7 1 2

3 100365533 exm333987 A G ADGRG7 2 4

3 100378687 exm334017 A G ADGRG7 0 1

3 100413760 exm334032 G A ADGRG7 12 25

3 100451393 exm334053 C A TFG 1 0

3 100467232 exm334078 G C TFG 18 33

3 100523692 exm334138 G C ABI3BP 25 40

3 100605084 exm334202 G A ABI3BP 0 9

3 101039185 exm334370 A C IMPG2 2 4

3 101060536 exm334400 A G SENP7 12 26

3 101062748 exm334409 G A SENP7 0 1

3 101086729 exm334446 G A SENP7 11 22

3 101445570 exm334720 A G CEP97 9 11

3 101474398 exm334752 A G CEP97 3 5

3 101476672 exm334766 A C CEP97 8 14

3 105258845 exm335105 A G ALCAM 3 11

3 105258861 exm335107 G A ALCAM 30 60

3 105377983 exm335153 A G CBLB 1 7

3 105438920 exm335224 G A CBLB 6 18

3 107096868 exm335296 A T CCDC54 8 13

3 107097030 exm335303 A G CCDC54 0 2

3 107097072 exm335304 G A CCDC54 12 24

3 107097407 exm335316 A G CCDC54 3 6

3 107435605 exm335334 C A BBX 8 19

3 107517405 exm335418 G C BBX 7 12

3 107881463 exm335482 G A IFT57 0 1

3 107884324 exm335489 G A IFT57 13 21

3 108074105 exm335564 C G HHLA2 1 3

3 108076849 exm335575 G A HHLA2 27 35

3 108100406 exm335600 A C MYH15 19 34

3 108110618 exm335623 G C MYH15 14 28

3 108112954 exm335628 A G MYH15 2 5

3 108147680 exm335719 A G MYH15 2 3

3 108181993 exm335794 A G MYH15 10 23

3 108276207 exm335921 T A CIP2A 6 18

3 108279656 exm335936 G A CIP2A 15 17

3 108300348 exm335984 A C CIP2A 7 8

3 108303989 exm336001 A C CIP2A 0 3

3 108335445 exm336026 A G DZIP3 0 1

3 108347982 exm336038 G A DZIP3 0 1

3 108366888 exm336084 A G DZIP3 15 20

3 108474696 exm336171 A C RETNLB 1 5

3 108475945 exm336179 A G RETNLB 0 4

3 108698491 exm336295 G A MORC1 7 13

3 108723983 exm336321 A G MORC1 2 12

3 111316970 exm336667 G A CD96 13 34

3 111603046 exm336757 G A PHLDB2 2 1

3 111603493 exm336771 A G PHLDB2 8 18

3 111603633 exm336779 G A PHLDB2 14 30

3 111603790 exm336784 G A PHLDB2 27 65

3 111603794 exm336785 A T PHLDB2 1 4

3 111667833 exm336877 A T PHLDB2 3 4

3 111681152 exm336905 G A PHLDB2 11 23

3 111710284 exm336969 A G ABHD10 6 13

3 111710398 exm336976 G A ABHD10 9 23

3 111766795 exm337022 A G TMPRSS7 1 1

3 111780734 exm337051 A G TMPRSS7 1 4

3 111842346 exm337174 A G GCSAM 5 6

3 111842534 exm337186 A G GCSAM 7 5

3 111873869 exm337231 C A SLC9C1 6 14

3 111901099 exm337269 G A SLC9C1 8 15

3 111923210 exm337292 G A SLC9C1 32 43

3 111927206 exm337301 A G SLC9C1 1 3

3 111993793 exm337361 T A SLC9C1 7 12

3 112324305 exm337573 G A CCDC80 2 2

3 112357548 exm337650 G A CCDC80 12 28

3 112357668 exm337659 G C CCDC80 7 18

3 112727222 exm337905 A C NEPRO 4 5

3 112730047 exm337921 G A NEPRO 0 6

3 113002261 exm338151 A G BOC 12 18

3 113049376 exm338246 A C CFAP44 6 16

3 113085143 exm338273 G A CFAP44 2 1

3 113092306 exm338276 A G CFAP44 2 13

3 113092365 exm338280 A C CFAP44 9 20

3 113119409 exm338315 A G CFAP44 6 13

3 113164170 exm338378 G C SPICE1 0 5

3 113165488 exm338384 C A SPICE1 4 8

3 113172548 exm338414 C A SPICE1 2 8

3 113176009 exm338429 C A SPICE1 5 15

3 113179497 exm338437 A G SPICE1 19 45

3 113187648 exm338457 A G SPICE1 0 2

3 113285276 exm338520 A G SIDT1 4 8

3 113325954 exm338608 A G SIDT1 3 7

3 113327011 exm338611 A G SIDT1 0 1

3 113329939 exm338635 G A SIDT1 1 0

3 113344949 exm338662 A G SIDT1 9 29

3 113374627 exm338705 A G USF3 4 7

3 113376342 exm338773 G A USF3 7 7

3 113376484 exm338780 A G USF3 1 2

3 113378554 exm338854 C G USF3 11 33

3 113563381 exm339000 A G GRAMD1C 14 30

3 113634682 exm339038 C A GRAMD1C 4 4

3 113697749 exm339174 C A CCDC191 1 1

3 113699599 exm339185 C G CCDC191 11 43

3 113729789 exm339225 A G CCDC191 0 1

3 118649018 exm339708 G A IGSF11 12 12

3 118865140 exm339721 G A TEX55 5 17

3 118865621 exm339740 A C TEX55 13 28

3 118865730 exm339752 A G TEX55 6 4

3 118865806 exm339758 G A TEX55 2 4

3 118866003 exm339774 G A TEX55 17 42

3 118867055 exm339797 T A TEX55 9 15

3 118867069 exm339798 A G TEX55 1 1

3 118931401 exm339847 A C B4GALT4 1 0

3 118931403 exm339848 A G B4GALT4 16 37

3 119133106 exm339987 G A ARHGAP31 7 13

3 119133120 exm339988 A C ARHGAP31 7 14

3 119134119 exm340031 C A ARHGAP31 10 7

3 119134234 exm340036 G A ARHGAP31 14 28

3 119155746 exm340072 A G TMEM39A 3 6

3 119171308 exm340099 A G TMEM39A 4 6

3 119222832 exm340189 G A TIMMDC1 8 20

3 119347561 exm340380 A G PLA1A 22 43

3 119379264 exm340454 A G POPDC2 12 15

3 119445077 exm340515 A G MAATS1 1 0

3 119469789 exm340591 A T MAATS1 6 16

3 119526120 exm340611 C G NR1I2 1 3

3 119533842 exm340660 A G NR1I2 17 26

3 119892232 exm340813 G A GPR156 3 3

3 119892272 exm340816 A G GPR156 10 14

3 120128482 exm340941 G A FSTL1 8 17

3 120352054 exm340996 G C HGD 4 9

3 120357389 exm341006 A G HGD 12 25

3 120500291 exm341154 A G GTF2E1 0 1

3 120952486 exm341198 A G STXBP5L 12 24

3 121126404 exm341266 A G STXBP5L 8 26

3 121155122 exm341309 A G POLQ 1 2

3 121207094 exm341409 A C POLQ 12 28

3 121207637 exm341434 A C POLQ 12 23

3 121207721 exm341439 G A POLQ 20 33

3 121215771 exm341524 A C POLQ 4 14

3 121350749 exm341815 A G HCLS1 3 4

3 121351257 exm341837 A G HCLS1 2 4

3 121351918 exm341852 A G HCLS1 5 7

3 121354620 exm341883 A G HCLS1 4 15

3 121377175 exm341921 G C HCLS1 9 16

3 121388141 exm341949 A G GOLGB1 3 4

3 121411420 exm342026 G A GOLGB1 8 13

3 121412702 exm342030 A G GOLGB1 4 11

3 121413176 exm342045 G A GOLGB1 5 11

3 121414165 exm342081 A C GOLGB1 1 0

3 121415370 exm342113 G A GOLGB1 0 2

3 121417641 exm342172 A G GOLGB1 7 40

3 121445881 exm342229 G A GOLGB1 16 31

3 121489286 exm342250 G A IQCB1 21 32

3 122003892 exm342728 A G CASR 1 9

3 122103120 exm342783 A G FAM162A 6 23

3 122128659 exm342807 G A FAM162A 6 19

3 122247449 exm342907 A G PARP9 1 2

3 122331994 exm343099 A G PARP15 2 1

3 122351058 exm343149 G A PARP15 2 13

3 122354766 exm343168 A G PARP15 18 25

3 122459520 exm343357 G A HSPBAP1 14 37

3 122474162 exm343374 A G HSPBAP1 9 13

3 122487657 exm343397 G A HSPBAP1 3 8

3 122645509 exm343588 A G SEMA5B 3 3

3 122646835 exm343605 A G SEMA5B 8 10

3 122662308 exm343622 A G SEMA5B 0 3

3 123337545 exm344039 A G MYLK 18 36

3 123385191 exm344140 G A MYLK 7 17

3 123444811 exm344267 A G MYLK 3 7

3 123452624 exm344287 A C MYLK 3 3

3 123663699 exm344425 A G CCDC14 5 10

3 124390722 exm344902 A G KALRN 17 24

3 124393226 exm344905 G A KALRN 3 4

3 124456424 exm345005 A G UMPS 5 13

3 124462813 exm345049 A G UMPS 5 13

3 124578125 exm345168 A T ITGB5 7 15

3 124578278 exm345179 A G ITGB5 1 3

3 124716596 exm345302 A G HEG1 9 20

3 124720710 exm345307 A G HEG1 0 1

3 124729336 exm345331 A G HEG1 4 13

3 124732102 exm345364 G A HEG1 17 34

3 124732334 exm345374 A G HEG1 14 28

3 124746241 exm345445 C G HEG1 12 24

3 124748129 exm345454 A G HEG1 7 22

3 124896668 exm345622 A G SLC12A8 23 40

3 124906235 exm345652 A G SLC12A8 19 27

3 125855608 exm346230 A G ALDH1L1 5 15

3 125865756 exm346272 A G ALDH1L1 6 11

3 126132999 exm346451 A C CFAP100 21 34

3 126137556 exm346480 A G CFAP100 1 1

3 126138949 exm346504 A G CFAP100 6 29

3 126158496 exm346587 A C ZXDC 1 2

3 126180503 exm346628 A G ZXDC 2 3

3 126180826 exm346648 G A ZXDC 6 10

3 126181042 exm346658 A G ZXDC 7 12

3 126191092 exm346679 G A ZXDC 2 4

3 126228436 exm346837 A G UROC1 9 20

3 126571571 exm346991 C A CHCHD6 18 59

3 126676329 exm347006 A G CHCHD6 12 27

3 127379473 exm347605 C G PODXL2 7 12

3 127396588 exm347746 A G ABTB1 2 5

3 128060445 exm348043 A G EEFSEC 3 11

3 128060547 exm348046 A G EEFSEC 0 1

3 128622922 exm348409 A G ACAD9 1 5

3 128622968 exm348412 G A ACAD9 3 2

3 128627887 exm348427 A G ACAD9 5 12

3 128696903 exm348492 A G KIAA1257 3 1

3 129120684 exm348884 A C EFCAB12 11 35

3 129137188 exm348940 A G EFCAB12 10 15

3 129155915 exm349030 A G MBD4 4 8

3 129179740 exm349076 A C IFT122 13 19

3 129183519 exm349087 G A IFT122 0 2

3 129185786 exm349096 G A IFT122 10 20

3 129196963 exm349131 A G IFT122 1 2

3 129218830 exm349205 C A IFT122 12 22

3 129225350 exm349240 C A IFT122 9 10

3 129238459 exm349293 A G IFT122 1 1

3 129290568 exm349613 G A PLXND1 2 2

3 129292495 exm349653 A C PLXND1 6 3

3 129294392 exm349679 A G PLXND1 8 16

3 129302489 exm349698 G A PLXND1 14 39

3 130095179 exm349931 G A COL6A5 0 1

3 130150310 exm350082 C A COL6A5 12 27

3 130159426 exm350107 G A COL6A5 4 5

3 130174433 exm350134 G A COL6A5 0 1

3 130187752 exm350142 A G COL6A5 11 22

3 130282344 exm350194 T A COL6A6 4 11

3 130284144 exm350220 A G COL6A6 0 1

3 130287078 exm350268 A C COL6A6 2 5

3 130289732 exm350287 G C COL6A6 0 2

3 130290007 exm350294 G A COL6A6 18 18

3 130290069 exm350300 A G COL6A6 2 3

3 130293208 exm350327 A G COL6A6 4 5

3 130300413 exm350339 A G COL6A6 4 9

3 130311412 exm350367 A C COL6A6 16 23

3 130340689 exm350411 A C COL6A6 1 5

3 130346199 exm350417 A G COL6A6 5 13

3 130353458 exm350418 G A COL6A6 4 8

3 130380600 exm350469 G A COL6A6 11 30

3 130405138 exm350536 G A PIK3R4 1 1

3 130422592 exm350548 A T PIK3R4 9 28

3 130732963 exm350740 A G ASTE1 9 21

3 130732991 exm350741 A C ASTE1 1 4

3 130733118 exm350749 C A ASTE1 5 9

3 130737383 exm350763 A G ASTE1 0 2

3 130743574 exm350792 G A ASTE1 8 15

3 130748640 exm350815 A G NEK11 5 9

3 130881308 exm350860 A G NEK11 10 19

3 130889695 exm350880 G A NEK11 2 10

3 131181683 exm350965 A G MRPL3 10 23

3 131206542 exm350986 C A MRPL3 0 5

3 132193820 exm351248 G A DNAJC13 7 6

3 132193853 exm351249 A C DNAJC13 0 2

3 132218621 exm351323 A G DNAJC13 1 0

3 132222224 exm351343 C A DNAJC13 3 3

3 132226100 exm351352 G A DNAJC13 15 43

3 132247160 exm351408 C A DNAJC13 1 6

3 132294653 exm351442 A G ACAD11 19 30

3 132297687 exm351458 A G ACAD11 10 22

3 132324000 exm351505 A C ACAD11 3 11

3 132337565 exm351514 A G ACAD11 0 2

3 132360965 exm351560 A G ACAD11 3 10

3 132378459 exm351576 G A ACAD11 25 55

3 133320184 exm352009 C A TOPBP1 8 29

3 133336147 exm352035 A G TOPBP1 0 1

3 133342192 exm352052 A G TOPBP1 1 7

3 133363070 exm352096 T A TOPBP1 6 7

3 133371326 exm352108 A T TOPBP1 5 16

3 133673940 exm352450 A C SLCO2A1 12 37

3 133673951 exm352452 G C SLCO2A1 9 13

3 133692537 exm352459 A G SLCO2A1 6 9

3 133894485 exm352498 A G RYK 0 4

3 133928551 exm352529 G A RYK 8 16

3 134077454 exm352558 G A AMOTL2 4 8

3 134078137 exm352563 G C AMOTL2 16 28

3 134079224 exm352586 A C AMOTL2 16 26

3 134277173 exm352762 G A CEP63 5 5

3 134920337 exm352981 A G EPHB1 3 5

3 134967350 exm352996 A G EPHB1 9 27

3 135721120 exm353044 C A PPP2R3A 7 11

3 135722103 exm353094 A G PPP2R3A 7 12

3 135975431 exm353260 A G PCCB 14 19

3 136016804 exm353279 C G PCCB 10 11

3 136664737 exm353496 A G NCK1 5 10

3 136699398 exm353528 A C IL20RB 7 18

3 136729021 exm353553 A G IL20RB 4 12

3 137781729 exm353653 A G DZIP1L 7 21

3 137786448 exm353667 A G DZIP1L 1 1

3 137811385 exm353749 G A DZIP1L 0 3

3 137816662 exm353760 G A DZIP1L 7 12

3 137822698 exm353784 A G DZIP1L 1 0

3 137843194 exm353798 G A A4GNT 7 17

3 137843363 exm353806 A G A4GNT 5 15

3 137849888 exm353837 A G A4GNT 12 21

3 138180999 exm354123 A C ESYT3 7 16

3 138187016 exm354144 A G ESYT3 20 59

3 138188981 exm354160 A T ESYT3 6 16

3 138191347 exm354181 A G ESYT3 3 3

3 138191410 exm354184 A C ESYT3 9 25

3 138191662 exm354199 A G ESYT3 1 1

3 138329852 exm354319 G A FAIM 1 2

3 138347997 exm354341 A G FAIM 0 3

3 139297832 exm354829 A G NMNAT3 2 1

3 140123456 exm354858 C A CLSTN2 5 21

3 140277571 exm354944 A G CLSTN2 5 10

3 140277687 exm354952 A G CLSTN2 34 50

3 140281682 exm354963 A C CLSTN2 10 18

3 140281704 exm354965 A G CLSTN2 17 40

3 140284955 exm354992 A G CLSTN2 11 20

3 140401373 exm355023 A C TRIM42 4 16

3 140406674 exm355061 A G TRIM42 5 13

3 140409919 exm355105 A G TRIM42 21 32

3 141011752 exm355260 A G PXYLP1 0 1

3 141011859 exm355264 A G PXYLP1 18 31

3 141162128 exm355310 G C ZBTB38 34 64

3 141162179 exm355313 A G ZBTB38 5 5

3 141163253 exm355349 A G ZBTB38 1 0

3 142066120 exm355780 G A XRN1 9 25

3 142066144 exm355781 G A XRN1 9 11

3 142075762 exm355787 G C XRN1 9 15

3 142123830 exm355850 A G XRN1 7 23

3 142141763 exm355900 A C XRN1 4 11

3 142232369 exm356024 A G ATR 0 1

3 142281353 exm356169 C G ATR 3 1

3 142537266 exm356370 A G PCOLCE2 0 4

3 142542415 exm356380 A G PCOLCE2 11 20

3 143082401 exm356661 A G SLC9A9 0 3

3 143550956 exm356764 A G SLC9A9 14 30

3 145794607 exm356848 G A PLOD2 10 15

3 145804593 exm356884 A G PLOD2 4 15

3 145912919 exm356949 G A PLSCR4 7 10

3 145924348 exm356992 A C PLSCR4 23 39

3 145939852 exm357007 A C PLSCR4 3 8

3 148562272 exm357287 A G CPB1 6 15

3 148583113 exm357324 A G CPA3 0 3

3 148586742 exm357342 A G CPA3 14 18

3 148596317 exm357348 G A CPA3 3 6

3 148757849 exm357481 C A HLTF 10 22

3 148777576 exm357528 G A HLTF 12 36

3 148781246 exm357540 A T HLTF 12 17

3 148857916 exm357599 A G HPS3 3 10

3 148858175 exm357606 G A HPS3 0 1

3 148859084 exm357618 A G HPS3 7 10

3 148904439 exm357798 A G CP 15 27

3 148930310 exm357882 A G CP 17 36

3 149245675 exm358025 T A WWTR1 9 20

3 149375068 exm358050 A G WWTR1 2 13

3 150176316 exm358358 A C TSC22D2 6 20

3 150176352 exm358361 A C TSC22D2 7 17

3 150384637 exm358473 G C ERICH6 0 6

3 150398311 exm358504 A C ERICH6 2 2

3 150400030 exm358510 G A ERICH6 1 5

3 150931605 exm358808 G A P2RY14 26 59

3 150931827 exm358818 G A P2RY14 5 7

3 151011985 exm358837 A G GPR87 4 15

3 151012481 exm358859 G A GPR87 4 10

3 151046398 exm358923 G A P2RY13 1 5

3 151046506 exm358931 A C P2RY13 3 5

3 151107797 exm359096 G C MED12L 1 2

3 151129087 exm359114 G A MED12L 2 5

3 151154509 exm359157 A G IGSF10 1 4

3 151155519 exm359216 G A IGSF10 1 9

3 151155702 exm359224 A G IGSF10 6 16

3 151156104 exm359246 G A IGSF10 6 19

3 151163021 exm359316 G A IGSF10 1 1

3 151163838 exm359364 G A IGSF10 4 2

3 151163913 exm359368 G A IGSF10 17 36

3 151164674 exm359406 A G IGSF10 15 36

3 151164827 exm359415 A G IGSF10 9 19

3 151165610 exm359460 G A IGSF10 4 5

3 151165643 exm359462 A C IGSF10 5 18

3 151165770 exm359468 A C IGSF10 24 38

3 151166615 exm359507 A G IGSF10 0 2

3 153839947 exm359884 C G ARHGEF26 9 15

3 153943770 exm359958 G C ARHGEF26 11 19

3 153993974 exm359992 A G DHX36 5 7

3 154139047 exm360124 C A GPR149 15 18

3 154146545 exm360149 A G GPR149 9 33

3 154861298 exm360271 C A MME 14 28

3 155200562 exm360384 A G PLCH1 20 32

3 155203168 exm360393 G A PLCH1 17 28

3 155210598 exm360414 C A PLCH1 4 8

3 155314113 exm360499 A G PLCH1 1 0

3 156763196 exm360881 C G LEKR1 3 10

3 157081386 exm361022 A G VEPH1 13 27

3 158310353 exm361286 A C MLF1 2 5

3 158366924 exm361350 G A GFM1 1 3

3 158369983 exm361357 G A GFM1 9 16

3 158409143 exm361465 G A GFM1 1 3

3 159995431 exm361737 A G IFT80 12 28

3 159997046 exm361738 G A IFT80 1 2

3 160025451 exm361763 A G IFT80 1 0

3 160037568 exm361769 G A IFT80 7 22

3 160073857 exm361788 C G IFT80 2 4

3 160141230 exm361908 C A SMC4 6 18

3 160143905 exm361924 G A SMC4 16 28

3 160150107 exm361960 G A SMC4 9 17

3 160156040 exm361977 C G TRIM59 3 8

3 160395139 exm362101 A C ARL14 4 9

3 160395207 exm362107 A G ARL14 18 20

3 160395381 exm362117 A G ARL14 15 25

3 161214753 exm362318 G A OTOL1 1 10

3 161220819 exm362337 A C OTOL1 0 1

3 164697211 exm362381 A G SI 5 4

3 164709997 exm362422 A G SI 8 28

3 164710159 exm362427 C A SI 9 19

3 164712165 exm362440 C G SI 6 16

3 164725726 exm362469 A G SI 16 45

3 164735584 exm362503 A G SI 9 22

3 164741443 exm362535 C A SI 5 8

3 164741458 exm362536 C A SI 3 4

3 164750368 exm362554 A G SI 8 24

3 164750447 exm362558 G A SI 27 37

3 164755793 exm362575 A G SI 8 14

3 165491264 exm362806 G A BCHE 1 0

3 165503962 exm362813 A G BCHE 2 8

3 165547330 exm362822 A G BCHE 4 9

3 166958644 exm362892 A C ZBBX 4 8

3 167045787 exm362958 G A ZBBX 23 38

3 167051635 exm362969 A G ZBBX 18 34

3 167051649 exm362970 A G ZBBX 4 8

3 167196729 exm363073 A T WDR49 2 4

3 167245724 exm363095 G A WDR49 3 5

3 167246997 exm363107 A C WDR49 1 3

3 167248995 exm363116 A G WDR49 1 3

3 167507124 exm363195 G A SERPINI1 4 11

3 167543061 exm363235 G A SERPINI1 23 43

3 167728092 exm363245 A G GOLIM4 5 6

3 167728167 exm363250 A G GOLIM4 0 1

3 167747019 exm363272 G A GOLIM4 7 16

3 168806925 exm363374 G A MECOM 1 6

3 168812942 exm363392 A G MECOM 13 21

3 168825728 exm363401 A G MECOM 4 9

3 168833357 exm363413 A G MECOM 19 27

3 168834317 exm363448 G C MECOM 5 8

3 168861536 exm363490 A C MECOM 15 27

3 169485261 exm363548 G A ACTRT3 6 20

3 169539938 exm363699 G A LRRIQ4 1 1

3 169540713 exm363736 G A LRRIQ4 4 11

3 169546720 exm363743 C G LRRIQ4 3 3

3 169555412 exm363769 G A LRRIQ4 3 6

3 169558086 exm363781 A G LRRC31 4 4

3 169569410 exm363790 C G LRRC31 9 30

3 169572694 exm363799 A G LRRC31 2 10

3 169574628 exm363819 A G LRRC31 9 28

3 169801866 exm363957 A T GPR160 7 6

3 169802446 exm363974 G A GPR160 20 27

3 169840471 exm364018 A G PHC3 4 6

3 169846767 exm364035 G A PHC3 4 9

3 170216583 exm364338 G A SLC7A14 13 34

3 170244550 exm364374 G A SLC7A14 0 5

3 171417542 exm364705 A G PLD1 5 16

3 171455341 exm364746 A G PLD1 3 3

3 171455739 exm364752 G A PLD1 8 18

3 172013225 exm364811 G A FNDC3B 11 28

3 172098778 exm364938 C A FNDC3B 14 27

3 172224402 exm365019 C G TNFSF10 6 7

3 172232659 exm365035 A G TNFSF10 1 3

3 172607362 exm365204 G A SPATA16 0 1

3 172631461 exm365210 G A SPATA16 24 52

3 172766761 exm365250 A G SPATA16 12 27

3 174814750 exm365382 G C NAALADL2 1 5

3 174814772 exm365384 A G NAALADL2 20 33

3 174814780 exm365386 A G NAALADL2 3 4

3 174814909 exm365391 A G NAALADL2 9 9

3 174951734 exm365403 C G NAALADL2 4 17

3 175042049 exm365420 C A NAALADL2 3 14

3 178968543 exm365677 A C KCNMB3 18 30

3 178968848 exm365690 A G KCNMB3 7 11

3 179408054 exm365992 A G USP13 2 4

3 179460087 exm366044 A G USP13 3 6

3 179462842 exm366050 G C USP13 4 9

3 180320969 exm366201 A G TTC14 0 4

3 180326538 exm366246 C A TTC14 3 5

3 180327918 exm366272 G C TTC14 14 18

3 180332740 exm366289 A G CCDC39 6 15

3 180334356 exm366301 G A CCDC39 8 13

3 180359874 exm366338 A G CCDC39 3 10

3 180366117 exm366359 A C CCDC39 18 26

3 180379734 exm366395 G A CCDC39 25 51

3 182740282 exm366717 A C MCCC1 15 34

3 182756854 exm366742 A G MCCC1 5 10

3 182853654 exm366817 C A LAMP3 3 7

3 182871964 exm366857 A G LAMP3 2 6

3 182910862 exm366887 A G MCF2L2 0 2

3 183014825 exm367005 A G MCF2L2 2 10

3 183017822 exm367013 A T MCF2L2 5 11

3 183209738 exm367077 G C KLHL6 4 7

3 183226046 exm367136 C A KLHL6 1 2

3 183442244 exm367258 G A YEATS2 1 8

3 183476676 exm367306 A C YEATS2 10 21

3 183508650 exm367354 A C YEATS2 5 23

3 183508675 exm367357 A G YEATS2 4 6

3 183774713 exm367775 G A HTR3C 0 3

3 183778031 exm367820 G A HTR3C 7 16

3 183855449 exm367928 A G EIF2B5 3 9

3 183860628 exm367989 C G EIF2B5 2 7

3 183860633 exm367990 A G EIF2B5 1 5

3 183861951 exm368006 A G EIF2B5 3 11

3 183905528 exm368190 A G ABCF3 11 12

3 183908982 exm368266 A G ABCF3 3 5

3 183910431 exm368276 A G ABCF3 2 4

3 183911445 exm368303 A G ABCF3 2 2

3 183975266 exm368489 A G EEF1AKMT4;EEF1AKMT4-ECE2 10 33

3 183975459 exm368506 A G EEF1AKMT4;EEF1AKMT4-ECE2 5 14

3 184007447 exm368620 G A ECE2;EEF1AKMT4-ECE2 3 10

3 184008903 exm368647 A G ECE2;EEF1AKMT4-ECE2 0 1

3 184041329 exm368915 A G EIF4G1 0 2

3 184046720 exm369003 A G EIF4G1 3 6

3 184064408 exm369102 A G CLCN2 3 21

3 184073170 exm369205 A G CLCN2 3 5

3 184076081 exm369254 A G CLCN2 3 20

3 184093693 exm369336 A G THPO 1 3

3 184633203 exm369793 A C VPS8 0 1

3 184769715 exm369873 C G VPS8 7 22

3 184910025 exm369912 G A EHHADH 9 18

3 184910372 exm369931 G A EHHADH 5 6

3 184910381 exm369932 A G EHHADH 14 35

3 184910382 exm369933 A G EHHADH 2 11

3 184910520 exm369935 G A EHHADH 8 8

3 184910535 exm369937 A G EHHADH 0 1

3 185146543 exm370041 C G MAP3K13 7 18

3 185184738 exm370106 G A MAP3K13 15 30

3 185191071 exm370113 G C MAP3K13 15 33

3 185327128 exm370281 G A SENP2 18 24

3 186006607 exm370651 A G DGKG 1 4

3 186335088 exm370870 G C AHSG 14 48

3 186338397 exm370883 C G AHSG 6 15

3 186383975 exm370997 A G HRG 0 4

3 186394864 exm371040 A G HRG 13 41

3 186394957 exm371047 A T HRG 8 28

3 186395355 exm371069 G C HRG 5 19

3 186461524 exm371180 A G KNG1 0 1

3 186572026 exm371358 A G ADIPOQ 4 15

3 186572089 exm371362 G A ADIPOQ 6 2

3 186940937 exm371515 A C MASP1 1 13

3 186947680 exm371549 A G MASP1 10 21

3 186961364 exm371631 A G MASP1 5 13

3 187003786 exm371684 A G MASP1 0 2

3 187088578 exm371706 C A RTP4 2 6

3 187088613 exm371710 A G RTP4 25 37

3 187088923 exm371724 A G RTP4 14 19

3 187387016 exm371743 A G SST 2 4

3 187387022 exm371745 C A SST 1 0

3 187446988 exm371858 A G BCL6 9 10

3 187447027 exm371863 G A BCL6 6 11

3 187447219 exm371868 A G BCL6 12 23

3 188327322 exm371975 A G LPP 13 27

3 188327403 exm371984 A G LPP 5 12

3 188426103 exm372017 A G LPP 2 7

3 188925368 exm372072 A C TPRG1 2 2

3 188933091 exm372076 G A TPRG1 3 8

3 188933136 exm372079 A G TPRG1 4 6

3 190105985 exm372365 A G CLDN16 0 2

3 190573340 exm372548 G C GMNC 17 51

3 190573374 exm372549 C G GMNC 17 51

3 191087740 exm372632 T A CCDC50 11 17

3 191093028 exm372642 A G CCDC50 13 38

3 192517085 exm372779 G A MB21D2 3 10

3 192517142 exm372782 G A MB21D2 2 3

3 192992920 exm372844 A G ATP13A5 8 15

3 193002884 exm372867 C A ATP13A5 17 34

3 193029725 exm372899 G C ATP13A5 22 46

3 193031875 exm372900 A G ATP13A5 5 10

3 193032785 exm372905 A G ATP13A5 8 14

3 193032832 exm372906 A G ATP13A5 2 10

3 193039554 exm372928 A G ATP13A5 0 1

3 193071964 exm373001 A C ATP13A5 14 21

3 193130054 exm373082 A G ATP13A4 1 6

3 193132522 exm373101 G A ATP13A4 1 6

3 193174817 exm373149 A C ATP13A4 2 16

3 193175184 exm373155 C G ATP13A4 8 18

3 193185125 exm373195 A G ATP13A4 1 7

3 193185128 exm373196 G A ATP13A4 24 45

3 193332564 exm373267 G C OPA1 3 7

3 193332718 exm373275 G A OPA1 5 25

3 194062675 exm373500 G A CPN2 18 38

3 194062821 exm373512 A G CPN2 11 32

3 194063280 exm373534 A G CPN2 4 14

3 194080335 exm373570 A G LRRC15 2 1

3 194080824 exm373606 A G LRRC15 3 8

3 194081525 exm373650 A G LRRC15 3 4

3 194325055 exm373881 A G TMEM44 18 34

3 194338475 exm373921 A G TMEM44 19 37

3 194373747 exm374045 A G LSG1 8 11

3 194379839 exm374061 A T LSG1 28 61

3 194386945 exm374071 A G LSG1 1 9

3 195295958 exm374416 G A APOD 4 14

3 195300800 exm374428 C A APOD 20 36

3 195460073 exm374584 A G MUC20 1 3

3 195486123 exm374687 A G MUC4 0 1

3 195487823 exm374694 A G MUC4 3 13

3 195495983 exm374756 A G MUC4 6 19

3 195505182 exm374790 A G MUC4 5 7

3 195516730 exm375729 A G MUC4 0 1

3 195516982 exm375740 A G MUC4 1 5

3 195517441 exm375765 C A MUC4 7 19

3 195518170 exm375793 A G MUC4 16 38

3 195591056 exm375817 A G TNK2 13 24

3 195594494 exm375846 A G TNK2 1 4

3 196051191 exm376439 A G TM4SF19 8 13

3 196214445 exm376567 A G RNF168 16 25

3 196236520 exm376614 A G SMCO1 9 13

3 196387121 exm376716 A G NRROS 5 8

3 196388134 exm376770 C G NRROS 6 22

3 196388474 exm376794 A G NRROS 4 10

3 196454936 exm376836 A G PIGX 5 11

3 196455600 exm376840 A G PIGX 20 42

3 196674944 exm377098 A G PIGZ 9 14

3 196675166 exm377119 A G PIGZ 5 8

3 196675397 exm377133 A G PIGZ 1 0

3 196736546 exm377211 A G MELTF 4 6

3 196736573 exm377215 A G MELTF 0 3

3 196749829 exm377296 A G MELTF 1 0

3 196771513 exm377362 A G DLG1 11 23

3 196921352 exm377452 G A DLG1 2 5

3 196921360 exm377453 G A DLG1 3 11

3 197239157 exm377489 C G BDH1 2 4

3 197241283 exm377514 A G BDH1 16 43

3 197249592 exm377519 A G BDH1 13 32

3 197408056 exm377599 A G RUBCN 0 4

3 197408097 exm377602 G A RUBCN 3 11

3 197421270 exm377638 G A RUBCN 6 12

3 197432039 exm377721 A G RUBCN 2 6

3 197556508 exm377817 A G LRCH3 3 5

3 197574881 exm377854 A G LRCH3 10 13

3 197639571 exm377908 C A IQCG 8 19

3 197659051 exm377919 G A IQCG 6 14

3 197665521 exm377933 A G IQCG 0 3

3 197670837 exm377961 A G IQCG 1 0

3 197670846 exm377962 A G IQCG 1 4

3 111887792 JHU_3.111887791 A C SLC9C1 10 25

3 113652367 JHU_3.113652366 G A GRAMD1C 21 44

3 118621500 JHU_3.118621499 A G IGSF11 29 54

3 37458758 JHU_3.37458757 C A C3orf35 12 31

3 67053926 JHU_3.67053925 G A KBTBD8 11 31

3 178962425 newrs145138176 A G KCNMB3 6 9

3 46415393 newrs147879075 A G CCR5 3 3

4 10089489 4:10089489-CT A G WDR1 1 5

4 101401143 4:101401143-TC G A EMCN 0 1

4 102751160 4:102751160-TC G A BANK1 0 6

4 110772785 4:110772785-TC G A LRIT3 2 14

4 115544713 4:115544713-CT A G UGT8 7 14

4 115597182 4:115597182-AG G A UGT8 24 37

4 115997346 4:115997346-TC G A NDST4 4 0

4 120192955 4:120192955-AG G A USP53 5 9

4 121737661 4:121737661-TC G A PRDM5 0 1

4 126240483 4:126240483-GA A G FAT4 3 6

4 126373696 4:126373696-CT A G FAT4 2 3

4 129099645 4:129099645-AG G A LARP1B 1 1

4 129131119 4:129131119-GA A G LARP1B 2 3

4 1348570 4:1348570-GA A G UVSSA 9 30

4 13600878 4:13600878-TC G A BOD1L1 0 1

4 1360144 4:1360144-GA A G UVSSA 0 1

4 138451145 4:138451145-TC G A PCDH18 2 3

4 140454300 4:140454300-GA A G SETD7 4 15

4 144617793 4:144617793-GA A G FREM3 15 40

4 144617943 4:144617943-GA A G FREM3 14 40

4 144922413 4:144922413-TG C A GYPB 1 5

4 148575417 4:148575417-AG G A PRMT9 5 22

4 151023735 4:151023735-CT A G DCLK2 8 11

4 151408963 4:151408963-CT A G LRBA 2 4

4 151792545 4:151792545-CT A G LRBA 2 3

4 152499026 4:152499026-CT A G FAM160A1 14 37

4 152577924 4:152577924-GA A G FAM160A1 4 4

4 153332898 4:153332898-TC G A FBXW7 4 18

4 153896446 4:153896446-TC G A FHDC1 3 10

4 156835545 4:156835545-CT A G TDO2 1 2

4 15964388 4:15964388-GA A G FGFBP2 2 4

4 159789441 4:159789441-CA A C FNIP2 2 2

4 15992883 4:15992883-AG G A PROM1 0 2

4 159956209 4:159956209-CT A G C4orf45 0 4

4 16077396 4:16077396-TC G A PROM1 5 12

4 164393803 4:164393803-GA A G TKTL2 2 4

4 164775204 4:164775204-TC G A MARCHF1 0 6

4 165118794 4:165118794-GT A C ANP32C 10 18

4 169145401 4:169145401-GA A G DDX60 1 4

4 169201563 4:169201563-CT A G DDX60 1 3

4 175897599 4:175897599-CT A G ADAM29 1 7

4 183720992 4:183720992-AC C A TENM3 1 4

4 184210645 4:184210645-AG G A WWC2 4 4

4 186353232 4:186353232-TC G A C4orf47 9 18

4 186997817 4:186997817-TG C A TLR3 5 12

4 187122460 4:187122460-AC C A CYP4V2 3 10

4 187131710 4:187131710-AC C A CYP4V2 7 11

4 187509838 4:187509838-CT A G FAT1 4 14

4 189068102 4:189068102-CT A G TRIML1 1 3

4 22444485 4:22444485-GT A C ADGRA3 6 10

4 25849452 4:25849452-AG G A SEL1L3 3 5

4 265154 4:265154-CT A G ZNF732 4 12

4 265807 4:265807-CA A C ZNF732 8 22

4 2990546 4:2990546-AG G A GRK4 9 12

4 3137674 4:3137674-GA A G HTT 7 12

4 36286062 4:36286062-AG G A DTHD1 2 5

4 36292219 4:36292219-GA A G DTHD1 13 25

4 36309855 4:36309855-TC G A DTHD1 2 6

4 38945167 4:38945167-GT A C FAM114A1 1 1

4 39217613 4:39217613-GA A G WDR19 1 1

4 40810397 4:40810397-TC G A NSUN7 0 1

4 4322670 4:4322670-CT A G ZBTB49 0 2

4 47525052 4:47525052-GA A G ATP10D 7 12

4 47765474 4:47765474-CT A G CORIN 1 2

4 52926953 4:52926953-CT A G SPATA18 3 2

4 53610639 4:53610639-TG C A ERVMER34-1 4 9

4 53611468 4:53611468-GT A C ERVMER34-1 13 15

4 55136805 4:55136805-GA A G PDGFRA 4 6

4 55138603 4:55138603-CT A G PDGFRA 2 16

4 57797227 4:57797227-CT A G REST 23 35

4 6290723 4:6290723-CT A G WFS1 20 42

4 6303534 4:6303534-CT A G WFS1 4 8

4 6595027 4:6595027-GA A G MAN2B2 2 2

4 68374781 4:68374781-AG G A CENPC 0 10

4 69093649 4:69093649-TG C A TMPRSS11B 1 2

4 69098107 4:69098107-AG G A TMPRSS11B 1 2

4 69535925 4:69535925-TC G A UGT2B15 2 0

4 70350974 4:70350974-GA A G UGT2B4 8 17

4 70504857 4:70504857-CA A C UGT2A1;UGT2A2 0 1

4 70513281 4:70513281-TC G A UGT2A1 0 2

4 71275653 4:71275653-AC C A OPRPN 3 7

4 7435045 4:7435045-GA A G PSAPL1 0 1

4 75937991 4:75937991-GA A G PARM1 12 17

4 76532514 4:76532514-AG G A CDKL2 14 26

4 76882182 4:76882182-CT A G SDAD1 1 3

4 77189901 4:77189901-CT A G FAM47E;FAM47E-STBD1 13 28

4 77228029 4:77228029-CT A G STBD1 6 12

4 78804419 4:78804419-GA A G MRPL1 1 9

4 79301101 4:79301101-GA A G FRAS1 0 7

4 79400662 4:79400662-GA A G FRAS1 0 1

4 79832175 4:79832175-GT A C BMP2K 10 23

4 81188061 4:81188061-CT A G FGF5 1 2

4 8233747 4:8233747-GT A C SH3TC1 10 14

4 88106822 4:88106822-CT A G KLHL8 3 3

4 88766942 4:88766942-GA A G MEPE 0 1

4 88957395 4:88957395-AC C A PKD2 0 1

4 89034589 4:89034589-CT A G ABCG2 0 2

4 98893528 4:98893528-TC G A STPG2 6 13

4 106157768 exm2063198 A G TET2 2 1

4 147830349 exm2067037 G A TTC29 5 23

4 154626355 exm2067782 G A TLR2 6 10

4 169348307 exm2069343 C G DDX60L 16 25

4 2083438 exm2071957 A C POLN 2 1

4 3495182 exm2073368 A G DOK7 1 0

4 37863204 exm2073593 G A PGM2 5 20

4 57357683 exm2076512 G A SRP72 0 1

4 72433547 exm2078447 G A SLC4A4 1 3

4 38907407 exm2256598 C A FAM114A1 9 15

4 114280136 exm2274045 C G ANK2 0 1

4 52861978 exm2275740 A G LRRC66 0 2

4 165961984 exm2277276 G A TRIM60 2 4

4 266171 exm378389 A G ZNF732 1 4

4 289272 exm378393 A G ZNF732 0 1

4 435522 exm378508 G A ZNF721 3 15

4 499568 exm378683 A G PIGG 10 22

4 501347 exm378691 A G PIGG 8 19

4 515567 exm378735 A G PIGG 23 53

4 515677 exm378743 A G PIGG 11 26

4 517639 exm378778 A G PIGG 0 1

4 520962 exm378790 A G PIGG 14 23

4 628493 exm378871 A G PDE6B 4 5

4 661663 exm379023 A G PDE6B 16 31

4 843508 exm379302 A G GAK 14 28

4 843720 exm379319 G A GAK 8 18

4 845720 exm379357 A G GAK 19 24

4 861143 exm379431 A G GAK 13 24

4 882746 exm379552 A G GAK 8 21

4 944278 exm379636 A G TMEM175 3 8

4 946233 exm379651 A G TMEM175 17 21

4 949223 exm379668 A G TMEM175 10 14

4 1343314 exm380740 A G UVSSA 9 18

4 1348576 exm380819 A G UVSSA 6 10

4 1725489 exm381372 A G TACC3 4 6

4 1843324 exm381852 A G LETM1 11 32

4 1843342 exm381854 G A LETM1 0 1

4 1940236 exm381948 C G NSD2 3 12

4 1956968 exm381983 A G NSD2 15 12

4 2077199 exm382244 A G POLN 14 25

4 2097595 exm382268 A G POLN 1 1

4 2160884 exm382298 A G POLN 3 14

4 2172456 exm382312 G A POLN 2 3

4 2200386 exm382362 A C POLN 7 14

4 2200402 exm382363 A G POLN 15 35

4 2209884 exm382375 A G POLN 2 6

4 2209899 exm382376 A C POLN 2 6

4 2210065 exm382380 A C POLN 20 37

4 2230858 exm382394 G A POLN 4 6

4 2306583 exm382589 A G ZFYVE28 15 33

4 2306662 exm382597 G C ZFYVE28 0 5

4 2632804 exm382749 C G FAM193A 12 23

4 2632805 exm382750 A G FAM193A 7 13

4 2673963 exm382814 A G FAM193A 12 27

4 2702034 exm382924 A G FAM193A 11 28

4 2746491 exm382979 G A TNIP2 11 19

4 2747198 exm382996 A G TNIP2 0 2

4 2828992 exm383060 A G SH3BP2 1 0

4 2831415 exm383085 C G SH3BP2 15 30

4 2941045 exm383413 A T NOP14 16 34

4 2941327 exm383423 A G NOP14 9 14

4 2949274 exm383468 C G NOP14 19 36

4 2964988 exm383544 A G NOP14 12 30

4 3040146 exm383640 C G GRK4 7 12

4 3148570 exm383798 A G HTT 7 12

4 3148653 exm383803 C A HTT 1 3

4 3189391 exm383869 A C HTT 2 4

4 3318339 exm384139 G A RGS12 3 6

4 3318768 exm384166 A G RGS12 7 18

4 3319366 exm384211 G A RGS12 2 4

4 3344760 exm384240 A G RGS12 2 7

4 3444477 exm384400 G C HGFAC 21 22

4 3444510 exm384403 G A HGFAC 1 3

4 3494494 exm384638 A G DOK7 1 0

4 3494617 exm384658 C G DOK7 7 21

4 3494971 exm384702 G C DOK7 1 0

4 4303927 exm385123 G C ZBTB49 4 12

4 4304599 exm385152 A G ZBTB49 14 18

4 5586510 exm385591 A G EVC2 3 9

4 5627593 exm385665 C G EVC2 0 1

4 5633725 exm385691 C A EVC2 2 9

4 5693048 exm385782 G A EVC2 5 10

4 5733236 exm385815 G C EVC 0 2

4 5733299 exm385820 A G EVC 8 16

4 5733336 exm385824 A C EVC 3 10

4 5754591 exm385874 A G EVC 6 13

4 5758026 exm385902 A G EVC 9 25

4 5785351 exm385915 G A EVC 10 24

4 5811263 exm385987 G A EVC 6 15

4 5812152 exm385996 A G EVC 9 17

4 6290751 exm386346 C A WFS1 16 33

4 6296909 exm386402 A G WFS1 12 29

4 6303301 exm386530 C G WFS1 4 15

4 6303878 exm386612 A G WFS1 12 25

4 6304118 exm386641 A G WFS1 12 17

4 6588866 exm386778 A G MAN2B2 14 13

4 6612710 exm386910 G A MAN2B2 14 30

4 6612981 exm386927 A G MAN2B2 7 11

4 6619152 exm386947 A G MAN2B2 0 1

4 6863215 exm387106 A G KIAA0232 9 15

4 6863742 exm387123 G A KIAA0232 11 12

4 6864544 exm387145 G A KIAA0232 6 21

4 6865507 exm387182 A G KIAA0232 6 21

4 6865797 exm387190 C A KIAA0232 3 7

4 7062677 exm387421 A G GRPEL1 15 18

4 7062894 exm387431 A G GRPEL1 11 24

4 7435886 exm387532 A G PSAPL1 5 7

4 7435943 exm387537 A G PSAPL1 1 1

4 7684579 exm387636 A C SORCS2 3 6

4 7691291 exm387642 A G SORCS2 10 21

4 7719795 exm387684 G A SORCS2 5 15

4 8214511 exm388129 A G SH3TC1 2 13

4 8218870 exm388175 A G SH3TC1 1 2

4 8372713 exm388571 A G ACOX3 0 1

4 8416586 exm388687 C A ACOX3 9 10

4 8416589 exm388689 G A ACOX3 5 14

4 8417620 exm388699 A G ACOX3 6 16

4 8418148 exm388717 C A ACOX3 9 10

4 8609123 exm389069 G A CPZ 2 6

4 8616217 exm389115 G C CPZ 0 1

4 8621112 exm389147 A G CPZ 1 13

4 9922113 exm389449 A G SLC2A9 2 4

4 9922128 exm389451 A G SLC2A9 11 27

4 10022947 exm389512 A G SLC2A9 9 18

4 10089539 exm389576 G A WDR1 8 6

4 10445794 exm389665 A C ZNF518B 16 35

4 10527472 exm389790 G A CLNK 15 37

4 10567765 exm389822 C A CLNK 18 34

4 13601329 exm390063 A G BOD1L1 11 15

4 13601341 exm390067 A G BOD1L1 8 10

4 13603038 exm390136 A G BOD1L1 22 43

4 13603591 exm390150 A G BOD1L1 29 53

4 13604034 exm390174 G A BOD1L1 2 7

4 13605040 exm390211 A G BOD1L1 3 4

4 13605618 exm390235 G C BOD1L1 0 3

4 15512942 exm390481 A G CC2D2A 0 5

4 15529082 exm390505 A G CC2D2A 3 4

4 15562176 exm390567 A G CC2D2A 2 3

4 15565120 exm390579 G A CC2D2A 3 6

4 15569097 exm390584 C G CC2D2A 2 2

4 15572102 exm390600 G A CC2D2A 2 3

4 15599051 exm390614 A G CC2D2A 7 26

4 15603037 exm390634 A G CC2D2A 1 7

4 15707208 exm390792 A G BST1 7 20

4 15709192 exm390801 A G BST1 11 12

4 15937670 exm390903 A G FGFBP1 6 11

4 15937916 exm390917 G A FGFBP1 1 2

4 15964134 exm390945 C A FGFBP2 2 6

4 15992916 exm391018 G A PROM1 4 7

4 15994002 exm391023 C A PROM1 15 42

4 16020034 exm391060 A G PROM1 5 8

4 16026841 exm391082 C G PROM1 12 45

4 16026921 exm391085 G A PROM1 0 1

4 16077427 exm391109 C G PROM1 5 12

4 16077475 exm391111 C A PROM1 0 3

4 17583923 exm391320 A G LAP3 3 7

4 17586746 exm391342 A G LAP3 19 24

4 17609080 exm391378 C G LAP3 10 16

4 17640880 exm391449 G C FAM184B 1 0

4 17706664 exm391489 A G FAM184B 1 2

4 17707449 exm391493 A G FAM184B 19 31

4 17827108 exm391596 C A NCAPG 15 17

4 17839273 exm391631 G A NCAPG 15 25

4 17843948 exm391657 A G NCAPG 7 12

4 17886146 exm391685 G A LCORL 7 10

4 20259514 exm391721 A G SLIT2 5 14

4 20490488 exm391742 A G SLIT2 8 11

4 20620616 exm391923 A G SLIT2 8 27

4 20715151 exm391970 G C PACRGL 14 30

4 22389468 exm392033 C A ADGRA3 2 12

4 22389707 exm392052 G C ADGRA3 12 21

4 22389867 exm392062 A T ADGRA3 3 8

4 22390043 exm392069 A G ADGRA3 5 13

4 22394175 exm392095 G A ADGRA3 12 26

4 22394239 exm392099 C A ADGRA3 11 27

4 22425837 exm392143 A G ADGRA3 16 19

4 22449130 exm392196 A C ADGRA3 12 21

4 24833212 exm392553 G C CCDC149 9 15

4 25028529 exm392657 A G LGI2 10 23

4 25664344 exm392986 A G SLC34A2 2 0

4 25664381 exm392991 A G SLC34A2 4 9

4 25673269 exm393030 A G SLC34A2 8 13

4 25750082 exm393098 A G SEL1L3 11 23

4 25769216 exm393122 G A SEL1L3 1 4

4 25819793 exm393164 G A SEL1L3 2 5

4 25823623 exm393179 A G SEL1L3 3 9

4 25831756 exm393185 A G SEL1L3 2 6

4 25835100 exm393193 A G SEL1L3 1 2

4 25849141 exm393211 A C SEL1L3 13 19

4 26487361 exm393359 A G CCKAR 1 7

4 26487431 exm393363 G A CCKAR 3 6

4 31144386 exm393739 G A PCDH7 2 7

4 36069552 exm393748 A C ARAP2 2 4

4 36093639 exm393790 A G ARAP2 0 1

4 36160441 exm393875 T A ARAP2 3 15

4 36230585 exm393971 G A ARAP2 3 7

4 36286015 exm393998 C A DTHD1 3 2

4 36340755 exm394040 A C DTHD1 7 16

4 37591794 exm394153 T A C4orf19 4 10

4 37591966 exm394159 A G C4orf19 6 20

4 37592249 exm394174 A T C4orf19 5 21

4 37636609 exm394208 A G RELL1 2 7

4 37831719 exm394249 A G PGM2 4 6

4 37841767 exm394270 A G PGM2 6 8

4 37850188 exm394305 A G PGM2 1 6

4 38016517 exm394401 G A TBC1D1 4 5

4 38019979 exm394408 A G TBC1D1 4 8

4 38774811 exm394600 G A TLR10 0 1

4 38774928 exm394608 A G TLR10 11 28

4 38775794 exm394642 G A TLR10 2 5

4 38775900 exm394646 G A TLR10 3 7

4 38799449 exm394785 G C TLR1 21 27

4 38800214 exm394822 C G TLR1 0 1

4 38828757 exm394837 A G TLR6 1 6

4 38828828 exm394842 A G TLR6 19 26

4 38829815 exm394881 G A TLR6 1 0

4 38830331 exm394905 A G TLR6 8 19

4 38830629 exm394924 G A TLR6 1 0

4 38879974 exm394955 A G FAM114A1 10 15

4 38933114 exm395011 A G FAM114A1 14 30

4 38995413 exm395064 A G TMEM156 3 6

4 39000322 exm395076 A G TMEM156 2 5

4 39033851 exm395083 A G TMEM156 14 31

4 39077757 exm395121 A G KLHL5 4 10

4 39083586 exm395127 A G KLHL5 7 13

4 39217529 exm395228 C G WDR19 1 3

4 39230243 exm395261 G A WDR19 12 29

4 39241898 exm395280 A G WDR19 8 28

4 39247045 exm395294 G A WDR19 17 37

4 39269621 exm395311 C A WDR19 4 6

4 39408699 exm395515 A G KLB 2 10

4 39408735 exm395518 A G KLB 3 3

4 39439368 exm395558 A G KLB 5 5

4 40104096 exm395912 G A N4BP2 6 4

4 40122313 exm395982 T A N4BP2 12 16

4 40122511 exm395988 C A N4BP2 23 34

4 40122771 exm395994 G A N4BP2 0 1

4 40146415 exm396071 G C N4BP2 4 7

4 40752736 exm396285 A T NSUN7 13 25

4 40763307 exm396304 A C NSUN7 9 11

4 40800878 exm396334 A G NSUN7 4 3

4 40818179 exm396361 G A APBB2 1 4

4 40946884 exm396401 A G APBB2 16 37

4 41615585 exm396533 A G LIMCH1 12 35

4 41663522 exm396608 A G LIMCH1 18 31

4 41664903 exm396617 G A LIMCH1 16 18

4 41686520 exm396655 A G LIMCH1 2 3

4 42457348 exm396981 G A ATP8A1 8 17

4 42524247 exm397011 G A ATP8A1 2 6

4 42895357 exm397090 A G GRXCR1 9 6

4 43032458 exm397113 A G GRXCR1 4 5

4 44682716 exm397221 G A GUF1 8 31

4 44685328 exm397237 G C GUF1 8 26

4 46043025 exm397332 A G GABRG1 1 5

4 47033936 exm397487 G A GABRB1 4 4

4 47427887 exm397537 G A GABRB1 1 0

4 47537994 exm397618 A G ATP10D 11 28

4 47570981 exm397713 A G ATP10D 4 9

4 47578863 exm397735 G A ATP10D 4 11

4 47628482 exm397819 A G CORIN 1 2

4 47679958 exm397882 A G CORIN 8 18

4 47682209 exm397892 A G CORIN 13 36

4 47788772 exm397930 A G CORIN 10 15

4 47880553 exm397973 A G NFXL1 6 11

4 47887633 exm397990 A G NFXL1 21 42

4 47905327 exm398029 A G NFXL1 10 23

4 47907352 exm398033 A G NFXL1 1 3

4 48027333 exm398141 G A NIPAL1 0 2

4 48037777 exm398158 A G NIPAL1 3 8

4 48038098 exm398174 G A NIPAL1 4 7

4 48502177 exm398494 A G FRYL 4 5

4 48559502 exm398650 A G FRYL 16 34

4 48583595 exm398736 A C FRYL 11 15

4 48622707 exm398772 A G FRYL 0 1

4 48853824 exm398808 C G OCIAD1 2 4

4 48862754 exm398828 G A OCIAD1 7 21

4 52729610 exm398958 C G DCUN1D4 4 4

4 52729855 exm398962 A C DCUN1D4 0 6

4 52860884 exm398999 C A LRRC66 0 3

4 52861308 exm399018 A G LRRC66 1 0

4 52869526 exm399068 A G LRRC66 19 28

4 52883563 exm399079 G A LRRC66 8 14

4 52938115 exm399159 A G SPATA18 0 1

4 52943024 exm399174 A G SPATA18 11 12

4 54011587 exm399383 G A SCFD2 1 1

4 54218834 exm399417 A G SCFD2 18 24

4 55127448 exm399751 A G PDGFRA 6 11

4 55136800 exm399793 G C PDGFRA 11 19

4 55564615 exm399927 A G KIT 1 3

4 55575673 exm399952 G A KIT 8 20

4 55593431 exm399963 A G KIT 5 5

4 55946112 exm400022 G A KDR 1 2

4 55946191 exm400027 A T KDR 13 29

4 55964925 exm400104 A G KDR 4 8

4 55976857 exm400166 A G KDR 7 13

4 55981047 exm400187 A G KDR 1 2

4 55984813 exm400197 A G KDR 2 2

4 55984931 exm400205 A T KDR 2 1

4 56308687 exm400289 A G CLOCK 1 1

4 56325147 exm400330 A G CLOCK 5 18

4 56325365 exm400335 C G CLOCK 2 3

4 56818300 exm400511 G A CEP135 1 6

4 56818360 exm400512 A G CEP135 5 16

4 56820412 exm400521 A G CEP135 6 23

4 56825871 exm400535 G A CEP135 16 35

4 56837458 exm400557 G A CEP135 10 15

4 56865523 exm400597 G A CEP135 13 22

4 56884001 exm400629 A G CEP135 14 23

4 57164405 exm400653 A G KIAA1211 3 6

4 57173854 exm400670 A G KIAA1211 1 3

4 57176832 exm400671 G A KIAA1211 1 3

4 57180701 exm400714 G A KIAA1211 30 57

4 57180968 exm400726 G C KIAA1211 2 2

4 57181833 exm400766 G A KIAA1211 15 30

4 57182709 exm400803 A G KIAA1211 15 32

4 57189623 exm400814 G A KIAA1211 4 17

4 57215803 exm400901 A G AASDH 1 3

4 57215969 exm400906 A G AASDH 0 1

4 57216026 exm400914 A G AASDH 6 15

4 57244449 exm400972 G A AASDH 7 7

4 57244512 exm400984 A T AASDH 9 11

4 57314612 exm401086 C G PAICS 21 24

4 57797879 exm401405 G A REST 7 15

4 62801682 exm401690 A G ADGRL3 7 22

4 65180381 exm401788 A G TECRL 14 31

4 68340623 exm401975 A G CENPC 7 10

4 68358623 exm401983 G A CENPC 10 19

4 68359617 exm401987 G C CENPC 20 30

4 68380070 exm402033 A G CENPC 17 40

4 68385211 exm402058 G A CENPC 10 19

4 68405599 exm402068 A G CENPC 27 36

4 68484738 exm402130 G A UBA6 0 2

4 68504780 exm402185 A T UBA6 9 15

4 68930453 exm402487 A G TMPRSS11F 11 32

4 68964752 exm402533 A G TMPRSS11F 1 2

4 69093763 exm402556 A G TMPRSS11B 11 13

4 69095169 exm402581 A G TMPRSS11B 3 3

4 69962449 exm403199 A C UGT2B7 0 1

4 70359421 exm403527 G C UGT2B4 5 12

4 70460939 exm403606 C G UGT2A1;UGT2A2 3 3

4 70460940 exm403607 A C UGT2A1;UGT2A2 2 4

4 70504751 exm403631 A G UGT2A1;UGT2A2 7 22

4 70504823 exm403633 C G UGT2A1;UGT2A2 6 24

4 70505036 exm403640 A G UGT2A1;UGT2A2 15 39

4 70513163 exm403687 A C UGT2A1 1 4

4 70596225 exm403710 G C SULT1B1 14 16

4 70599222 exm403720 A C SULT1B1 1 1

4 70803539 exm403803 A G CSN1S1 6 23

4 70810605 exm403820 G A CSN1S1 5 15

4 71063773 exm404008 A G ODAM 16 29

4 71114913 exm404074 A G CSN3 9 28

4 71114914 exm404075 A G CSN3 7 15

4 71275480 exm404207 A C OPRPN 11 21

4 71275759 exm404223 C A OPRPN 0 2

4 71472001 exm404404 A G AMBN 5 16

4 71472053 exm404407 C A AMBN 10 12

4 71509314 exm404534 A G ENAM 0 2

4 71510549 exm404587 C G ENAM 3 7

4 71555623 exm404666 A G UTP3 20 28

4 72620789 exm405032 A G GC 3 4

4 72634084 exm405078 A C GC 0 1

4 72897770 exm405089 A G NPFFR2 3 4

4 72994525 exm405114 G A NPFFR2 15 36

4 73003816 exm405124 A G NPFFR2 11 18

4 73013366 exm405162 A G NPFFR2 3 4

4 73013389 exm405163 T A NPFFR2 3 4

4 73188784 exm405285 A G ADAMTS3 10 22

4 74279351 exm405646 G A ALB 3 14

4 74280852 exm405649 A C ALB 0 5

4 74281976 exm405651 A G ALB 1 0

4 74316394 exm405758 A G AFP 3 9

4 74318329 exm405785 A G AFP 18 29

4 74347532 exm405800 C A AFM 5 12

4 74354474 exm405841 T A AFM 2 2

4 74442178 exm405882 C A RASSF6 4 11

4 74451031 exm405907 A G RASSF6 4 13

4 74459184 exm405923 A G RASSF6 5 12

4 74464386 exm405931 A C RASSF6 6 13

4 74477505 exm405938 G A RASSF6 23 36

4 75681135 exm406311 C A BTC 6 12

4 75695286 exm406316 A G BTC 14 12

4 75695345 exm406318 G A BTC 1 6

4 75937800 exm406336 A G PARM1 13 29

4 76529013 exm406518 G A CDKL2 5 4

4 76529038 exm406520 G A CDKL2 17 31

4 76782056 exm406682 A G PPEF2 11 17

4 76787346 exm406690 A G PPEF2 9 15

4 76787439 exm406695 A C PPEF2 5 23

4 76794311 exm406713 A G PPEF2 1 8

4 76797652 exm406740 G A PPEF2 4 3

4 76809396 exm406781 A G PPEF2 15 40

4 76811249 exm406795 C A PPEF2 6 25

4 76813036 exm406807 A T PPEF2 4 12

4 76878548 exm406913 A C SDAD1 0 4

4 77184996 exm407298 A G FAM47E;FAM47E-STBD1 5 27

4 77230359 exm407330 A G STBD1 3 7

4 77274368 exm407429 G A CCDC158 3 6

4 77283381 exm407440 A G CCDC158 2 4

4 77288866 exm407455 G C CCDC158 1 2

4 77288874 exm407456 A C CCDC158 9 29

4 77290670 exm407467 A G CCDC158 13 34

4 77305566 exm407515 A G CCDC158 15 43

4 77357273 exm407534 A G SHROOM3 1 2

4 77476909 exm407548 C G SHROOM3 5 12

4 77651958 exm407555 A G SHROOM3 3 7

4 77660427 exm407594 C A SHROOM3 8 26

4 77661017 exm407628 C A SHROOM3 8 13

4 77661704 exm407664 C A SHROOM3 11 33

4 77675925 exm407743 A G SHROOM3 6 9

4 77817325 exm407847 A G SOWAHB 8 23

4 77817933 exm407872 A G SOWAHB 1 2

4 78804493 exm408120 A G MRPL1 9 25

4 78804530 exm408124 C G MRPL1 2 8

4 79186260 exm408207 A G FRAS1 1 4

4 79239985 exm408267 A G FRAS1 1 6

4 79293958 exm408308 A G FRAS1 12 24

4 79387495 exm408509 A G FRAS1 2 9

4 79394691 exm408534 G A FRAS1 6 13

4 79400644 exm408563 A G FRAS1 8 31

4 79442730 exm408689 G A FRAS1 16 30

4 79458330 exm408713 A C FRAS1 8 16

4 79460455 exm408715 G A FRAS1 20 36

4 79462135 exm408747 A G FRAS1 4 10

4 79494382 exm408759 A G ANXA3 15 28

4 79522685 exm408796 A G ANXA3 12 18

4 79525504 exm408803 A G ANXA3 7 25

4 79531235 exm408810 G A ANXA3 7 2

4 79772190 exm408845 A G BMP2K 12 26

4 79786856 exm408859 A G BMP2K 12 25

4 79793944 exm408898 C A BMP2K 5 6

4 79793949 exm408899 G A BMP2K 0 1

4 79832195 exm408933 C G BMP2K 19 36

4 80327944 exm409036 A G GK2 0 1

4 80328063 exm409048 G A GK2 1 3

4 81207708 exm409293 A G FGF5 1 1

4 81207749 exm409296 G A FGF5 3 4

4 82362055 exm409510 G A RASGEF1B 2 8

4 82380597 exm409561 A C RASGEF1B 0 5

4 83740367 exm409773 G A SEC31A 2 1

4 83782827 exm409871 A T SEC31A 7 17

4 84227441 exm410229 A G HPSE 5 8

4 84347155 exm410312 G A HELQ 7 19

4 84353354 exm410336 C A HELQ 1 10

4 84369985 exm410378 A G HELQ 22 40

4 84374630 exm410391 G A HELQ 5 14

4 84374746 exm410399 A G HELQ 6 11

4 84374966 exm410405 A G HELQ 5 14

4 84383935 exm410470 G A ABRAXAS1 4 12

4 85538744 exm410616 A C CDS1 2 6

4 85569724 exm410643 A G CDS1 16 25

4 85638153 exm410735 G A WDFY3 0 1

4 85699687 exm410823 A G WDFY3 0 1

4 85717696 exm410859 C G WDFY3 4 18

4 85724595 exm410875 A G WDFY3 10 23

4 85738576 exm410894 A G WDFY3 0 8

4 85738655 exm410897 C A WDFY3 4 7

4 85742624 exm410913 G A WDFY3 14 30

4 86915883 exm411021 G A ARHGAP24 4 6

4 86916225 exm411035 A C ARHGAP24 1 2

4 87610221 exm411138 A G PTPN13 2 3

4 87622723 exm411166 A G PTPN13 10 22

4 87666269 exm411230 A C PTPN13 2 10

4 87671686 exm411233 A G PTPN13 2 2

4 87692393 exm411304 A G PTPN13 23 48

4 87730949 exm411379 G C PTPN13 0 3

4 87968165 exm411498 A C AFF1 6 10

4 87968375 exm411512 A G AFF1 2 6

4 87968514 exm411519 A G AFF1 4 5

4 87968736 exm411529 C A AFF1 1 6

4 88116591 exm411745 A C KLHL8 1 0

4 88414804 exm411926 G A SPARCL1 13 18

4 88414970 exm411934 A G SPARCL1 0 1

4 88415396 exm411950 G A SPARCL1 1 4

4 88415566 exm411957 C G SPARCL1 12 35

4 88533290 exm411978 A G DSPP 5 3

4 88534326 exm412028 A G DSPP 12 24

4 88537715 exm412356 A C DSPP 5 15

4 88583351 exm412384 A G DMP1 3 5

4 88583654 exm412400 G A DMP1 1 7

4 88723842 exm412452 A G IBSP 16 31

4 88732918 exm412483 A T IBSP 36 68

4 88767006 exm412542 A G MEPE 22 35

4 88967919 exm412686 C A PKD2 9 27

4 88989102 exm412740 A G PKD2 10 25

4 88996805 exm412765 A G PKD2 0 1

4 89018628 exm412786 G A ABCG2 3 6

4 89338691 exm412998 G A HERC6 14 27

4 89583660 exm413224 A G HERC3 8 15

4 89602427 exm413271 A C HERC3 13 30

4 89680051 exm413425 A G FAM13A 0 3

4 89772246 exm413464 A G FAM13A 25 58

4 89912172 exm413479 A G FAM13A 12 33

4 90035221 exm413539 G A TIGD2 0 2

4 90035697 exm413555 C A TIGD2 10 12

4 90170118 exm413631 G A GPRIN3 19 42

4 90816463 exm413743 A T MMRN1 6 14

4 90856383 exm413802 A G MMRN1 5 7

4 90856847 exm413819 G A MMRN1 3 8

4 90857320 exm413839 A G MMRN1 2 9

4 91230693 exm413955 A G CCSER1 1 4

4 94031946 exm414039 G A GRID2 5 12

4 94547444 exm414101 A G GRID2 25 38

4 95129699 exm414175 A G SMARCAD1 17 30

4 95155148 exm414180 G A SMARCAD1 8 13

4 95376504 exm414298 G A PDLIM5 22 37

4 95539204 exm414360 A G PDLIM5 11 18

4 96045029 exm414424 A G BMPR1B 24 48

4 96046210 exm414430 G A BMPR1B 6 10

4 96091414 exm414489 A G UNC5C 11 27

4 96140365 exm414554 G A UNC5C 3 0

4 96761323 exm414621 A G PDHA2 6 19

4 96761324 exm414622 A G PDHA2 7 20

4 96761547 exm414632 A C PDHA2 6 19

4 96762370 exm414680 A G PDHA2 30 35

4 100239284 exm415185 T A ADH1B 10 13

4 100336690 exm415262 A G ADH7 5 13

4 100350696 exm415313 A G ADH7 14 20

4 100470310 exm415385 A C TRMT10A 8 15

4 100472128 exm415399 A G TRMT10A 3 7

4 100478524 exm415408 A G TRMT10A 1 4

4 100503146 exm415433 A G MTTP 1 2

4 100532606 exm415530 C G MTTP 1 4

4 102751014 exm415820 C G BANK1 4 21

4 102751045 exm415825 A G BANK1 7 16

4 102783776 exm415852 A G BANK1 1 3

4 102791797 exm415859 A C BANK1 4 6

4 102994851 exm415936 A G BANK1 3 8

4 103522114 exm416065 A G NFKB1 16 33

4 103556180 exm416156 A G MANBA 8 15

4 103595227 exm416214 A C MANBA 7 18

4 103968223 exm416403 G A SLC9B2 5 8

4 103970062 exm416417 A C SLC9B2 3 7

4 103971482 exm416423 G A SLC9B2 8 14

4 103971500 exm416426 G A SLC9B2 8 23

4 103979051 exm416435 A G SLC9B2 12 31

4 104041412 exm416544 A C CENPE 5 9

4 104044243 exm416551 G A CENPE 8 12

4 104055001 exm416565 G A CENPE 7 5

4 104060947 exm416577 C G CENPE 8 10

4 104062206 exm416602 G A CENPE 3 9

4 104066814 exm416633 G A CENPE 5 9

4 104067125 exm416640 A G CENPE 7 5

4 104070095 exm416659 C G CENPE 23 37

4 104117108 exm416778 C G CENPE 6 18

4 104117353 exm416790 A G CENPE 5 13

4 106155533 exm416904 A G TET2 12 25

4 106156163 exm416927 A G TET2 4 6

4 106156187 exm416928 A G TET2 7 22

4 106157398 exm416961 G A TET2 15 29

4 106157698 exm416973 G A TET2 1 5

4 106196834 exm417041 A G TET2 1 5

4 106819144 exm417317 C G NPNT 13 34

4 106859520 exm417331 A G NPNT 3 7

4 106880251 exm417379 G A NPNT 6 19

4 107156462 exm417477 A G TBCK 32 58

4 107165912 exm417497 G A TBCK 14 30

4 107181616 exm417525 A G TBCK 9 13

4 107216291 exm417527 A G TBCK 6 6

4 107249244 exm417546 G C AIMP1 1 4

4 107847043 exm417617 C G DKK2 2 1

4 108552932 exm417645 G C PAPSS1 17 21

4 108575955 exm417672 A G PAPSS1 27 57

4 109084731 exm417903 A G LEF1 9 16

4 110394162 exm418184 A G SEC24B 16 37

4 110415840 exm418206 A G SEC24B 1 1

4 110441604 exm418241 C A SEC24B 9 12

4 110673648 exm418459 G A CFI 6 15

4 110749236 exm418573 A G RRH 11 34

4 110757188 exm418597 A C RRH 10 20

4 110758625 exm418605 A G RRH 0 1

4 110763725 exm418615 C G RRH 8 12

4 110772701 exm418632 A G LRIT3 2 9

4 110772947 exm418639 A T LRIT3 12 26

4 110789102 exm418656 C G LRIT3 0 1

4 110791347 exm418695 A G LRIT3 11 33

4 110882053 exm418767 A G EGF 7 16

4 110884383 exm418782 A C EGF 12 25

4 110902111 exm418827 A T EGF 15 30

4 110909805 exm418843 A G EGF 7 14

4 111434571 exm418965 A C ENPEP 13 28

4 111482692 exm419035 G A ENPEP 4 8

4 113350387 exm419226 A C ALPK1 23 36

4 113353156 exm419294 A G ALPK1 3 13

4 113482146 exm419457 A G ZGRF1 32 49

4 113524723 exm419512 A G ZGRF1 26 40

4 113540422 exm419600 G A ZGRF1 10 22

4 113541267 exm419615 G A ZGRF1 5 10

4 113565835 exm419629 A G LARP7 25 43

4 113568359 exm419654 G C LARP7 0 1

4 113568544 exm419663 A G LARP7 0 3

4 114239712 exm419872 A G ANK2 4 6

4 114278753 exm420138 G A ANK2 0 1

4 114279697 exm420189 C G ANK2 1 1

4 114280063 exm420200 C A ANK2 4 8

4 114286315 exm420227 A C ANK2 6 5

4 114290875 exm420247 A G ANK2 1 10

4 114294471 exm420264 G A ANK2 1 4

4 115792029 exm420495 C G NDST4 13 18

4 115898352 exm420520 G A NDST4 3 6

4 118975586 exm420643 A G NDST3 3 11

4 119174666 exm420703 T A NDST3 10 20

4 119948504 exm421063 G C SYNPO2 18 45

4 119948506 exm421064 A G SYNPO2 4 10

4 119952955 exm421147 A G SYNPO2 37 62

4 120085477 exm421222 G A MYOZ2 3 8

4 120169906 exm421244 A T USP53 1 4

4 120192664 exm421297 T A USP53 7 19

4 120192717 exm421300 A C USP53 14 43

4 120192853 exm421305 G A USP53 3 7

4 120193017 exm421312 A G USP53 14 43

4 120214185 exm421340 G A USP53 5 9

4 120528342 exm421502 A G PDE5A 3 5

4 121719544 exm421587 A T PRDM5 2 6

4 122250537 exm421782 A G QRFPR 11 27

4 122250555 exm421785 G A QRFPR 3 12

4 122250603 exm421786 A G QRFPR 9 40

4 122251598 exm421796 G A QRFPR 10 8

4 122725835 exm421957 A G EXOSC9 0 1

4 123130479 exm422292 A G KIAA1109 4 8

4 123150288 exm422324 A G KIAA1109 18 34

4 123161375 exm422361 G A KIAA1109 13 27

4 123166228 exm422374 G A KIAA1109 1 3

4 123167395 exm422379 A G KIAA1109 1 0

4 123171659 exm422400 A T KIAA1109 0 2

4 123179900 exm422422 A G KIAA1109 6 10

4 123202746 exm422476 G A KIAA1109 0 7

4 123280769 exm422611 G A KIAA1109 0 1

4 123663259 exm422725 G A BBS12 15 21

4 123664512 exm422780 A G BBS12 0 2

4 123797480 exm422832 A C FGF2 22 39

4 123797526 exm422835 G C FGF2 6 7

4 123855512 exm422922 G A SPATA5 1 6

4 123855513 exm422923 G A SPATA5 2 9

4 123855642 exm422927 A C SPATA5 7 26

4 123868550 exm422953 A G SPATA5 0 2

4 125590492 exm423057 A G ANKRD50 19 24

4 125593261 exm423181 G A ANKRD50 21 38

4 126238395 exm423258 A G FAT4 0 1

4 126239421 exm423297 A G FAT4 3 4

4 126240377 exm423326 A C FAT4 11 21

4 126241528 exm423372 G A FAT4 2 7

4 126336105 exm423465 G A FAT4 22 53

4 126336849 exm423497 A G FAT4 1 0

4 126355433 exm423513 G A FAT4 13 19

4 126369952 exm423545 G A FAT4 21 39

4 126373425 exm423689 G A FAT4 4 8

4 126373570 exm423698 A G FAT4 10 19

4 126402750 exm423757 A G FAT4 1 1

4 126411388 exm423791 A G FAT4 1 4

4 126412637 exm423864 A G FAT4 10 19

4 128577820 exm423912 A G INTU 4 7

4 128626829 exm423947 T A INTU 2 8

4 128715251 exm424045 G A HSPA4L 7 20

4 128722949 exm424058 A G HSPA4L 15 37

4 128741710 exm424101 G A HSPA4L 9 19

4 128861116 exm424290 A G MFSD8 13 33

4 128951772 exm424351 A G ABHD18 1 0

4 129012661 exm424376 A G LARP1B 5 11

4 129120649 exm424436 G A LARP1B 7 20

4 129812279 exm424590 G C SCLT1 5 6

4 134072578 exm424772 A G PCDH10 18 24

4 134084225 exm424826 A C PCDH10 3 6

4 138452842 exm424984 A G PCDH18 7 18

4 140450287 exm425361 A G SETD7 1 13

4 140810546 exm425480 G C MAML3 3 8

4 140811997 exm425553 A G MAML3 26 54

4 141313429 exm425592 C A CLGN 8 10

4 141315174 exm425613 G A CLGN 0 1

4 141321589 exm425635 A G CLGN 10 21

4 141323155 exm425641 A G CLGN 0 1

4 141323200 exm425643 A T CLGN 10 20

4 141481151 exm425715 G A UCP1 1 3

4 144618496 exm426474 A T FREM3 13 16

4 144918733 exm426589 A G GYPB 14 16

4 144918755 exm426591 A C GYPB 4 4

4 146064547 exm426899 A C OTUD4 4 6

4 146073711 exm426925 G A OTUD4 3 7

4 146601588 exm427075 G A C4orf51 14 33

4 146648084 exm427078 A G C4orf51 9 17

4 147247142 exm427270 A G SLC10A7 11 14

4 147796024 exm427418 A G TTC29 2 7

4 148559696 exm427494 A G PRMT9 1 1

4 148575705 exm427548 G A PRMT9 2 6

4 148579110 exm427555 C G PRMT9 1 2

4 148579111 exm427556 A G PRMT9 3 4

4 148589774 exm427573 A G PRMT9 0 1

4 148787942 exm427650 G A ARHGAP10 9 22

4 148886191 exm427701 C A ARHGAP10 3 6

4 148968201 exm427736 G A ARHGAP10 16 22

4 148984378 exm427746 G A ARHGAP10 12 25

4 148985578 exm427753 A G ARHGAP10 5 18

4 149356352 exm427808 G A NR3C2 10 24

4 149357246 exm427840 G A NR3C2 17 34

4 151160918 exm427928 C A DCLK2 3 6

4 151199033 exm427972 A G LRBA 3 8

4 151392781 exm428060 G A LRBA 4 9

4 151765814 exm428206 A G LRBA 1 1

4 151814257 exm428317 A G LRBA 3 6

4 151935601 exm428390 G A LRBA 12 19

4 152065202 exm428474 A G SH3D19 2 10

4 152570638 exm428620 A G FAM160A1 8 23

4 152629221 exm428709 A G GATB 12 30

4 152638153 exm428723 A C GATB 10 9

4 152638163 exm428724 A G GATB 10 12

4 152638226 exm428727 A C GATB 10 21

4 152679964 exm428736 G A GATB 0 4

4 153273707 exm428798 A G FBXW7 9 21

4 153864453 exm428996 G A FHDC1 4 12

4 153864520 exm429003 A G FHDC1 13 24

4 153897567 exm429159 C A FHDC1 0 1

4 154502566 exm429346 G A TMEM131L 4 12

4 154506707 exm429375 A G TMEM131L 6 9

4 154523749 exm429435 A G TMEM131L 3 10

4 154557616 exm429540 A G TMEM131L 7 18

4 154624136 exm429547 A G TLR2 6 8

4 154624316 exm429556 G A TLR2 3 2

4 154625965 exm429636 C A TLR2 1 5

4 154631550 exm429662 A G RNF175 12 28

4 154631587 exm429664 C G RNF175 21 51

4 154644537 exm429692 G A RNF175 27 55

4 154669859 exm429703 A G RNF175 7 22

4 154669906 exm429708 A G RNF175 0 1

4 155156674 exm429801 A G DCHS2 14 30

4 155157646 exm429835 A G DCHS2 10 27

4 155158081 exm429862 A G DCHS2 3 5

4 155176722 exm429896 A G DCHS2 7 19

4 155176782 exm429899 T A DCHS2 0 1

4 155219257 exm429921 G A DCHS2 2 9

4 155219338 exm429931 G A DCHS2 13 20

4 155219549 exm429944 C A DCHS2 8 9

4 155225953 exm429973 A C DCHS2 0 2

4 155226021 exm429975 A G DCHS2 24 33

4 155241735 exm429995 A G DCHS2 8 9

4 155243574 exm430027 A G DCHS2 0 1

4 155253964 exm430071 A G DCHS2 7 18

4 155254051 exm430074 A G DCHS2 8 20

4 155254146 exm430078 A C DCHS2 9 31

4 155254355 exm430091 A C DCHS2 3 12

4 155256125 exm430117 G A DCHS2 12 16

4 155505592 exm430382 A G FGA 0 1

4 155505692 exm430386 C G FGA 13 18

4 155507215 exm430414 G A FGA 19 41

4 155665881 exm430556 A C LRAT 4 10

4 155670206 exm430562 A G LRAT 1 0

4 155719287 exm430581 G A RBM46 10 21

4 156281375 exm430711 G A MAP9 0 2

4 156281490 exm430718 A C MAP9 8 9

4 156764844 exm430990 A G ASIC5 5 9

4 156764932 exm430997 C G ASIC5 4 11

4 156784660 exm431021 G A ASIC5 10 22

4 156841062 exm431103 A T TDO2 3 12

4 159091422 exm431374 A T GASK1B 4 9

4 159091864 exm431392 A G GASK1B 2 9

4 159092523 exm431423 A G GASK1B 5 7

4 159638271 exm431682 G A PPID 3 7

4 159638346 exm431688 A G PPID 7 10

4 159790198 exm431805 A G FNIP2 2 5

4 159790261 exm431812 A G FNIP2 1 1

4 159894311 exm431864 G A C4orf45 1 3

4 159894353 exm431868 A G C4orf45 4 14

4 160277263 exm432023 A G RAPGEF2 3 6

4 164394321 exm432352 A C TKTL2 7 16

4 164394333 exm432353 G A TKTL2 13 18

4 164394616 exm432373 G C TKTL2 6 12

4 164394765 exm432378 A C TKTL2 4 4

4 164534644 exm432454 A G MARCHF1 5 14

4 165118820 exm432498 A G ANP32C 9 16

4 165961828 exm432568 A C TRIM60 17 26

4 165962462 exm432596 A C TRIM60 4 15

4 166261514 exm432761 G A MSMO1 5 5

4 166388933 exm432798 A G CPE 3 11

4 166414347 exm432827 A G CPE 21 42

4 166976336 exm432907 G A TLL1 7 15

4 169100440 exm433075 G A ANXA10 11 20

4 169100461 exm433077 G A ANXA10 4 9

4 169142960 exm433104 A G DDX60 6 11

4 169172157 exm433149 A G DDX60 20 33

4 169189058 exm433198 A G DDX60 0 1

4 169206006 exm433252 A G DDX60 1 6

4 169208334 exm433265 A G DDX60 3 5

4 169229168 exm433307 A G DDX60 12 28

4 169299588 exm433350 A G DDX60L 10 24

4 169315740 exm433379 A G DDX60L 4 10

4 169336811 exm433419 A G DDX60L 3 5

4 169343015 exm433450 A G DDX60L 12 23

4 169348404 exm433469 A G DDX60L 10 18

4 169383140 exm433527 A C DDX60L 1 2

4 169433157 exm433571 C G PALLD 16 35

4 169433386 exm433582 G A PALLD 3 7

4 169433511 exm433587 G A PALLD 4 21

4 170037611 exm433773 C A SH3RF1 19 27

4 170038932 exm433800 A G SH3RF1 2 9

4 170359351 exm433904 G A NEK1 0 5

4 170498175 exm433961 C A NEK1 10 8

4 170671813 exm434085 A T HPF1 0 2

4 170678985 exm434094 A G HPF1 0 2

4 170912549 exm434099 G A MFAP3L 9 18

4 170926905 exm434141 A C MFAP3L 0 1

4 174169359 exm434283 G A GALNT7 11 30

4 174216988 exm434304 T A GALNT7 9 24

4 175220304 exm434513 A G CEP44 16 21

4 175229856 exm434534 G A CEP44 11 30

4 175898811 exm434744 A G ADAM29 4 4

4 177049919 exm434868 G A WDR17 1 4

4 177077250 exm434947 C A WDR17 10 23

4 177084327 exm434968 A G WDR17 5 6

4 177146444 exm435130 A G ASB5 15 44

4 177650736 exm435208 T A VEGFC 0 2

4 177650824 exm435211 C G VEGFC 2 7

4 183609373 exm435505 A G TENM3 5 8

4 183713987 exm435646 C G TENM3 10 26

4 183721275 exm435706 G C TENM3 1 6

4 184182124 exm435802 A G WWC2 14 22

4 184192366 exm435861 C A WWC2 1 1

4 184210648 exm435899 A G WWC2 5 8

4 184614208 exm436141 C A TRAPPC11 7 12

4 184615117 exm436159 C A TRAPPC11 2 6

4 184930752 exm436251 A G STOX2 14 18

4 184931688 exm436284 A G STOX2 4 2

4 185678380 exm436699 A G ACSL1 9 19

4 185697709 exm436767 A G ACSL1 3 7

4 185724475 exm436799 A G ACSL1 9 16

4 186085260 exm436905 G A CFAP97 11 18

4 186231836 exm437005 G A SNX25 3 7

4 186339620 exm437220 G A UFSP2 9 24

4 186339821 exm437226 G A UFSP2 4 3

4 186380455 exm437317 C A CCDC110 12 22

4 186380477 exm437318 A G CCDC110 9 26

4 186380617 exm437326 A G CCDC110 14 24

4 186380893 exm437337 G A CCDC110 24 37

4 186381382 exm437356 G A CCDC110 10 27

4 186381392 exm437357 C G CCDC110 24 36

4 186532996 exm437468 A G SORBS2 10 15

4 186544194 exm437494 A G SORBS2 4 11

4 186544880 exm437518 C G SORBS2 5 13

4 186583365 exm437606 A G SORBS2 20 37

4 187004135 exm437674 G A TLR3 5 9

4 187075633 exm437751 A G FAM149A 8 17

4 187077200 exm437762 A T FAM149A 13 32

4 187077204 exm437764 A G FAM149A 13 29

4 187084086 exm437803 G A FAM149A 7 20

4 187086607 exm437836 A G FAM149A 0 1

4 187088147 exm437839 A G FAM149A 26 47

4 187153432 exm437956 A C KLKB1 3 9

4 187171404 exm437979 A C KLKB1 16 36

4 187172420 exm437997 C G KLKB1 17 38

4 187172422 exm437998 G A KLKB1 19 23

4 187179320 exm438060 C A KLKB1 8 24

4 187509760 exm438248 A G FAT1 1 3

4 187510072 exm438267 A G FAT1 8 12

4 187510339 exm438283 A G FAT1 5 13

4 187524176 exm438376 A G FAT1 1 2

4 187524658 exm438395 G C FAT1 2 5

4 187524714 exm438400 A G FAT1 11 21

4 187525083 exm438429 G A FAT1 1 1

4 187538239 exm438522 G A FAT1 3 15

4 187539216 exm438546 A G FAT1 1 5

4 187540622 exm438615 G A FAT1 6 17

4 187540635 exm438617 C A FAT1 0 1

4 187541379 exm438654 G A FAT1 16 28

4 187549401 exm438738 C G FAT1 2 4

4 187549473 exm438743 A G FAT1 1 12

4 187549809 exm438752 G A FAT1 12 23

4 187549905 exm438759 A G FAT1 7 11

4 187557863 exm438779 A G FAT1 3 9

4 187627765 exm438815 G A FAT1 1 1

4 187627818 exm438819 A G FAT1 5 16

4 187629232 exm438880 A G FAT1 5 7

4 187629268 exm438885 G A FAT1 0 1

4 187630099 exm438931 A G FAT1 3 11

4 189012577 exm439020 C G TRIML2 13 29

4 189012810 exm439037 A G TRIML2 0 4

4 189012901 exm439047 G A TRIML2 4 8

4 189020223 exm439068 C A TRIML2 6 27

4 189022310 exm439080 T A TRIML2 4 17

4 189022350 exm439085 A G TRIML2 10 16

4 189060998 exm439116 A G TRIML1 8 22

4 170428901 JHU_4.170428900 A G NEK1 8 17

4 3015553 JHU_4.3015552 A G GRK4 2 2

4 47538044 JHU_4.47538043 A G ATP10D 21 61

4 77660299 JHU_4.77660298 C A SHROOM3 28 67

4 70359452 kgp21028703 A G UGT2B4 10 17

4 36345134 newrs149895631 A G DTHD1 13 26

4 38776383 variant.80568 A G TLR10 1 2

4 8229684 Variant13150 D I SH3TC1 14 27

5 121488382 5:121488382-AC C A ZNF474 14 27

5 132197798 5:132197798-TC G A GDF9 7 11

5 135587517 5:135587517-CT A G TRPC7 11 19

5 138707859 5:138707859-CT A G SLC23A1 4 10

5 139744198 5:139744198-CT A G SLC4A9 5 8

5 139887564 5:139887564-AG G A ANKHD1;ANKHD1-EIF4EBP3 0 1

5 140222063 5:140222063-CA A C PCDHA8 0 1

5 140307088 5:140307088-CT A G PCDHAC1 5 9

5 140530973 5:140530973-GA A G PCDHB6 2 17

5 140579961 5:140579961-TG C A PCDHB11 10 11

5 140594923 5:140594923-GT A C PCDHB13 16 42

5 140720142 5:140720142-GA A G PCDHGA2 17 39

5 140723835 5:140723835-CT A G PCDHGA3 16 27

5 140750776 5:140750776-GA A G PCDHGB3 1 1

5 140788998 5:140788998-TC G A PCDHGB6 1 4

5 140798373 5:140798373-CT A G PCDHGB7 1 1

5 141026772 5:141026772-AC C A FCHSD1 1 2

5 141325356 5:141325356-GA A G PCDH12 0 1

5 141358186 5:141358186-CA A C RNF14 13 28

5 14716916 5:14716916-TC G A ANKH 0 1

5 149425057 5:149425057-AG G A HMGXB3 4 7

5 149429924 5:149429924-CT A G HMGXB3 0 2

5 149431516 5:149431516-GA A G HMGXB3 0 3

5 149583563 5:149583563-GA A G SLC6A7 0 1

5 150859027 5:150859027-GA A G SLC36A1 2 3

5 150945437 5:150945437-AC C A FAT2 2 1

5 154214232 5:154214232-CT A G FAXDC2 3 2

5 156456807 5:156456807-AC C A HAVCR1 5 11

5 156589656 5:156589656-GT A C FAM71B 14 25

5 156926606 5:156926606-TC G A ADAM19 10 25

5 159507663 5:159507663-CT A G PWWP2A 1 0

5 162909710 5:162909710-AC C A HMMR 10 17

5 167835612 5:167835612-CT A G WWC1 2 1

5 16794852 5:16794852-GA A G MYO10 2 1

5 168189644 5:168189644-CT A G SLIT3 2 5

5 171780979 5:171780979-GA A G SH3PXD2B 2 9

5 176070108 5:176070108-GA A G EIF4E1B 3 19

5 176072412 5:176072412-AC C A EIF4E1B 1 6

5 176523736 5:176523736-CA A C FGFR4 2 8

5 179196082 5:179196082-GA A G MAML1 1 5

5 179260185 5:179260185-CT A G SQSTM1 1 1

5 179394009 5:179394009-GA A G RNF130 1 3

5 180166899 5:180166899-GA A G OR2Y1 13 25

5 180277423 5:180277423-TC G A ZFP62 0 4

5 180277992 5:180277992-CT A G ZFP62 1 0

5 205573 5:205573-CT A G CCDC127 2 2

5 32098669 5:32098669-GA A G PDZD2 3 13

5 32230375 5:32230375-GT A C MTMR12 1 1

5 33461375 5:33461375-TG C A TARS 9 18

5 34863027 5:34863027-AC C A TTC23L 4 12

5 34908877 5:34908877-TC G A RAD1 12 27

5 34915875 5:34915875-GT A C BRIX1 4 13

5 35740343 5:35740343-AG G A SPEF2 1 4

5 36219752 5:36219752-CT A G NADK2 1 2

5 36268331 5:36268331-CT A G RANBP3L 1 7

5 37184911 5:37184911-CT A G CPLANE1 2 5

5 38337670 5:38337670-CT A G EGFLAM 8 28

5 38352349 5:38352349-CT A G EGFLAM 1 4

5 434377 5:434377-GA A G AHRR 16 22

5 43613046 5:43613046-AG G A NNT 7 23

5 49706799 5:49706799-CA A C EMB 11 21

5 54410081 5:54410081-CT A G CDC20B 15 34

5 55147451 5:55147451-CT A G IL31RA 16 45

5 55192244 5:55192244-GA A G IL31RA 1 0

5 56177582 5:56177582-TC G A MAP3K1 6 24

5 56208889 5:56208889-CT A G SETD9 8 11

5 61714858 5:61714858-AG G A IPO11 4 11

5 61876550 5:61876550-GT A C LRRC70 5 12

5 61877084 5:61877084-GA A G LRRC70 16 32

5 66461974 5:66461974-GA A G MAST4 4 7

5 66478938 5:66478938-GA A G CD180 16 35

5 68687772 5:68687772-AC C A RAD17 7 17

5 70805761 5:70805761-AG G A BDP1 1 1

5 74442775 5:74442775-CT A G ANKRD31 4 9

5 74484329 5:74484329-GA A G ANKRD31 13 16

5 74484432 5:74484432-GA A G ANKRD31 0 3

5 78384350 5:78384350-GA A G BHMT2 5 12

5 78416284 5:78416284-AG G A BHMT 3 2

5 78426859 5:78426859-GA A G BHMT 1 2

5 7867550 5:7867550-CA A C FASTKD3 1 6

5 7870962 5:7870962-GA A G MTRR 4 5

5 79032050 5:79032050-AG G A CMYA5 4 4

5 79815520 5:79815520-TC G A FAM151B 1 0

5 82807936 5:82807936-CT A G VCAN 8 10

5 82833288 5:82833288-CT A G VCAN 2 4

5 82835880 5:82835880-CT A G VCAN 2 2

5 90008115 5:90008115-TG C A ADGRV1 4 5

5 90136800 5:90136800-AG G A ADGRV1 2 14

5 94803683 5:94803683-GA A G TTC37 11 12

5 96084271 5:96084271-GA A G CAST 6 18

5 126774170 exm2085462 A C MEGF10 3 7

5 13769215 exm2087723 A C DNAH5 0 1

5 14498645 exm2090730 A C TRIO 2 5

5 148689588 exm2091661 A G AFAP1L1 0 1

5 156479402 exm2093394 G A HAVCR1 18 27

5 170725816 exm2095149 A G RANBP17 13 26

5 178294024 exm2097065 A G ZNF354B 0 1

5 180166997 exm2097919 A G OR2Y1 4 13

5 32089157 exm2098757 A G PDZD2 6 9

5 33527438 exm2098974 G C ADAMTS12 1 8

5 35955850 exm2099449 G A UGT3A1 2 4

5 42695158 exm2100776 A C GHR 10 16

5 72419676 exm2103777 A G TMEM171 4 7

5 78379457 exm2104735 G A BHMT2 1 2

5 819693 exm2105491 A G ZDHHC11 0 2

5 82837946 exm2105619 A G VCAN 1 2

5 127613625 exm2275818 A C FBN2 1 0

5 40853113 exm2276911 G A CARD6 0 1

5 140474 exm439706 A G PLEKHG4B 1 2

5 140636 exm439716 A G PLEKHG4B 18 29

5 140795 exm439724 A G PLEKHG4B 0 3

5 143239 exm439731 A G PLEKHG4B 2 9

5 155476 exm439764 A G PLEKHG4B 2 10

5 156208 exm439770 G A PLEKHG4B 23 39

5 169559 exm439906 G A PLEKHG4B 1 6

5 206013 exm440124 A G CCDC127 3 4

5 376760 exm440356 A G AHRR 12 23

5 475076 exm440608 A G SLC9A3 9 13

5 480033 exm440667 A G SLC9A3 6 5

5 619212 exm440786 C A CEP72 8 9

5 637637 exm440842 G A CEP72 0 1

5 639231 exm440864 A C CEP72 26 46

5 647988 exm440908 A G CEP72 5 4

5 819671 exm440986 A G ZDHHC11 6 15

5 819698 exm440992 A C ZDHHC11 8 11

5 825329 exm441004 T A ZDHHC11 1 0

5 850518 exm441068 A G ZDHHC11 0 2

5 1065583 exm441485 A G SLC12A7 7 11

5 1216767 exm441764 A G SLC6A19 0 1

5 1232393 exm441856 A G SLC6A18 6 20

5 1235736 exm441887 A G SLC6A18 0 2

5 1325874 exm442212 A G CLPTM1L 4 1

5 1338078 exm442242 A G CLPTM1L 36 64

5 1401064 exm442284 G A SLC6A3 1 0

5 1414844 exm442329 G A SLC6A3 0 1

5 1463848 exm442404 A G LPCAT1 1 2

5 1463927 exm442407 G A LPCAT1 0 1

5 5209210 exm442885 A G ADAMTS16 1 1

5 5242229 exm442946 A G ADAMTS16 6 11

5 5460774 exm443081 A G ICE1 12 20

5 5460995 exm443085 A C ICE1 6 19

5 5461552 exm443109 A C ICE1 9 17

5 5462494 exm443144 C A ICE1 16 32

5 5462538 exm443148 A C ICE1 6 19

5 5462730 exm443157 C G ICE1 2 7

5 5463410 exm443181 C A ICE1 4 7

5 5464533 exm443236 A C ICE1 18 33

5 5464962 exm443253 G A ICE1 4 14

5 5465316 exm443259 G A ICE1 2 4

5 7757647 exm443662 G C ADCY2 3 2

5 7757697 exm443665 C A ADCY2 7 19

5 7832048 exm443735 A C C5orf49 5 16

5 7867353 exm443828 G A FASTKD3 1 5

5 7868034 exm443857 A C FASTKD3 1 4

5 7878101 exm443907 A G MTRR 1 9

5 7878424 exm443932 T A MTRR 1 3

5 7883344 exm443936 A G MTRR 10 23

5 7893013 exm443975 A G MTRR 9 20

5 9044606 exm444024 A G SEMA5A 0 1

5 9063124 exm444067 A G SEMA5A 0 2

5 9122898 exm444109 A G SEMA5A 1 1

5 9190574 exm444146 A G SEMA5A 5 14

5 9197387 exm444159 A G SEMA5A 2 6

5 9202121 exm444166 A G SEMA5A 17 35

5 10236693 exm444274 G A ATPSCKMT 0 3

5 10239233 exm444282 A C ATPSCKMT 12 25

5 10280590 exm444396 G A CMBL 0 1

5 10286484 exm444412 A G CMBL 9 15

5 10286547 exm444414 A G CMBL 0 5

5 10461378 exm444559 A G ROPN1L 1 1

5 10465022 exm444569 G A ROPN1L 18 40

5 10683679 exm444605 A G DAP 3 8

5 13692131 exm444784 A G DNAH5 7 11

5 13700903 exm444795 A C DNAH5 5 9

5 13717471 exm444838 G A DNAH5 15 25

5 13719141 exm444859 G A DNAH5 6 10

5 13736021 exm444903 A G DNAH5 6 8

5 13766245 exm444971 A G DNAH5 6 15

5 13776677 exm445004 A C DNAH5 6 15

5 13786303 exm445033 C G DNAH5 8 31

5 13786377 exm445040 G A DNAH5 7 15

5 13793711 exm445069 A G DNAH5 5 15

5 13794053 exm445077 A G DNAH5 7 16

5 13817689 exm445125 A G DNAH5 4 11

5 13820544 exm445133 A G DNAH5 8 13

5 13820581 exm445137 A G DNAH5 4 12

5 13824358 exm445151 G C DNAH5 7 11

5 13830823 exm445179 A G DNAH5 12 32

5 13841196 exm445201 C A DNAH5 3 7

5 13870935 exm445316 A G DNAH5 7 18

5 13870976 exm445320 A G DNAH5 25 33

5 13871060 exm445325 G A DNAH5 8 18

5 13914697 exm445447 A G DNAH5 1 2

5 13914699 exm445449 G C DNAH5 3 6

5 13916540 exm445461 G A DNAH5 4 6

5 13923484 exm445507 A G DNAH5 0 1

5 13923528 exm445510 C G DNAH5 15 34

5 13931323 exm445518 A G DNAH5 10 24

5 14492907 exm445828 A C TRIO 0 1

5 14497004 exm445831 A C TRIO 0 1

5 14498633 exm445856 G A TRIO 8 25

5 16670988 exm446320 G C MYO10 18 34

5 16701140 exm446427 A G MYO10 4 7

5 16701875 exm446471 G C MYO10 3 11

5 16711332 exm446498 A C MYO10 5 14

5 16877744 exm446579 A G MYO10 4 8

5 21751961 exm446749 G A CDH12 11 22

5 21760715 exm446777 G A CDH12 1 8

5 21842417 exm446816 A G CDH12 2 2

5 31983263 exm447513 T A PDZD2 8 18

5 31983428 exm447521 A G PDZD2 6 35

5 32010445 exm447551 A G PDZD2 7 22

5 32074462 exm447656 A G PDZD2 1 0

5 32087326 exm447689 A C PDZD2 0 1

5 32087662 exm447706 A G PDZD2 11 9

5 32087891 exm447723 A G PDZD2 1 0

5 32088073 exm447729 G A PDZD2 1 3

5 32088500 exm447748 A G PDZD2 0 1

5 32088942 exm447766 C G PDZD2 4 12

5 32089103 exm447771 A G PDZD2 11 18

5 32089831 exm447821 A G PDZD2 0 3

5 32090776 exm447875 A G PDZD2 2 5

5 32098522 exm447916 A G PDZD2 10 23

5 32098563 exm447918 A G PDZD2 18 38

5 32108215 exm447940 G A PDZD2 1 1

5 32248962 exm448038 G A MTMR12 1 1

5 32263316 exm448045 A G MTMR12 3 10

5 33448705 exm448280 A G TARS 5 7

5 33448792 exm448285 A C TARS 1 1

5 33576410 exm448435 G A ADAMTS12 4 16

5 34823686 exm448981 C A RAI14 16 29

5 34829851 exm449014 C A RAI14 0 9

5 34880310 exm449053 A G TTC23L 4 9

5 34880331 exm449055 A G TTC23L 13 30

5 34909420 exm449067 G A RAD1 2 8

5 34923254 exm449126 A G BRIX1 4 12

5 34925545 exm449141 C A BRIX1 8 16

5 34937648 exm449170 A G DNAJC21 3 10

5 34949744 exm449208 A G DNAJC21 2 4

5 34950386 exm449216 A G DNAJC21 7 23

5 35068967 exm449392 A T PRLR 1 2

5 35072712 exm449399 C A PRLR 8 14

5 35667226 exm449467 G A SPEF2 2 8

5 35667268 exm449470 A G SPEF2 1 2

5 35700837 exm449524 A G SPEF2 10 22

5 35705844 exm449536 C A SPEF2 3 2

5 35763641 exm449583 G C SPEF2 15 30

5 35776479 exm449612 G A SPEF2 2 7

5 35792507 exm449625 G A SPEF2 2 6

5 35867525 exm449682 C A IL7R 3 10

5 35867559 exm449685 G A IL7R 9 12

5 35874622 exm449717 A G IL7R 7 22

5 35876256 exm449731 A G IL7R 1 1

5 35955951 exm449838 A G UGT3A1 2 5

5 35965745 exm449859 A C UGT3A1 2 11

5 36049416 exm449937 A G UGT3A2 20 34

5 36052063 exm449946 T A UGT3A2 15 33

5 36064366 exm449947 A G UGT3A2 10 23

5 36064392 exm449948 G A UGT3A2 4 16

5 36226617 exm450125 C A NADK2 19 33

5 36680556 exm450261 A G SLC1A3 6 8

5 36955688 exm450275 G A NIPBL 2 10

5 36985089 exm450328 C A NIPBL 2 3

5 36985173 exm450333 A C NIPBL 1 1

5 37138835 exm450553 G A CPLANE1 7 12

5 37184968 exm450729 G C CPLANE1 4 12

5 37328495 exm450931 G A NUP155 4 6

5 38406251 exm451178 A G EGFLAM 0 2

5 38407981 exm451205 A C EGFLAM 13 21

5 38409148 exm451209 A G EGFLAM 10 14

5 38427316 exm451249 A C EGFLAM 0 2

5 38427321 exm451250 A G EGFLAM 0 1

5 38435305 exm451261 A G EGFLAM 16 33

5 38438393 exm451267 A G EGFLAM 0 1

5 38451538 exm451291 A G EGFLAM 4 5

5 38458490 exm451301 A G EGFLAM 15 28

5 38493858 exm451374 A T LIFR 9 13

5 38493874 exm451375 G A LIFR 21 60

5 38506031 exm451398 G A LIFR 14 33

5 38883961 exm451478 A G OSMR 12 20

5 38884114 exm451485 T A OSMR 15 24

5 38924534 exm451540 A C OSMR 13 23

5 38933005 exm451564 C G OSMR 11 36

5 38933485 exm451583 A G OSMR 0 2

5 39119027 exm451777 G A FYB1 2 3

5 39126202 exm451791 C G FYB1 16 31

5 39153695 exm451818 C G FYB1 1 3

5 39202773 exm451849 A C FYB1 3 5

5 39311376 exm451906 A G C9 5 17

5 39341303 exm451931 A C C9 0 1

5 39376173 exm451972 A C DAB2 12 26

5 39377320 exm452000 C G DAB2 9 16

5 40691893 exm452105 A G PTGER4 46 73

5 40692272 exm452119 A G PTGER4 1 4

5 40692317 exm452120 A G PTGER4 1 0

5 40841741 exm452257 A G CARD6 11 26

5 40843735 exm452286 A C CARD6 1 3

5 40852701 exm452311 A C CARD6 4 15

5 40852920 exm452319 A G CARD6 14 31

5 40853031 exm452321 A G CARD6 3 7

5 40853376 exm452333 A G CARD6 19 41

5 40853487 exm452339 C G CARD6 1 10

5 40945424 exm452426 G C C7 7 12

5 40962201 exm452473 G A C7 2 11

5 40998779 exm452536 G A MROH2B 25 54

5 41000865 exm452550 A G MROH2B 3 7

5 41018868 exm452623 A G MROH2B 5 16

5 41045927 exm452669 A G MROH2B 15 25

5 41052592 exm452696 G A MROH2B 3 4

5 41052617 exm452697 A G MROH2B 0 1

5 41055888 exm452706 A G MROH2B 3 7

5 41064577 exm452722 A T MROH2B 4 14

5 41862758 exm452993 A G OXCT1 0 2

5 42689094 exm453082 A C GHR 7 13

5 42718577 exm453118 G A GHR 11 20

5 43175194 exm453343 C G ZNF131 9 29

5 43175202 exm453344 C A ZNF131 6 3

5 43246005 exm453356 A G NIM1K 11 29

5 43280431 exm453402 G C NIM1K 1 3

5 43382029 exm453461 A C CCL28 4 4

5 43412366 exm453471 G A CCL28 17 24

5 43653243 exm453720 A G NNT 7 21

5 43656074 exm453729 A G NNT 13 45

5 43656866 exm453735 A G NNT 0 2

5 43675755 exm453753 A G NNT 1 0

5 43700321 exm453761 G A NNT 6 17

5 43704469 exm453769 A G NNT 12 14

5 49699209 exm453972 G A EMB 3 3

5 50685756 exm454119 G A ISL1 3 11

5 52160871 exm454194 G C ITGA1 4 11

5 52216237 exm454264 G A ITGA1 8 20

5 52248266 exm454319 A C ITGA1 1 10

5 54327290 exm454825 A G GZMK 0 2

5 54327334 exm454829 A G GZMK 0 3

5 54329597 exm454838 C A GZMK 1 13

5 54410065 exm454891 A G CDC20B 16 37

5 54416315 exm454903 A G CDC20B 11 29

5 54420707 exm454913 A G CDC20B 16 37

5 54423155 exm454924 A G CDC20B 0 3

5 54424334 exm454930 A C CDC20B 16 30

5 54439342 exm454946 C A CDC20B 4 5

5 54468450 exm454994 G A CDC20B 4 13

5 54527249 exm455008 A G CCNO 5 12

5 54527254 exm455009 C G CCNO 3 6

5 54572102 exm455121 C A DHX29 8 7

5 54579372 exm455151 G A DHX29 0 3

5 54585213 exm455176 A G DHX29 23 56

5 54683850 exm455288 A G MTREX 1 1

5 54710038 exm455313 G A MTREX 9 10

5 55063738 exm455483 G A DDX4 1 0

5 55086523 exm455531 A G DDX4 3 4

5 55147460 exm455572 G A IL31RA 10 30

5 55212626 exm455676 A G IL31RA 11 33

5 55248135 exm455736 A G IL6ST 21 38

5 55272085 exm455789 C G IL6ST 2 9

5 55406966 exm455805 A G ANKRD55 0 4

5 55407523 exm455824 A C ANKRD55 16 23

5 55422877 exm455847 G A ANKRD55 19 35

5 55466608 exm455874 A G ANKRD55 8 22

5 56155651 exm455919 A G MAP3K1 2 6

5 56178587 exm456019 C A MAP3K1 1 2

5 56542196 exm456212 G A GPBP1 11 17

5 56542226 exm456214 G A GPBP1 16 25

5 56777508 exm456256 A G ACTBL2 5 6

5 56777915 exm456276 A G ACTBL2 14 21

5 56777916 exm456277 A G ACTBL2 10 24

5 56778093 exm456287 A G ACTBL2 6 13

5 58147096 exm456453 A G RAB3C 7 14

5 58147141 exm456457 C A RAB3C 20 34

5 60217981 exm456738 A T ERCC8 0 1

5 61811328 exm457041 C G IPO11 0 1

5 61856986 exm457071 A G IPO11 0 1

5 61923026 exm457117 G A IPO11 9 32

5 64824298 exm457537 C G CENPK 6 12

5 64850659 exm457558 G A CENPK 6 11

5 65288668 exm457867 G A ERBIN 2 4

5 65290647 exm457873 G A ERBIN 9 24

5 65317158 exm457882 G A ERBIN 13 25

5 65321320 exm457896 C A ERBIN 2 8

5 65321695 exm457901 G C ERBIN 0 1

5 65349298 exm457947 G C ERBIN 1 3

5 65349563 exm457957 G A ERBIN 14 24

5 65349908 exm457973 A T ERBIN 0 3

5 65465971 exm458062 A G SREK1 6 13

5 65465986 exm458063 A G SREK1 11 22

5 66461363 exm458361 A G MAST4 10 23

5 66461884 exm458389 A G MAST4 6 6

5 66461930 exm458390 A G MAST4 0 2

5 66462325 exm458406 A T MAST4 10 20

5 66479433 exm458465 A G CD180 2 11

5 66479605 exm458473 A G CD180 3 3

5 66479896 exm458493 A G CD180 5 16

5 68399865 exm458611 A G SLC30A5 4 11

5 68414334 exm458659 A G SLC30A5 1 2

5 68414387 exm458662 G A SLC30A5 7 6

5 68555733 exm458813 G A CDK7 0 1

5 68568858 exm458822 A G CDK7 7 11

5 68578845 exm458834 C A CCDC125 3 8

5 68603827 exm458871 A G CCDC125 2 3

5 68603851 exm458872 C A CCDC125 15 20

5 68616156 exm458886 C A CCDC125 7 15

5 68669714 exm458975 G A RAD17 23 29

5 68669736 exm458976 A C RAD17 3 14

5 68715601 exm459084 T A MARVELD2 10 23

5 68716312 exm459125 A C MARVELD2 0 2

5 70759923 exm459360 A G BDP1 0 1

5 70797462 exm459421 G A BDP1 3 9

5 70798541 exm459433 G A BDP1 35 64

5 70805683 exm459463 G A BDP1 2 3

5 70806131 exm459482 G C BDP1 11 26

5 70818130 exm459567 A C BDP1 14 16

5 70858268 exm459684 G A BDP1 6 1

5 70895532 exm459711 A G MCCC2 2 2

5 70931069 exm459739 A G MCCC2 2 6

5 70945029 exm459754 G A MCCC2 0 2

5 70948552 exm459777 C G MCCC2 3 6

5 71491311 exm459895 G A MAP1B 17 40

5 71492066 exm459935 A G MAP1B 13 35

5 71492418 exm459957 A G MAP1B 5 16

5 71494083 exm460016 G C MAP1B 0 5

5 71739983 exm460245 T A ZNF366 14 31

5 71740073 exm460251 T A ZNF366 18 39

5 71756974 exm460322 A G ZNF366 1 1

5 72313066 exm460423 A G FCHO2 5 13

5 72383464 exm460470 G A FCHO2 7 16

5 72427529 exm460535 A C TMEM171 4 6

5 72469090 exm460540 A G TMEM174 5 12

5 72469416 exm460560 A G TMEM174 11 26

5 72469608 exm460569 A C TMEM174 2 5

5 72469694 exm460574 G C TMEM174 6 15

5 72864061 exm460680 A C UTP15 6 18

5 72874584 exm460723 C A UTP15 5 17

5 72874672 exm460728 C G UTP15 5 8

5 72875159 exm460734 A G UTP15 9 21

5 72875903 exm460742 C A UTP15 0 3

5 73091203 exm460813 G C ARHGEF28 6 8

5 73128166 exm460817 A G ARHGEF28 4 12

5 73188022 exm460926 G A ARHGEF28 4 7

5 74017590 exm461098 G C GFM2 5 17

5 74021793 exm461111 G A GFM2 9 17

5 74021950 exm461119 A T GFM2 7 11

5 74032707 exm461134 A G GFM2 7 11

5 74034229 exm461139 A G GFM2 5 14

5 74035892 exm461150 G A GFM2 16 39

5 74035914 exm461151 A C GFM2 7 15

5 74035959 exm461152 G C GFM2 10 17

5 74091908 exm461260 A G FAM169A 25 46

5 74109839 exm461287 G A FAM169A 15 27

5 74400378 exm461374 G A ANKRD31 12 19

5 74892422 exm461671 A G POLK 9 21

5 75427627 exm461775 C A SV2C 2 1

5 75428074 exm461799 A G SV2C 11 16

5 75490853 exm461835 A C SV2C 4 6

5 75587140 exm461866 A G SV2C 10 29

5 75919012 exm462040 G A F2RL2 5 10

5 75936917 exm462073 A G IQGAP2 11 28

5 75969841 exm462126 G A IQGAP2 2 4

5 76331387 exm462390 G A AGGF1 6 22

5 76331449 exm462394 A G AGGF1 9 26

5 76733214 exm462621 A G WDR41 4 16

5 76734102 exm462628 A G WDR41 0 6

5 76734132 exm462632 A G WDR41 6 16

5 77316593 exm462768 G A AP3B1 10 21

5 77461480 exm462838 G A AP3B1 19 35

5 77590366 exm462875 G C AP3B1 1 2

5 78135241 exm462968 A G ARSB 1 3

5 78181501 exm462977 G A ARSB 4 12

5 78265011 exm463011 A G ARSB 9 24

5 78294107 exm463035 A G DMGDH 5 8

5 78324375 exm463059 G C DMGDH 2 2

5 78351682 exm463140 G A DMGDH 4 12

5 78379026 exm463181 A G BHMT2 0 1

5 78379069 exm463185 A G BHMT2 17 47

5 78417158 exm463222 A G BHMT 0 4

5 78915852 exm463382 G A TENT2 11 24

5 78938689 exm463409 A G TENT2 3 7

5 78981028 exm463444 A G TENT2 3 8

5 79024866 exm463456 G C CMYA5 2 15

5 79024916 exm463462 A G CMYA5 5 10

5 79025441 exm463489 A G CMYA5 9 22

5 79027379 exm463563 A G CMYA5 9 21

5 79027464 exm463565 A G CMYA5 6 15

5 79027494 exm463568 A T CMYA5 4 10

5 79028025 exm463590 G A CMYA5 9 21

5 79028742 exm463617 A G CMYA5 0 1

5 79029000 exm463625 A G CMYA5 9 21

5 79029013 exm463626 C G CMYA5 7 16

5 79029675 exm463649 C G CMYA5 6 14

5 79030468 exm463683 C A CMYA5 1 1

5 79030983 exm463701 A G CMYA5 6 6

5 79031579 exm463726 A G CMYA5 7 13

5 79031711 exm463731 A G CMYA5 7 13

5 79032026 exm463741 A G CMYA5 9 21

5 79032372 exm463753 A G CMYA5 9 21

5 79032962 exm463783 A G CMYA5 0 2

5 79033529 exm463803 A G CMYA5 9 21

5 79041252 exm463870 A G CMYA5 1 1

5 79054599 exm463879 A G CMYA5 32 59

5 79089292 exm463903 A G CMYA5 10 12

5 79095299 exm463915 G A CMYA5 16 31

5 79284099 exm463943 G A MTX3 12 25

5 79355634 exm464009 A G THBS4 4 3

5 79361257 exm464018 A G THBS4 6 17

5 79366969 exm464053 T A THBS4 0 3

5 79616278 exm464164 G A SPZ1 11 23

5 79734563 exm464290 G A ZFYVE16 35 46

5 79739007 exm464307 C A ZFYVE16 11 28

5 79809524 exm464405 G A FAM151B 9 25

5 79854826 exm464458 G A ANKRD34B 1 2

5 79855114 exm464469 G A ANKRD34B 1 5

5 79855228 exm464473 A G ANKRD34B 2 3

5 80024738 exm464615 G A MSH3 11 15

5 80037285 exm464617 C A MSH3 10 28

5 80083428 exm464671 G A MSH3 8 17

5 80109487 exm464679 G A MSH3 5 13

5 80338786 exm464722 A G RASGRF2 3 7

5 80409593 exm464794 A G RASGRF2 3 5

5 80409640 exm464797 A G RASGRF2 13 32

5 80476005 exm464823 G A RASGRF2 0 1

5 80513260 exm464850 A G RASGRF2 4 3

5 80600611 exm464931 G A ZCCHC9 6 7

5 80600821 exm464946 G A ZCCHC9 1 0

5 80604431 exm464957 A G ZCCHC9 2 8

5 80607095 exm464971 G A ZCCHC9 2 8

5 80608363 exm464974 C A ZCCHC9 5 13

5 80626733 exm465001 A G ACOT12 3 4

5 80639660 exm465024 G A ACOT12 7 14

5 82491697 exm465247 C G XRCC4 10 28

5 82500734 exm465259 A C XRCC4 8 12

5 82808072 exm465314 A G VCAN 17 41

5 82815525 exm465343 A G VCAN 15 36

5 82816791 exm465393 A G VCAN 15 35

5 82816793 exm465394 A C VCAN 5 11

5 82817838 exm465432 C G VCAN 10 8

5 82834572 exm465507 T A VCAN 13 24

5 82835304 exm465533 G A VCAN 9 22

5 82836201 exm465573 G A VCAN 11 23

5 82836311 exm465579 G A VCAN 1 2

5 82836834 exm465604 A C VCAN 5 10

5 82837247 exm465621 A G VCAN 1 1

5 82837695 exm465639 A G VCAN 2 1

5 86564430 exm465841 C G RASA1 5 17

5 86564564 exm465850 A G RASA1 2 5

5 89930946 exm466319 C A ADGRV1 9 15

5 89938764 exm466343 G A ADGRV1 3 6

5 89948255 exm466383 G A ADGRV1 5 4

5 89971213 exm466450 A G ADGRV1 6 11

5 89981639 exm466500 A G ADGRV1 11 13

5 90000210 exm466589 A G ADGRV1 11 25

5 90001299 exm466599 C A ADGRV1 0 4

5 90004717 exm466609 A G ADGRV1 4 10

5 90008144 exm466619 G A ADGRV1 4 9

5 90021438 exm466663 G A ADGRV1 7 13

5 90040920 exm466682 A G ADGRV1 17 27

5 90041590 exm466692 G A ADGRV1 3 9

5 90049470 exm466699 G A ADGRV1 13 34

5 90119330 exm466908 A G ADGRV1 4 12

5 90149952 exm466943 G A ADGRV1 3 10

5 90159576 exm466950 A C ADGRV1 1 3

5 90449159 exm466986 C A ADGRV1 7 19

5 94253638 exm467415 A G MCTP1 11 44

5 94749787 exm467498 A G FAM81B 30 68

5 94820477 exm467577 A G TTC37 2 10

5 94826668 exm467586 G C TTC37 4 11

5 94838684 exm467618 A G TTC37 3 5

5 95011166 exm467906 A G SPATA9 3 10

5 95011279 exm467910 A G SPATA9 6 16

5 95728710 exm468098 C A PCSK1 0 2

5 95748035 exm468154 G A PCSK1 0 1

5 95751785 exm468164 G A PCSK1 24 38

5 96058392 exm468201 A C CAST 2 5

5 96076456 exm468231 G A CAST 3 6

5 96078438 exm468262 A G CAST 2 10

5 96082087 exm468282 A T CAST 11 30

5 96090384 exm468305 A G CAST 13 28

5 96139428 exm468536 A G ERAP1 3 5

5 96139595 exm468552 A G ERAP1 38 61

5 96215729 exm468575 A C ERAP2 5 19

5 96215747 exm468578 C G ERAP2 5 12

5 96237991 exm468673 T A ERAP2 2 3

5 96239153 exm468678 G A ERAP2 2 12

5 96315087 exm468737 A G LNPEP 9 23

5 96315088 exm468738 A G LNPEP 1 6

5 96315411 exm468747 C A LNPEP 10 16

5 96320815 exm468752 A G LNPEP 1 3

5 96362335 exm468824 C G LNPEP 5 5

5 96362385 exm468826 G A LNPEP 2 3

5 96500786 exm468885 C G RIOK2 10 24

5 96503387 exm468891 A G RIOK2 1 1

5 96507040 exm468907 A G RIOK2 4 4

5 96508943 exm468911 A G RIOK2 0 1

5 98115367 exm468967 A G RGMB 5 6

5 98129080 exm469003 A G RGMB 1 8

5 98194682 exm469048 G A CHD1 7 25

5 98228403 exm469116 A T CHD1 4 6

5 101709088 exm469343 G A SLCO6A1 5 10

5 101815901 exm469425 C A SLCO6A1 7 19

5 101815983 exm469430 G A SLCO6A1 7 19

5 101816115 exm469438 A G SLCO6A1 7 19

5 101834413 exm469449 G A SLCO6A1 1 1

5 102203032 exm469467 G C PAM 1 2

5 102310135 exm469525 A G PAM 5 11

5 102326054 exm469535 A G PAM 5 8

5 102338811 exm469544 G A PAM 1 4

5 102433349 exm469619 G A GIN1 14 22

5 102520389 exm469733 A G PPIP5K2 2 3

5 102530663 exm469753 A G PPIP5K2 0 2

5 102895072 exm469859 G A NUDT12 0 2

5 108698676 exm470092 G A PJA2 5 5

5 108714685 exm470139 G A PJA2 5 5

5 109049221 exm470167 A G MAN2A1 2 7

5 109110536 exm470216 A G MAN2A1 2 5

5 109120497 exm470234 G A MAN2A1 1 4

5 110428093 exm470471 C A WDR36 9 14

5 111504782 exm470789 A C EPB41L4A 0 1

5 112163681 exm470983 A G APC 6 9

5 112173752 exm471010 A G APC 0 2

5 112173884 exm471014 A G APC 12 18

5 112174941 exm471044 C A APC 3 9

5 112175240 exm471056 G C APC 15 31

5 112179557 exm471212 G A APC 2 3

5 112403779 exm471438 A G MCC 2 8

5 112403854 exm471446 C A MCC 2 5

5 112851022 exm471680 A C YTHDC2 6 20

5 112868684 exm471699 A G YTHDC2 4 12

5 112889370 exm471759 G C YTHDC2 22 45

5 112915373 exm471824 G A YTHDC2 2 7

5 112920102 exm471845 G A YTHDC2 12 17

5 112920108 exm471847 C G YTHDC2 22 44

5 112926768 exm471851 G A YTHDC2 22 46

5 114480435 exm472007 G A TRIM36 1 2

5 114506846 exm472032 G A TRIM36 1 1

5 115319078 exm472405 G A LVRN 2 4

5 115323557 exm472418 C G LVRN 14 32

5 115346567 exm472480 C G LVRN 1 2

5 115350190 exm472487 A C LVRN 6 12

5 115350212 exm472488 G A LVRN 3 8

5 115428310 exm472544 C G COMMD10 5 7

5 115469825 exm472556 C A COMMD10 10 15

5 115782476 exm472585 A C SEMA6A 0 2

5 115811248 exm472647 A C SEMA6A 7 12

5 115811273 exm472649 A G SEMA6A 0 1

5 115813578 exm472656 G A SEMA6A 6 16

5 118451951 exm472813 C A DMXL1 16 28

5 118454596 exm472820 G A DMXL1 5 10

5 118468862 exm472846 A C DMXL1 8 25

5 118480316 exm472888 A G DMXL1 0 2

5 118484637 exm472919 T A DMXL1 11 24

5 118484821 exm472928 G A DMXL1 13 33

5 118506179 exm473028 G A DMXL1 8 21

5 118506685 exm473049 A G DMXL1 17 29

5 118507599 exm473065 C G DMXL1 10 17

5 118529578 exm473106 A G DMXL1 2 2

5 118533576 exm473123 A G DMXL1 1 3

5 118539031 exm473128 A G DMXL1 0 3

5 118969755 exm473345 A C FAM170A 7 12

5 121355025 exm473473 A T SRFBP1 3 6

5 121356304 exm473497 A G SRFBP1 2 7

5 121356403 exm473505 A C SRFBP1 13 20

5 121487886 exm473584 G A ZNF474 8 22

5 121488431 exm473616 G A ZNF474 19 34

5 121758649 exm473655 G A SNCAIP 2 6

5 121761192 exm473705 A C SNCAIP 4 11

5 121785608 exm473730 A G SNCAIP 13 34

5 122758665 exm474094 G A CEP120 7 9

5 123979991 exm474171 A G ZNF608 10 14

5 123980324 exm474189 A G ZNF608 1 0

5 124080046 exm474304 A G ZNF608 5 5

5 125887751 exm474418 G C ALDH7A1 0 1

5 125939409 exm474494 A G PHAX 21 41

5 125939436 exm474495 A G PHAX 3 8

5 125939818 exm474516 A G PHAX 1 5

5 126732263 exm474719 A G MEGF10 6 7

5 126758369 exm474781 A C MEGF10 5 13

5 126781311 exm474832 A G MEGF10 8 16

5 127469872 exm475013 G A SLC12A2 0 1

5 127493761 exm475041 G A SLC12A2 12 20

5 127520179 exm475081 A C SLC12A2 2 4

5 127624232 exm475211 G A FBN2 11 31

5 127637137 exm475245 A G FBN2 1 2

5 127671227 exm475341 G A FBN2 16 43

5 127671254 exm475344 G A FBN2 2 7

5 127686688 exm475403 G A FBN2 3 7

5 128321002 exm475573 A G SLC27A6 2 4

5 128351606 exm475588 A G SLC27A6 8 15

5 128351680 exm475594 G A SLC27A6 1 3

5 128351735 exm475595 A G SLC27A6 1 3

5 128362864 exm475603 C A SLC27A6 19 35

5 129520227 exm475859 G A CHSY3 8 14

5 129520279 exm475864 A C CHSY3 15 31

5 130517930 exm475945 C A LYRM7 2 6

5 130517944 exm475946 C A LYRM7 19 36

5 130766905 exm476004 A G RAPGEF6 13 17

5 130799921 exm476084 G A RAPGEF6 1 2

5 130939019 exm476158 C A RAPGEF6 1 2

5 131302110 exm476301 A G ACSL6 5 8

5 131302170 exm476304 A G ACSL6 26 55

5 131308521 exm476320 A G ACSL6 9 11

5 131325138 exm476375 A G ACSL6 5 12

5 131726578 exm476770 G A SLC22A5 25 40

5 131728202 exm476773 C A SLC22A5 6 15

5 131729496 exm476789 C G SLC22A5 0 2

5 131729880 exm476792 A C SLC22A5 22 31

5 131995423 exm477068 A C IL13 6 11

5 132084106 exm477187 G A CCNI2 8 15

5 132087697 exm477202 A G CCNI2 7 8

5 132197396 exm477478 A G GDF9 12 23

5 132197442 exm477481 A G GDF9 2 4

5 132270330 exm477674 A G AFF4 0 4

5 132387979 exm477708 T A HSPA4 16 41

5 132406153 exm477719 A G HSPA4 2 12

5 132426998 exm477763 A G HSPA4 3 11

5 132437499 exm477788 A G HSPA4 1 1

5 133644061 exm478204 G A CDKL3 3 6

5 133914568 exm478358 A G JADE2 10 26

5 133914640 exm478365 A G JADE2 3 5

5 133997168 exm478436 A G SEC24A 13 20

5 134305715 exm478788 A G CATSPER3 3 4

5 134344573 exm478825 A G CATSPER3 17 33

5 134782579 exm478977 G A DCANP1 8 13

5 134782728 exm478987 A T DCANP1 9 11

5 135382678 exm479285 A T TGFBI 2 4

5 135388634 exm479305 A G TGFBI 1 0

5 135390525 exm479330 C A TGFBI 0 2

5 135391499 exm479349 A G TGFBI 1 2

5 135392451 exm479355 A G TGFBI 8 9

5 135601967 exm479467 G A TRPC7 12 23

5 135693038 exm479504 G C TRPC7 12 30

5 136974818 exm479615 G A KLHL3 3 7

5 137045507 exm479657 G A KLHL3 1 1

5 137284675 exm479889 A G FAM13B 7 11

5 137295403 exm479924 G A FAM13B 5 15

5 137320974 exm479932 A G FAM13B 3 6

5 137426668 exm480010 A G WNT8A 24 41

5 137426727 exm480014 A G WNT8A 3 7

5 137451417 exm480023 G A NME5 7 12

5 137454567 exm480036 G A NME5 17 29

5 137476500 exm480063 A G BRD8 10 27

5 137486493 exm480080 A C BRD8 3 9

5 137506599 exm480190 A G BRD8 0 3

5 137515392 exm480208 A G KIF20A 0 3

5 137521217 exm480288 G A KIF20A 1 0

5 137627784 exm480473 A G CDC25C 1 4

5 137665298 exm480490 A G CDC25C 19 24

5 137727169 exm480617 A C KDM3B 3 11

5 138718219 exm481355 A G SLC23A1 2 3

5 138764329 exm481484 G C DNAJC18 21 50

5 138773124 exm481488 A G DNAJC18 4 8

5 139189086 exm481644 A G PSD2 4 7

5 139189174 exm481651 A G PSD2 0 1

5 139189345 exm481662 G C PSD2 16 24

5 139193291 exm481691 G C PSD2 2 4

5 139222049 exm481775 C G PSD2 6 14

5 139231255 exm481804 A G NRG2 23 31

5 139267090 exm481862 A G NRG2 1 6

5 139739898 exm481992 G A SLC4A9 6 16

5 139743342 exm482043 A G SLC4A9 6 19

5 139828814 exm482165 A G ANKHD1;ANKHD1-EIF4EBP3 2 15

5 139876612 exm482214 A G ANKHD1;ANKHD1-EIF4EBP3 4 4

5 139908926 exm482303 A G ANKHD1;ANKHD1-EIF4EBP3 7 21

5 139917800 exm482350 G A ANKHD1;ANKHD1-EIF4EBP3 11 30

5 139941976 exm482567 A G APBB3 5 7

5 140011548 exm482662 A G CD14 3 3

5 140011785 exm482669 A C CD14 10 20

5 140044576 exm482847 A G WDR55 6 14

5 140048707 exm482878 G A WDR55 15 19

5 140166347 exm483156 A G PCDHA1 4 5

5 140181359 exm483452 A G PCDHA3 17 27

5 140182722 exm483522 G A PCDHA3 11 23

5 140207681 exm483832 G A PCDHA6 12 36

5 140208301 exm483862 C A PCDHA6 0 3

5 140209988 exm483996 A G PCDHA6 17 31

5 140238124 exm484611 A T PCDHA10 0 2

5 140249154 exm484635 A G PCDHA11 10 21

5 140249554 exm484650 A G PCDHA11 4 4

5 140256202 exm484797 G A PCDHA12 6 8

5 140257401 exm484872 A G PCDHA12 18 31

5 140307271 exm485048 G A PCDHAC1 12 22

5 140307567 exm485063 A C PCDHAC1 2 10

5 140307964 exm485087 A G PCDHAC1 6 23

5 140432208 exm485336 G A PCDHB1 16 29

5 140433211 exm485372 A T PCDHB1 2 0

5 140474467 exm485391 A C PCDHB2 7 15

5 140474796 exm485406 C A PCDHB2 8 25

5 140475377 exm485423 G C PCDHB2 1 3

5 140480442 exm485526 A G PCDHB3 1 1

5 140480682 exm485537 A G PCDHB3 1 2

5 140481276 exm485558 G C PCDHB3 12 28

5 140515404 exm485758 A C PCDHB5 8 11

5 140515578 exm485764 C G PCDHB5 4 7

5 140529975 exm485888 G A PCDHB6 2 4

5 140530598 exm485919 C A PCDHB6 14 23

5 140530632 exm485921 A G PCDHB6 4 6

5 140579498 exm486809 A G PCDHB11 11 14

5 140579889 exm486827 C A PCDHB11 14 27

5 140579929 exm486831 C A PCDHB11 1 2

5 140594675 exm487148 A C PCDHB13 10 22

5 140596092 exm487247 G A PCDHB13 10 23

5 140604011 exm487292 T A PCDHB14 16 42

5 140604213 exm487299 A G PCDHB14 2 7

5 140682891 exm487556 C A SLC25A2 2 8

5 140683242 exm487572 G A SLC25A2 12 29

5 140718936 exm487747 G A PCDHGA2 1 3

5 140719230 exm487762 G A PCDHGA2 0 1

5 140720954 exm487863 G A PCDHGA2 16 33

5 140724259 exm487898 C G PCDHGA3 10 24

5 140724990 exm487928 G A PCDHGA3 1 7

5 140735499 exm488121 A C PCDHGA4 6 19

5 140740132 exm488212 A G PCDHGB2 0 2

5 140740910 exm488233 G A PCDHGB2 2 8

5 140744651 exm488323 G C PCDHGA5 18 34

5 140750712 exm488427 A G PCDHGB3 2 3

5 140768577 exm488742 C G PCDHGB4 1 4

5 140768709 exm488747 A G PCDHGB4 4 12

5 140768844 exm488756 A G PCDHGB4 8 10

5 140773225 exm488830 A G PCDHGA8 1 3

5 140778879 exm488927 C A PCDHGB5 11 28

5 140780029 exm488987 A C PCDHGB5 5 13

5 140784495 exm489068 A G PCDHGA9 4 8

5 140784674 exm489078 C A PCDHGA9 1 2

5 140788965 exm489144 G A PCDHGB6 3 7

5 140795128 exm489278 G A PCDHGA10 32 57

5 140797827 exm489311 A G PCDHGB7 4 3

5 140801771 exm489444 A G PCDHGA11 7 15

5 140801950 exm489450 G A PCDHGA11 15 34

5 140802316 exm489459 G A PCDHGA11 12 15

5 140802620 exm489477 G A PCDHGA11 15 21

5 140811573 exm489560 A G PCDHGA12 6 19

5 140811605 exm489563 A G PCDHGA12 0 1

5 140811738 exm489575 G A PCDHGA12 6 10

5 140811814 exm489578 G C PCDHGA12 2 3

5 140856045 exm489635 T A PCDHGC3 2 6

5 140856422 exm489651 G A PCDHGC3 16 29

5 140951529 exm489972 A G DIAPH1 2 6

5 141021111 exm490182 A G FCHSD1 0 4

5 141021294 exm490184 C A FCHSD1 11 20

5 141025730 exm490232 C A FCHSD1 5 11

5 141026182 exm490235 A C FCHSD1 1 0

5 141033870 exm490319 C A ARAP3 1 3

5 141041285 exm490386 A G ARAP3 0 1

5 141049343 exm490439 A G ARAP3 4 10

5 141051274 exm490459 A G ARAP3 10 7

5 141052986 exm490509 A G ARAP3 1 1

5 141053220 exm490511 A G ARAP3 2 3

5 141243141 exm490623 A G PCDH1 3 6

5 141248994 exm490726 A G PCDH1 1 0

5 141304176 exm490735 A G DELE1 2 2

5 141304248 exm490738 A G DELE1 1 2

5 141304989 exm490741 A G DELE1 3 8

5 141309209 exm490766 A G DELE1 18 29

5 141337177 exm491013 C G PCDH12 3 7

5 141367344 exm491062 G C RNF14 2 6

5 145393493 exm491642 A G SH3RF2 6 13

5 145393494 exm491643 A G SH3RF2 8 17

5 145439627 exm491699 G A SH3RF2 9 27

5 145613243 exm491983 T A RBM27 17 29

5 145638056 exm492000 G A RBM27 15 23

5 145894731 exm492240 A G GPR151 2 4

5 145895074 exm492259 A G GPR151 3 13

5 145895088 exm492260 C G GPR151 2 0

5 145895387 exm492272 A G GPR151 3 8

5 147806815 exm493002 G A FBXO38 0 5

5 147807057 exm493010 A G FBXO38 0 5

5 148384455 exm493244 A T SH3TC2 7 6

5 148388420 exm493254 A G SH3TC2 1 1

5 148407433 exm493347 A G SH3TC2 12 17

5 148407527 exm493352 A G SH3TC2 0 1

5 148407773 exm493362 A G SH3TC2 0 2

5 148408100 exm493382 G A SH3TC2 10 18

5 148408117 exm493384 A G SH3TC2 2 4

5 148422274 exm493420 A G SH3TC2 3 11

5 148431783 exm493444 A C SH3TC2 1 3

5 148563057 exm493459 G A ABLIM3 14 52

5 148619424 exm493535 A G ABLIM3 1 4

5 148682030 exm493632 G A AFAP1L1 6 11

5 148695816 exm493675 G A AFAP1L1 4 10

5 148697346 exm493684 G A AFAP1L1 6 15

5 148699986 exm493703 G A AFAP1L1 4 7

5 149001530 exm493996 G A ARHGEF37 23 33

5 149003583 exm494002 G C ARHGEF37 4 15

5 149003626 exm494004 A G ARHGEF37 0 3

5 149212301 exm494094 A G PPARGC1B 2 4

5 149212352 exm494100 A G PPARGC1B 5 10

5 149213053 exm494151 C G PPARGC1B 7 12

5 149213218 exm494168 A G PPARGC1B 3 12

5 149216276 exm494194 A G PPARGC1B 3 5

5 149265922 exm494315 A G PDE6A 7 12

5 149276063 exm494335 C A PDE6A 0 1

5 149360451 exm494482 G A SLC26A2 1 5

5 149361243 exm494507 G A SLC26A2 9 37

5 149374515 exm494518 G A TIGD6 13 27

5 149374585 exm494522 G C TIGD6 3 12

5 149375133 exm494546 G A TIGD6 4 4

5 149384422 exm494580 A G HMGXB3 12 14

5 149441152 exm494732 A G CSF1R 6 18

5 149447852 exm494742 A G CSF1R 12 27

5 149456926 exm494782 A G CSF1R 1 9

5 149459750 exm494803 C A CSF1R 13 20

5 149459893 exm494814 A G CSF1R 0 2

5 149509446 exm494951 A G PDGFRB 18 36

5 149509461 exm494952 A G PDGFRB 0 1

5 149677480 exm495325 A G ARSI 11 25

5 149754216 exm495446 A C TCOF1 29 57

5 149754948 exm495469 G A TCOF1 4 10

5 149755443 exm495496 A G TCOF1 4 5

5 149755744 exm495502 C G TCOF1 1 4

5 149755839 exm495507 C A TCOF1 6 26

5 149782801 exm495640 A G CD74 9 26

5 149786874 exm495666 A G CD74 5 8

5 150028582 exm495891 A G SYNPO 0 8

5 150029579 exm495947 A G SYNPO 5 8

5 150036119 exm495972 G A SYNPO 0 2

5 150050049 exm495996 C G MYOZ3 1 1

5 150051171 exm496007 C A MYOZ3 3 5

5 150056367 exm496028 A G MYOZ3 5 5

5 150413290 exm496326 A G TNIP1 9 12

5 150431799 exm496381 G A TNIP1 17 30

5 150436517 exm496391 A G TNIP1 20 39

5 150497395 exm496444 G A ANXA6 20 29

5 150664199 exm496681 G A SLC36A3 4 3

5 150675808 exm496718 A G SLC36A3 7 13

5 150701744 exm496770 A G SLC36A2 0 1

5 150726946 exm496837 G A SLC36A2 11 19

5 150838427 exm496842 A G SLC36A1 0 1

5 150885322 exm496933 C G FAT2 9 19

5 150885451 exm496939 A G FAT2 9 19

5 150887066 exm496967 A G FAT2 8 20

5 150907564 exm497086 A G FAT2 1 2

5 150908750 exm497096 A G FAT2 7 17

5 150921981 exm497178 C G FAT2 3 11

5 150922589 exm497210 A G FAT2 3 15

5 150924087 exm497264 A G FAT2 3 14

5 150925883 exm497341 G A FAT2 25 33

5 150932918 exm497387 A G FAT2 18 28

5 150934209 exm497399 A C FAT2 20 28

5 150945830 exm497462 A G FAT2 5 10

5 150946478 exm497498 G A FAT2 1 2

5 150946743 exm497507 T A FAT2 5 15

5 150947162 exm497524 G A FAT2 1 1

5 151045999 exm497607 A C SPARC 1 0

5 153406864 exm498016 G A FAM114A2 12 25

5 153406881 exm498018 A G FAM114A2 0 1

5 153709239 exm498118 G A GALNT10 0 2

5 153755996 exm498138 G A GALNT10 2 1

5 153796456 exm498196 G A GALNT10 3 4

5 154200906 exm498393 A C FAXDC2 6 11

5 154202049 exm498398 A G FAXDC2 8 12

5 154203080 exm498414 A G FAXDC2 2 7

5 154203121 exm498418 A G FAXDC2 13 20

5 154275863 exm498503 A G GEMIN5 12 26

5 154287276 exm498563 A G GEMIN5 1 14

5 154296611 exm498592 A G GEMIN5 5 19

5 154311021 exm498638 A G GEMIN5 0 5

5 154396295 exm498812 G A KIF4B 5 16

5 156346485 exm498886 A G TIMD4 2 6

5 156381695 exm498960 A C TIMD4 5 10

5 156514129 exm499069 A G HAVCR2 3 3

5 156514189 exm499071 A G HAVCR2 3 2

5 156522370 exm499080 G C HAVCR2 8 18

5 156565812 exm499116 G A MED7 11 30

5 156565880 exm499122 A G MED7 8 17

5 156589696 exm499146 G A FAM71B 0 1

5 156589838 exm499152 C A FAM71B 6 12

5 156590267 exm499185 A T FAM71B 9 11

5 156650008 exm499263 C G ITK 3 3

5 156659403 exm499269 A G ITK 2 9

5 156890181 exm499499 A C NIPAL4 1 2

5 156929816 exm499637 A G ADAM19 5 17

5 157053365 exm499701 A G SOX30 6 15

5 157053610 exm499713 G A SOX30 4 7

5 157073747 exm499742 A C SOX30 13 37

5 157214823 exm499922 A G CLINT1 2 5

5 157218761 exm499934 G A CLINT1 10 21

5 158139228 exm499983 A G EBF1 6 16

5 158743788 exm500154 A C IL12B 3 1

5 158745850 exm500167 A G IL12B 10 23

5 158747467 exm500179 A G IL12B 6 20

5 158750143 exm500191 A G IL12B 9 35

5 159520921 exm500337 A G PWWP2A 2 7

5 159680551 exm500411 A G CCNJL 3 11

5 159686536 exm500439 A G CCNJL 2 2

5 159686752 exm500445 A G CCNJL 0 1

5 159707588 exm500456 G A CCNJL 11 28

5 159822263 exm500582 C A ZBED8 9 10

5 159992594 exm500701 C A ATP10B 4 10

5 160016684 exm500723 G A ATP10B 19 43

5 160034015 exm500763 A G ATP10B 7 16

5 160034020 exm500764 A G ATP10B 15 26

5 160049586 exm500839 A G ATP10B 4 6

5 161116672 exm501014 A G GABRA6 7 9

5 161119160 exm501045 G A GABRA6 6 17

5 162896686 exm501251 A G HMMR 17 35

5 162910329 exm501312 A G HMMR 14 25

5 167642176 exm501528 A G TENM2 14 34

5 167645826 exm501567 C G TENM2 3 5

5 167689255 exm501682 A G TENM2 15 28

5 167855044 exm501797 G A WWC1 4 7

5 167858430 exm501830 A G WWC1 13 31

5 167919703 exm501930 G A RARS 1 2

5 167919848 exm501936 A G RARS 1 6

5 167933095 exm501963 T A RARS 9 21

5 167943822 exm501985 A G RARS 4 9

5 168096793 exm502100 G A SLIT3 0 5

5 169020482 exm502434 A C SPDL1 17 27

5 169025501 exm502467 G A SPDL1 20 46

5 169026043 exm502477 A G SPDL1 2 9

5 169028383 exm502487 A G SPDL1 1 1

5 169111224 exm502541 G A DOCK2 11 22

5 169111233 exm502542 A G DOCK2 1 4

5 169129364 exm502577 G A DOCK2 0 1

5 169129408 exm502578 A G DOCK2 12 34

5 169469096 exm502741 A G DOCK2 5 12

5 169504802 exm502789 G A DOCK2 26 40

5 169504823 exm502791 C G DOCK2 11 27

5 169508983 exm502824 G A DOCK2 5 10

5 169533092 exm502839 A G FOXI1 5 10

5 169535254 exm502868 A G FOXI1 1 5

5 170319406 exm503185 G A RANBP17 4 9

5 170668064 exm503316 C A RANBP17 11 36

5 170720930 exm503343 A G RANBP17 4 8

5 171479958 exm503533 A G STK10 1 0

5 171482707 exm503553 A G STK10 3 4

5 171482726 exm503554 A G STK10 1 3

5 171532728 exm503645 A G STK10 8 13

5 171765711 exm503755 A G SH3PXD2B 8 12

5 175782737 exm504902 A G KIAA1191 4 15

5 175782744 exm504903 A C KIAA1191 8 18

5 176002295 exm505215 A G CDHR2 7 23

5 176002580 exm505224 A G CDHR2 4 3

5 176005481 exm505292 A G CDHR2 4 7

5 176011557 exm505328 A G CDHR2 17 36

5 176011579 exm505332 G A CDHR2 3 13

5 176011968 exm505361 A G CDHR2 19 28

5 176011984 exm505362 A G CDHR2 0 1

5 176025791 exm505508 G A GPRIN1 5 18

5 176026315 exm505549 A G GPRIN1 2 3

5 176072513 exm505627 A G EIF4E1B 1 5

5 176072868 exm505629 A G EIF4E1B 6 8

5 176079877 exm505654 G A TSPAN17 0 1

5 176314206 exm505918 G A HK3 18 35

5 176314610 exm505940 A G HK3 2 5

5 176335565 exm506070 G A UIMC1 2 5

5 176520465 exm506281 A G FGFR4 6 8

5 176522675 exm506313 A G FGFR4 9 16

5 176524540 exm506357 A G FGFR4 6 15

5 176524573 exm506361 A G FGFR4 2 10

5 176562864 exm506391 A G NSD1 3 8

5 176636974 exm506418 A G NSD1 2 14

5 176637471 exm506438 A G NSD1 7 19

5 176638789 exm506498 G A NSD1 1 3

5 176675204 exm506531 A G NSD1 13 24

5 176795725 exm506860 A G RGS14 2 8

5 176795796 exm506862 A G RGS14 9 18

5 176812816 exm506913 A G SLC34A1 0 3

5 176813450 exm506936 A C SLC34A1 0 2

5 176821194 exm506979 A G SLC34A1 3 5

5 176936490 exm507617 C G DOK3 3 3

5 176936531 exm507619 C A DOK3 0 3

5 177573274 exm508276 A G RMND5B 2 4

5 177574595 exm508287 A G RMND5B 4 10

5 178043935 exm508691 A T CLK4 2 6

5 178139907 exm508756 A C ZNF354A 27 47

5 178140214 exm508768 A C ZNF354A 6 10

5 178140619 exm508785 A G ZNF354A 6 27

5 178309896 exm508832 G A ZNF354B 0 1

5 178310073 exm508839 G A ZNF354B 2 3

5 178311021 exm508881 A G ZNF354B 8 17

5 178359180 exm508934 G A ZFP2 7 14

5 178359353 exm508946 G A ZFP2 4 14

5 178359386 exm508948 A G ZFP2 2 7

5 178408704 exm509020 A G GRM6 4 14

5 178410213 exm509060 G A GRM6 11 32

5 178413234 exm509074 A G GRM6 1 1

5 178413688 exm509139 A G GRM6 1 2

5 178416277 exm509187 A G GRM6 2 2

5 178418946 exm509214 G A GRM6 5 9

5 178505836 exm509286 A G ZNF354C 21 37

5 178507004 exm509330 G A ZNF354C 14 23

5 178507048 exm509334 C G ZNF354C 28 64

5 179195867 exm509938 A G MAML1 10 24

5 179201214 exm509965 A G MAML1 6 13

5 179201298 exm509969 A G MAML1 3 4

5 179260099 exm510210 G C SQSTM1 9 25

5 179264633 exm510279 C G MRNIP 2 19

5 179264666 exm510281 A G MRNIP 9 22

5 179275051 exm510320 C A MRNIP 7 30

5 179280392 exm510325 A G MRNIP 25 49

5 179290537 exm510340 A C TBC1D9B 1 6

5 179306653 exm510476 C G TBC1D9B 11 24

5 179393923 exm510562 A G RNF130 8 22

5 179393926 exm510564 A G RNF130 11 29

5 179407158 exm510577 G A RNF130 0 1

5 180030221 exm510978 A G FLT4 18 39

5 180039606 exm511027 A G FLT4 12 21

5 180055936 exm511164 T A FLT4 25 44

5 180166985 exm511287 A C OR2Y1 12 14

5 180326326 exm511409 G A BTNL8 6 12

5 180335697 exm511422 A G BTNL8 8 13

5 180338426 exm511444 A G BTNL8 7 13

5 180338468 exm511449 A G BTNL8 4 9

5 180338584 exm511455 A G BTNL8 2 2

5 180431750 exm511553 A G BTNL3 3 8

5 180432813 exm511580 A G BTNL3 14 27

5 180472512 exm511587 A G BTNL9 16 29

5 154395682 JHU_5.154395681 A G KIF4B 20 32

5 180057293 JHU_5.180057292 G A FLT4 14 17

5 66492412 JHU_5.66492411 C G CD180 29 48

5 71479599 JHU_5.71479598 A G MAP1B 16 26

5 79026434 JHU_5.79026433 A G CMYA5 0 1

5 112128144 Variant63519 A G APC 3 1

6 105776785 6:105776785-CT A G PREP 1 1

6 112397149 6:112397149-TC G A TUBE1 1 1

6 112480078 6:112480078-GA A G LAMA4 1 0

6 116836860 6:116836860-GA A G CALHM5 7 19

6 117641041 6:117641041-AG G A ROS1 0 1

6 117706959 6:117706959-GT A C ROS1 0 1

6 129621997 6:129621997-AG G A LAMA2 3 11

6 132860260 6:132860260-GA A G TAAR9 7 15

6 132860431 6:132860431-TC G A TAAR9 5 11

6 132910626 6:132910626-GA A G TAAR5 2 3

6 133044958 6:133044958-GA A G VNN3 4 5

6 133045881 6:133045881-GA A G VNN3 0 3

6 133047897 6:133047897-CT A G VNN3 1 0

6 133078563 6:133078563-GT A C VNN2 22 51

6 133118216 6:133118216-AG G A SLC18B1 26 45

6 135358439 6:135358439-TC G A HBS1L 9 12

6 137519611 6:137519611-CT A G IFNGR1 1 1

6 138753018 6:138753018-CT A G NHSL1 11 22

6 138754299 6:138754299-TC G A NHSL1 11 22

6 142704947 6:142704947-CA A C ADGRG6 2 2

6 142726832 6:142726832-TC G A ADGRG6 4 14

6 146987957 6:146987957-AG G A ADGB 18 24

6 147073671 6:147073671-CT A G ADGB 8 19

6 147599289 6:147599289-AG G A STXBP5 1 4

6 151198779 6:151198779-GA A G MTHFD1L 9 17

6 151670035 6:151670035-TC G A AKAP12 7 7

6 151672795 6:151672795-CT A G AKAP12 1 2

6 151673382 6:151673382-GA A G AKAP12 3 5

6 152545681 6:152545681-CT A G SYNE1 1 5

6 152694285 6:152694285-TC G A SYNE1 0 2

6 154360852 6:154360852-TC G A OPRM1 6 5

6 158540110 6:158540110-AG G A SERAC1 10 15

6 158579334 6:158579334-GA A G SERAC1 14 34

6 158923416 6:158923416-GA A G TULP4 0 2

6 159464639 6:159464639-TG C A TAGAP 1 4

6 160231083 6:160231083-TC G A PNLDC1 19 40

6 160494969 6:160494969-GA A G IGF2R 3 5

6 160525770 6:160525770-CT A G IGF2R 2 3

6 167728873 6:167728873-CT A G UNC93A 8 17

6 168352152 6:168352152-CT A G AFDN 5 8

6 168440828 6:168440828-CT A G KIF25 2 6

6 168462534 6:168462534-CT A G FRMD1 0 2

6 170700107 6:170700107-CT A G FAM120B 12 27

6 170846369 6:170846369-CA A C PSMB1 7 8

6 17765021 6:17765021-GA A G KIF13A 1 1

6 20490428 6:20490428-GA A G E2F3 0 3

6 24302055 6:24302055-GA A G DCDC2 0 1

6 24810007 6:24810007-GA A G RIPOR2 1 2

6 26056173 6:26056173-CT A G HIST1H1C 1 0

6 26408177 6:26408177-GA A G BTN3A1 3 6

6 26459732 6:26459732-AG G A BTN2A1 6 10

6 27278469 6:27278469-AG G A POM121L2 0 3

6 27279933 6:27279933-CT A G POM121L2 1 4

6 28227810 6:28227810-AG G A NKAPL 0 4

6 29455534 6:29455534-CA A C MAS1L 14 26

6 30627867 6:30627867-TC G A DHX16 7 18

6 30883962 6:30883962-GA A G VARS2 10 33

6 30916828 6:30916828-TC G A MUCL3 2 2

6 3104474 6:3104474-GA A G RIPK1 12 24

6 31079606 6:31079606-CA A C C6orf15 3 4

6 31378864 6:31378864-AG G A MICA 12 9

6 31608463 6:31608463-TC G A BAG6 0 7

6 31640015 6:31640015-TG C A LY6G5B 1 5

6 32083614 6:32083614-GA A G ATF6B 2 2

6 32171576 6:32171576-GA A G NOTCH4 1 0

6 32188224 6:32188224-GA A G NOTCH4 0 3

6 32190344 6:32190344-CT A G NOTCH4 6 6

6 32261764 6:32261764-GA A G TSBP1 6 22

6 33173457 6:33173457-CT A G HSD17B8 4 19

6 33174418 6:33174418-GA A G HSD17B8 7 20

6 35705851 6:35705851-CT A G ARMC12 4 9

6 35745671 6:35745671-AG G A CLPSL2 7 14

6 35763566 6:35763566-GA A G CLPS 0 1

6 36169294 6:36169294-GA A G BRPF3 1 1

6 38704991 6:38704991-TC G A DNAH8 0 1

6 39874329 6:39874329-CT A G MOCS1 5 10

6 4077614 6:4077614-AG G A FAM217A 4 12

6 41754047 6:41754047-CT A G PRICKLE4 3 10

6 41900430 6:41900430-AG G A BYSL 5 9

6 42096435 6:42096435-CT A G C6orf132 5 6

6 42937470 6:42937470-GT A C PEX6 1 4

6 42941790 6:42941790-TC G A PEX6 5 7

6 43307255 6:43307255-TC G A ZNF318 1 1

6 43309882 6:43309882-TC G A ZNF318 14 29

6 43412898 6:43412898-AG G A ABCC10 1 2

6 44272267 6:44272267-CT A G AARS2 1 5

6 44336198 6:44336198-CA A C SPATS1 1 6

6 46047619 6:46047619-CT A G CLIC5 1 3

6 46656591 6:46656591-CA A C TDRD6 1 1

6 46658560 6:46658560-GA A G TDRD6 1 4

6 46659052 6:46659052-TG C A TDRD6 5 6

6 46661788 6:46661788-TG C A TDRD6 1 1

6 46851856 6:46851856-CT A G ADGRF5 1 1

6 47646748 6:47646748-TC G A ADGRF2 12 30

6 49700983 6:49700983-AC C A CRISP3 1 5

6 54095704 6:54095704-GA A G MLIP 0 1

6 54804632 6:54804632-AG G A FAM83B 15 22

6 54805891 6:54805891-AG G A FAM83B 5 15

6 56500405 6:56500405-GT A C DST 2 6

6 64404534 6:64404534-GA A G PHF3 11 23

6 65300194 6:65300194-GA A G EYS 3 7

6 65300425 6:65300425-CT A G EYS 7 23

6 65622479 6:65622479-GA A G EYS 4 6

6 656898 6:656898-AC C A HUS1B 3 8

6 66063381 6:66063381-AG G A EYS 3 9

6 70409074 6:70409074-AG G A LMBRD1 6 8

6 71004213 6:71004213-CT A G COL9A1 6 6

6 72922889 6:72922889-GA A G RIMS1 2 12

6 75834054 6:75834054-TC G A COL12A1 3 8

6 75855119 6:75855119-TC G A COL12A1 2 3

6 8064552 6:8064552-TC G A BLOC1S5 0 6

6 80749459 6:80749459-AG G A TTK 0 3

6 84290270 6:84290270-CA A C SNAP91 0 1

6 89859120 6:89859120-TC G A PM20D2 0 1

6 90042897 6:90042897-TC G A UBE2J1 9 19

6 90367954 6:90367954-CT A G MDN1 2 1

6 90576853 6:90576853-AC C A CASP8AP2 11 17

6 32303692 exm-rs1265754 A T TSBP1 8 14

6 107780213 exm2107788 A G PDSS2 0 1

6 109767557 exm2108236 A G MICAL1 7 12

6 129802475 exm2110710 A G LAMA2 20 47

6 147685274 exm2113348 G A STXBP5 3 4

6 151914299 exm2113984 A G CCDC170 11 23

6 24429297 exm2118003 G C GPLD1 2 3

6 32085655 exm2121766 G A ATF6B 1 0

6 4069388 exm2125273 G A FAM217A 9 19

6 42713409 exm2125990 C A TBCC 0 1

6 43414035 exm2126817 A G ABCC10 8 13

6 49663608 exm2128131 A G CRISP2 2 5

6 71011736 exm2130162 A G COL9A1 3 6

6 99848970 exm2133391 A G PNISR 19 40

6 151247337 exm2257597 G A MTHFD1L 5 15

6 151626987 exm2275917 A G AKAP12 0 1

6 152631566 exm2275919 A G SYNE1 2 4

6 39325072 exm2276871 A G KIF6 1 0

6 73017069 exm2277328 A G RIMS1 8 10

6 656473 exm512356 A G HUS1B 17 27

6 2766560 exm512738 A G WRNIP1 0 3

6 2779708 exm512787 G A WRNIP1 5 16

6 2785311 exm512806 G A WRNIP1 18 35

6 2785484 exm512819 C G WRNIP1 2 4

6 2893754 exm512940 G A SERPINB9 7 11

6 2900695 exm512952 A G SERPINB9 3 6

6 3010337 exm513041 G A NQO2 1 0

6 3010340 exm513042 G A NQO2 3 11

6 3012778 exm513056 A G NQO2 4 12

6 3015818 exm513064 A G NQO2 1 2

6 3017160 exm513070 A G NQO2 1 1

6 3077184 exm513104 A T RIPK1 3 6

6 3083337 exm513123 A G RIPK1 2 5

6 3106237 exm513168 G A RIPK1 0 5

6 3129333 exm513220 A C BPHL 20 36

6 3137725 exm513231 C A BPHL 3 9

6 3140743 exm513240 A G BPHL 1 1

6 3152763 exm513244 A G BPHL 4 6

6 4037656 exm513568 G A PRPF4B 5 26

6 4037762 exm513574 A G PRPF4B 0 4

6 4068932 exm513626 G A FAM217A 9 16

6 4069048 exm513632 C A FAM217A 7 14

6 4073519 exm513675 G A FAM217A 11 21

6 4996460 exm513937 A G RPP40 1 3

6 5000840 exm513966 A T RPP40 10 31

6 5369309 exm514036 A T FARS2 12 26

6 5545479 exm514066 G A FARS2 2 7

6 6174908 exm514159 A G F13A1 8 25

6 6266854 exm514211 A G F13A1 2 1

6 7182280 exm514308 A G RREB1 3 8

6 7211164 exm514334 A C RREB1 6 10

6 7229549 exm514370 A G RREB1 5 9

6 7231220 exm514475 A G RREB1 3 13

6 7231237 exm514477 A G RREB1 20 33

6 7373406 exm514683 G A CAGE1 15 29

6 7373434 exm514684 G C CAGE1 4 15

6 7404738 exm514769 A G RIOK1 12 29

6 7405508 exm514787 A G RIOK1 0 1

6 7417658 exm514822 A G RIOK1 6 16

6 7562954 exm514858 A G DSP 1 5

6 7565727 exm514876 A T DSP 2 4

6 7575687 exm514947 A G DSP 14 30

6 7580912 exm515027 A G DSP 6 10

6 7583814 exm515107 G C DSP 0 1

6 7845478 exm515328 A G BMP6 9 30

6 7862555 exm515340 A G BMP6 0 1

6 7888987 exm515403 G A TXNDC5 5 13

6 7888990 exm515404 A G TXNDC5 4 15

6 7899909 exm515440 C A TXNDC5 5 12

6 8015926 exm515475 G A BLOC1S5 0 5

6 10557417 exm515854 G A GCNT2 2 3

6 10587057 exm515897 A G GCNT2 3 7

6 10702799 exm515961 A G PAK1IP1 0 1

6 10709190 exm515977 G A PAK1IP1 7 14

6 10709590 exm515986 G A PAK1IP1 4 5

6 10877548 exm516218 C G GCM2 21 36

6 10877559 exm516219 A G GCM2 21 37

6 10882006 exm516229 C G GCM2 8 16

6 10894336 exm516252 C A SYCP2L 12 22

6 10935327 exm516332 A G SYCP2L 4 8

6 10942931 exm516342 G A SYCP2L 3 13

6 11005622 exm516405 A G ELOVL2 1 1

6 11005676 exm516407 C G ELOVL2 8 16

6 11190436 exm516543 A C NEDD9 5 12

6 11190591 exm516553 G A NEDD9 1 7

6 11191291 exm516584 A C NEDD9 9 20

6 11191425 exm516590 A G NEDD9 2 7

6 12120290 exm516721 A G HIVEP1 11 30

6 12121653 exm516773 G A HIVEP1 2 3

6 12123266 exm516839 A G HIVEP1 1 4

6 12124240 exm516883 A C HIVEP1 1 6

6 12124584 exm516901 G A HIVEP1 1 3

6 12125507 exm516942 A G HIVEP1 4 8

6 12125934 exm516965 C G HIVEP1 4 16

6 12163831 exm517021 G A HIVEP1 3 8

6 13316853 exm517220 A G TBC1D7;TBC1D7-LOC100130357 3 5

6 13595765 exm517372 C A SIRT5 3 13

6 13612060 exm517406 G A SIRT5 5 16

6 15523448 exm517894 A G DTNBP1 11 23

6 16143437 exm517987 G C MYLIP 10 18

6 16145315 exm518004 A G MYLIP 1 0

6 16327335 exm518171 A G ATXN1 15 28

6 16328447 exm518278 G C ATXN1 17 35

6 17616926 exm518492 G C NUP153 1 7

6 17629141 exm518526 A G NUP153 0 3

6 17646360 exm518609 G A NUP153 3 8

6 17764465 exm518705 A G KIF13A 0 1

6 17764534 exm518709 A G KIF13A 6 22

6 17764812 exm518723 A C KIF13A 12 36

6 17855690 exm518891 A G KIF13A 3 20

6 17873630 exm518898 C A KIF13A 4 9

6 24178676 exm519629 A G DCDC2 3 9

6 24278382 exm519651 A G DCDC2 1 5

6 24289124 exm519660 C A DCDC2 10 32

6 24445774 exm519831 A G GPLD1 1 4

6 24505196 exm520033 A C ALDH5A1 6 16

6 24528249 exm520060 A G ALDH5A1 9 7

6 24528331 exm520067 G A ALDH5A1 1 1

6 24532351 exm520073 A G ALDH5A1 13 23

6 24533753 exm520079 C A ALDH5A1 0 1

6 24572893 exm520167 G A KIAA0319 11 14

6 24576643 exm520176 C G KIAA0319 4 8

6 24576771 exm520181 A T KIAA0319 8 6

6 24777517 exm520402 C A GMNN 5 6

6 24781753 exm520411 G A GMNN 16 26

6 24825482 exm520440 A G RIPOR2 2 7

6 25606312 exm520702 G C CARMIL1 1 0

6 25619804 exm520722 G A CARMIL1 1 2

6 25653628 exm520732 T A SCGN 5 13

6 25819762 exm521005 A G SLC17A1 0 4

6 25826764 exm521023 A C SLC17A1 1 1

6 26056053 exm521569 A G HIST1H1C 26 39

6 26056129 exm521580 A C HIST1H1C 5 12

6 26056604 exm521676 A G HIST1H1C 3 4

6 26107873 exm521802 G A HIST1H1T 1 1

6 26107877 exm521803 G A HIST1H1T 0 2

6 26108117 exm521830 A G HIST1H1T 4 8

6 26108245 exm521848 A G HIST1H1T 3 3

6 26216843 exm522273 A G HIST1H2BG 19 28

6 26234665 exm522366 A G HIST1H1D 6 7

6 26234797 exm522384 G A HIST1H1D 4 6

6 26385257 exm522701 A G BTN2A2 6 19

6 26388372 exm522722 A C BTN2A2 10 11

6 26392629 exm522755 A G BTN2A2 28 44

6 26392917 exm522774 A T BTN2A2 6 11

6 26409890 exm522836 G C BTN3A1 2 1

6 26449895 exm523041 A G BTN3A3 13 20

6 26452349 exm523075 A G BTN3A3 4 9

6 26452389 exm523077 G A BTN3A3 0 9

6 26452619 exm523087 A G BTN3A3 6 9

6 26459864 exm523107 A C BTN2A1 3 3

6 26545621 exm523316 A G HMGN4 1 1

6 26545625 exm523317 C A HMGN4 1 4

6 26597411 exm523337 A C ABT1 6 20

6 26598159 exm523340 G A ABT1 4 24

6 27215709 exm523577 A G PRSS16 11 31

6 27215814 exm523583 A G PRSS16 0 2

6 27216987 exm523594 A G PRSS16 0 1

6 27277503 exm523663 A G POM121L2 17 37

6 27277521 exm523664 A C POM121L2 2 10

6 27278634 exm523688 C G POM121L2 1 0

6 27279632 exm523707 A C POM121L2 0 2

6 27879055 exm524307 A T OR2B2 1 5

6 27925394 exm524369 G A OR2B6 2 0

6 27925784 exm524388 A G OR2B6 3 9

6 28094610 exm524458 A G ZSCAN16 10 22

6 28094631 exm524459 A C ZSCAN16 1 1

6 28097720 exm524491 A G ZSCAN16 15 31

6 28117331 exm524507 A G ZKSCAN8 13 36

6 28121790 exm524554 A G ZKSCAN8 7 13

6 28194963 exm524574 G A ZSCAN9 16 35

6 28195278 exm524583 G A ZSCAN9 3 3

6 28227388 exm524725 T A NKAPL 7 21

6 28227819 exm524746 G A NKAPL 2 4

6 28251610 exm524805 A G PGBD1 5 18

6 28251663 exm524808 G A PGBD1 13 29

6 28254927 exm524833 A T PGBD1 3 2

6 28269321 exm524876 G A PGBD1 6 10

6 28269871 exm524902 A G PGBD1 5 7

6 28472266 exm525139 A G GPX6 2 2

6 28473521 exm525141 A G GPX6 12 34

6 28542491 exm525265 C A ZBED9 0 1

6 28547085 exm525314 C A ZBED9 2 5

6 28964249 exm525440 A G ZNF311 12 35

6 29323837 exm525770 G A OR5V1 1 14

6 29364508 exm525837 G A OR12D2 1 2

6 29365331 exm525883 A C OR12D2 12 30

6 29407961 exm525942 A G OR10C1 10 23

6 29430388 exm526033 C G OR2H1 20 35

6 29454769 exm526062 G A MAS1L 0 1

6 29454836 exm526064 A G MAS1L 2 6

6 29454951 exm526070 T A MAS1L 0 2

6 29455208 exm526081 A G MAS1L 3 9

6 29641329 exm526395 A G ZFP57 8 11

6 29692810 exm526451 A C HLA-F 0 1

6 30078262 exm527077 G A TRIM31 10 9

6 30080582 exm527119 G A TRIM31 0 5

6 30114877 exm527163 A G TRIM40 5 13

6 30114897 exm527164 A G TRIM40 27 51

6 30131585 exm527308 A G TRIM15 3 2

6 30131711 exm527311 G C TRIM15 4 12

6 30136302 exm527331 C G TRIM15 2 2

6 30308326 exm527521 G A TRIM39;TRIM39-RPP21 4 15

6 30309530 exm527525 A C TRIM39;TRIM39-RPP21 17 36

6 30617680 exm528098 A G C6orf136 1 7

6 30619137 exm528106 A G C6orf136 5 20

6 30671229 exm528409 A G MDC1 24 44

6 30671760 exm528428 A G MDC1 29 43

6 30671969 exm528438 G C MDC1 29 43

6 30682856 exm528656 A G MDC1 1 2

6 30856555 exm528774 G A DDR1 31 38

6 30865215 exm528889 C G DDR1 2 4

6 30883589 exm529014 G A VARS2 11 26

6 30919561 exm529250 G A MUCL3 6 6

6 31079951 exm529692 G A C6orf15 7 19

6 31084034 exm529728 A G CDSN 2 3

6 31084856 exm529774 G A CDSN 20 39

6 31085286 exm529802 A G CDSN 8 13

6 31105928 exm529835 A G PSORS1C2 9 17

6 31106236 exm529846 A G PSORS1C1 22 41

6 31557381 exm531154 A G NCR3 9 14

6 31557640 exm531163 A G NCR3 3 5

6 31595926 exm531303 A G PRRC2A 0 2

6 31599370 exm531349 G C PRRC2A 17 22

6 31604074 exm531493 G A PRRC2A 11 18

6 31605016 exm531519 G A PRRC2A 29 56

6 31614286 exm531639 A G BAG6 3 12

6 31639950 exm531821 G A LY6G5B 14 22

6 31721166 exm532172 A C MSH5 0 3

6 31725978 exm532184 G C MSH5 3 5

6 31726056 exm532188 A G MSH5 2 12

6 31741049 exm532347 A C VWA7 8 11

6 31743959 exm532379 A G VWA7 6 7

6 31747510 exm532424 A G VARS 17 47

6 31760656 exm532535 A G VARS 0 7

6 31762990 exm532556 G C VARS 1 5

6 31831478 exm532778 G A SLC44A4 1 1

6 31842535 exm532874 G A SLC44A4 1 0

6 31842598 exm532878 T A SLC44A4 16 29

6 31915584 exm533229 C A CFB 3 12

6 31916231 exm533247 C A CFB 9 15

6 31927839 exm533378 G A SKIV2L 4 9

6 31928463 exm533395 G A SKIV2L 0 1

6 31931696 exm533454 A G SKIV2L 7 15

6 31931858 exm533465 A G SKIV2L 4 6

6 31935750 exm533508 A G SKIV2L 2 1

6 32029180 exm534134 A G TNXB 11 25

6 32032628 exm534170 G C TNXB 16 47

6 32036449 exm534217 A G TNXB 1 4

6 32036755 exm534226 G A TNXB 16 35

6 32049429 exm534294 A C TNXB 20 24

6 32052216 exm534307 A G TNXB 32 67

6 32056625 exm534349 A C TNXB 10 33

6 32065113 exm534431 A G TNXB 7 22

6 32087633 exm534500 G A ATF6B 2 5

6 32088850 exm534513 G A ATF6B 0 1

6 32095465 exm534536 A G ATF6B 3 10

6 32123494 exm534616 A G PPT2 5 9

6 32149219 exm534767 A G AGER 1 0

6 32151443 exm534819 A G AGER 3 6

6 32151458 exm534821 A G AGER 14 33

6 32182016 exm535073 A G NOTCH4 2 5

6 32184835 exm535088 A G NOTCH4 12 24

6 32185796 exm535099 A G NOTCH4 2 3

6 32187420 exm535106 A G NOTCH4 3 16

6 32190440 exm535168 A G NOTCH4 7 11

6 32190549 exm535176 T A NOTCH4 1 3

6 32261252 exm535230 C A TSBP1 8 14

6 32362521 exm535361 A C BTNL2 1 4

6 32363888 exm535389 A G BTNL2 1 5

6 32364011 exm535395 G A BTNL2 1 4

6 32364092 exm535402 G A BTNL2 5 35

6 32370927 exm535437 A G BTNL2 2 6

6 32370993 exm535447 A G BTNL2 6 13

6 32632833 exm536425 G A HLA-DQB1 2 4

6 32634282 exm536439 G A HLA-DQB1 2 4

6 32797773 exm536714 G A TAP2 10 16

6 32803109 exm536766 G A TAP2 0 3

6 32806007 exm536807 A G TAP2 17 30

6 32813421 exm536865 A C TAP1 4 12

6 32818242 exm536937 G A TAP1 23 34

6 32906586 exm537079 A G HLA-DMB 1 1

6 32906652 exm537081 A T HLA-DMB 1 1

6 32906716 exm537082 G A HLA-DMB 1 2

6 32917411 exm537102 A G HLA-DMA 1 1

6 32917498 exm537105 G C HLA-DMA 1 1

6 33043865 exm537472 A G HLA-DPB1 2 2

6 33048538 exm537495 G A HLA-DPB1 9 21

6 33048649 exm537521 A C HLA-DPB1 14 23

6 33052981 exm537555 A C HLA-DPB1 5 13

6 33131501 exm537585 A G COL11A2 1 5

6 33146726 exm537772 C G COL11A2 1 0

6 33156869 exm537837 A G COL11A2 10 18

6 33271606 exm538449 G C TAPBP 16 41

6 33271971 exm538457 A G TAPBP 11 19

6 33287520 exm538627 A G DAXX 2 10

6 33288285 exm538651 A G DAXX 2 20

6 33365808 exm538709 A C KIFC1 2 2

6 33371778 exm538731 A C KIFC1 3 3

6 33372980 exm538757 A G KIFC1 2 5

6 33384730 exm538884 C G CUTA 5 15

6 33384734 exm538885 A C CUTA 4 12

6 33589404 exm539164 A G ITPR3 1 6

6 33634942 exm539245 C G ITPR3 7 13

6 33638551 exm539289 A G ITPR3 1 1

6 33639821 exm539300 A C ITPR3 9 9

6 33647700 exm539363 T A ITPR3 11 30

6 33653486 exm539464 A G ITPR3 6 32

6 33653971 exm539475 A G ITPR3 1 2

6 33658853 exm539544 G C ITPR3 13 27

6 34803204 exm540310 A C UHRF1BP1 0 1

6 34824082 exm540326 G A UHRF1BP1 4 5

6 34824121 exm540329 G A UHRF1BP1 0 1

6 34826678 exm540386 A C UHRF1BP1 4 9

6 34826921 exm540400 G C UHRF1BP1 5 8

6 34827086 exm540409 A G UHRF1BP1 0 1

6 34838694 exm540442 A G UHRF1BP1 7 6

6 34850826 exm540479 G C TAF11 10 7

6 34855591 exm540482 G C TAF11 9 22

6 34855731 exm540487 G C TAF11 0 5

6 35210839 exm540809 A G SCUBE3 12 26

6 35210900 exm540816 A G SCUBE3 0 5

6 35210998 exm540824 A G SCUBE3 3 10

6 35211427 exm540827 A G SCUBE3 9 12

6 35212504 exm540842 A G SCUBE3 15 33

6 35467758 exm541301 A G TULP1 3 4

6 35467767 exm541303 A G TULP1 18 40

6 35467877 exm541309 G A TULP1 4 11

6 35471362 exm541317 A G TULP1 19 33

6 35477415 exm541353 G C TULP1 8 13

6 35477630 exm541364 A G TULP1 3 16

6 35478710 exm541382 G C TULP1 3 7

6 35704964 exm541471 A G ARMC12 3 4

6 35705894 exm541492 A C ARMC12 12 22

6 35715164 exm541514 G A ARMC12 22 49

6 35716319 exm541521 C A ARMC12 20 49

6 35716450 exm541532 A G ARMC12 8 9

6 35747207 exm541556 A G CLPSL2 2 5

6 35762937 exm541589 A G CLPS 4 5

6 35763017 exm541591 A G CLPS 3 7

6 35825141 exm541673 G A SRPK1 1 5

6 35837421 exm541681 G A SRPK1 14 11

6 36168628 exm542001 G A BRPF3 16 37

6 36259274 exm542145 A G PNPLA1 3 16

6 36263171 exm542175 A G PNPLA1 6 15

6 36287177 exm542240 A G BNIP5 0 3

6 36291180 exm542267 A G BNIP5 2 7

6 36293083 exm542282 A G BNIP5 2 4

6 36298073 exm542333 G A BNIP5 2 6

6 36343673 exm542413 A G ETV7 23 33

6 36651904 exm542617 A G CDKN1A 1 4

6 36652067 exm542625 A C CDKN1A 3 13

6 37186479 exm543188 A G TMEM217 0 1

6 37612408 exm543469 C G MDGA1 5 17

6 37631768 exm543562 G A MDGA1 3 9

6 38702364 exm543731 A G DNAH8 2 3

6 38704912 exm543736 G A DNAH8 18 29

6 38759353 exm543824 G A DNAH8 11 25

6 38795987 exm543882 A G DNAH8 1 5

6 38810634 exm543915 A T DNAH8 5 6

6 38818036 exm543928 A G DNAH8 9 23

6 38820530 exm543936 C G DNAH8 15 32

6 38840915 exm544021 G A DNAH8 21 50

6 38863970 exm544088 A G DNAH8 5 13

6 38877354 exm544115 G A DNAH8 10 25

6 38877423 exm544119 A T DNAH8 6 8

6 38891879 exm544176 A G DNAH8 1 3

6 38917231 exm544247 A T DNAH8 13 32

6 38957948 exm544301 A G DNAH8 9 14

6 39328259 exm544702 G A KIF6 1 0

6 39507949 exm544731 A C KIF6 4 13

6 39545926 exm544749 A G KIF6 9 17

6 39564011 exm544779 A G KIF6 5 8

6 39682532 exm544807 G A KIF6 4 15

6 39877665 exm545032 A G MOCS1 0 1

6 39880692 exm545044 A G MOCS1 9 10

6 39881102 exm545049 A T MOCS1 39 51

6 39883940 exm545074 A G MOCS1 13 25

6 40359728 exm545124 A G LRFN2 2 5

6 40400080 exm545219 A G LRFN2 3 9

6 40400195 exm545229 A G LRFN2 0 1

6 41000790 exm545307 A G UNC5CL 18 34

6 41001675 exm545321 A G UNC5CL 4 6

6 41001868 exm545334 A C UNC5CL 0 1

6 41010795 exm545356 A G TSPO2 5 9

6 41011370 exm545363 G A TSPO2 1 4

6 41162204 exm545551 A C TREML2 2 6

6 41166155 exm545598 G A TREML2 5 20

6 41738892 exm546125 A G FRS3 4 10

6 41740596 exm546166 G A FRS3 11 24

6 41752770 exm546208 A G PRICKLE4 6 13

6 41753105 exm546216 C G PRICKLE4 5 13

6 41766425 exm546256 C A USP49 15 18

6 41766441 exm546260 A G USP49 8 21

6 41889383 exm546392 A C BYSL 2 8

6 42073759 exm546601 A G C6orf132 0 1

6 42074872 exm546623 C A C6orf132 15 24

6 42141469 exm546660 A G GUCA1A 20 31

6 42141503 exm546664 A G GUCA1A 7 15

6 42141505 exm546665 A G GUCA1A 4 3

6 42146021 exm546669 A C GUCA1A 17 39

6 42203984 exm546809 A C TRERF1 1 2

6 42231219 exm546874 A G TRERF1 7 8

6 42231275 exm546884 G C TRERF1 23 41

6 42236094 exm546907 A G TRERF1 10 30

6 42237037 exm546949 A G TRERF1 2 3

6 42618068 exm547087 G A UBR2 3 15

6 42627467 exm547113 A G UBR2 5 7

6 42643812 exm547133 G A UBR2 0 1

6 42644551 exm547142 A G UBR2 4 8

6 42713307 exm547255 A G TBCC 0 1

6 42713809 exm547280 A G TBCC 0 1

6 42797152 exm547305 A G BICRAL 3 10

6 42832579 exm547365 G C BICRAL 4 10

6 42890933 exm547481 G A PTCRA 3 5

6 42893263 exm547513 A G PTCRA 7 8

6 42932835 exm547619 A G PEX6 8 20

6 42936039 exm547662 A C PEX6 6 10

6 42937685 exm547692 A G PEX6 11 25

6 42946204 exm547722 G A PEX6 11 30

6 43012613 exm548036 A G CUL7 20 35

6 43013989 exm548059 A G CUL7 18 36

6 43014697 exm548082 A G CUL7 4 6

6 43018872 exm548142 A G CUL7 7 19

6 43020391 exm548182 A G CUL7 8 16

6 43029288 exm548266 A G KLC4 0 1

6 43034864 exm548313 A C KLC4 10 30

6 43039980 exm548340 A G KLC4 0 2

6 43040700 exm548348 A G KLC4 5 11

6 43098393 exm548411 A G PTK7 10 29

6 43100231 exm548431 A G PTK7 25 34

6 43100339 exm548441 G A PTK7 3 6

6 43153786 exm548632 A G CUL9 4 3

6 43170558 exm548767 A G CUL9 0 7

6 43172498 exm548798 A C CUL9 2 5

6 43189433 exm548919 A G CUL9 5 21

6 43191017 exm548938 A G CUL9 1 1

6 43269348 exm549264 A G SLC22A7 5 6

6 43269420 exm549270 A G SLC22A7 0 1

6 43305393 exm549373 A G ZNF318 5 7

6 43323178 exm549545 A G ZNF318 7 15

6 43400691 exm549677 A G ABCC10 9 26

6 43400743 exm549678 G A ABCC10 6 6

6 43403513 exm549712 A G ABCC10 3 3

6 43418773 exm549926 A G DLK2 4 16

6 43422536 exm549961 A G DLK2 4 13

6 43484855 exm550188 A G POLR1C 11 21

6 43492353 exm550272 G A XPO5 20 28

6 43494464 exm550289 G A XPO5 18 31

6 43523626 exm550347 C G XPO5 7 21

6 43527979 exm550354 A G XPO5 5 8

6 43535018 exm550363 A G XPO5 13 18

6 43572467 exm550431 G C POLH 16 26

6 43578397 exm550435 G A POLH 1 3

6 43581724 exm550454 C A POLH 11 17

6 43581897 exm550461 A G POLH 3 12

6 44103069 exm550899 A G TMEM63B 5 9

6 44115169 exm550940 A G TMEM63B 10 14

6 44137700 exm551029 A G CAPN11 3 11

6 44145155 exm551078 A C CAPN11 25 52

6 44148711 exm551104 A G CAPN11 9 9

6 44248124 exm551491 A G TCTE1 7 19

6 44255337 exm551554 A G TCTE1 2 3

6 44255495 exm551569 G A TCTE1 2 6

6 44328296 exm551766 C A SPATS1 0 5

6 44921076 exm551904 G A SUPT3H 13 22

6 45289587 exm551940 A G SUPT3H 9 15

6 45882045 exm552055 C G CLIC5 8 15

6 46107343 exm552106 G A ENPP4 4 11

6 46107595 exm552112 A G ENPP4 5 22

6 46111330 exm552147 C A ENPP4 1 6

6 46135603 exm552194 C A ENPP5 3 16

6 46135885 exm552205 A G ENPP5 4 4

6 46623768 exm552339 A G SLC25A27 8 22

6 46626759 exm552342 A G SLC25A27 8 30

6 46638913 exm552367 A G SLC25A27 12 27

6 46657184 exm552441 A G TDRD6 5 14

6 46658044 exm552478 G A TDRD6 2 0

6 46659780 exm552569 C A TDRD6 10 17

6 46660316 exm552599 G A TDRD6 10 33

6 46660495 exm552609 A G TDRD6 13 31

6 46660627 exm552616 C G TDRD6 5 6

6 46660657 exm552618 A G TDRD6 5 6

6 46661021 exm552636 A G TDRD6 16 26

6 46661335 exm552646 G A TDRD6 9 22

6 46661488 exm552656 G A TDRD6 8 18

6 46661534 exm552662 G A TDRD6 0 2

6 46766883 exm552808 A C MEP1A 6 24

6 46800823 exm552852 G A MEP1A 6 7

6 46846043 exm553054 T A ADGRF5 14 30

6 46979756 exm553218 G A ADGRF1 0 3

6 46996760 exm553292 A G ADGRF1 10 17

6 47563669 exm553460 A C CD2AP 9 21

6 47576899 exm553485 A G CD2AP 3 7

6 47576937 exm553487 A G CD2AP 2 2

6 47649169 exm553543 G A ADGRF2 3 9

6 47650196 exm553585 G A ADGRF2 6 13

6 47650255 exm553587 G A ADGRF2 10 18

6 47654748 exm553600 A T ADGRF2 7 16

6 47682131 exm553663 A C ADGRF4 0 1

6 47682212 exm553676 A G ADGRF4 2 3

6 47682500 exm553697 A G ADGRF4 11 26

6 47686168 exm553744 A G ADGRF4 1 5

6 49409599 exm553941 A G MMUT 3 1

6 49421373 exm553976 A G MMUT 1 1

6 49426896 exm553994 A G MMUT 1 1

6 49663567 exm554154 G A CRISP2 5 20

6 49666215 exm554173 G A CRISP2 12 23

6 49701469 exm554215 A G CRISP3 4 8

6 49754021 exm554254 A G PGK2 7 9

6 49754446 exm554279 A G PGK2 7 17

6 49754534 exm554285 A G PGK2 12 40

6 49754822 exm554298 A G PGK2 11 19

6 51484077 exm554572 G C PKHD1 7 8

6 51484226 exm554580 A G PKHD1 3 7

6 51524036 exm554624 A G PKHD1 1 2

6 51524065 exm554625 A G PKHD1 7 12

6 51524714 exm554659 G A PKHD1 7 12

6 51720866 exm554795 A G PKHD1 0 1

6 51747934 exm554818 A G PKHD1 11 18

6 51771039 exm554843 G A PKHD1 7 17

6 51777285 exm554868 C A PKHD1 16 28

6 51889737 exm554928 A G PKHD1 3 6

6 51890770 exm554981 A G PKHD1 1 1

6 51910905 exm555045 G A PKHD1 1 1

6 51914991 exm555063 A G PKHD1 6 12

6 51918875 exm555082 A G PKHD1 2 8

6 51920485 exm555102 A G PKHD1 0 1

6 51921546 exm555107 G A PKHD1 1 0

6 51921714 exm555110 G A PKHD1 13 28

6 51923378 exm555131 A G PKHD1 3 5

6 51935819 exm555157 A G PKHD1 16 24

6 52303361 exm555481 A G EFHC1 2 4

6 52319056 exm555503 A G EFHC1 14 33

6 52329845 exm555516 A G EFHC1 16 27

6 52355117 exm555541 G A EFHC1 8 19

6 52357071 exm555544 C A EFHC1 1 2

6 52546620 exm555607 A G TMEM14A 0 2

6 52546658 exm555610 A G TMEM14A 0 2

6 52617692 exm555646 C A GSTA2 6 11

6 52617738 exm555648 A G GSTA2 2 2

6 52871155 exm555875 G A ICK 7 23

6 52874333 exm555882 A G ICK 5 19

6 53516572 exm556185 A G KLHL31 0 4

6 53517103 exm556209 G A KLHL31 4 12

6 54735051 exm556537 T A FAM83B 3 7

6 54792319 exm556555 A G FAM83B 12 22

6 54805966 exm556625 G A FAM83B 21 34

6 55039416 exm556668 A C HCRTR2 0 1

6 55120058 exm556686 G A HCRTR2 3 4

6 55196575 exm556727 C G GFRAL 2 6

6 55214895 exm556737 A G GFRAL 2 6

6 55214932 exm556738 A C GFRAL 2 6

6 55216194 exm556744 A G GFRAL 5 15

6 55216344 exm556756 G A GFRAL 2 6

6 55264227 exm556773 G A GFRAL 3 7

6 55381349 exm556816 A G HMGCLL1 17 37

6 55406577 exm556821 C A HMGCLL1 5 8

6 55684511 exm556873 G A BMP5 12 37

6 55939059 exm556957 A C COL21A1 5 19

6 56029268 exm556990 A G COL21A1 10 27

6 56033094 exm556999 A G COL21A1 12 24

6 56035494 exm557001 A G COL21A1 3 5

6 56342280 exm557089 G A DST 1 1

6 56401738 exm557228 A C DST 3 2

6 56418319 exm557275 A G DST 3 6

6 56420275 exm557280 C A DST 5 7

6 56481892 exm557686 A G DST 13 30

6 56483313 exm557723 A G DST 7 10

6 56483409 exm557728 G A DST 6 14

6 56483871 exm557748 A G DST 11 17

6 56484281 exm557764 C A DST 2 6

6 56484432 exm557772 A G DST 9 23

6 56484772 exm557791 A C DST 14 18

6 56485113 exm557805 G A DST 13 33

6 56917508 exm558038 A G KIAA1586 11 27

6 56917520 exm558040 G A KIAA1586 5 16

6 56919345 exm558108 G A KIAA1586 5 11

6 64394066 exm558595 G A PHF3 6 17

6 64413470 exm558700 C A PHF3 16 35

6 64422311 exm558741 G C PHF3 4 6

6 65300143 exm558872 G C EYS 15 16

6 65523385 exm558927 G C EYS 9 22

6 66044979 exm558988 A T EYS 7 17

6 66112473 exm559021 G A EYS 5 8

6 66115146 exm559027 A G EYS 15 48

6 66204585 exm559044 G A EYS 5 10

6 66204588 exm559045 A G EYS 1 1

6 69348796 exm559081 A G ADGRB3 16 25

6 69665952 exm559114 G A ADGRB3 7 17

6 69684657 exm559128 A C ADGRB3 1 0

6 69944925 exm559173 G A ADGRB3 12 36

6 70410677 exm559258 G A LMBRD1 0 1

6 70637860 exm559332 A G COL19A1 6 14

6 70639419 exm559340 G C COL19A1 7 21

6 70639431 exm559341 A G COL19A1 5 23

6 70672731 exm559372 C A COL19A1 7 21

6 70852699 exm559419 A G COL19A1 6 20

6 70866095 exm559468 A G COL19A1 9 31

6 70866220 exm559471 A G COL19A1 4 17

6 70866229 exm559473 A C COL19A1 6 18

6 70877936 exm559490 A G COL19A1 2 4

6 70916883 exm559546 A G COL19A1 2 12

6 70981371 exm559666 A G COL9A1 1 1

6 71195918 exm559820 C A FAM135A 10 15

6 71235363 exm559893 G A FAM135A 13 14

6 71269470 exm559954 A G FAM135A 21 37

6 72984123 exm560271 A G RIMS1 11 11

6 73933476 exm560431 A G KHDC1L 3 7

6 73934059 exm560440 G C KHDC1L 12 30

6 74104789 exm560582 A G DDX43 11 35

6 74115413 exm560604 T A DDX43 11 24

6 74331619 exm560907 A G SLC17A5 28 40

6 74348195 exm560924 G A SLC17A5 16 38

6 74407128 exm560951 A G CD109 15 28

6 74473314 exm561005 G A CD109 21 38

6 74475690 exm561016 G A CD109 20 34

6 74475716 exm561017 G A CD109 29 49

6 74475803 exm561022 G A CD109 7 11

6 74476701 exm561029 A G CD109 21 35

6 74497005 exm561077 C A CD109 8 18

6 74497021 exm561079 A G CD109 1 8

6 74497126 exm561083 A G CD109 10 26

6 75797410 exm561180 A G COL12A1 7 13

6 75818844 exm561226 G A COL12A1 2 7

6 75822985 exm561231 A G COL12A1 2 3

6 75825507 exm561236 A G COL12A1 1 5

6 75834971 exm561279 A T COL12A1 10 12

6 75840573 exm561293 A G COL12A1 12 28

6 75843603 exm561316 A G COL12A1 11 23

6 75844564 exm561331 G A COL12A1 7 15

6 75887459 exm561476 A G COL12A1 1 2

6 75893140 exm561506 C G COL12A1 1 2

6 76022319 exm561650 A G FILIP1 6 9

6 76024124 exm561710 A G FILIP1 3 3

6 76024704 exm561733 A G FILIP1 1 0

6 76369083 exm561818 C G SENP6 5 8

6 76412531 exm561872 A G SENP6 13 35

6 76412776 exm561883 G A SENP6 4 11

6 76564921 exm561935 A G MYO6 14 28

6 76623870 exm562031 A G MYO6 4 13

6 76640787 exm562061 G A IMPG1 6 17

6 76731853 exm562147 A G IMPG1 0 1

6 76782204 exm562176 A T IMPG1 1 6

6 79655763 exm562300 C A PHIP 2 5

6 79665367 exm562322 G A PHIP 1 0

6 80196789 exm562454 A C LCA5 15 25

6 80196972 exm562460 G A LCA5 3 6

6 80202304 exm562512 G C LCA5 3 12

6 80223128 exm562530 A G LCA5 0 1

6 80724261 exm562662 C G TTK 11 29

6 80732172 exm562673 C G TTK 1 1

6 80745051 exm562705 G A TTK 4 11

6 80749554 exm562717 A G TTK 12 29

6 83667103 exm563048 C G UBE3D 8 24

6 83667138 exm563052 A G UBE3D 3 12

6 83838673 exm563164 A G DOP1A 1 6

6 83841928 exm563182 A C DOP1A 1 5

6 83847495 exm563226 A G DOP1A 6 8

6 83847590 exm563230 G A DOP1A 1 3

6 83847699 exm563233 G C DOP1A 1 3

6 83848222 exm563248 A G DOP1A 3 10

6 83891624 exm563366 C A PGM3 0 1

6 83900554 exm563386 G A PGM3 1 6

6 84291977 exm563567 A G SNAP91 21 34

6 84350864 exm563632 A C SNAP91 3 1

6 84862669 exm563816 G A CEP162 3 13

6 84862690 exm563817 A T CEP162 7 25

6 84865049 exm563827 A G CEP162 14 34

6 84871572 exm563845 A G CEP162 8 16

6 84881338 exm563855 A G CEP162 13 17

6 84925563 exm563961 C G CEP162 7 19

6 86223935 exm564159 A G SNX14 1 6

6 86256863 exm564194 A C SNX14 7 23

6 87966277 exm564429 A G ZNF292 6 14

6 87966936 exm564458 C G ZNF292 2 9

6 87968085 exm564507 A G ZNF292 2 8

6 87971020 exm564609 G A ZNF292 4 1

6 88123610 exm564717 A G CFAP206 1 0

6 88125541 exm564725 A G CFAP206 10 22

6 88136331 exm564752 A T CFAP206 1 3

6 88138466 exm564761 C A CFAP206 12 17

6 88173747 exm564780 A G CFAP206 14 36

6 88231226 exm564879 G A RARS2 6 5

6 88240570 exm564903 A G RARS2 2 2

6 88317511 exm564990 A G ORC3 4 5

6 88321832 exm564997 G A ORC3 13 26

6 88321953 exm565002 A G ORC3 5 19

6 88346196 exm565022 A C ORC3 1 8

6 89868128 exm565382 G A PM20D2 24 34

6 89871861 exm565390 A G PM20D2 4 16

6 89967489 exm565485 A G GABRR2 0 6

6 89967675 exm565491 G A GABRR2 11 28

6 89975365 exm565513 A G GABRR2 0 1

6 89977454 exm565527 C A GABRR2 1 7

6 90039669 exm565585 C A UBE2J1 9 20

6 90340446 exm565747 A G ANKRD6 28 57

6 90340454 exm565749 G A ANKRD6 15 31

6 90354856 exm565797 G C MDN1 7 11

6 90362818 exm565826 G C MDN1 1 5

6 90368348 exm565859 T A MDN1 2 11

6 90372574 exm565890 C A MDN1 28 57

6 90372634 exm565891 C G MDN1 7 10

6 90383916 exm565940 A G MDN1 3 12

6 90399835 exm566043 G A MDN1 11 12

6 90402844 exm566073 A G MDN1 1 2

6 90403824 exm566080 A G MDN1 2 13

6 90403833 exm566081 G C MDN1 0 3

6 90405377 exm566092 A G MDN1 12 30

6 90417176 exm566166 A G MDN1 15 31

6 90422459 exm566187 G A MDN1 15 34

6 90424391 exm566197 C G MDN1 5 7

6 90437650 exm566263 G C MDN1 4 6

6 90442330 exm566279 A G MDN1 4 7

6 90448116 exm566288 A G MDN1 1 0

6 90455049 exm566313 G A MDN1 6 13

6 90461235 exm566365 G A MDN1 17 39

6 90494813 exm566436 A G MDN1 10 22

6 90504414 exm566462 A G MDN1 2 3

6 90513051 exm566468 A G MDN1 4 7

6 90572732 exm566526 A G CASP8AP2 10 18

6 90576005 exm566575 G A CASP8AP2 10 22

6 90576667 exm566583 A C CASP8AP2 5 20

6 90605664 exm566746 A G GJA10 0 3

6 90642235 exm566759 C G BACH2 1 8

6 90660090 exm566792 G A BACH2 1 6

6 90660398 exm566806 A C BACH2 1 3

6 90660863 exm566827 A G BACH2 2 4

6 90660954 exm566830 C G BACH2 4 7

6 90661356 exm566848 A G BACH2 5 4

6 90661373 exm566851 C G BACH2 8 11

6 94120219 exm567000 A G EPHA7 0 3

6 94120639 exm567010 G A EPHA7 0 3

6 96034857 exm567046 G A MANEA 9 22

6 96053815 exm567066 G A MANEA 1 0

6 96053922 exm567072 A T MANEA 23 35

6 96053941 exm567073 G A MANEA 2 6

6 97051593 exm567227 A G FHL5 23 44

6 97052644 exm567231 A G FHL5 19 33

6 97414949 exm567347 A G KLHL32 8 17

6 97587028 exm567404 A T KLHL32 2 1

6 97599702 exm567422 A G MMS22L 11 17

6 97613263 exm567441 G A MMS22L 7 10

6 97621027 exm567453 C A MMS22L 16 45

6 97627380 exm567468 A G MMS22L 4 10

6 97677080 exm567500 G A MMS22L 8 12

6 97694509 exm567525 A G MMS22L 4 10

6 97730335 exm567569 C G MMS22L 11 12

6 99848550 exm567790 G A PNISR 6 28

6 99893693 exm567907 G A USP45 11 27

6 99893698 exm567908 A C USP45 11 27

6 99894201 exm567935 G A USP45 2 2

6 99894321 exm567939 A G USP45 3 23

6 99956505 exm567982 A G USP45 4 23

6 99958057 exm567994 A G USP45 2 7

6 100838674 exm568182 G A SIM1 3 5

6 100868721 exm568218 A G SIM1 34 64

6 100868779 exm568222 A C SIM1 30 63

6 100960590 exm568270 A G ASCC3 10 23

6 101090504 exm568359 G A ASCC3 10 22

6 101103660 exm568409 C G ASCC3 12 27

6 101163360 exm568430 G A ASCC3 11 25

6 101215148 exm568454 C A ASCC3 4 9

6 101215184 exm568456 G A ASCC3 4 6

6 101247393 exm568471 A G ASCC3 17 31

6 105726242 exm568859 G C PREP 0 1

6 106552846 exm568964 G A PRDM1 19 37

6 106553314 exm568995 A G PRDM1 2 5

6 106960245 exm569073 A G CRYBG1 20 34

6 106967358 exm569118 A G CRYBG1 19 35

6 106967482 exm569122 G A CRYBG1 1 4

6 106967554 exm569124 G A CRYBG1 6 17

6 106968205 exm569184 G A CRYBG1 19 35

6 106968600 exm569207 A G CRYBG1 1 3

6 106969027 exm569233 G A CRYBG1 0 3

6 106975290 exm569267 G A CRYBG1 6 11

6 106992504 exm569301 A G CRYBG1 2 6

6 107001385 exm569329 A G CRYBG1 1 1

6 107003686 exm569335 A G CRYBG1 1 0

6 107102618 exm569486 A G QRSL1 5 14

6 107103496 exm569493 A G QRSL1 4 4

6 107110988 exm569504 A G QRSL1 4 7

6 107780483 exm569745 G A PDSS2 4 10

6 108616303 exm570080 G A AFG1L 4 18

6 108677977 exm570114 A G AFG1L 32 58

6 108831424 exm570147 A G AFG1L 13 29

6 109471351 exm570459 G C CEP57L1 13 23

6 109481786 exm570490 G A CEP57L1 0 3

6 109764814 exm570683 G C SMPD2 0 1

6 109766710 exm570736 A G MICAL1 0 2

6 109767059 exm570743 G A MICAL1 19 33

6 109769085 exm570796 A G MICAL1 5 16

6 109772822 exm570868 A G MICAL1 0 1

6 109787446 exm570950 A G ZBTB24 6 10

6 109863301 exm571098 A C AK9 0 2

6 109954416 exm571190 C A AK9 0 1

6 109962806 exm571201 A G AK9 7 9

6 110059611 exm571278 A G FIG4 2 2

6 110062679 exm571282 G A FIG4 19 34

6 110422928 exm571405 A G WASF1 3 6

6 110423268 exm571420 A G WASF1 10 17

6 110536489 exm571483 C G CDC40 10 20

6 110567409 exm571513 A G METTL24 2 0

6 110620177 exm571518 G A METTL24 2 4

6 110714025 exm571561 A G DDO 1 6

6 110714126 exm571569 A G DDO 9 37

6 110714206 exm571578 A C DDO 4 12

6 110714357 exm571584 A G DDO 10 23

6 110714423 exm571590 A G DDO 2 6

6 110736706 exm571632 G A DDO 4 10

6 111587204 exm571993 G A MFSD4B 0 3

6 111588192 exm572030 A G MFSD4B 8 9

6 111688647 exm572136 C A REV3L 9 7

6 111688653 exm572137 G A REV3L 8 9

6 111693952 exm572161 A C REV3L 5 7

6 111695281 exm572225 G A REV3L 0 3

6 111695444 exm572235 G A REV3L 5 9

6 111695579 exm572238 G A REV3L 10 16

6 111695999 exm572264 A G REV3L 8 9

6 111697090 exm572312 A G REV3L 4 5

6 111699015 exm572351 G A REV3L 3 5

6 111896989 exm572431 A G TRAF3IP2 0 1

6 111912471 exm572439 G C TRAF3IP2 10 21

6 111913099 exm572465 C A TRAF3IP2 8 19

6 112382324 exm572563 A G CCN6 0 1

6 112386144 exm572578 T A CCN6 22 38

6 112393155 exm572609 A G TUBE1 3 15

6 112397170 exm572626 G A TUBE1 15 30

6 112397269 exm572631 A G TUBE1 2 8

6 112430662 exm572671 G A LAMA4 17 32

6 112457432 exm572751 A G LAMA4 1 2

6 112461988 exm572777 A G LAMA4 3 5

6 112479963 exm572829 A C LAMA4 2 1

6 112513042 exm572927 A G LAMA4 9 21

6 112537612 exm572949 G A LAMA4 3 5

6 112575027 exm572955 C G LAMA4 4 10

6 116264315 exm573180 A G FRK 17 27

6 116265462 exm573186 G A FRK 2 5

6 116289888 exm573215 A G FRK 5 4

6 116325108 exm573219 A G FRK 7 18

6 116439063 exm573273 G A NT5DC1 4 16

6 116441632 exm573294 A G COL10A1 0 3

6 116446613 exm573358 C A COL10A1 8 19

6 116559477 exm573379 G A NT5DC1 11 30

6 116599859 exm573442 G A TSPYL1 0 1

6 116600602 exm573481 A G TSPYL1 1 2

6 116600738 exm573488 A G TSPYL1 8 20

6 116757751 exm573571 G C DSE 9 16

6 116758282 exm573589 A G DSE 7 10

6 116758318 exm573590 C A DSE 0 2

6 116836916 exm573681 A G CALHM5 4 7

6 116837115 exm573690 A G CALHM5 18 26

6 116938093 exm573765 A C RSPH4A 2 4

6 116948950 exm573792 C G RSPH4A 3 13

6 116953554 exm573849 G C RSPH4A 6 9

6 117083172 exm573986 A G FAM162B 16 33

6 117083186 exm573988 G C FAM162B 0 1

6 117113382 exm574013 C G GPRC6A 5 5

6 117113976 exm574081 A G GPRC6A 14 32

6 117114123 exm574089 A G GPRC6A 1 4

6 117114290 exm574096 G A GPRC6A 20 53

6 117116965 exm574106 A G GPRC6A 9 29

6 117121767 exm574108 C A GPRC6A 21 39

6 117127724 exm574120 G A GPRC6A 5 10

6 117128107 exm574134 G A GPRC6A 0 2

6 117128185 exm574137 C A GPRC6A 1 0

6 117128308 exm574139 A G GPRC6A 13 37

6 117198581 exm574174 A G RFX6 3 6

6 117248673 exm574254 A G RFX6 12 19

6 117250009 exm574263 G C RFX6 1 6

6 117250076 exm574266 A C RFX6 3 7

6 117609714 exm574332 G C ROS1 3 6

6 117622241 exm574358 G C ROS1 7 9

6 117662588 exm574445 A G ROS1 1 2

6 117662757 exm574451 G A ROS1 0 2

6 117724379 exm574666 A G ROS1 23 44

6 118786639 exm574893 G A CEP85L 8 10

6 118790266 exm574895 A C CEP85L 2 5

6 118803021 exm574916 C A CEP85L 4 3

6 118832526 exm574942 A G CEP85L 1 9

6 119234579 exm575051 G A MCM9 19 20

6 119238738 exm575053 A G MCM9 2 0

6 119337914 exm575163 G A FAM184A 11 19

6 119345698 exm575207 G A FAM184A 0 2

6 119500995 exm575241 G A MAN1A1 11 30

6 119669641 exm575297 C G MAN1A1 2 4

6 121401942 exm575319 A G TBC1D32 5 11

6 121625703 exm575465 G A TBC1D32 6 10

6 123600207 exm575882 A C TRDN 6 13

6 123637602 exm575886 A G TRDN 12 22

6 125379191 exm576152 A G RNF217 0 1

6 125403624 exm576170 G A RNF217 12 41

6 125403700 exm576173 T A RNF217 7 10

6 126210797 exm576398 A G NCOA7 0 1

6 126334193 exm576530 G A TRMT11 9 20

6 126359894 exm576551 A G TRMT11 6 21

6 127608365 exm576627 G A RNF146 1 5

6 127768515 exm576750 A C KIAA0408 5 14

6 127898537 exm576883 A T C6orf58 3 5

6 128134386 exm576956 A G THEMIS 0 3

6 128135032 exm576991 A G THEMIS 7 16

6 129470160 exm577266 A G LAMA2 7 13

6 129571272 exm577314 A G LAMA2 11 25

6 129571288 exm577315 A G LAMA2 16 34

6 129571347 exm577318 G A LAMA2 1 8

6 129612776 exm577366 A G LAMA2 0 3

6 129641684 exm577434 G A LAMA2 4 15

6 129663525 exm577445 A G LAMA2 6 10

6 129687396 exm577460 A G LAMA2 11 29

6 129704328 exm577479 A G LAMA2 11 21

6 129722454 exm577505 A G LAMA2 2 12

6 129723539 exm577513 A G LAMA2 13 33

6 129748940 exm577524 G C LAMA2 20 31

6 129774209 exm577571 G A LAMA2 2 3

6 129775355 exm577577 G A LAMA2 5 6

6 129775375 exm577579 A G LAMA2 11 27

6 129785553 exm577604 C A LAMA2 11 20

6 129786384 exm577610 G A LAMA2 5 7

6 129828658 exm577695 A G LAMA2 9 27

6 129828685 exm577697 A G LAMA2 16 23

6 130497146 exm578007 A G SAMD3 0 2

6 130536388 exm578042 C A SAMD3 27 45

6 131190746 exm578148 G A EPB41L2 4 12

6 131206343 exm578191 A G EPB41L2 0 1

6 131277417 exm578259 C A EPB41L2 2 6

6 131486337 exm578294 C A AKAP7 3 10

6 131910690 exm578396 C G MED23 2 5

6 131944519 exm578485 A G MED23 2 2

6 131973691 exm578530 A G ENPP3 2 7

6 132204839 exm578792 C A ENPP1 9 19

6 132859628 exm579003 C G TAAR9 15 34

6 132860164 exm579029 C A TAAR9 16 34

6 132910567 exm579192 G C TAAR5 13 32

6 132966389 exm579280 G A TAAR1 13 29

6 132966782 exm579306 A G TAAR1 1 0

6 133032854 exm579398 A G VNN1 18 29

6 133032882 exm579399 A G VNN1 2 2

6 133078633 exm579550 A G VNN2 22 38

6 133092234 exm579573 G A SLC18B1 2 3

6 133100534 exm579597 A G SLC18B1 9 20

6 134495156 exm579959 T A SGK1 2 11

6 135239993 exm580034 A G ALDH8A1 1 4

6 135263631 exm580064 C A ALDH8A1 1 4

6 135323909 exm580160 C A HBS1L 8 16

6 135358904 exm580188 G A HBS1L 3 1

6 135363244 exm580209 A G HBS1L 16 32

6 135375791 exm580214 C A HBS1L 3 2

6 135516944 exm580253 A G MYB 15 40

6 135521489 exm580286 C A MYB 6 13

6 135521495 exm580287 A G MYB 11 22

6 135524449 exm580302 A G MYB 1 1

6 135611603 exm580324 A G AHI1 12 29

6 135679271 exm580343 A G AHI1 11 22

6 135732574 exm580370 G A AHI1 6 15

6 135732616 exm580372 G A AHI1 2 4

6 135732649 exm580374 G A AHI1 7 11

6 135751024 exm580391 A G AHI1 17 26

6 135778797 exm580437 A C AHI1 5 11

6 136677899 exm580918 A G MAP7 13 23

6 136693680 exm580985 A C MAP7 3 22

6 136710517 exm581010 A G MAP7 5 11

6 136944014 exm581103 G A MAP3K5 21 45

6 137245731 exm581244 A G SLC35D3 2 8

6 137323353 exm581283 C A IL20RA 1 3

6 137325825 exm581287 C A IL20RA 1 3

6 137330455 exm581298 A G IL20RA 2 11

6 137528119 exm581440 A G IFNGR1 1 13

6 138584014 exm581716 C G ARFGEF3 2 8

6 138584380 exm581731 G A ARFGEF3 1 1

6 139094917 exm582094 A C CCDC28A 8 19

6 139159581 exm582154 A G ECT2L 12 35

6 139167750 exm582178 G A ECT2L 4 6

6 139609718 exm582567 A G TXLNB 0 2

6 139609747 exm582568 G A TXLNB 8 11

6 142397030 exm582657 A G NMBR 4 10

6 142399822 exm582671 G A NMBR 19 28

6 142688969 exm582779 G A ADGRG6 6 15

6 142714127 exm582825 A G ADGRG6 14 29

6 142724940 exm582851 A G ADGRG6 16 34

6 143081571 exm582957 A G HIVEP2 1 1

6 143091222 exm583001 A G HIVEP2 9 15

6 143092151 exm583033 G A HIVEP2 2 5

6 143093360 exm583084 A G HIVEP2 8 14

6 143816923 exm583294 G A FUCA2 18 34

6 143823085 exm583299 A G FUCA2 22 45

6 143825075 exm583318 G A FUCA2 1 2

6 144086665 exm583412 A G PHACTR2 0 1

6 144743015 exm583659 A G UTRN 13 36

6 144747588 exm583685 C G UTRN 16 29

6 144750789 exm583697 T A UTRN 1 8

6 144780413 exm583773 A G UTRN 15 40

6 144820481 exm583861 G C UTRN 1 1

6 144843159 exm583919 A G UTRN 3 7

6 145051599 exm584040 A G UTRN 14 29

6 145103227 exm584090 A G UTRN 0 3

6 146126643 exm584294 A T FBXO30 14 31

6 146127406 exm584322 A C FBXO30 15 23

6 146262971 exm584427 A C SHPRH 12 26

6 146271534 exm584480 A G SHPRH 1 0

6 146480637 exm584534 A G GRM1 18 31

6 146625869 exm584551 A G GRM1 25 51

6 146755390 exm584628 A G GRM1 2 6

6 146755508 exm584634 A G GRM1 10 24

6 147045373 exm584736 A G ADGB 11 36

6 147047296 exm584742 G A ADGB 12 38

6 147703956 exm584930 T A STXBP5 1 13

6 149699523 exm585259 A G TAB2 3 9

6 149699925 exm585269 G A TAB2 3 12

6 149783057 exm585387 A G ZC3H12D 4 16

6 149783095 exm585390 G A ZC3H12D 5 9

6 149982881 exm585562 A T LATS1 2 10

6 150004616 exm585608 A G LATS1 0 1

6 150016243 exm585647 A G LATS1 5 6

6 150022974 exm585655 G A LATS1 11 26

6 150141821 exm585761 A G LRP11 3 7

6 150164148 exm585791 A G LRP11 6 21

6 150239484 exm585895 A G RAET1G 10 14

6 150240233 exm585902 G A RAET1G 1 1

6 151140892 exm586316 G C PLEKHG1 1 5

6 151152250 exm586358 G A PLEKHG1 7 13

6 151152805 exm586393 A G PLEKHG1 3 7

6 151161245 exm586450 A G PLEKHG1 21 33

6 151161377 exm586461 A G PLEKHG1 2 6

6 151161872 exm586485 A G PLEKHG1 0 2

6 151162004 exm586489 C A PLEKHG1 6 22

6 151239713 exm586522 A G MTHFD1L 10 22

6 151270231 exm586580 A G MTHFD1L 4 10

6 151626965 exm586675 A T AKAP12 24 54

6 151671365 exm586771 A C AKAP12 8 15

6 151672185 exm586813 A T AKAP12 0 1

6 151674196 exm586915 G C AKAP12 15 22

6 151865790 exm587150 A G CCDC170 6 12

6 151894499 exm587167 G A CCDC170 4 12

6 151914339 exm587197 A G CCDC170 0 1

6 151917660 exm587222 A G CCDC170 2 5

6 151936638 exm587226 A C CCDC170 0 2

6 151939215 exm587241 A C CCDC170 6 19

6 152453291 exm587349 A G SYNE1 10 14

6 152469200 exm587419 A G SYNE1 4 9

6 152485375 exm587475 A G SYNE1 7 15

6 152497676 exm587506 C G SYNE1 3 15

6 152527320 exm587530 G C SYNE1 12 22

6 152534783 exm587570 C A SYNE1 5 23

6 152557350 exm587661 A G SYNE1 6 11

6 152605116 exm587750 C G SYNE1 2 5

6 152631869 exm587817 A G SYNE1 14 30

6 152651368 exm587919 T A SYNE1 3 11

6 152651599 exm587929 A G SYNE1 5 12

6 152655352 exm588001 A C SYNE1 21 45

6 152665271 exm588029 A G SYNE1 11 25

6 152671811 exm588063 G A SYNE1 3 20

6 152671865 exm588065 C A SYNE1 25 40

6 152690259 exm588183 A G SYNE1 4 9

6 152690727 exm588190 G A SYNE1 6 4

6 152694302 exm588198 C G SYNE1 2 4

6 152708464 exm588251 A C SYNE1 12 25

6 152718031 exm588296 G A SYNE1 5 19

6 152718033 exm588297 C G SYNE1 14 33

6 152722356 exm588312 G A SYNE1 5 11

6 152737765 exm588348 G A SYNE1 4 22

6 152746658 exm588377 A C SYNE1 1 1

6 152772294 exm588482 A T SYNE1 3 4

6 152779933 exm588503 A G SYNE1 10 17

6 152783946 exm588515 G A SYNE1 15 33

6 152809602 exm588559 G A SYNE1 0 1

6 152847259 exm588596 A G SYNE1 5 11

6 153019197 exm588616 G A MYCT1 6 11

6 153043263 exm588641 G A MYCT1 10 20

6 154411240 exm588860 A T OPRM1 1 4

6 154412236 exm588870 A G OPRM1 13 26

6 154751632 exm589039 A C CNKSR3 9 18

6 155139718 exm589135 A G SCAF8 1 5

6 155153307 exm589188 A G SCAF8 7 8

6 155153313 exm589189 A G SCAF8 1 3

6 155450378 exm589234 G C TIAM2 7 13

6 155451168 exm589265 A G TIAM2 0 1

6 155451351 exm589273 A G TIAM2 6 4

6 155451372 exm589277 G A TIAM2 14 29

6 155503390 exm589367 A G TIAM2 15 37

6 155571006 exm589425 G A TIAM2 7 17

6 155573093 exm589448 A G TIAM2 8 10

6 155577706 exm589481 A C TIAM2 2 8

6 155577707 exm589482 A G TIAM2 4 4

6 155618218 exm589595 G A TFB1M 10 15

6 155635541 exm589615 G A TFB1M 11 15

6 155743917 exm589647 A G NOX3 3 6

6 155761166 exm589683 A G NOX3 1 2

6 155776223 exm589707 A G NOX3 5 25

6 158535845 exm590461 C A SERAC1 2 5

6 158870124 exm590573 A G TULP4 14 34

6 158910698 exm590624 A G TULP4 17 41

6 158914685 exm590634 A G TULP4 1 1

6 158923466 exm590684 G C TULP4 3 2

6 158923574 exm590691 A G TULP4 1 0

6 158994507 exm590827 A G TMEM181 16 24

6 159086455 exm590945 A G SYTL3 8 17

6 159178316 exm590998 A G SYTL3 4 12

6 159178409 exm591008 A C SYTL3 7 15

6 159185582 exm591049 A C SYTL3 7 13

6 159403577 exm591225 C A RSPH3 2 3

6 159457502 exm591320 C A TAGAP 18 32

6 159458016 exm591340 A C TAGAP 16 32

6 159459194 exm591352 A G TAGAP 16 32

6 159621087 exm591411 A G FNDC1 1 0

6 160239592 exm592000 T A PNLDC1 2 7

6 160240303 exm592023 G A PNLDC1 0 1

6 160328380 exm592062 A C MAS1 5 7

6 160468908 exm592208 A G IGF2R 0 9

6 160471599 exm592230 T A IGF2R 2 1

6 160493038 exm592336 A G IGF2R 1 3

6 160500712 exm592389 A G IGF2R 8 31

6 160501175 exm592399 A G IGF2R 11 33

6 160505075 exm592410 A T IGF2R 2 8

6 160505207 exm592416 G A IGF2R 2 2

6 160543034 exm592503 C G SLC22A1 1 3

6 160543080 exm592506 A G SLC22A1 33 56

6 160543220 exm592521 A G SLC22A1 8 19

6 160553407 exm592555 A C SLC22A1 0 4

6 160560824 exm592588 A G SLC22A1 2 4

6 161006172 exm592939 C A LPA 13 27

6 161006177 exm592940 A G LPA 1 2

6 161020641 exm593003 A C LPA 0 2

6 161022107 exm593016 A G LPA 2 2

6 161152905 exm593174 A G PLG 0 1

6 161173962 exm593211 A G PLG 1 4

6 161508880 exm593316 C A MAP3K4 19 40

6 161512457 exm593337 A G MAP3K4 3 7

6 161781225 exm593479 A G PRKN 15 30

6 162206876 exm593508 G A PRKN 4 10

6 165711487 exm593686 A G C6orf118 3 5

6 165711544 exm593691 A G C6orf118 2 6

6 165712942 exm593697 C G C6orf118 7 10

6 165715713 exm593752 A G C6orf118 5 17

6 165808688 exm593800 A G PDE10A 0 2

6 165808716 exm593803 C G PDE10A 2 2

6 166571907 exm593878 A G TBXT 1 3

6 166743706 exm593996 A C SFT2D1 6 11

6 166743735 exm593997 A G SFT2D1 0 2

6 167343167 exm594158 C G RNASET2 0 1

6 167343185 exm594160 A C RNASET2 6 22

6 167360217 exm594193 G A RNASET2 15 32

6 167453435 exm594277 A T FGFR1OP 9 19

6 167570789 exm594351 A G GPR31 3 5

6 167711466 exm594482 A G UNC93A 16 33

6 167721269 exm594546 A G UNC93A 11 32

6 167753675 exm594615 A G TTLL2 29 52

6 167754341 exm594656 G A TTLL2 9 14

6 167754454 exm594669 G A TTLL2 8 12

6 167755090 exm594714 A G TTLL2 6 8

6 168297647 exm594905 G A AFDN 13 34

6 168314939 exm594943 A G AFDN 6 9

6 168316046 exm594957 G A AFDN 12 26

6 168351978 exm595016 T A AFDN 20 32

6 168352445 exm595044 A C AFDN 4 10

6 168439301 exm595220 G A KIF25 10 29

6 168442765 exm595253 A G KIF25 15 30

6 168442775 exm595254 A C KIF25 20 39

6 168443267 exm595262 C G KIF25 6 14

6 168457824 exm595299 A G FRMD1 3 2

6 168463623 exm595363 A G FRMD1 18 36

6 169629723 exm595727 A G THBS2 18 24

6 169640686 exm595809 A G THBS2 15 20

6 169648646 exm595854 A G THBS2 0 7

6 169648732 exm595860 G A THBS2 8 14

6 169648759 exm595861 A G THBS2 1 2

6 169649047 exm595873 A G THBS2 5 17

6 169982996 exm595894 A C WDR27 11 24

6 170002413 exm595910 C G WDR27 3 2

6 170059245 exm595996 A G WDR27 2 5

6 170060821 exm596015 C G WDR27 1 0

6 170155444 exm596224 A G ERMARD 2 23

6 170155468 exm596226 A C ERMARD 10 31

6 170159119 exm596244 G A ERMARD 2 2

6 170159989 exm596251 A T ERMARD 26 34

6 170173427 exm596288 A G ERMARD 1 3

6 170176154 exm596304 G A ERMARD 14 26

6 170639554 exm596607 C G FAM120B 13 41

6 170858167 exm596671 G A PSMB1 4 9

6 116720514 JHU_6.116720513 A G DSE 9 26

6 31118898 JHU_6.31118897 T A CCHCR1 25 43

6 31379016 JHU_6.31379015 A G MICA 9 16

6 46632594 JHU_6.46632593 G A SLC25A27 8 20

6 150210539 newrs112453646 A C RAET1E 25 47

6 66005927 newrs142450703 A G EYS 18 37

6 64776324 newrs145623359 A G EYS 20 27

6 56044484 newrs181443791 A C COL21A1 4 5

7 100404052 7:100404052-CT A G EPHB4 14 26

7 100685834 7:100685834-AG G A MUC17 0 1

7 101183219 7:101183219-GA A G COL26A1 6 7

7 102584867 7:102584867-CT A G LRRC17 4 7

7 103292201 7:103292201-GA A G RELN 0 1

7 107216863 7:107216863-AG G A DUS4L;DUS4L-BCAP29 9 12

7 107423280 7:107423280-CT A G SLC26A3 14 31

7 11022288 7:11022288-AC C A PHF14 4 8

7 114563533 7:114563533-GT A C MDFIC 1 10

7 120655789 7:120655789-AG G A CPED1 13 23

7 121726133 7:121726133-GA A G AASS 0 1

7 12428940 7:12428940-TG C A VWDE 11 21

7 126544671 7:126544671-AG G A GRM8 12 29

7 12662456 7:12662456-GA A G SCIN 2 5

7 128415528 7:128415528-AG G A OPN1SW 0 3

7 128455912 7:128455912-AG G A CCDC136 1 2

7 129691075 7:129691075-CT A G ZC3HC1 13 37

7 129939149 7:129939149-GA A G CPA4 1 1

7 132070005 7:132070005-CT A G PLXNA4 0 1

7 133994148 7:133994148-AC C A SLC35B4 2 9

7 134618496 7:134618496-GA A G CALD1 5 8

7 134719318 7:134719318-CT A G AGBL3 0 1

7 134800284 7:134800284-GA A G AGBL3 0 2

7 134870940 7:134870940-TC G A WDR91 1 1

7 137092623 7:137092623-TC G A DGKI 0 4

7 138413606 7:138413606-TC G A ATP6V0A4 3 3

7 138434044 7:138434044-TC G A ATP6V0A4 3 7

7 138738274 7:138738274-CT A G ZC3HAV1 1 0

7 140386917 7:140386917-TC G A ADCK2 15 27

7 142562110 7:142562110-CA A C EPHB6 2 6

7 142625953 7:142625953-CT A G TRPV5 7 16

7 142829295 7:142829295-AG G A PIP 9 22

7 144094583 7:144094583-GA A G NOBOX 16 29

7 149153005 7:149153005-CT A G ZNF777 2 12

7 149547287 7:149547287-TG C A ZNF862 6 8

7 150174813 7:150174813-AC C A GIMAP8 10 23

7 150554171 7:150554171-CT A G AOC1 0 2

7 150557578 7:150557578-GA A G AOC1 25 38

7 150713610 7:150713610-AG G A ATG9B 18 25

7 150934869 7:150934869-GA A G CHPF2 1 6

7 151874692 7:151874692-GA A G KMT2C 4 11

7 154379570 7:154379570-CT A G DPP6 0 2

7 157985077 7:157985077-GA A G PTPRN2 2 5

7 158727128 7:158727128-CT A G WDR60 1 0

7 20683197 7:20683197-CT A G ABCB5 3 13

7 20683227 7:20683227-TC G A ABCB5 1 5

7 20691082 7:20691082-CT A G ABCB5 2 5

7 20766691 7:20766691-GA A G ABCB5 0 2

7 21599255 7:21599255-AG G A DNAH11 0 1

7 21765487 7:21765487-AG G A DNAH11 2 9

7 21781723 7:21781723-TC G A DNAH11 2 4

7 21827050 7:21827050-AG G A DNAH11 3 6

7 2252860 7:2252860-CT A G MAD1L1 0 2

7 2262292 7:2262292-CT A G MAD1L1 1 8

7 26217669 7:26217669-AG G A NFE2L3 10 17

7 2646876 7:2646876-TG C A IQCE 7 14

7 27809349 7:27809349-GT A C TAX1BP1 2 7

7 2802249 7:2802249-GA A G GNA12 2 1

7 2956986 7:2956986-TC G A CARD11 4 9

7 29915534 7:29915534-GA A G WIPF3 0 4

7 30469032 7:30469032-AC C A NOD1 2 5

7 32249110 7:32249110-GA A G PDE1C 2 12

7 33015986 7:33015986-AG G A FKBP9 3 5

7 38500927 7:38500927-CT A G AMPH 1 2

7 38813779 7:38813779-CA A C VPS41 4 8

7 40789051 7:40789051-GA A G SUGCT 0 2

7 4185438 7:4185438-CT A G SDK1 3 9

7 44120340 7:44120340-GA A G POLM 10 24

7 47467947 7:47467947-TC G A TNS3 0 1

7 48556409 7:48556409-AG G A ABCA13 1 3

7 5232786 7:5232786-CT A G WIPI2 6 13

7 5529775 7:5529775-GA A G FBXL18 1 1

7 56022686 7:56022686-CT A G MRPS17 12 16

7 56022852 7:56022852-AG G A MRPS17 2 7

7 5642987 7:5642987-CT A G FSCN1 10 36

7 5662649 7:5662649-CT A G RNF216 12 15

7 63538334 7:63538334-AG G A ZNF727 8 14

7 64452158 7:64452158-CT A G ERV3-1 10 20

7 64452526 7:64452526-AT A T ERV3-1 3 5

7 76069856 7:76069856-CT A G ZP3 0 1

7 80293741 7:80293741-GA A G CD36 1 3

7 80303398 7:80303398-G-A A G CD36 3 7

7 825232 7:825232-CT A G DNAAF5 2 6

7 872174 7:872174-CT A G SUN1 1 3

7 87780302 7:87780302-CA A C ADAM22 1 7

7 912195 7:912195-CT A G SUN1 8 18

7 91671427 7:91671427-CT A G AKAP9 1 1

7 92099283 7:92099283-CT A G ERVW-1 31 72

7 92099310 7:92099310-AG G A ERVW-1 3 2

7 92122430 7:92122430-TG C A PEX1 1 1

7 97617739 7:97617739-TG C A OCM2 3 6

7 99129790 7:99129790-GA A G ZKSCAN5 1 1

7 99144391 7:99144391-GA A G FAM200A 1 1

7 99145519 7:99145519-TC G A FAM200A 0 1

7 99170909 7:99170909-CT A G ZNF655 7 17

7 99247731 7:99247731-GA A G CYP3A5 2 4

7 99662218 7:99662218-CA A C ZSCAN21 3 6

7 99695495 7:99695495-GT A C MCM7 5 8

7 99758145 7:99758145-CT A G GAL3ST4 10 17

7 100685612 exm2134514 C A MUC17 8 18

7 100855929 exm2134727 G C PLOD3 5 13

7 103777294 exm2135557 C A ORC5 1 2

7 105641910 exm2135830 A G CDHR3 2 9

7 122130281 exm2137796 G A CADPS2 9 13

7 123092970 exm2137911 A G IQUB 7 16

7 123594498 exm2138071 A G SPAM1 2 3

7 12675735 exm2138181 A G SCIN 8 19

7 138758745 exm2140443 A G ZC3HAV1 8 22

7 1478557 exm2142420 A G MICALL2 5 16

7 151791576 exm2144226 C A GALNT11 18 26

7 25175950 exm2146931 A G C7orf31 6 12

7 37890034 exm2148910 G A NME8 1 0

7 4051752 exm2149352 G A SDK1 2 2

7 47915787 exm2150765 C A PKD1L1 0 3

7 48550771 exm2151223 A G ABCA13 1 2

7 75186963 exm2154568 A G HIP1 21 34

7 75583335 exm2154654 A G POR 0 1

7 77011932 exm2155151 A C GSAP 10 14

7 99526646 exm2158705 A C GJC3 0 3

7 48313381 exm2274236 A G ABCA13 0 2

7 195679 exm596831 A G FAM20C 8 21

7 195681 exm596832 A G FAM20C 11 22

7 803506 exm597111 A G DNAAF5 1 12

7 803515 exm597112 G A DNAAF5 5 5

7 878536 exm597201 A G SUN1 1 5

7 893125 exm597304 G A SUN1 13 26

7 900000 exm597349 A G SUN1 25 32

7 901098 exm597356 A G SUN1 3 5

7 1478487 exm598119 T A MICALL2 18 30

7 2284237 exm599323 G C NUDT1 0 1

7 2290617 exm599357 A G NUDT1 0 2

7 2302956 exm599402 C G SNX8 0 3

7 2304078 exm599420 A G SNX8 3 5

7 2581814 exm599969 A G BRAT1 16 27

7 2617988 exm600112 A G IQCE 0 1

7 2748717 exm600448 A G AMZ1 9 19

7 2771003 exm600573 A G GNA12 3 7

7 2946283 exm600656 A G CARD11 9 30

7 2951891 exm600682 A G CARD11 7 19

7 2951925 exm600683 A G CARD11 9 9

7 2959240 exm600732 A G CARD11 1 1

7 2969669 exm600788 C G CARD11 17 32

7 2969698 exm600793 A C CARD11 17 32

7 2987413 exm600854 A C CARD11 7 20

7 3861149 exm600913 A G SDK1 3 5

7 4008969 exm600998 A G SDK1 3 5

7 4116656 exm601105 A G SDK1 14 18

7 4167045 exm601177 A G SDK1 2 2

7 4201448 exm601271 A G SDK1 5 9

7 4259894 exm601392 A C SDK1 11 20

7 4260921 exm601399 A C SDK1 1 6

7 4285381 exm601464 A G SDK1 5 16

7 4827859 exm601782 A G AP5Z1 0 1

7 4830897 exm601877 G A AP5Z1 14 23

7 4841614 exm601967 A G RADIL 16 27

7 4855862 exm602009 G A RADIL 6 21

7 4899575 exm602101 A G PAPOLB 13 26

7 4899640 exm602105 A G PAPOLB 1 8

7 4899667 exm602108 G A PAPOLB 3 7

7 5269291 exm602511 A G WIPI2 12 26

7 5401346 exm602934 A G TNRC18 12 25

7 5662544 exm603441 C G RNF216 3 10

7 5760692 exm603507 T A RNF216 3 6

7 5781280 exm603564 A G RNF216 9 20

7 5792601 exm603582 G A RNF216 3 12

7 6026708 exm603845 A C PMS2 20 27

7 6035238 exm603904 A C PMS2 2 3

7 6042238 exm603927 A G PMS2 3 3

7 6066396 exm604048 G A EIF2AK1 6 12

7 6080686 exm604111 T A EIF2AK1 32 52

7 6196414 exm604365 A G USP42 17 37

7 6621269 exm604851 C A ZDHHC4 17 20

7 6621814 exm604857 C A ZDHHC4 0 1

7 7483242 exm605381 A G COL28A1 7 14

7 7495692 exm605405 A C COL28A1 8 14

7 7550759 exm605436 A C COL28A1 17 38

7 7561580 exm605455 G A COL28A1 5 16

7 11022095 exm605814 G A PHF14 12 30

7 11500372 exm606007 A G THSD7A 29 46

7 11501732 exm606015 G A THSD7A 3 10

7 11582666 exm606061 A G THSD7A 1 3

7 11582733 exm606065 G A THSD7A 3 7

7 11630141 exm606067 A G THSD7A 2 6

7 11871406 exm606145 C G THSD7A 17 23

7 11871419 exm606146 A G THSD7A 2 22

7 12412829 exm606321 A G VWDE 13 16

7 12644253 exm606399 A G SCIN 0 1

7 12680117 exm606432 G A SCIN 0 1

7 12684308 exm606452 A C SCIN 0 1

7 15430312 exm606680 A T AGMO 2 6

7 15433761 exm606694 A G AGMO 6 7

7 15458194 exm606699 A T AGMO 15 26

7 15601379 exm606727 G A AGMO 5 17

7 16341073 exm606805 A G CRPPA 3 2

7 16640445 exm606886 A G ANKMY2 1 7

7 16655389 exm606925 G A ANKMY2 3 3

7 17373654 exm607146 A G AHR 13 18

7 17378904 exm607170 C A AHR 10 6

7 17378917 exm607171 G A AHR 21 41

7 17379437 exm607199 G A AHR 6 12

7 18066679 exm607321 G A PRPS1L1 5 7

7 18067174 exm607338 C A PRPS1L1 22 36

7 18067261 exm607342 G C PRPS1L1 5 24

7 18631217 exm607367 C G HDAC9 12 16

7 18674363 exm607373 A G HDAC9 4 4

7 18705957 exm607394 C G HDAC9 1 1

7 20193979 exm607622 C A MACC1 7 15

7 20197918 exm607629 C A MACC1 8 25

7 20198845 exm607669 G A MACC1 0 1

7 20198917 exm607675 A G MACC1 1 1

7 20199023 exm607680 C A MACC1 17 29

7 20199532 exm607711 A G MACC1 0 1

7 20199589 exm607712 A C MACC1 7 14

7 20201458 exm607734 A G MACC1 1 1

7 20418794 exm607759 G C ITGB8 4 10

7 20434528 exm607787 A C ITGB8 1 1

7 20682892 exm607851 A G ABCB5 1 14

7 20682949 exm607856 A G ABCB5 24 42

7 20685479 exm607873 A G ABCB5 8 20

7 20689724 exm607899 A G ABCB5 31 60

7 20698164 exm607927 C A ABCB5 1 3

7 20721141 exm607947 A G ABCB5 3 2

7 20721165 exm607951 A G ABCB5 0 3

7 20738141 exm607966 A G ABCB5 1 2

7 20739715 exm607979 A G ABCB5 3 4

7 20744360 exm607981 C A ABCB5 7 20

7 20768033 exm608015 A C ABCB5 0 4

7 20768077 exm608017 A T ABCB5 21 37

7 20778733 exm608024 A G ABCB5 6 7

7 20782542 exm608035 G A ABCB5 1 3

7 20785017 exm608060 A G ABCB5 4 7

7 20795099 exm608072 G A ABCB5 6 11

7 21584742 exm608239 A G DNAH11 6 8

7 21598585 exm608252 A T DNAH11 7 13

7 21678607 exm608466 G A DNAH11 1 9

7 21775369 exm608601 A G DNAH11 1 3

7 21778438 exm608612 A G DNAH11 14 29

7 21788220 exm608662 G C DNAH11 12 14

7 21913099 exm608867 A G DNAH11 11 24

7 21920494 exm608873 C A DNAH11 4 12

7 21934511 exm608896 A G DNAH11 7 19

7 21951353 exm609014 G C CDCA7L 1 1

7 21951363 exm609015 A G CDCA7L 8 15

7 22985359 exm609223 A G FAM126A 4 8

7 22985710 exm609238 A G FAM126A 10 23

7 23017959 exm609277 A C FAM126A 2 8

7 23240102 exm609410 A G NUP42 6 23

7 23793953 exm609829 A G STK31 27 54

7 23808726 exm609850 A G STK31 3 12

7 23827618 exm609897 G A STK31 4 9

7 24742444 exm610028 A G GSDME 3 7

7 24749882 exm610068 G A GSDME 10 20

7 24756912 exm610087 A G GSDME 3 13

7 24874298 exm610196 A G OSBPL3 5 10

7 24902911 exm610225 A G OSBPL3 3 4

7 25175788 exm610290 A C C7orf31 3 6

7 25176355 exm610315 A G C7orf31 1 4

7 25182373 exm610334 A G C7orf31 4 9

7 26224637 exm610486 C G NFE2L3 1 1

7 26225179 exm610537 G A NFE2L3 3 15

7 27203346 exm611060 G C HOXA9 5 8

7 27204921 exm611095 A C HOXA9 16 29

7 27824897 exm611328 G A TAX1BP1 3 7

7 29924072 exm611850 G A WIPF3 7 12

7 29994945 exm611933 C G SCRN1 22 25

7 30088851 exm611995 A T PLEKHA8 0 2

7 30088981 exm612000 G A PLEKHA8 9 15

7 30094331 exm612012 A G PLEKHA8 16 22

7 30100518 exm612020 G A PLEKHA8 3 6

7 30475632 exm612103 A G NOD1 1 1

7 30486592 exm612115 A G NOD1 31 46

7 30491609 exm612170 C A NOD1 0 2

7 30492366 exm612207 A G NOD1 8 17

7 30695556 exm612410 G A CRHR2 2 5

7 30706883 exm612456 A C CRHR2 6 7

7 30795106 exm612504 G A INMT 0 3

7 30795315 exm612528 A G INMT 1 2

7 30818064 exm612555 A G MINDY4 2 5

7 30825571 exm612580 A G MINDY4 14 37

7 30830967 exm612594 A T MINDY4 6 9

7 30831163 exm612604 A G MINDY4 8 18

7 31011625 exm612785 C G GHRHR 6 19

7 31018852 exm612848 G A GHRHR 27 29

7 31123789 exm612883 G A ADCYAP1R1 7 10

7 31126580 exm612895 C G ADCYAP1R1 16 31

7 31609381 exm612967 G A ITPRID1 16 25

7 31617480 exm612984 A G ITPRID1 11 20

7 31617627 exm612989 A G ITPRID1 4 6

7 31617702 exm612992 G A ITPRID1 14 26

7 31682908 exm613042 G A ITPRID1 2 5

7 31682927 exm613043 A T ITPRID1 6 17

7 31683331 exm613052 A G ITPRID1 11 31

7 31692244 exm613080 G A ITPRID1 15 33

7 31793149 exm613127 A G PDE1C 0 3

7 31855672 exm613151 A C PDE1C 3 6

7 33397562 exm613678 G A BBS9 2 5

7 33545253 exm613713 G A BBS9 11 21

7 33573603 exm613721 T A BBS9 10 22

7 34086005 exm613796 A G BMPER 7 12

7 34125410 exm613832 G A BMPER 4 4

7 34867125 exm613921 C G NPSR1 3 6

7 34867169 exm613925 A G NPSR1 6 12

7 36396795 exm614312 A G KIAA0895 18 32

7 36396861 exm614314 A G KIAA0895 17 27

7 36445900 exm614366 A G ANLN 3 6

7 36450740 exm614402 A G ANLN 0 2

7 36561680 exm614515 A G AOAH 4 10

7 36579939 exm614529 A G AOAH 9 20

7 36579986 exm614533 A C AOAH 3 4

7 36588221 exm614538 A G AOAH 0 2

7 37901702 exm614794 A G NME8 7 8

7 37907421 exm614819 A G NME8 17 35

7 37923923 exm614829 G A NME8 7 20

7 37934073 exm614850 C A NME8 17 31

7 37936527 exm614860 A G NME8 5 9

7 38431385 exm615307 C G AMPH 10 21

7 38433795 exm615334 C G AMPH 13 33

7 42004017 exm616048 C A GLI3 7 14

7 42004062 exm616052 A G GLI3 4 9

7 42007446 exm616185 A G GLI3 6 15

7 42012033 exm616193 A G GLI3 7 21

7 43647893 exm616643 G A STK17A 10 25

7 43916738 exm616822 A G URGCP 3 10

7 44098033 exm617071 A G DBNL 10 20

7 44118394 exm617217 G C POLM 3 7

7 44146151 exm617254 A G AEBP1 7 17

7 44153825 exm617424 A G AEBP1 2 6

7 44574135 exm617961 A G NPC1L1 4 11

7 44578812 exm618021 A G NPC1L1 3 9

7 44578918 exm618028 A G NPC1L1 14 32

7 44579062 exm618037 G C NPC1L1 1 4

7 44579601 exm618080 G A NPC1L1 8 14

7 44579797 exm618094 T A NPC1L1 3 5

7 44579814 exm618096 A G NPC1L1 16 27

7 44605663 exm618117 C A DDX56 9 14

7 44608766 exm618137 A G DDX56 0 3

7 44611208 exm618172 A G DDX56 5 7

7 44612026 exm618181 A G DDX56 1 3

7 45016587 exm618735 A C MYO1G 0 2

7 45018542 exm618744 A G MYO1G 15 36

7 45067314 exm618756 C G CCM2 2 10

7 45141667 exm618996 A G TBRG4 11 31

7 45222793 exm619105 A G RAMP3 9 22

7 45222968 exm619115 A C RAMP3 0 2

7 47319774 exm619349 A G TNS3 16 28

7 47408426 exm619488 G A TNS3 10 11

7 47409107 exm619525 A G TNS3 2 5

7 47474935 exm619581 A G TNS3 20 49

7 47842894 exm619671 G A PKD1L1 2 2

7 47860690 exm619740 A G PKD1L1 11 21

7 47874805 exm619790 G A PKD1L1 0 2

7 47884668 exm619851 A G PKD1L1 1 1

7 47892736 exm619864 A G PKD1L1 1 0

7 47897379 exm619890 C G PKD1L1 2 7

7 47898311 exm619897 A G PKD1L1 3 2

7 47913560 exm619924 A C PKD1L1 18 39

7 47925424 exm619978 A G PKD1L1 3 8

7 47925520 exm619985 A G PKD1L1 1 5

7 47925619 exm620000 A G PKD1L1 10 17

7 47942032 exm620059 A G PKD1L1 0 2

7 47942035 exm620060 G A PKD1L1 13 18

7 47947752 exm620099 A G PKD1L1 3 5

7 47947836 exm620104 G A PKD1L1 12 34

7 47955065 exm620108 C A PKD1L1 11 25

7 47968830 exm620115 A G PKD1L1 5 11

7 47970807 exm620138 A G PKD1L1 18 39

7 47971562 exm620148 A G PKD1L1 13 24

7 48045622 exm620259 T A SUN3 15 25

7 48046775 exm620264 A G SUN3 7 16

7 48056891 exm620280 A G SUN3 13 23

7 48065476 exm620285 G A SUN3 13 23

7 48081051 exm620303 A G C7orf57 1 4

7 48086082 exm620312 A T C7orf57 5 14

7 48266869 exm620440 G A ABCA13 3 15

7 48266877 exm620442 A G ABCA13 3 8

7 48280558 exm620467 C A ABCA13 1 2

7 48311658 exm620513 A G ABCA13 6 12

7 48311884 exm620519 A G ABCA13 15 26

7 48311941 exm620526 A G ABCA13 10 31

7 48312084 exm620530 G C ABCA13 11 18

7 48312637 exm620553 A C ABCA13 2 4

7 48315202 exm620649 G A ABCA13 13 16

7 48315552 exm620660 G A ABCA13 8 19

7 48318527 exm620718 A T ABCA13 2 6

7 48318613 exm620722 G A ABCA13 1 1

7 48416127 exm620870 A G ABCA13 1 2

7 48428682 exm620880 A G ABCA13 2 2

7 48431543 exm620889 A G ABCA13 9 16

7 48450123 exm620910 A G ABCA13 3 2

7 48547481 exm620980 A G ABCA13 2 5

7 48556328 exm620994 G A ABCA13 1 1

7 50513062 exm621246 G A FIGNL1 2 5

7 50514340 exm621303 A G FIGNL1 7 21

7 50514904 exm621332 A G FIGNL1 3 1

7 51094269 exm621553 A G COBL 17 30

7 51095696 exm621576 G A COBL 24 35

7 51096355 exm621620 A G COBL 0 2

7 51096394 exm621624 A G COBL 28 37

7 51096910 exm621665 A G COBL 0 2

7 51097193 exm621683 A G COBL 5 15

7 51097255 exm621688 A G COBL 1 3

7 51287525 exm621767 A G COBL 20 33

7 55238142 exm622017 G C EGFR 10 21

7 55240692 exm622036 C A EGFR 4 9

7 55874811 exm622267 A G SEPTIN14 2 3

7 55902230 exm622281 G A SEPTIN14 1 5

7 55910775 exm622288 A C SEPTIN14 11 26

7 64166802 exm623109 A C ZNF107 3 3

7 64167502 exm623136 C A ZNF107 11 21

7 64363742 exm623327 A C ZNF273 6 14

7 64388629 exm623361 G A ZNF273 12 22

7 64439216 exm623438 A G ERV3-1-ZNF117;ZNF117 19 35

7 64439785 exm623460 C G ERV3-1-ZNF117;ZNF117 5 7

7 64451709 exm623475 A G ERV3-1 7 13

7 64452458 exm623518 C A ERV3-1 27 49

7 64453085 exm623557 A G ERV3-1 15 23

7 65706155 exm623946 A G TPST1 2 6

7 65751580 exm623954 A G TPST1 0 3

7 72744321 exm625228 C G FKBP6 5 17

7 72756809 exm625252 A G FKBP6 4 18

7 72912892 exm625432 G A BAZ1B 3 7

7 72992858 exm625562 G C TBL2 29 50

7 73731968 exm626621 A G CLIP2 1 4

7 73753191 exm626644 A G CLIP2 6 14

7 73790458 exm626699 A G CLIP2 4 11

7 73803540 exm626753 A G CLIP2 3 7

7 73811558 exm626771 A G CLIP2 11 16

7 75172253 exm627612 G A HIP1 22 35

7 75197531 exm627734 A G HIP1 2 10

7 75513055 exm627848 A G RHBDD2 1 2

7 75608848 exm627887 G A POR 0 1

7 75694252 exm628202 G C MDH2 1 0

7 75695613 exm628209 G A MDH2 13 11

7 76021292 exm628362 A G SSC4D 4 14

7 76024576 exm628378 A G SSC4D 1 4

7 76871048 exm628852 A G CCDC146 5 14

7 76909913 exm628923 G A CCDC146 7 15

7 76922297 exm628953 C A CCDC146 15 29

7 76942810 exm629004 A C GSAP 12 32

7 76958692 exm629031 C G GSAP 0 4

7 77011944 exm629106 G A GSAP 4 9

7 77378843 exm629249 A G RSBN1L 11 25

7 77408295 exm629294 G A RSBN1L 0 1

7 77549759 exm629349 G A PHTF2 7 15

7 77551959 exm629350 G C PHTF2 2 7

7 80088014 exm629630 A C GNAT3 18 39

7 80091826 exm629639 A G GNAT3 1 0

7 80292448 exm629722 A G CD36 2 3

7 82474616 exm630227 A G PCLO 2 8

7 82508736 exm630246 A G PCLO 11 31

7 82579137 exm630342 C G PCLO 12 21

7 82580531 exm630402 A G PCLO 7 10

7 82582200 exm630453 A G PCLO 5 12

7 82582698 exm630472 A T PCLO 4 6

7 82584637 exm630563 G A PCLO 8 23

7 82585002 exm630579 A G PCLO 6 18

7 82595242 exm630636 A C PCLO 10 25

7 82595361 exm630640 G A PCLO 10 15

7 82595736 exm630664 A G PCLO 14 31

7 82784660 exm630743 A G PCLO 27 57

7 82784995 exm630761 G A PCLO 8 15

7 82785182 exm630770 G C PCLO 8 15

7 83591080 exm630915 G C SEMA3A 16 34

7 83634712 exm630950 A G SEMA3A 5 5

7 83634817 exm630956 G A SEMA3A 11 21

7 83636783 exm630962 A G SEMA3A 5 20

7 83823846 exm631007 C A SEMA3A 0 5

7 83823894 exm631009 G C SEMA3A 11 25

7 84651806 exm631090 G A SEMA3D 0 4

7 84697540 exm631116 A G SEMA3D 0 5

7 86542411 exm631276 A G KIAA1324L 19 32

7 86554972 exm631300 C A KIAA1324L 2 3

7 86800364 exm631358 C G DMTF1 0 2

7 86823333 exm631421 G A DMTF1 4 11

7 86988538 exm631475 C A CROT 12 22

7 87079357 exm631692 A G ABCB4 2 7

7 87082273 exm631698 G A ABCB4 0 1

7 87101971 exm631711 A G ABCB4 25 52

7 87160789 exm631781 G A ABCB1 12 10

7 87229440 exm631879 G A ABCB1 2 0

7 87466120 exm631943 G A SLC25A40 12 13

7 87479242 exm631971 A G SLC25A40 1 3

7 87792353 exm632159 A T ADAM22 15 35

7 87822534 exm632187 A G ADAM22 16 30

7 87837866 exm632198 A G SRI 11 18

7 87839361 exm632207 G A SRI 10 23

7 88963041 exm632362 T A ZNF804B 10 15

7 88964034 exm632408 A G ZNF804B 4 11

7 88964350 exm632426 A C ZNF804B 10 19

7 88964776 exm632443 G A ZNF804B 6 30

7 88965021 exm632462 A G ZNF804B 7 20

7 88965189 exm632479 G A ZNF804B 17 30

7 89938628 exm632826 A G CFAP69 1 2

7 89938653 exm632828 G A CFAP69 15 22

7 89939448 exm632839 A G CFAP69 2 2

7 89982260 exm632868 G A GTPBP10 9 24

7 90001496 exm632884 C G GTPBP10 18 45

7 91503356 exm633130 A G MTERF1 18 39

7 91503384 exm633131 G C MTERF1 18 39

7 91622249 exm633182 A C AKAP9 1 2

7 91622257 exm633183 A C AKAP9 0 2

7 91625067 exm633197 G A AKAP9 3 14

7 91630457 exm633212 C A AKAP9 1 0

7 91630603 exm633220 C G AKAP9 6 11

7 91630882 exm633232 G A AKAP9 4 7

7 91646406 exm633303 A G AKAP9 6 26

7 91651648 exm633307 C A AKAP9 0 1

7 91659223 exm633317 A G AKAP9 3 0

7 91682099 exm633366 G A AKAP9 11 22

7 91694701 exm633399 G A AKAP9 21 39

7 91709085 exm633442 G A AKAP9 15 25

7 91713984 exm633476 G C AKAP9 0 3

7 91715644 exm633489 A G AKAP9 5 10

7 91726960 exm633549 A G AKAP9 12 20

7 91732035 exm633569 G C AKAP9 17 26

7 91732110 exm633579 A G AKAP9 8 20

7 91830054 exm633700 A G KRIT1 5 10

7 91830719 exm633707 C A KRIT1 17 22

7 91863836 exm633754 A G KRIT1 0 1

7 91870334 exm633777 G A KRIT1 1 4

7 92120653 exm634033 G A PEX1 6 18

7 92131349 exm634084 C G PEX1 21 43

7 92132493 exm634090 G A PEX1 31 72

7 92134072 exm634099 A G PEX1 2 1

7 92134142 exm634103 A C PEX1 3 3

7 92146895 exm634148 G A PEX1 24 40

7 92147192 exm634162 A G PEX1 10 27

7 92730850 exm634316 A G SAMD9 12 41

7 92761026 exm634519 A G SAMD9L 1 3

7 92762214 exm634548 A G SAMD9L 1 1

7 92763720 exm634592 A G SAMD9L 6 9

7 92763913 exm634600 A C SAMD9L 2 16

7 92848687 exm634696 A G HEPACAM2 3 4

7 93516157 exm634912 A G TFPI2 6 9

7 93518412 exm634926 A G TFPI2 11 29

7 93518529 exm634930 A G TFPI2 13 26

7 94038721 exm635047 A C COL1A2 1 2

7 94057691 exm635225 A G COL1A2 5 7

7 94057692 exm635226 A G COL1A2 17 30

7 94540527 exm635468 A G PPP1R9A 0 3

7 94917978 exm635570 A C PPP1R9A 1 2

7 94991765 exm635672 G A PON3 3 13

7 94992109 exm635676 G A PON3 3 3

7 95709792 exm635965 A G DYNC1I1 2 5

7 97617828 exm636273 A G OCM2 11 23

7 97821682 exm636377 G A LMTK2 19 33

7 97822305 exm636409 A G LMTK2 8 16

7 97822322 exm636411 A C LMTK2 1 1

7 97847327 exm636577 A G TECPR1 11 15

7 97863119 exm636691 G A TECPR1 2 7

7 97874361 exm636740 A G TECPR1 0 1

7 98506462 exm637111 C G TRRAP 1 2

7 98554070 exm637293 A G TRRAP 17 32

7 98573791 exm637367 C G TRRAP 10 20

7 98608778 exm637483 A G TRRAP 2 5

7 99030926 exm637980 G A ATP5MF-PTCD1;PTCD1 5 11

7 99032471 exm637993 A G ATP5MF-PTCD1;PTCD1 4 8

7 99084555 exm638160 A G ZNF789 8 25

7 99084746 exm638171 A G ZNF789 6 16

7 99084756 exm638173 A G ZNF789 2 5

7 99091864 exm638219 A G ZNF394 9 23

7 99097533 exm638258 G A ZNF394 11 26

7 99123669 exm638300 A G ZKSCAN5 9 13

7 99128746 exm638317 A G ZKSCAN5 0 1

7 99158216 exm638410 T A ZNF655 5 6

7 99159522 exm638415 G C ZNF655 0 1

7 99264299 exm638635 G A CYP3A5 0 2

7 99312240 exm638701 G A CYP3A7;CYP3A7-CYP3A51P 0 4

7 99359800 exm638762 A G CYP3A4 7 17

7 99359829 exm638766 A G CYP3A4 1 0

7 99500926 exm639007 C G TRIM4 8 24

7 99527231 exm639090 C A GJC3 10 17

7 99569627 exm639138 A G AZGP1 1 3

7 99621928 exm639168 A G ZKSCAN1 1 0

7 99627508 exm639172 A G ZKSCAN1 3 4

7 99661810 exm639260 A G ZSCAN21 1 0

7 99668842 exm639295 G A ZNF3 5 13

7 99669712 exm639326 A G ZNF3 0 2

7 99669832 exm639338 A G ZNF3 15 16

7 99695787 exm639504 G A MCM7 9 17

7 99702947 exm639622 A G AP4M1 22 52

7 99704079 exm639648 A C AP4M1 0 3

7 99757982 exm640012 G A GAL3ST4 1 0

7 99764358 exm640056 A G GAL3ST4 3 14

7 99957008 exm640506 A G PILRB 5 6

7 99997218 exm640563 A G PILRA 9 17

7 99997454 exm640569 A G PILRA 5 5

7 100014711 exm640640 G A ZCWPW1 14 30

7 100146467 exm640984 A G AGFG2 4 14

7 100159986 exm641016 C G AGFG2 4 12

7 100189443 exm641216 G A FBXO24 1 3

7 100203360 exm641318 A G PCOLCE 5 11

7 100204134 exm641323 A C PCOLCE 2 2

7 100218497 exm641396 G A TFR2 6 13

7 100225205 exm641438 T A TFR2 12 17

7 100225860 exm641456 A G TFR2 0 3

7 100226905 exm641464 G A TFR2 4 11

7 100369544 exm642226 A T ZAN 1 4

7 100382437 exm642314 A C ZAN 3 15

7 100401183 exm642439 A G EPHB4 1 6

7 100417218 exm642536 A G EPHB4 16 15

7 100454700 exm642660 A C SLC12A9 1 2

7 100465747 exm642802 A C TRIP6 1 3

7 100465824 exm642810 A G TRIP6 1 1

7 100470824 exm642871 A G TRIP6 14 27

7 100633938 exm643587 A G MUC12 10 13

7 100675572 exm643956 A G MUC17 16 26

7 100675713 exm643960 A G MUC17 4 9

7 100679778 exm644385 A G MUC17 16 27

7 100685067 exm644958 A G MUC17 1 6

7 100685103 exm644962 A G MUC17 2 3

7 100685673 exm644992 A C MUC17 0 2

7 100685994 exm645012 A G MUC17 3 4

7 100692151 exm645087 C A MUC17 12 27

7 100693848 exm645104 A G MUC17 11 21

7 100855631 exm645661 A G PLOD3 1 1

7 100856425 exm645685 A G PLOD3 7 7

7 101183198 exm645918 A G COL26A1 9 6

7 101844882 exm646140 A G CUX1 5 19

7 101844993 exm646148 G C CUX1 7 8

7 101845066 exm646150 A G CUX1 0 1

7 101845140 exm646156 A G CUX1 0 2

7 101882626 exm646209 A G CUX1 8 10

7 101917530 exm646247 A G CUX1 2 5

7 101926028 exm646289 A G CUX1 1 4

7 102453914 exm646836 A G FBXL13 8 19

7 102518808 exm646856 A C FBXL13 14 16

7 102574715 exm646910 G A LRRC17 2 5

7 102575018 exm646924 A T LRRC17 7 14

7 102604037 exm646954 A G FBXL13 1 5

7 102604045 exm646955 A G FBXL13 1 1

7 102760507 exm647100 A G NAPEPLD 4 11

7 103018088 exm647341 C A SLC26A5 3 7

7 103018219 exm647348 G A SLC26A5 14 25

7 103194115 exm647603 A C RELN 3 15

7 103194153 exm647607 A G RELN 8 8

7 103205735 exm647645 C G RELN 1 3

7 103214713 exm647694 G A RELN 2 2

7 103236985 exm647725 A G RELN 0 1

7 103292112 exm647774 C A RELN 3 3

7 103805693 exm647878 T A ORC5 3 2

7 104730657 exm648045 A G KMT2E 0 1

7 105429058 exm648505 A G ATXN7L1 1 1

7 105621462 exm648552 G A CDHR3 1 3

7 105658381 exm648628 A G CDHR3 10 34

7 105662869 exm648652 A C CDHR3 14 30

7 105664944 exm648666 A G CDHR3 17 26

7 105672929 exm648683 A G CDHR3 5 7

7 105673118 exm648695 C A CDHR3 5 5

7 106509435 exm648878 A G PIK3CG 2 5

7 106515226 exm648915 A C PIK3CG 1 7

7 106515241 exm648916 A G PIK3CG 1 4

7 106851618 exm649053 G C COG5 2 3

7 106888938 exm649073 A G COG5 10 35

7 106938653 exm649103 G A COG5 1 4

7 106938716 exm649108 A G COG5 1 4

7 106938740 exm649110 A G COG5 6 17

7 107013085 exm649131 T A COG5 10 30

7 107194775 exm649203 A G COG5 0 4

7 107258904 exm649319 A G BCAP29;DUS4L-BCAP29 17 28

7 107258931 exm649323 A T BCAP29;DUS4L-BCAP29 0 6

7 107314728 exm649373 A G SLC26A4 4 12

7 107323753 exm649390 A G SLC26A4 3 5

7 107323945 exm649393 G A SLC26A4 3 10

7 107329565 exm649405 A G SLC26A4 4 13

7 107344800 exm649456 A C SLC26A4 3 9

7 107350548 exm649466 C A SLC26A4 14 33

7 107350599 exm649470 A C SLC26A4 8 30

7 107408324 exm649558 G C SLC26A3 20 43

7 107431533 exm649649 A G SLC26A3 2 5

7 107569962 exm649795 G A LAMB1 0 1

7 107599806 exm649922 A G LAMB1 20 25

7 107599827 exm649924 A G LAMB1 7 12

7 107599842 exm649926 C G LAMB1 9 35

7 107601091 exm649948 G C LAMB1 8 23

7 107603471 exm649981 A G LAMB1 5 14

7 107605124 exm649997 A G LAMB1 9 9

7 107615741 exm650012 G A LAMB1 0 2

7 107671300 exm650085 A G LAMB4 1 4

7 107720211 exm650260 G C LAMB4 7 11

7 107735722 exm650287 A T LAMB4 3 10

7 107746975 exm650342 A C LAMB4 5 10

7 107749668 exm650358 C G LAMB4 35 44

7 107830092 exm650462 G A NRCAM 2 3

7 107834852 exm650490 A G NRCAM 12 35

7 107836311 exm650497 G C NRCAM 4 8

7 107850039 exm650518 G A NRCAM 6 5

7 108137038 exm650580 G A PNPLA8 7 19

7 108155026 exm650609 A G PNPLA8 4 4

7 110763811 exm650794 G A LRRN3 8 31

7 111580166 exm651034 G A DOCK4 0 2

7 111638537 exm651068 A G DOCK4 1 2

7 111935962 exm651102 A C ZNF277 1 8

7 111958309 exm651111 A C ZNF277 5 7

7 112097088 exm651170 G A IFRD1 0 1

7 112412892 exm651274 A G TMEM168 4 6

7 113518037 exm651395 G C PPP1R3A 0 1

7 113518262 exm651403 A G PPP1R3A 9 22

7 113518665 exm651428 G C PPP1R3A 9 29

7 113518880 exm651441 A G PPP1R3A 3 6

7 113519930 exm651483 C A PPP1R3A 9 11

7 114582402 exm651626 A T MDFIC 10 17

7 114582414 exm651628 A G MDFIC 1 10

7 116395422 exm651889 A G MET 11 22

7 116409777 exm651932 A G MET 8 23

7 116918397 exm652106 A G WNT2 1 1

7 116962978 exm652137 A C WNT2 2 7

7 117144390 exm652219 A G CFTR 0 2

7 117176711 exm652261 A T CFTR 7 12

7 117368227 exm652540 A G CTTNBP2 0 2

7 117400548 exm652585 G A CTTNBP2 5 5

7 117431308 exm652625 C G CTTNBP2 6 19

7 117431971 exm652652 G A CTTNBP2 2 1

7 117450854 exm652693 A G CTTNBP2 3 3

7 119915155 exm652806 A G KCND2 1 6

7 120382604 exm652839 A G KCND2 0 1

7 120428696 exm652867 G A TSPAN12 21 35

7 120478935 exm652897 A G TSPAN12 3 13

7 121650388 exm653289 A G PTPRZ1 8 13

7 121650421 exm653293 A G PTPRZ1 2 4

7 121651510 exm653346 A G PTPRZ1 1 1

7 121651826 exm653365 G A PTPRZ1 6 12

7 121652941 exm653398 A C PTPRZ1 3 6

7 121653331 exm653416 A G PTPRZ1 2 2

7 121699896 exm653534 C A PTPRZ1 3 9

7 121732911 exm653591 A G AASS 2 5

7 122047674 exm653739 G C CADPS2 2 8

7 122338323 exm653836 A G RNF133 0 1

7 122338516 exm653848 A C RNF133 12 39

7 122338770 exm653863 G A RNF133 8 25

7 122635027 exm653920 A G TAS2R16 11 29

7 122635208 exm653931 A G TAS2R16 6 12

7 122635349 exm653936 G A TAS2R16 11 29

7 122808603 exm654011 G C SLC13A1 0 2

7 122809286 exm654019 C G SLC13A1 2 8

7 123101414 exm654071 A G IQUB 9 19

7 123152210 exm654142 G A IQUB 0 2

7 123296084 exm654269 A G LMOD2 6 13

7 123302244 exm654287 A G LMOD2 7 5

7 123508328 exm654393 G A HYAL4 0 1

7 123508361 exm654394 G A HYAL4 23 38

7 123516944 exm654449 A G HYAL4 1 3

7 123593641 exm654464 G A SPAM1 3 4

7 123599571 exm654506 G A SPAM1 17 30

7 123599845 exm654516 T A SPAM1 4 3

7 124404339 exm654614 T A GPR37 2 2

7 124493144 exm654698 G A POT1 18 26

7 124503610 exm654713 G A POT1 6 7

7 126086242 exm654728 G A GRM8 9 16

7 126173556 exm654759 A G GRM8 28 47

7 126173898 exm654777 A G GRM8 7 22

7 127222544 exm654962 A G GCC1 14 32

7 127223107 exm654986 G A GCC1 3 7

7 127235509 exm655069 A G FSCN3 8 19

7 127251702 exm655160 A G PAX4 1 4

7 127255083 exm655200 A C PAX4 6 18

7 127668769 exm655300 A C LRRC4 19 33

7 127668907 exm655305 G A LRRC4 12 28

7 127668978 exm655310 G C LRRC4 3 11

7 128097359 exm655716 A G HILPDA 26 38

7 128097466 exm655722 A C HILPDA 14 17

7 128317782 exm655824 A C FAM71F2 4 7

7 128317797 exm655825 A G FAM71F2 0 3

7 128320127 exm655828 A G FAM71F2 2 3

7 128441408 exm656039 G A CCDC136 5 5

7 128446382 exm656070 G A CCDC136 4 12

7 128454863 exm656138 A G CCDC136 9 16

7 128454943 exm656144 C A CCDC136 11 15

7 128483567 exm656321 A G FLNC 4 12

7 128486412 exm656387 A G FLNC 18 35

7 129691082 exm657444 A G ZC3HC1 5 11

7 129856075 exm657577 C G SSMEM1 5 9

7 129856240 exm657584 A G SSMEM1 7 20

7 129951878 exm657722 A G CPA4 5 12

7 129999481 exm657770 A G CPA5 4 7

7 130002732 exm657786 A G CPA5 15 27

7 130038789 exm657920 C G CEP41 5 9

7 131189129 exm658281 A G PODXL 3 9

7 131190754 exm658299 A G PODXL 2 4

7 131191054 exm658307 A G PODXL 2 1

7 131191418 exm658314 A G PODXL 11 20

7 131194329 exm658343 A G PODXL 10 31

7 131196015 exm658374 G C PODXL 1 4

7 131815315 exm658404 A G PLXNA4 1 5

7 132070047 exm658637 A G PLXNA4 11 26

7 132193334 exm658708 G A PLXNA4 13 25

7 132481247 exm658740 A G CHCHD3 10 17

7 132481336 exm658746 A G CHCHD3 11 29

7 132959762 exm658785 A G EXOC4 9 29

7 133059694 exm658833 A C EXOC4 8 7

7 133622765 exm658894 C G EXOC4 3 12

7 133689783 exm658905 A G EXOC4 6 6

7 133821837 exm658956 A G LRGUK 13 33

7 133824207 exm658958 A G LRGUK 2 9

7 133884104 exm659023 G A LRGUK 12 36

7 133884144 exm659024 A G LRGUK 0 1

7 133886236 exm659027 A T LRGUK 13 28

7 133932345 exm659043 A G LRGUK 8 18

7 133943142 exm659057 A G LRGUK 0 1

7 133943151 exm659058 A G LRGUK 4 7

7 133948668 exm659064 G A LRGUK 6 10

7 133948672 exm659065 C G LRGUK 12 16

7 133979799 exm659085 G C SLC35B4 0 1

7 134001469 exm659127 A G SLC35B4 5 4

7 134130035 exm659137 A G AKR1B1 7 6

7 134135581 exm659169 A T AKR1B1 1 0

7 134135621 exm659176 G A AKR1B1 12 36

7 134215554 exm659214 A G AKR1B10 0 1

7 134216765 exm659220 A G AKR1B10 2 4

7 134618397 exm659411 C G CALD1 5 9

7 134719600 exm659505 A G AGBL3 11 20

7 134730317 exm659517 A G AGBL3 5 24

7 134874189 exm659640 A C WDR91 5 23

7 135255936 exm659847 A G NUP205 0 1

7 135261718 exm659862 A G NUP205 8 12

7 135282128 exm659923 A G NUP205 2 2

7 135303329 exm659997 G A NUP205 9 12

7 136700286 exm660279 T A CHRM2 4 7

7 136700385 exm660283 G A CHRM2 10 32

7 136700472 exm660284 G C CHRM2 1 11

7 137293774 exm660420 G A DGKI 1 0

7 137600758 exm660532 G A CREB3L2 4 12

7 137613006 exm660545 A G CREB3L2 9 10

7 138239499 exm660647 A G TRIM24 2 10

7 138239655 exm660653 G C TRIM24 5 17

7 138281268 exm660717 G A SVOPL 0 1

7 138305825 exm660725 A G SVOPL 2 13

7 138305838 exm660726 A G SVOPL 15 32

7 138329518 exm660769 A G SVOPL 1 0

7 138394472 exm660831 A G ATP6V0A4 6 17

7 138400611 exm660841 A G ATP6V0A4 5 13

7 138432174 exm660892 T A ATP6V0A4 2 3

7 138455976 exm660949 A G ATP6V0A4 1 3

7 138566179 exm661073 A G KIAA1549 2 8

7 138601515 exm661137 A G KIAA1549 7 9

7 138601683 exm661151 A G KIAA1549 1 1

7 138601730 exm661154 A G KIAA1549 8 9

7 138602267 exm661184 A T KIAA1549 8 16

7 138602570 exm661198 A G KIAA1549 7 15

7 138603048 exm661225 A C KIAA1549 3 4

7 138603236 exm661237 C G KIAA1549 13 12

7 138603441 exm661251 A G KIAA1549 3 12

7 138603662 exm661263 A C KIAA1549 7 11

7 138603680 exm661267 A G KIAA1549 19 26

7 138603968 exm661288 A C KIAA1549 2 18

7 138758679 exm661390 A G ZC3HAV1 5 5

7 138764878 exm661453 C G ZC3HAV1 6 14

7 138768744 exm661462 G A ZC3HAV1 6 18

7 138916654 exm661544 A C UBN2 9 16

7 138946363 exm661585 A G UBN2 9 24

7 139655310 exm662026 G C TBXAS1 11 21

7 139655339 exm662030 A T TBXAS1 2 3

7 139715566 exm662071 A G TBXAS1 11 25

7 139715645 exm662080 A C TBXAS1 13 18

7 139717602 exm662097 C A TBXAS1 1 2

7 139719829 exm662101 A G TBXAS1 6 10

7 139724496 exm662115 T A PARP12 1 2

7 139726120 exm662138 A G PARP12 0 1

7 139727184 exm662145 A G PARP12 10 20

7 140221837 exm662556 G A DENND2A 16 35

7 140285507 exm662642 A G DENND2A 3 11

7 140301503 exm662671 C A DENND2A 8 19

7 140301971 exm662695 A G DENND2A 2 2

7 140373873 exm662738 A G ADCK2 8 17

7 140379042 exm662758 A G ADCK2 5 16

7 140379043 exm662759 A G ADCK2 0 1

7 140390574 exm662790 C G ADCK2 0 1

7 141255283 exm662925 G A AGK 14 31

7 141255295 exm662927 G A AGK 7 24

7 141313971 exm662936 G C AGK 10 22

7 141341064 exm662954 C A AGK 8 10

7 141362538 exm662982 A G DENND11 10 12

7 141386452 exm663025 G A DENND11 0 1

7 141464029 exm663127 G A TAS2R3 5 5

7 141464050 exm663131 G A TAS2R3 8 18

7 141464307 exm663140 A G TAS2R3 8 12

7 141478859 exm663212 G A TAS2R4 5 7

7 141478953 exm663216 A G TAS2R4 0 4

7 141478964 exm663218 A G TAS2R4 0 4

7 141491042 exm663273 A C TAS2R5 12 26

7 141491049 exm663274 C G TAS2R5 12 13

7 141673251 exm663427 G A TAS2R38 3 5

7 141673299 exm663429 A G TAS2R38 9 19

7 141720785 exm663468 A G MGAM 17 36

7 141726957 exm663495 G A MGAM 3 13

7 141727444 exm663502 A G MGAM 10 27

7 141730471 exm663523 G A MGAM 18 40

7 141752139 exm663590 G A MGAM 6 6

7 141752722 exm663608 G A MGAM 21 51

7 141803238 exm663790 G A MGAM 4 12

7 142561922 exm664985 A G EPHB6 1 2

7 142562010 exm664993 A G EPHB6 9 17

7 142562484 exm665014 A G EPHB6 6 21

7 142566065 exm665074 A G EPHB6 4 9

7 142570231 exm665158 G A TRPV6 2 9

7 142574256 exm665234 A G TRPV6 4 4

7 142605840 exm665291 A G TRPV5 0 4

7 142625327 exm665352 A G TRPV5 4 3

7 142630489 exm665388 G A TRPV5 4 9

7 142641426 exm665487 A G KEL 3 10

7 142649646 exm665517 A G KEL 1 0

7 142655008 exm665554 A G KEL 13 37

7 142723454 exm665594 A G OR9A2 2 7

7 142723505 exm665596 G A OR9A2 2 7

7 142749822 exm665647 A G OR6V1 20 42

7 142836634 exm665707 A G PIP 4 21

7 142836665 exm665709 C G PIP 13 28

7 142880830 exm665723 A G TAS2R39 15 39

7 142881243 exm665740 C G TAS2R39 4 4

7 142919440 exm665757 G A TAS2R40 4 19

7 142919988 exm665777 G A TAS2R40 14 32

7 142962325 exm665809 A G GSTK1 4 8

7 142965902 exm665820 G A GSTK1 1 2

7 142985603 exm665861 A G CASP2 1 7

7 142991379 exm665891 G C CASP2 0 1

7 142997061 exm665906 C G CASP2 15 34

7 143029874 exm666028 A G CLCN1 2 2

7 143036612 exm666043 G A CLCN1 2 9

7 143049017 exm666122 A G CLCN1 11 24

7 143079922 exm666201 C A ZYX 2 2

7 143079970 exm666204 A G ZYX 12 34

7 143080092 exm666210 G C ZYX 5 6

7 143088584 exm666292 A G EPHA1 0 1

7 143091417 exm666326 A G EPHA1 3 4

7 143091962 exm666334 C A EPHA1 9 22

7 143092269 exm666348 A G EPHA1 27 47

7 143094693 exm666382 G C EPHA1 34 54

7 143095467 exm666404 A G EPHA1 4 1

7 143098598 exm666485 G A EPHA1 1 3

7 143140675 exm666501 A G TAS2R60 3 6

7 143141109 exm666518 G C TAS2R60 5 10

7 143141230 exm666525 A C TAS2R60 32 58

7 143657104 exm666798 G A OR2F1 6 16

7 143657535 exm666818 A G OR2F1 1 4

7 143657635 exm666825 G A OR2F1 11 22

7 143747667 exm666901 G A OR2A5 2 11

7 143747799 exm666910 G A OR2A5 23 36

7 143748430 exm666951 G A OR2A5 10 21

7 143827134 exm667157 A G OR2A14 5 21

7 144094418 exm667386 G A NOBOX 28 47

7 144096892 exm667424 C A NOBOX 3 1

7 144097213 exm667428 A G NOBOX 2 8

7 144098621 exm667457 A G NOBOX 6 13

7 146741075 exm667571 A G CNTNAP2 0 1

7 146829473 exm667606 G A CNTNAP2 9 21

7 147600681 exm667660 G A CNTNAP2 7 23

7 147914562 exm667716 A C CNTNAP2 19 44

7 147926795 exm667720 G A CNTNAP2 20 40

7 148701005 exm667965 A C PDIA4 20 41

7 148703121 exm668017 G A PDIA4 3 6

7 148709067 exm668039 A G PDIA4 0 2

7 148712113 exm668069 G A PDIA4 6 10

7 148718200 exm668086 C A PDIA4 12 22

7 148801033 exm668195 G C ZNF425 3 5

7 148801971 exm668248 G A ZNF425 9 16

7 148851037 exm668304 G A ZNF398 0 1

7 148875839 exm668342 C G ZNF398 4 7

7 148949845 exm668526 G A ZNF212 2 5

7 148950740 exm668534 G A ZNF212 11 26

7 149133917 exm668727 A G ZNF777 1 0

7 149463204 exm669185 A T ZNF467 1 4

7 149463266 exm669186 A C ZNF467 1 1

7 149557722 exm670288 G A ZNF862 22 47

7 149559029 exm670346 A G ZNF862 3 4

7 149561323 exm670387 A G ZNF862 13 42

7 150034453 exm670519 A G LRRC61 11 17

7 150034722 exm670530 A G LRRC61 16 33

7 150163799 exm670697 G A GIMAP8 5 10

7 150164342 exm670723 A G GIMAP8 5 23

7 150171165 exm670728 A G GIMAP8 2 6

7 150174540 exm670765 A C GIMAP8 10 24

7 150217627 exm670813 G A GIMAP7 3 10

7 150217735 exm670818 G A GIMAP7 18 44

7 150390040 exm670987 G A GIMAP2 12 21

7 150390255 exm670996 G A GIMAP2 16 35

7 150490238 exm671149 A G TMEM176B 20 38

7 150493495 exm671185 A G TMEM176B 0 1

7 150493585 exm671189 A G TMEM176B 0 3

7 150500527 exm671225 A G TMEM176A 6 10

7 150501508 exm671248 A C TMEM176A 1 1

7 150721071 exm671870 A G ATG9B 3 5

7 150731483 exm671947 C A ABCB8 7 17

7 150732987 exm671979 A G ABCB8 1 2

7 150741183 exm672048 A G ABCB8 11 29

7 150934709 exm672954 A G CHPF2 5 14

7 151071225 exm673141 A G NUB1 10 21

7 151072995 exm673147 A G NUB1 6 16

7 151073747 exm673151 A G NUB1 2 3

7 151684294 exm673743 A G GALNTL5 34 59

7 151700036 exm673764 A G GALNTL5 8 19

7 151716755 exm673778 A G GALNTL5 4 14

7 151805206 exm673874 G C GALNT11 0 1

7 151859495 exm674067 C G KMT2C 17 39

7 151859683 exm674078 A G KMT2C 0 5

7 151877127 exm674230 A C KMT2C 12 15

7 151878292 exm674251 C A KMT2C 14 22

7 151945503 exm674485 T A KMT2C 9 31

7 151946979 exm674493 A C KMT2C 13 24

7 151949735 exm674511 G A KMT2C 2 3

7 152007115 exm674544 G A KMT2C 1 2

7 154645534 exm674901 C A DPP6 21 37

7 154863097 exm675123 A G HTR5A 7 19

7 155537824 exm675418 A G RBM33 3 11

7 155556681 exm675443 G A RBM33 0 1

7 156451209 exm675556 A G RNF32 10 24

7 156468480 exm675568 A G RNF32 1 1

7 156742676 exm675681 A G NOM1 17 28

7 156752836 exm675753 A G NOM1 4 14

7 156755825 exm675780 A G NOM1 3 2

7 156756633 exm675797 A T NOM1 1 0

7 156759050 exm675808 G A NOM1 6 6

7 156759675 exm675813 G A NOM1 5 10

7 157931073 exm676245 G C PTPRN2 2 8

7 158454921 exm676424 A G NCAPG2 23 33

7 158472777 exm676465 A G NCAPG2 6 14

7 158677277 exm676699 A C WDR60 15 29

7 158679717 exm676702 A G WDR60 2 12

7 158698692 exm676718 A C WDR60 18 36

7 158706999 exm676738 A G WDR60 8 22

7 158711522 exm676744 A G WDR60 1 5

7 158718971 exm676764 A G WDR60 8 28

7 158719119 exm676772 A G WDR60 4 3

7 23240263 JHU_7.23240262 A G NUP42 2 7

7 92732769 JHU_7.92732768 G A SAMD9 9 17

7 99308452 kgp13727307 A C CYP3A7;CYP3A7-CYP3A51P 1 5

7 123610273 newrs111754670 A G SPAM1 4 5

7 44089863 newrs145680549 A G DBNL 3 6

7 20687233 newrs150279505 A G ABCB5 11 23

7 87035792 rs31655 A G ABCB4 7 11

7 20180774 variant.94795 A G MACC1 0 4

8 100654621 8:100654621-TG C A VPS13B 1 4

8 101153590 8:101153590-TC G A FBXO43 0 1

8 101620766 8:101620766-CT A G SNX31 0 1

8 10468054 8:10468054-CT A G RP1L1 9 27

8 10469534 8:10469534-GA A G RP1L1 0 1

8 10623383 8:10623383-GT A C PINX1 4 8

8 110463249 8:110463249-AG G A PKHD1L1 10 19

8 110493753 8:110493753-CA A C PKHD1L1 4 5

8 124138858 8:124138858-GA A G TBC1D31 12 32

8 124665124 8:124665124-CT A G KLHL38 7 14

8 131073268 8:131073268-GA A G ASAP1 1 1

8 132999877 8:132999877-AG G A EFR3A 7 14

8 133058101 8:133058101-GA A G OC90 1 6

8 133851776 8:133851776-AG G A PHF20L1 2 10

8 133881996 8:133881996-GA A G TG 16 34

8 139662011 8:139662011-TC G A COL22A1 9 11

8 142204312 8:142204312-GA A G DENND3 3 3

8 144812319 8:144812319-CT A G FAM83H 1 0

8 144892902 8:144892902-GA A G SCRIB 1 1

8 145541782 8:145541782-AG G A DGAT1 0 2

8 145625831 8:145625831-GA A G CPSF1 1 6

8 145738349 8:145738349-GT A C RECQL4 26 55

8 146157700 8:146157700-AC C A ZNF16 9 20

8 146171408 8:146171408-TG C A ZNF16 1 12

8 146278038 8:146278038-GA A G C8orf33 8 15

8 17612730 8:17612730-CT A G MTUS1 9 15

8 17731935 8:17731935-TC G A FGL1 10 16

8 17916924 8:17916924-GA A G ASAH1 1 0

8 2088653 8:2088653-AG G A MYOM2 11 12

8 21964876 8:21964876-CT A G NUDT18 16 32

8 22005713 8:22005713-GA A G LGI3 5 11

8 22452101 8:22452101-TC G A PDLIM2 3 7

8 22471761 8:22471761-GT A C CCAR2 10 13

8 23012480 8:23012480-AG G A TNFRSF10D 2 3

8 24775215 8:24775215-CT A G NEFM 1 5

8 25268713 8:25268713-GA A G DOCK5 4 15

8 25365046 8:25365046-CT A G CDCA2 16 25

8 27146788 8:27146788-GA A G TRIM35 9 22

8 27394367 8:27394367-GA A G EPHX2 6 28

8 28575273 8:28575273-AG G A EXTL3 1 1

8 2876157 8:2876157-CT A G CSMD1 1 3

8 30921921 8:30921921-GA A G WRN 2 6

8 37732721 8:37732721-GA A G RAB11FIP1 3 4

8 38385908 8:38385908-CT A G C8orf86 19 43

8 38677111 8:38677111-CT A G TACC1 2 1

8 38873678 8:38873678-TG C A ADAM9 1 0

8 39103645 8:39103645-CT A G ADAM32 18 36

8 41791883 8:41791883-CA A C KAT6A 9 9

8 48730122 8:48730122-CT A G PRKDC 9 17

8 48752626 8:48752626-AG G A PRKDC 1 3

8 48771426 8:48771426-GA A G PRKDC 2 4

8 48802948 8:48802948-CA A C PRKDC 9 31

8 55537354 8:55537354-GT A C RP1 0 2

8 57353988 8:57353988-CT A G PENK 7 20

8 61765534 8:61765534-AG G A CHD7 3 2

8 6302592 8:6302592-AC C A MCPH1 8 23

8 6728339 8:6728339-AC C A DEFB1 2 5

8 68004071 8:68004071-TC G A CSPP1 3 10

8 71619272 8:71619272-AG G A XKR9 28 38

8 72938260 8:72938260-GA A G TRPA1 5 15

8 77765398 8:77765398-AG G A ZFHX4 26 57

8 82437325 8:82437325-GA A G FABP12 7 9

8 82665301 8:82665301-GA A G CHMP4C 2 11

8 90967686 8:90967686-TC G A NBN 12 21

8 92401580 8:92401580-TC G A SLC26A7 0 2

8 95518762 8:95518762-CT A G VIRMA 0 1

8 97243340 8:97243340-CT A G UQCRB 11 22

8 99140692 8:99140692-GA A G POP1 1 1

8 11637261 exm2161283 G A NEIL2 27 55

8 1806227 exm2168006 A G ARHGEF10 4 12

8 22880453 exm2169606 A G TNFRSF10B 1 0

8 27634171 exm2170749 A G ESCO2 0 2

8 30703730 exm2171426 G A TEX15 1 3

8 39624429 exm2172642 A G ADAM2 0 1

8 39780133 exm2172694 C G IDO1 0 1

8 56699587 exm2174247 A G TGS1 2 1

8 68018178 exm2175449 G A CSPP1 11 26

8 99158904 exm2178451 A G POP1 2 1

8 133036775 exm2246462 A G OC90 6 12

8 17883113 exm2246700 G A PCM1 1 1

8 39486932 exm2246924 G A ADAM18 1 4

8 618626 exm677142 A C ERICH1 0 1

8 618700 exm677148 A C ERICH1 9 18

8 618756 exm677155 A T ERICH1 4 10

8 1719270 exm677441 G A CLN8 0 2

8 1719510 exm677458 A G CLN8 14 32

8 1728651 exm677492 A G CLN8 0 2

8 1808209 exm677536 A G ARHGEF10 12 17

8 1846672 exm677648 A T ARHGEF10 9 24

8 1853782 exm677659 G A ARHGEF10 8 16

8 1893743 exm677762 A C ARHGEF10 2 9

8 1893794 exm677764 A G ARHGEF10 11 22

8 1905038 exm677791 G A ARHGEF10 5 16

8 1905080 exm677794 G C ARHGEF10 1 3

8 1905157 exm677802 A G ARHGEF10 14 25

8 2000398 exm677902 A G MYOM2 3 12

8 2000409 exm677905 A G MYOM2 16 35

8 2005888 exm677937 A G MYOM2 13 29

8 2021518 exm677998 T A MYOM2 6 13

8 2027658 exm678042 A G MYOM2 1 4

8 2037988 exm678080 A C MYOM2 12 25

8 2041894 exm678114 A G MYOM2 11 36

8 2054382 exm678219 A C MYOM2 2 11

8 2092894 exm678389 A G MYOM2 0 1

8 2820084 exm678450 C A CSMD1 26 35

8 2823344 exm678474 A G CSMD1 1 6

8 2949111 exm678599 G C CSMD1 14 38

8 3059201 exm678745 C G CSMD1 6 16

8 3063043 exm678748 A G CSMD1 1 5

8 3076959 exm678772 G A CSMD1 33 53

8 3166014 exm678829 A C CSMD1 13 36

8 3351163 exm678990 G C CSMD1 2 3

8 3432493 exm678997 G C CSMD1 3 9

8 3855584 exm679054 A G CSMD1 6 13

8 6289091 exm679136 C G MCPH1 19 32

8 6302304 exm679183 G A MCPH1 5 11

8 6335130 exm679246 A G MCPH1 1 7

8 6690413 exm679587 A G XKR5 1 4

8 6728222 exm679597 C A DEFB1 1 5

8 8175717 exm680041 A G PRAG1 1 5

8 8185746 exm680139 G A PRAG1 1 0

8 8197002 exm680158 A G PRAG1 2 3

8 8233872 exm680179 C A PRAG1 7 17

8 8234682 exm680248 A G PRAG1 17 34

8 8234980 exm680272 G C PRAG1 0 1

8 8235297 exm680295 A G PRAG1 0 1

8 8239085 exm680331 G C PRAG1 10 14

8 8748068 exm680432 G A MFHAS1 10 31

8 8748849 exm680473 C A MFHAS1 11 24

8 10466128 exm681187 A G RP1L1 3 6

8 10467248 exm681291 A G RP1L1 0 1

8 10468451 exm681410 A G RP1L1 2 4

8 10470632 exm681590 G A RP1L1 1 1

8 10474055 exm681624 A C RP1L1 3 10

8 10480186 exm681647 G A RP1L1 1 0

8 10480189 exm681648 G A RP1L1 4 23

8 10480582 exm681712 G C RP1L1 24 43

8 10622930 exm681870 G A PINX1 11 29

8 10623240 exm681891 G A PINX1 1 3

8 10623254 exm681893 A C PINX1 1 7

8 11405588 exm682374 G C BLK 12 20

8 11412353 exm682402 C G BLK 4 8

8 11628991 exm682565 G A NEIL2 1 3

8 11637173 exm682580 C G NEIL2 12 26

8 11637290 exm682591 A G NEIL2 8 17

8 11643553 exm682629 A C NEIL2 1 0

8 11643672 exm682635 A C NEIL2 2 9

8 11643693 exm682637 A C NEIL2 7 18

8 12870279 exm683366 A G TRMT9B 5 16

8 12879276 exm683434 A C TRMT9B 27 52

8 12947856 exm683486 A C DLC1 11 25

8 12957699 exm683607 G A DLC1 6 13

8 13251126 exm683681 A G DLC1 28 40

8 13259100 exm683699 A G DLC1 26 40

8 13356962 exm683731 C A DLC1 1 7

8 13357414 exm683754 G A DLC1 9 21

8 13357502 exm683761 A G DLC1 6 22

8 16012615 exm683977 A C MSR1 7 13

8 16026056 exm683996 G A MSR1 5 14

8 16032710 exm684016 G A MSR1 3 8

8 16032791 exm684025 A C MSR1 0 6

8 17396380 exm684430 A G SLC7A2 18 48

8 17419461 exm684549 A G SLC7A2 23 44

8 17419551 exm684556 A G SLC7A2 8 20

8 17422602 exm684575 A G SLC7A2 8 17

8 17478685 exm684633 A G PDGFRL 9 29

8 17486068 exm684637 G A PDGFRL 15 31

8 17486166 exm684646 A G PDGFRL 4 9

8 17510766 exm684716 G C MTUS1 26 37

8 17513386 exm684737 C G MTUS1 6 10

8 17532727 exm684757 A G MTUS1 4 15

8 17541944 exm684771 C A MTUS1 7 13

8 17542024 exm684780 A G MTUS1 1 7

8 17573401 exm684807 A C MTUS1 11 18

8 17611789 exm684892 A G MTUS1 6 18

8 17612019 exm684918 G A MTUS1 3 9

8 17612134 exm684928 C A MTUS1 13 24

8 17612782 exm684979 A G MTUS1 0 1

8 17612838 exm684981 A G MTUS1 1 7

8 17612849 exm684983 C G MTUS1 2 7

8 17613310 exm685014 A G MTUS1 1 7

8 17722162 exm685023 G A FGL1 5 7

8 17726069 exm685038 A C FGL1 1 2

8 17731914 exm685077 A T FGL1 12 24

8 17739594 exm685101 G A FGL1 3 5

8 17739639 exm685111 A C FGL1 14 32

8 17739672 exm685115 A C FGL1 2 8

8 17743051 exm685126 T A FGL1 13 25

8 17804833 exm685189 C G PCM1 3 3

8 17804851 exm685190 G A PCM1 0 2

8 17814898 exm685234 C A PCM1 1 0

8 17817635 exm685262 G A PCM1 5 7

8 17867185 exm685399 A T PCM1 4 16

8 17868817 exm685411 C A PCM1 0 1

8 17871528 exm685425 A G PCM1 8 11

8 17924739 exm685539 T A ASAH1 12 17

8 18257583 exm685657 T A NAT2 2 2

8 18257985 exm685673 C A NAT2 6 19

8 18258091 exm685680 A G NAT2 5 4

8 18258196 exm685692 A G NAT2 8 18

8 18258279 exm685698 G A NAT2 14 31

8 18658794 exm685775 A G PSD3 3 2

8 18725224 exm685811 C A PSD3 10 31

8 18725253 exm685813 A G PSD3 7 12

8 18729577 exm685866 G A PSD3 2 2

8 19192358 exm685958 A T SH2D4A 13 17

8 19218745 exm685970 G A SH2D4A 7 16

8 19221726 exm685996 A G SH2D4A 5 6

8 19263451 exm686052 A G CSGALNACT1 6 20

8 19362918 exm686130 G A CSGALNACT1 3 8

8 19363116 exm686144 A G CSGALNACT1 1 0

8 19690816 exm686233 A G INTS10 3 4

8 20004884 exm686369 A G SLC18A1 9 14

8 20030603 exm686427 G A SLC18A1 11 16

8 20107433 exm686572 A G LZTS1 0 1

8 20107772 exm686597 C G LZTS1 0 2

8 21769432 exm686790 G A DOK2 0 1

8 21883181 exm686931 C A NPM2 6 12

8 21965274 exm687212 A G NUDT18 4 12

8 22006170 exm687554 A G LGI3 1 1

8 22011662 exm687607 G A LGI3 6 7

8 22031177 exm687695 A C BMP1 11 25

8 22031205 exm687698 A G BMP1 34 42

8 22033703 exm687700 A G BMP1 4 2

8 22058684 exm687839 A G BMP1 11 32

8 22059345 exm687851 A G BMP1 0 1

8 22064886 exm687874 A G BMP1 0 3

8 22422009 exm688394 G C SORBS3 15 18

8 22422030 exm688396 G A SORBS3 4 10

8 22432189 exm688479 A G SORBS3 13 16

8 22447142 exm688531 G C PDLIM2 3 14

8 22473646 exm688748 C A CCAR2 3 5

8 22476360 exm688793 C G CCAR2 6 13

8 22476708 exm688808 A G CCAR2 1 0

8 22481833 exm688859 A G BIN3 1 0

8 22494441 exm688876 G A BIN3 3 8

8 22582461 exm688954 C G PEBP4 19 39

8 22675174 exm688964 A T PEBP4 1 1

8 22785097 exm688978 G A PEBP4 1 0

8 22785182 exm688985 A G PEBP4 19 36

8 22885246 exm689153 A G TNFRSF10B 4 4

8 22900701 exm689184 A G TNFRSF10B 4 10

8 23054646 exm689367 A C TNFRSF10A 4 5

8 23054713 exm689371 A G TNFRSF10A 0 1

8 23069635 exm689416 A G TNFRSF10A 21 40

8 23174557 exm689607 A G LOXL2 0 1

8 23225791 exm689705 A C LOXL2 4 2

8 23294661 exm689757 A G ENTPD4 0 2

8 23306403 exm689809 A G ENTPD4 7 10

8 24170994 exm689954 A C ADAM28 1 3

8 24200656 exm690038 C A ADAM28 2 6

8 24207470 exm690052 G A ADAM28 0 1

8 24323219 exm690202 A G ADAM7 2 5

8 24326313 exm690224 G A ADAM7 9 24

8 24342812 exm690245 A G ADAM7 6 26

8 24349562 exm690276 A G ADAM7 2 10

8 24357716 exm690298 A T ADAM7 11 21

8 24775155 exm690391 A C NEFM 17 35

8 24775477 exm690411 A C NEFM 7 16

8 25174610 exm690576 A G DOCK5 0 1

8 25222153 exm690644 A G DOCK5 11 23

8 25246686 exm690697 G A DOCK5 5 12

8 25261108 exm690738 A G DOCK5 0 2

8 25267684 exm690763 G A DOCK5 1 8

8 25343296 exm690919 A G CDCA2 11 16

8 25364343 exm690957 A G CDCA2 21 38

8 26366103 exm691204 A G PNMA2 1 4

8 26627944 exm691347 A C ADRA1A 17 22

8 27156013 exm691524 A G TRIM35 10 16

8 27168348 exm691531 C G TRIM35 9 21

8 27168512 exm691538 C A TRIM35 6 18

8 27293810 exm691637 A G PTK2B 11 19

8 27295333 exm691647 A G PTK2B 2 6

8 27362515 exm691885 A G EPHX2 0 1

8 27373864 exm691921 A G EPHX2 2 1

8 27399010 exm691958 A G EPHX2 0 3

8 27402007 exm691979 C G EPHX2 4 22

8 27402020 exm691981 A G EPHX2 1 2

8 27634331 exm692270 A G ESCO2 24 36

8 27645464 exm692299 C A ESCO2 12 33

8 27779093 exm692416 A G SCARA5 3 7

8 27779648 exm692445 A G SCARA5 6 14

8 27824039 exm692456 A G SCARA5 11 25

8 27950736 exm692594 A G ELP3 6 9

8 28304779 exm692813 C G FBXO16 5 17

8 28314293 exm692824 G C FBXO16 1 3

8 28638369 exm693098 G A INTS9 11 10

8 28695158 exm693134 A G INTS9 15 26

8 28965673 exm693251 G A KIF13B 0 3

8 29006153 exm693338 T A KIF13B 4 20

8 29025045 exm693368 A G KIF13B 24 51

8 30550502 exm693741 G A GSR 16 35

8 30553995 exm693754 A G GSR 2 6

8 30694618 exm693878 G A TEX15 1 0

8 30700170 exm693950 A G TEX15 10 25

8 30700248 exm693953 C A TEX15 3 5

8 30700379 exm693956 G A TEX15 3 5

8 30700439 exm693958 G A TEX15 10 22

8 30701210 exm693987 C G TEX15 2 6

8 30701995 exm694014 C A TEX15 27 66

8 30702165 exm694018 A G TEX15 1 1

8 30702695 exm694038 G A TEX15 6 9

8 30703991 exm694103 C G TEX15 5 10

8 30704897 exm694146 G A TEX15 12 26

8 30705249 exm694160 T A TEX15 17 32

8 30705342 exm694162 C A TEX15 1 8

8 30705815 exm694174 G A TEX15 4 24

8 30706073 exm694189 G A TEX15 7 10

8 30706446 exm694207 C A TEX15 9 20

8 30916679 exm694259 A G WRN 8 12

8 30922521 exm694278 A G WRN 5 7

8 30925839 exm694293 C A WRN 7 5

8 30938570 exm694308 A G WRN 0 1

8 30954294 exm694357 A G WRN 6 16

8 30999037 exm694410 A G WRN 2 7

8 32505390 exm694539 A G NRG1 12 23

8 32505432 exm694541 A C NRG1 7 16

8 32621349 exm694614 G A NRG1 1 2

8 36642002 exm695107 A G KCNU1 7 8

8 36763273 exm695198 C G KCNU1 4 3

8 37688966 exm695474 A G ADGRA2 8 9

8 37689038 exm695477 A G ADGRA2 3 1

8 37702445 exm695675 A G BRF2 3 2

8 37728950 exm695763 A G RAB11FIP1 4 4

8 37729777 exm695815 A G RAB11FIP1 1 1

8 37729898 exm695823 A G RAB11FIP1 0 6

8 37729955 exm695828 A G RAB11FIP1 10 26

8 37730200 exm695844 A G RAB11FIP1 0 2

8 37732240 exm695880 A G RAB11FIP1 6 18

8 37734755 exm695916 A G RAB11FIP1 8 8

8 38065205 exm696214 A G BAG4 2 3

8 38091918 exm696250 A G DDHD2 12 24

8 38139080 exm696386 A G NSD3 19 33

8 38173493 exm696435 C A NSD3 6 18

8 38184289 exm696445 A G NSD3 4 10

8 38205569 exm696478 A G NSD3 4 15

8 38257914 exm696496 G C LETM2 6 17

8 38258455 exm696501 A G LETM2 8 20

8 38369982 exm696679 G C C8orf86 0 1

8 38373958 exm696696 A T C8orf86 7 14

8 38385917 exm696709 A G C8orf86 2 3

8 38677745 exm696777 A G TACC1 1 5

8 38677907 exm696784 A G TACC1 9 26

8 38678000 exm696792 A G TACC1 20 33

8 38693758 exm696823 A G TACC1 1 4

8 38700814 exm696839 C A TACC1 4 14

8 38869207 exm696986 A G ADAM9 5 1

8 38948816 exm697067 A G ADAM9 2 7

8 39022312 exm697112 G A ADAM32 4 6

8 39022585 exm697114 A T ADAM32 15 25

8 39068671 exm697129 G A ADAM32 16 23

8 39468070 exm697236 C A ADAM18 18 35

8 39525632 exm697287 A G ADAM18 0 1

8 39535051 exm697297 A G ADAM18 4 12

8 39645629 exm697411 A G ADAM2 2 2

8 39695662 exm697450 A G ADAM2 6 14

8 39780089 exm697475 G C IDO1 23 31

8 39862893 exm697551 A T IDO2 12 16

8 39871107 exm697555 A C IDO2 20 34

8 39871115 exm697556 A C IDO2 20 33

8 41519348 exm697831 A G ANK1 19 30

8 41543675 exm697908 A G ANK1 21 54

8 41834732 exm698395 A G KAT6A 18 31

8 42039506 exm698522 A G PLAT 0 1

8 42044965 exm698550 A G PLAT 13 28

8 42048904 exm698574 A C PLAT 7 25

8 42173814 exm698620 A G IKBKB 2 3

8 42294832 exm698825 A G SLC20A2 1 8

8 42297141 exm698855 A G SLC20A2 12 29

8 42977853 exm699261 G A POMK 15 29

8 42977869 exm699262 G A POMK 15 36

8 42977973 exm699266 A G POMK 7 16

8 48775016 exm699786 G A PRKDC 15 35

8 48852225 exm699964 A G PRKDC 16 19

8 48877161 exm700026 A G MCM4 1 1

8 48877227 exm700027 G A MCM4 2 2

8 48885469 exm700090 A G MCM4 1 2

8 48889280 exm700124 A G MCM4 24 57

8 52284560 exm700344 A T PXDNL 13 25

8 52287175 exm700351 A G PXDNL 0 1

8 52320657 exm700364 C A PXDNL 4 4

8 52321470 exm700400 A G PXDNL 2 4

8 52336225 exm700456 A G PXDNL 2 9

8 52336255 exm700459 A C PXDNL 8 16

8 52359719 exm700469 A C PXDNL 6 23

8 52361678 exm700477 A G PXDNL 0 1

8 52384792 exm700501 A G PXDNL 1 0

8 53025889 exm700607 G A ST18 3 9

8 53071616 exm700663 A G ST18 4 10

8 53073986 exm700668 A G ST18 3 4

8 53084357 exm700692 G A ST18 11 19

8 53084540 exm700702 A G ST18 7 14

8 53092685 exm700728 A G ST18 11 24

8 53586530 exm700925 G A RB1CC1 7 9

8 53596481 exm700951 A G RB1CC1 7 9

8 55049089 exm701265 A G MRPL15 14 25

8 55059962 exm701294 A C MRPL15 5 11

8 55537629 exm701401 A G RP1 3 19

8 55538687 exm701423 A G RP1 9 14

8 55538891 exm701431 G A RP1 4 5

8 55539574 exm701458 C A RP1 9 20

8 55540418 exm701486 C G RP1 1 6

8 55540472 exm701489 G A RP1 4 5

8 55540915 exm701504 C A RP1 1 4

8 55540953 exm701506 G A RP1 7 13

8 55541396 exm701523 A G RP1 13 22

8 55542143 exm701557 A G RP1 7 32

8 55542609 exm701582 A G RP1 2 19

8 56698298 exm701685 C G TGS1 0 1

8 56698945 exm701708 G A TGS1 10 23

8 56702797 exm701756 G A TGS1 5 9

8 56702798 exm701757 G C TGS1 13 32

8 56711671 exm701788 A G TGS1 14 36

8 56711690 exm701790 G A TGS1 9 24

8 57228723 exm702034 A G SDR16C5 7 26

8 57354172 exm702095 A G PENK 6 17

8 57354340 exm702107 G A PENK 2 3

8 59059214 exm702192 A G FAM110B 4 11

8 59059228 exm702194 A G FAM110B 3 10

8 59343215 exm702226 C A UBXN2B 7 16

8 59346981 exm702232 G A UBXN2B 1 0

8 59496687 exm702377 G A NSMAF 3 6

8 59498295 exm702382 A G NSMAF 7 9

8 59513865 exm702452 C A NSMAF 0 3

8 59728090 exm702545 A G TOX 0 2

8 59851907 exm702578 G A TOX 2 8

8 59852001 exm702586 G A TOX 3 8

8 61654703 exm702695 A G CHD7 6 11

8 61714146 exm702769 T A CHD7 13 15

8 61743055 exm702805 A G CHD7 17 33

8 61765767 exm702891 T A CHD7 3 5

8 61768682 exm702910 A G CHD7 6 5

8 62563618 exm703140 A G ASPH 6 10

8 63492175 exm703217 G A NKAIN3 17 32

8 63659683 exm703229 A G NKAIN3 6 10

8 66631598 exm703541 A G PDE7A 6 9

8 66692011 exm703585 A G PDE7A 0 1

8 66963810 exm703595 G C DNAJC5B 5 22

8 66963814 exm703596 A G DNAJC5B 1 2

8 66989037 exm703610 C G DNAJC5B 0 1

8 67061980 exm703664 A G TRIM55 9 15

8 67066435 exm703705 A G TRIM55 5 12

8 67356627 exm703812 G C ADHFE1 2 4

8 67357484 exm703822 A G ADHFE1 5 9

8 67357506 exm703823 A C ADHFE1 0 1

8 67478431 exm703923 C A MYBL1 18 44

8 67488288 exm703938 A G MYBL1 3 7

8 67505363 exm703961 T A MYBL1 9 19

8 67786401 exm704157 A G MCMDC2 0 1

8 67789687 exm704176 G A MCMDC2 6 5

8 67796120 exm704202 C G MCMDC2 20 49

8 68007676 exm704337 A C CSPP1 8 17

8 68007859 exm704343 A G CSPP1 17 22

8 68074097 exm704419 C A CSPP1 17 35

8 68113722 exm704474 C G ARFGEF1 3 8

8 68204111 exm704584 A G ARFGEF1 3 9

8 68346398 exm704658 A G CPA6 0 7

8 68396032 exm704660 A G CPA6 9 27

8 68421768 exm704684 G C CPA6 18 47

8 68930106 exm704722 A G PREX2 4 12

8 68942817 exm704743 G A PREX2 2 20

8 69017584 exm704860 A C PREX2 1 1

8 69020558 exm704874 A G PREX2 19 38

8 70533373 exm705101 A G SULF1 7 24

8 74600942 exm706217 A G STAU2 3 7

8 74893653 exm706295 A G TMEM70 6 13

8 75737657 exm706483 A G PI15 13 22

8 75737689 exm706485 A G PI15 8 14

8 77616519 exm706654 G C ZFHX4 14 20

8 77761919 exm706786 A G ZFHX4 2 5

8 77764484 exm706840 G A ZFHX4 0 1

8 77766749 exm706914 A G ZFHX4 6 11

8 77768390 exm706965 C A ZFHX4 26 57

8 77775350 exm706972 A G ZFHX4 14 22

8 77776491 exm707021 G C ZFHX4 1 5

8 82441724 exm707610 C A FABP12 13 27

8 82441816 exm707614 A G FABP12 11 21

8 82606946 exm707705 G A SLC10A5 3 20

8 82667691 exm707766 A G CHMP4C 22 34

8 86021994 exm707885 A G LRRCC1 14 27

8 86025310 exm707892 A G LRRCC1 12 31

8 86049731 exm707961 A G LRRCC1 4 8

8 86050583 exm707971 A G LRRCC1 5 12

8 86360293 exm708155 A G CA3 3 10

8 87060725 exm708204 A G PSKH2 1 5

8 87076415 exm708232 A G PSKH2 2 12

8 87076650 exm708247 C A PSKH2 8 17

8 87076810 exm708256 A G PSKH2 11 17

8 87226866 exm708340 G A SLC7A13 32 52

8 87229732 exm708344 C A SLC7A13 1 6

8 87229946 exm708351 A G SLC7A13 1 3

8 87241965 exm708375 A T SLC7A13 12 24

8 87543456 exm708551 A G CPNE3 20 37

8 87560599 exm708584 G A CPNE3 3 4

8 87567100 exm708605 A G CPNE3 17 31

8 87567156 exm708609 G A CPNE3 5 15

8 87567168 exm708610 A G CPNE3 6 14

8 87588303 exm708657 G A CNGB3 7 14

8 87641188 exm708698 A G CNGB3 13 32

8 87641222 exm708703 C A CNGB3 15 22

8 87656040 exm708718 G A CNGB3 1 1

8 87917373 exm708799 A C CNBD1 16 18

8 87951922 exm708805 G A CNBD1 1 5

8 88218342 exm708811 A G CNBD1 14 24

8 88298852 exm708837 G A CNBD1 18 36

8 90965540 exm709116 C G NBN 6 17

8 90967554 exm709126 C A NBN 3 2

8 90967591 exm709129 G A NBN 2 16

8 90982700 exm709155 G A NBN 7 17

8 92307854 exm709482 A G SLC26A7 6 9

8 92365184 exm709532 A G SLC26A7 6 11

8 92378838 exm709544 G A SLC26A7 1 2

8 95161082 exm710028 A C CDH17 3 4

8 95183099 exm710083 A G CDH17 5 9

8 95183176 exm710087 G A CDH17 2 4

8 95183191 exm710089 A G CDH17 19 32

8 95186213 exm710097 A T CDH17 2 3

8 95262626 exm710127 A G GEM 4 4

8 95262656 exm710129 A C GEM 7 20

8 95399369 exm710204 C A RAD54B 4 12

8 95404046 exm710214 G A RAD54B 3 10

8 95404100 exm710217 A G RAD54B 4 12

8 95419819 exm710252 G A RAD54B 5 6

8 95470528 exm710267 A G RAD54B 1 5

8 95479680 exm710273 C G RAD54B 26 29

8 95531387 exm710397 A G VIRMA 8 24

8 95538641 exm710422 G A VIRMA 7 24

8 95541487 exm710460 G C VIRMA 7 19

8 95853722 exm710693 A C INTS8 0 1

8 95884143 exm710767 G A INTS8 4 16

8 95897725 exm710819 C A CCNE2 1 2

8 97244011 exm711027 C A UQCRB 8 18

8 97270767 exm711097 G A MTERF3 15 36

8 97797213 exm711209 G A CPQ 0 2

8 97797231 exm711210 G A CPQ 15 35

8 97892201 exm711242 C G CPQ 4 8

8 98041637 exm711252 A G CPQ 0 1

8 98155253 exm711267 G A CPQ 14 32

8 98943205 exm711485 A G MATN2 19 41

8 99030247 exm711596 A C MATN2 1 7

8 99146829 exm711808 A G POP1 2 8

8 99169852 exm711874 G A POP1 6 20

8 99170131 exm711890 C G POP1 0 1

8 100147926 exm712134 A G VPS13B 5 4

8 100286553 exm712223 T A VPS13B 11 19

8 100494026 exm712292 G C VPS13B 9 22

8 100791188 exm712451 A G VPS13B 3 7

8 100861113 exm712539 A T VPS13B 3 19

8 100865852 exm712554 A G VPS13B 11 29

8 100866361 exm712582 G A VPS13B 4 8

8 100874030 exm712597 A G VPS13B 1 8

8 100887752 exm712636 G A VPS13B 3 8

8 101011594 exm712697 A G RGS22 4 14

8 101011612 exm712699 A G RGS22 35 52

8 101014581 exm712707 G A RGS22 10 28

8 101051163 exm712733 A G RGS22 4 7

8 101051173 exm712735 G A RGS22 7 23

8 101059805 exm712751 G C RGS22 2 14

8 101149809 exm712827 C A FBXO43 2 4

8 101178106 exm712904 A G SPAG1 2 3

8 101190141 exm712911 A G SPAG1 1 4

8 101190156 exm712912 G A SPAG1 2 11

8 101196191 exm712921 A G SPAG1 0 1

8 101609014 exm713150 C A SNX31 7 18

8 101620783 exm713161 A C SNX31 15 25

8 101625250 exm713168 A G SNX31 1 4

8 104390349 exm714138 A G CTHRC1 5 13

8 104390460 exm714142 A G CTHRC1 1 5

8 104394704 exm714143 A G CTHRC1 7 14

8 104394710 exm714144 A C CTHRC1 16 36

8 104415504 exm714178 A G SLC25A32 3 2

8 104417045 exm714182 A G SLC25A32 13 26

8 104432659 exm714250 A G DCAF13 19 32

8 104453836 exm714295 A G DCAF13 0 2

8 104897606 exm714344 C G RIMS2 2 2

8 104897687 exm714351 T A RIMS2 13 37

8 104897852 exm714357 G A RIMS2 17 34

8 105001543 exm714455 A G RIMS2 7 13

8 105001576 exm714459 A G RIMS2 0 1

8 105503152 exm714724 C A LRP12 5 9

8 105510131 exm714780 C A LRP12 2 6

8 106814181 exm714891 G C ZFPM2 7 14

8 106814811 exm714911 G A ZFPM2 26 58

8 106814997 exm714916 A G ZFPM2 0 2

8 108334162 exm715137 A C ANGPT1 2 2

8 108334192 exm715140 G A ANGPT1 2 4

8 110283256 exm715381 G A NUDCD1 22 58

8 110283353 exm715385 C A NUDCD1 22 58

8 110397780 exm715475 G A PKHD1L1 19 41

8 110412397 exm715520 G A PKHD1L1 1 4

8 110454373 exm715666 A G PKHD1L1 13 30

8 110455229 exm715671 G A PKHD1L1 4 5

8 110457131 exm715693 A G PKHD1L1 5 9

8 110461642 exm715735 A G PKHD1L1 4 6

8 110467070 exm715779 G A PKHD1L1 1 7

8 110471889 exm715790 A G PKHD1L1 2 7

8 110476470 exm715808 A G PKHD1L1 5 12

8 110477352 exm715838 C A PKHD1L1 0 1

8 110477472 exm715843 A G PKHD1L1 0 1

8 110478811 exm715846 C G PKHD1L1 14 28

8 110492352 exm715884 A G PKHD1L1 6 17

8 110499005 exm715907 A G PKHD1L1 0 1

8 110523152 exm715975 C G PKHD1L1 10 24

8 110535565 exm716010 A G PKHD1L1 0 1

8 113277705 exm716248 T A CSMD3 4 14

8 113303799 exm716282 G A CSMD3 27 42

8 113504810 exm716439 A G CSMD3 0 1

8 113563027 exm716472 C A CSMD3 18 40

8 113668545 exm716541 A G CSMD3 1 14

8 113694722 exm716547 G A CSMD3 3 6

8 113702099 exm716566 A T CSMD3 1 2

8 113960037 exm716596 A C CSMD3 1 5

8 113988161 exm716606 A G CSMD3 4 12

8 114449037 exm716665 A G CSMD3 6 17

8 116426736 exm716679 A G TRPS1 8 10

8 116599271 exm716705 A G TRPS1 7 8

8 116617001 exm716754 A G TRPS1 3 6

8 116631970 exm716786 A T TRPS1 2 3

8 118170004 exm717031 A G SLC30A8 10 15

8 118174044 exm717037 A G SLC30A8 2 5

8 118184886 exm717060 A G SLC30A8 2 12

8 119945383 exm717266 A G TNFRSF11B 0 3

8 120430496 exm717375 G A CCN3 6 7

8 120435251 exm717390 A G CCN3 6 19

8 120581603 exm717436 G A ENPP2 1 7

8 120583076 exm717444 A G ENPP2 3 24

8 121238907 exm717882 G A COL14A1 5 8

8 121290713 exm717961 A G COL14A1 1 3

8 121292281 exm717971 A G COL14A1 7 20

8 121467679 exm718136 T A MTBP 1 3

8 121519002 exm718189 A G MTBP 13 38

8 121530200 exm718218 A G MTBP 11 20

8 123964774 exm718408 A C ZHX2 12 27

8 123964813 exm718411 G A ZHX2 0 2

8 123964819 exm718412 A G ZHX2 2 6

8 123965305 exm718447 A G ZHX2 5 4

8 123965365 exm718449 A G ZHX2 0 2

8 123966052 exm718489 G A ZHX2 6 17

8 123966085 exm718494 A G ZHX2 33 63

8 124105856 exm718582 A G TBC1D31 10 23

8 124138340 exm718623 G A TBC1D31 14 25

8 124243936 exm718807 C G C8orf76;ZHX1-C8orf76 1 1

8 124251302 exm718815 A G C8orf76;ZHX1-C8orf76 6 6

8 124346156 exm718957 G A ATAD2 23 42

8 124351667 exm718971 A G ATAD2 0 4

8 124357302 exm718975 A G ATAD2 5 10
[truncated: 449,119 more chars]
